# Electrochemical hydroxylation of alkenes with H_2_O

**DOI:** 10.1093/nsr/nwag047

**Published:** 2026-01-24

**Authors:** Guoqing Yang, Jingpei Jia, Yuan Deng, Youai Qiu

**Affiliations:** State Key Laboratory and Institute of Elemento-Organic Chemistry, Frontiers Science Center for New Organic Matter, Haihe Laboratory of Sustainable Chemical Transformations, College of Chemistry, Academy for Advanced Interdisciplinary Studies, Nankai University, Tianjin 300071, China; State Key Laboratory and Institute of Elemento-Organic Chemistry, Frontiers Science Center for New Organic Matter, Haihe Laboratory of Sustainable Chemical Transformations, College of Chemistry, Academy for Advanced Interdisciplinary Studies, Nankai University, Tianjin 300071, China; State Key Laboratory and Institute of Elemento-Organic Chemistry, Frontiers Science Center for New Organic Matter, Haihe Laboratory of Sustainable Chemical Transformations, College of Chemistry, Academy for Advanced Interdisciplinary Studies, Nankai University, Tianjin 300071, China; State Key Laboratory and Institute of Elemento-Organic Chemistry, Frontiers Science Center for New Organic Matter, Haihe Laboratory of Sustainable Chemical Transformations, College of Chemistry, Academy for Advanced Interdisciplinary Studies, Nankai University, Tianjin 300071, China; State Key Laboratory of Coordination Chemistry, School of Chemistry and Chemical Engineering, Nanjing University, Nanjing 210093, China

**Keywords:** organic electrosynthesis, hydroxylation, alkenes, water, cobalt catalysis

## Abstract

Alcohol is one of the most prevalent functional groups found in biologically active molecules with unparalleled abundance and structural diversity. The hydroxylation reaction of alkenes represents a convenient approach for synthesizing such molecules. However, a general and direct method featuring a broad substrate scope and good functional group tolerance remains elusive. Herein, we report our efforts in applying electrochemistry to accomplish the hydroxylation of alkenes utilizing H_2_O as the sole source of hydroxyl without the need for stoichiometric oxidants and strong acidic reagents. This process operates via the formation of a high-valence cobalt(IV) intermediate, which could readily be entrapped by water to deliver the desired alcohol product. Mechanistic studies, cyclic voltammetry analysis and density functional theory calculations have been conducted to verify the feasibility of cobalt valence changes, including the generation of cobalt hydride species, the formation of intermediates following hydrogen atom transfer, the oxidation potential from Co(III) to Co(IV), which substantiate the proposed reaction pathways. Furthermore, the late-stage modification of biorelevant molecules has demonstrated promising potential applications for this approach in synthetic chemistry.

## Teaser

Electrochemical hydroxylation of diverse alkenes with H_2_O as the hydroxyl source demonstrates promising potential applications in synthetic chemistry.

## INTRODUCTION

Alcohol is a privileged synthetic motif and functional group that widely exists in biologically active molecules and pharmaceutical compounds [1–3]. They can also be key intermediates in synthetic chemistry and used as solvents in organic synthesis and extraction processes; hence it is of great importance and significance to develop efficient and general methods to access them. Typical methods [4–8] to access alcohols include hydrolysis of halogenated hydrocarbons [6], reduction of carbonyl compounds [7], and the reaction of Grignard reagents with carbonyl compounds [8]. However, the generality of these classic reactions varies from case to case due to their uncertain reactivity and different sources of reactant. From the perspective of atom economy and accessibility, the hydroxylation of different types of alkenes under mild conditions provides a straightforward and highly attractive strategy for the synthesis of alcohols.

**Figure 1.**
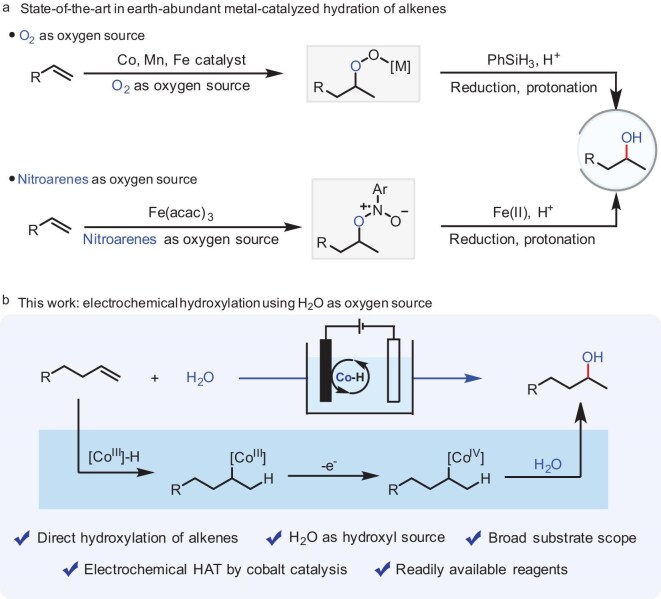
Catalyzed hydroxylation of alkenes.

Notably, the hydroxylation of alkenes could be used to obtain the corresponding alcohols [9,10], for example, ethanol, has been prepared industrially on a large scale. Meanwhile, typical hydroxylation reactions of alkenes using H_2_O as the oxygen source to obtain alcohols described in organic chemistry textbooks usually include hydration reactions and oxymercuration-reduction reactions [11–13]. The hydration process normally occurs through a carbocation intermediate stoichiometrically using a strong acid catalyst. However, the mechanism involving carbocation intermediates often leads to rearrangement side reactions [14]. Concurrently, significant drawbacks of oxymercuration-reduction reactions include the stoichiometric requirements of mercuric acetate and complex processes, both of which reduce the practicality of the protocols [15]. Notably, the Mukaiyama-type hydration of alkene provided a practical approach to the synthesis of alcohols with O_2_ as the oxygen source in the presence of first-row transition metals (Co/Mn/Fe); the key peroxy intermediate is formed by insertion of oxygen, followed by reduction and protonation to afford the alcohols (Fig. 1a, top) [16–20]. Recently, Studer’s group [21] further developed anaerobic Mukaiyama-type hydration of alkene with nitroarenes as the oxygen source (Fig. 1a, bottom). Despite these advances, the development of a greener and facile method for hydroxylation of alkenes using H_2_O as the oxygen source under mild reaction conditions remains highly desirable.

Over the past few decades, electrochemical strategies have received great attention as an alternative and environmentally friendly method for sustainable transformations in modern organic synthesis [22–33]. Among these, electrochemical conversion of organic compounds with small molecules have attracted considerable interest [34], including CO_2_ [35–37], H_2_O [38–44], NH_3_ [45,46], and others [47–50], which could be used as ideal functional group precursors for organic synthesis. With our continuing interest in electrochemical transformations of water [51,52], we sought to explore whether the electrochemical hydroxylation of alkenes could be achieved. Several inspiring reports have shown that the electrochemical cobalt-catalyzed hydrogen atom transfer (HAT) process exhibits promising reactivity [53–69]; however, the use of less reactive but more easily available water as an ideal hydroxyl source still remains relatively underdeveloped. The primary challenges and difficulties lie in the significantly weaker nucleophilicity of water compared to alcohols, phenols, and carboxylic acids, followed by selectivity control of the whole process with potentially diverse reactivities derived from the cobalt intermediates and possible over-oxidation of the alcohol products.

Herein, we report our efforts in moving this reaction forward by realizing electrochemical hydroxylation of alkenes with H_2_O. This approach uses readily available cobalt catalysts and reagents, delivering diversified alcohols with excellent regioselectivity (Fig. 1b). Salient features of our method include: (1) direct hydroxylation of alkenes with H_2_O; (2) electrochemical HAT by cobalt catalysis; (3) water is used as an economical, eco-friendly, and easily available hydroxyl source; (4) it is a general method that tolerates unactivated alkenes; (5) late-stage functionalization of bioactive molecules can be achieved, which offers a potential application in synthetic chemistry, pharmaceutical science, and other fields.

## RESULTS AND DISCUSSION

### Screening reaction conditions

We commenced our study by screening various reaction parameters for the hydroxylation of 4-(pent-4-en-1-yloxy)-1,1′-biphenyl **1a** with H_2_O (Table 1). The model reaction conditions were carried out in an undivided cell with a graphite felt (GF) anode and a platinum plate (Pt) cathode under 5.0 mA constant current electrolysis (CCE). With the addition of a cobalt catalyst (Co-1), silane (Ph_2_SiH_2_), and electrolyte (TBABF_4_), we could obtain the target product **1** in 92% isolated yield (Table 1, entry 1). Next, key observations were made by evaluating different types of catalysts. We discovered that the steric effects of the R^2^ groups on the cobalt complex have a significant impact on the reaction. When R^2^ = H, regardless of the electronegativity of the substituents on the R^1^ group, no target product was generated (see Supplementary Information, Table S1). Yet, when R^2^ = ^*t*^Bu, R^1^ = H, it could give the target product in 16% yield (Table S1, entry 2). Subsequently, the investigation focused on the diamine substructure within the catalyst and it was found that the application of cyclohexyl or *ortho*-biaryl in cobalt complex resulted in a sharp decline in the yield (Table S1, entries 3 and 4). We also replaced the central metal with iron or manganese, but there was no observable reaction (Table 1, entries 2 and 3). In addition, different silanes were evaluated (Table 1, entries 4 and 5), among which Ph_2_SiH_2_ was found to be the best hydrogen donor, providing **1** in good yield. Screening various solvents revealed that alcoholic solvents are the optimal choice, which may facilitate the formation of a solvent-caged radical pair [55,61], whereas *N,N*-dimethylformamide (DMF) leads to no reaction (Table 1, entry 6). Other conventional reaction parameters were further optimized by changing the current magnitude and replacing the electrode materials. No significant influence on the yield was observed upon using 3 mA or 10 mA or when using a platinum plate instead of graphite felt, both resulting in a diminished yield (Table 1, entries 7–9). Notably, reducing the amount of catalyst to 1 mol% achieved moderate yield (Table 1, entry 10). Control experiments showed that the reaction cannot proceed in the absence of electricity, silane, or cobalt catalyst (Table 1, entries 11 and 12).

**Table 1.** Screening of the reaction conditions.^a^

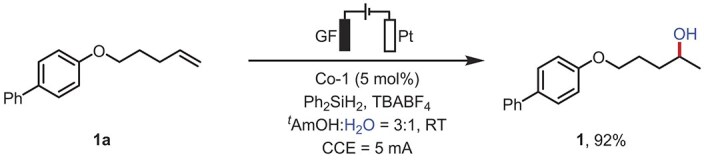		
Entry	Variation from standard conditions^a^	Yield of **1** (%)^b^
**1**	**None**	**92**
2	Fe-1 instead of Co-1	N.R.
3	Mn-1 instead of Co-1	N.R.
4	PhSiH_3_ instead of Ph_2_SiH_2_	41
5	Ph(CH_3_)_2_SiH instead of Ph_2_SiH_2_	64
6	^*i*^PrOH/HFIP/DMF instead of ^*t*^AmOH	32/60/trace
7	3 mA instead of 5 mA	88
8	10 mA instead of 5 mA	90
9	Pt(+) instead of GF(+)	25
10	Co-1 (1 mol%)	54
11	No Ph_2_SiH_2_	N.R.
12	No current or cobalt catalyst	N.R.
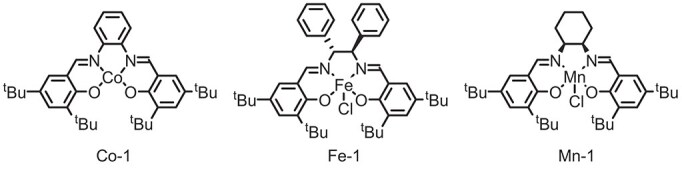		

^a^Reaction conditions: undivided cell, **1a** (0.2 mmol), Co-1 (0.01 mmol, 5 mol%), Ph_2_SiH_2_ (0.4 mmol, 2.0 equiv.), TBABF_4_ (0.2 mmol, 1.0 equiv.) in ^*t*^AmOH:H_2_O = 3:1 (4.0 mL), room temperature, 12 h, under Ar, graphite felt as the anode, and platinum plate as the cathode, CCE = 5.0 mA, N.R. = no reaction. ^b^Isolated yield.

**Figure 2.**
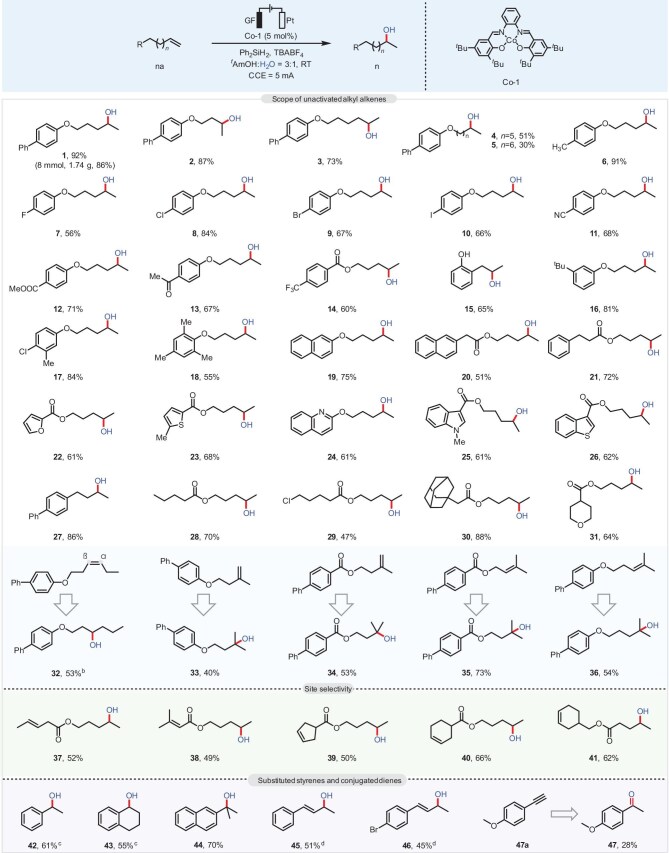
Scope of the reaction with unactivated alkyl alkenes. *^a^*Reaction conditions: alkene (0.2 mmol), Co-1 (0.01 mmol, 5 mol%), Ph_2_SiH_2_ (0.4 mmol, 2.0 equiv.), TBABF_4_ (0.2 mmol, 1.0 equiv.) in *^t^*AmOH:H_2_O = 3:1 (4.0 mL), room temperature, 12 h, under Ar, graphite felt as the anode, and platinum plate as the cathode, CCE = 5.0 mA. *^b^ α*:*β* = 1:7.6. *^c^* 0.3 mmol, *^d^* Ph_2_SiH_2_ (3.0 equiv.), 3 h.

With the optimized conditions established, we then explored the scope and generality of alkenes in the reaction (Fig. 2). Initially, we started with relatively challenging unactivated alkenes, and we found that terminal alkenes with longer chain lengths (*n* = 2 to 6) (**2**–**5**) gave the corresponding hydroxylated products in moderate to good yields. A wide range of terminal alkenes with aromatic rings containing both electron-rich (**6**) and electron-withdrawing groups (**7**–**14**), such as F, Cl, Br, I, COOMe, CN and CF_3_, can be well-tolerated to afford the product in good to excellent yield. Furthermore, a substrate with OH substituent at the *ortho*-position of the benzene ring (**15**), a ^*t*^Bu substituent at the *meta*-position of the benzene ring (**16**), a disubstituted or trisubstituted benzene ring process (**17** and **18**), or a naphthalene ring (**19**) all worked smoothly in this reaction. Besides, we found that the substrates derived from acids can also accommodate functional groups such as CF_3_ (**14**). Notably, the presence of an ester group within the molecule did not negatively impact the reactivity of these compounds (**20** and **21**). Of note, a variety of heterocyclic rings (**22**–**26**), including furan, thiophene, quinoline, *N*-methyl indole, and benzothiophene, were all well tolerated. Next, we found that a terminal alkene without O atoms could also be successfully employed in the reaction (**27**). We also realized that the standard reaction conditions could be applied to the hydroxylation of aliphatic alkenes with an ester group (**28**–**31**), such as valeric acid and 5-chlorovaleric acid derivatives, to give the corresponding product **28** (70%) and **29** (47%), respectively. Notably, using our method, the internal alkene (**32**) and poly-substituted alkenes (**33**–**36**) could be successfully engaged in the hydroxylation reaction with H_2_O.

To further probe the site selectivity of this transformation, we tested the hydroxylation of alkenes in substrates bearing more than one potential C=C site for functionalization. In general, we found there was a preference for hydroxylation to occur on the C=C bond located at the conjugated position of the benzene ring and at the terminal position. For example, for substrates (**37**–**41**) containing an internal alkene or poly-substituted alkene, as well as a terminal alkene, the terminal site was selectively functionalized in all of the cases. According to subsequent mechanistic studies and possible reaction mechanisms, the reason is attributed to the sluggish Co–H addition step in the case of increased steric hinderance and the stability of the carbon radicals generated after hydrogen atom transfer. Importantly, due to the high utility of α-methylbenzyl alcohol and allyl alcohol derivatives, this protocol was further extended to the hydroxylation of substituted styrenes and phenyl-substituted 1,3-dienes. As shown in Fig. 2, simple styrenes (**42**) could work smoothly under standard conditions to give the hydroxylation product in moderate to good yields. Besides, dihydronaphthalene (**43**) and the hindered 2-isopropenylnaphthalene (**44**) were subjected to the same standard conditions and afforded the desired hydroxylation products. Notably, 1-phenyl-1,3-butadiene and its derivatives (**45**–**46**) reacted successfully to give 1,2-difunctionalization products in moderate yield, while the phenylacetylene substrates yielded the corresponding ketone products (**47**) thus showing the excellent applicability of this strategy.

**Figure 3.**
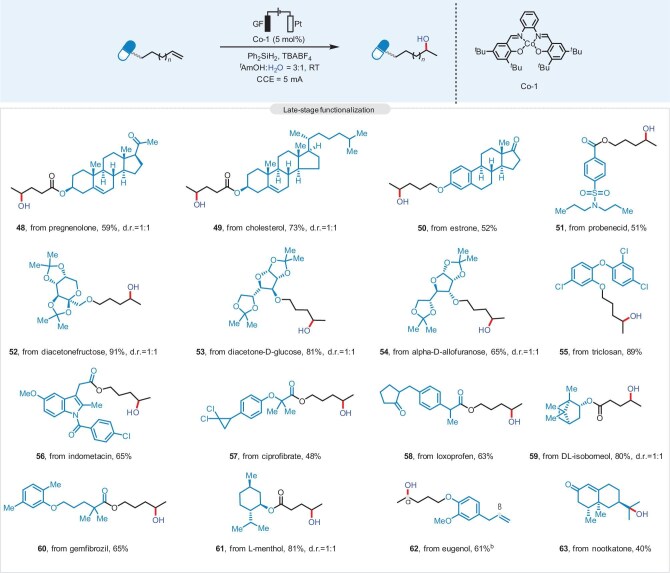
Scope of late-stage modification of biorelevant compounds. *^a^*Reaction conditions: alkene (0.2 mmol), Co-1 (0.01 mmol, 5 mol%), Ph_2_SiH_2_ (0.4 mmol, 2.0 equiv.), TBABF_4_ (0.2 mmol, 1.0 equiv.) in *^t^*AmOH:H_2_O = 3:1 (4.0 mL), room temperature, 12 h, under Ar, graphite felt as the anode, and platinum plate as the cathode, CCE = 5.0 mA.*^b^ α*:*β* = 6:1.

Considering the crucial role of alcohol compounds in natural products and pharmaceutical molecules, this protocol was further extended to the hydroxylation of complex compounds (Fig. 3). Unactivated alkenes derived from pregnenolone (**48**), cholesterol (**49**), estrone (**50**), probenecid (**51**), diacetonefructose (**52**), diacetone-*D*-glucose (**53**), α-*D*-allofuranose (**54**), triclosan (**55**), indometacin (**56**), ciprofibrate (**57**), loxoprofen (**58**), *DL*-isoborneol (**59**), gemfibrozil (**60**), *L*-menthol (**61**), and eugenol (**62**) were all suitable for this method and provided the desired hydroxylated products in moderate to good yields. Surprisingly, this strategy could also be proficiently applied to the direct hydroxylation of a natural product, nootkatone (**63**), which demonstrates the versatility of our electrochemical system and offers a potential synthetic method for the introduction of a hydroxyl group to some complex natural compounds and drugs.

### Study on electrochemical hydroxylation reaction and mechanism

**Figure 4.**
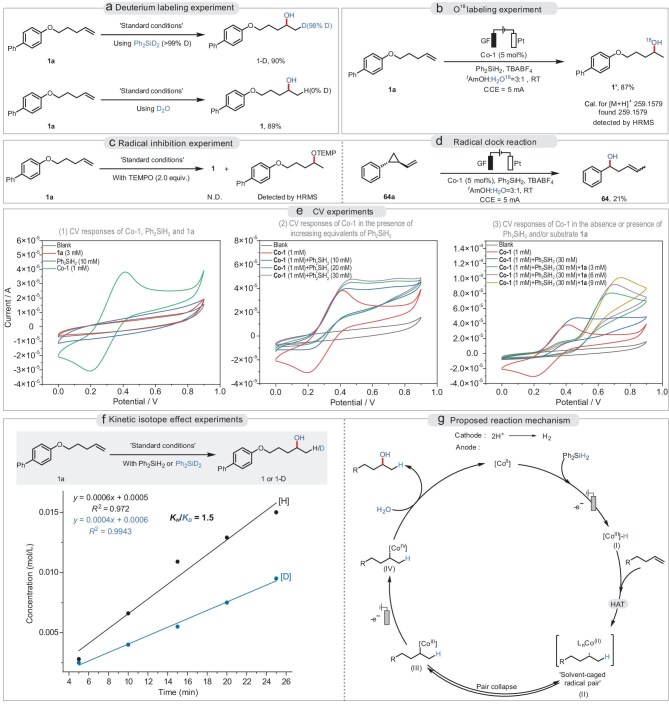
Mechanistic studies. (a) Deuterium labeling experiment. (b) O^18^ labeling experiment. (c) Radical inhibition experiment. (d) Radical clock reaction. (e) CV experiments. (f) Kinetic isotope effect experiments. (g) Proposed reaction mechanism.

In addition, the scalability of the reaction up to the gram scale was confirmed. Gratifyingly, the target product **1** could be obtained in 86% yield with only 1 mol% catalyst loading. To gain insights into the mechanism of this transformation, several mechanistic experiments were carried out. Initially, we investigated the sources of hydrogen and hydroxyl groups in the product. We conducted a deuterium-labeling experiment using Ph_2_SiD_2_ and D_2_O, which resulted in **1-D** in 90% yield with >90% D incorporation and **1** in 89% yield with 0% D incorporation, providing evidence that the hydrogen atom came from the silane reagent (Fig. 4a). Subsequently, an ^18^O labeling experiment (Fig. 4b) showed that the hydroxyl group of the product **1’** was from water in the reaction system (detected by high-resolution mass spectrometry (HRMS)). Furthermore, the catalytic system of **1a** was completely inhibited in the presence of stoichiometric (2,2,6,6-tetramethylpiperidin-1-yl)oxyl (TEMPO), and the corresponding TEMPO-trapped product was detected (Fig. 4c), which suggests an alkyl radical is probably involved in this transformation. Next, radical clock experiments (Fig. 4d) using an alkene with a cyclopropyl motif showed that the substrate underwent a ring-opening process and provided the hydroxylation product (**64**) in 21% yield, which is consistent with the radical characteristics of an intermediate generated by HAT as hypothesized. A study on the deuterium kinetic isotope effects (KIEs) was also conducted. The *k*_H_/*k*_D_ value was found to be 1.5, revealing a secondary kinetic deuterium effect (Fig. 4f).

Thereafter, more detailed mechanistic studies were conducted by means of cyclic voltammetry (CV) to enable in-depth analysis of the change in valence state of the catalyst. We first performed CV studies on the substrate of **1a**, Co-1, and silane. The Co-1 catalyst exhibited a Co(II/III) redox couple (Fig. 4e-(1), green line), with an oxidation peak at 0.42 V vs Ag/AgCl and a reduction peak at 0.20 V vs Ag/AgCl. In contrast, substrate **1a** and silane did not exhibit any oxidative behavior. After adding increasing equivalents of Ph_2_SiH_2_ (Fig. 4e-(2)), we observed that the cobalt catalyst only exhibited an irreversible oxidation peak, which we interpreted as the formation of a putative cobalt hydride species. Notably, based on the CV response (Fig. 4e-(2)), with the addition of substrate **1a**, the oxidation peak of the cobalt catalyst significantly decreased, while a new irreversible oxidation peak appeared at 0.68 V vs Ag/AgCl. Meanwhile, the second oxidation peak gradually increased with the incremental addition of substrate in equimolar amounts (Fig. 4e-(3)). After ruling out the possibility of direct oxidation of the substrate, it can be inferred that the new oxidation peak likely corresponds to a species or intermediate formed during the interaction between the substrate and the cobalt catalyst. According to existing literature reports [61–64], the cobalt hydride species undergo metal-hydrogen atom transfer with the alkene, simultaneously forming a ‘solvent-cage radical pair’, which leads to the formation of carbon-centered radicals or alkylcobalt complexes as the cage escaped or pair collapsed. Density functional theory (DFT) calculation results reveal that overall the conversion of **1a** and intermediate (**I**) is relatively thermoneutral. However, intermediate (**III**) is thermodynamically more favourable (see Fig. S15). Computational analysis further reveals an oxidation potential of 0.55 V vs Ag/AgCl for the transformation of the alkyl-Co(III) complex to its high-valence Co(IV) counterpart (see Fig. S16). To validate this prediction, we synthesized an analogous alkyl-Co(III) complex (for more details, please see the Supplementary Information S21) and conducted cyclic voltammetry measurements (see Fig. S14). The observed oxidation peak at 0.61 V aligns with the pathway outlined in Fig. 4e-(3). These experimental results strongly support our mechanistic proposal that the second oxidation event corresponds to the oxidation of the Co-alkyl intermediate (III) to the high-valence Co(IV) species (IV).

In accordance with our experimental results and literature reports [65–69], a possible mechanism was proposed for the electrochemical hydroxylation of alkenes with H_2_O (Fig. 4g). Initially, this transformation is induced by the formation of a cobalt hydride species (**I**), which involves the oxidation of the Co(II) catalyst and rapid combination with Ph_2_SiH_2_. Then, the cobalt hydride species (**I**) undergoes HAT with the alkene substrate to generate a radical pair, which is known as a ‘solvent-caged radical pair’. Subsequent collapse of the radical pair generates the alkylcobalt (III) complex (**III**), which then undergoes a second electrochemical oxidation step to form the key alkylcobalt (IV) intermediate (**IV**). Finally, this species is rapidly captured by H_2_O to yield the desired hydroxylation product and regenerates the Co(II) complex (for more details for DFT calculations, please see Fig. S17).

## CONCLUSION

In conclusion, we have successfully accomplished electrochemical hydroxylation of alkenes with H_2_O. This method utilizes readily available alkenes and eco-friendly H_2_O along with an earth-abundant cobalt catalyst, enabling direct hydroxylation of diverse alkenes through a general electrochemical Co(II/III/IV) process. The electrooxidation realizes two key elementary steps in the cobalt catalytic cycle: (1) cobalt-hydride generation by electrochemical oxidation of the cobalt precursor and (2) the oxidation of the alkyl Co(III) complex to a key alkyl Co(IV) intermediate. Furthermore, this process is compatible with a variety of sensitive groups and challenging unactivated alkenes, including phenyl-substituted 1,3-diene. Detailed mechanistic studies and control experiments further substantiated our proposed mechanism. We anticipate that this electrochemical protocol will provide a new avenue for the synthesis of alcohols and the late-stage derivatization of biorelevant compounds.

## METHODS

### General procedure of electrochemical hydroxylation of alkyl alkenes with H_2_O

Electrocatalysis was carried out in an undivided cell with graphite felt electrodes (10 mm × 20 mm × 5 mm) and platinum electrodes (10 mm × 15 mm × 0.25 mm). To a 15.0-mL oven-dried undivided cell equipped with a magnetic bar we added substrates of alkene (0.2 mmol, 1.0 equiv.), Co-1 (0.01 mmol, 5 mol%), and TBABF_4_ (0.2 mmol, 1.0 equiv.). The tube was then evacuated and back-filled under Ar flow (this procedure was repeated three times), and 2-methyl-2-butanol (3.0 mL), H_2_O (1.0 mL) and Ph_2_SiH_2_ (0.4 mmol, 2.0 equiv.) were added successively via a syringe. The electrocatalysis was performed at room temperature with a constant current of 5 mA maintained for 12 h. After that, the electrodes were washed with EtOAc (5 mL × 3) in an ultrasonic bath. Then H_2_O (10 mL) was added to the system, and the resulting mixture was extracted with EtOAc (10 mL × 3). The combined organic phase was washed with a saturated solution of NaCl (10 mL × 3) and dried with anhydrous Na_2_SO_4_, filtered, and concentrated *in vacuo*. The crude product was purified by column chromatography to furnish the desired product.

## Supplementary Material

nwag047_Supplemental_File
