# Supplementary material for: Electrochemical hydroxylation of alkenes with H2O
Source: Natl Sci Rev. 2026 Jan 24;13(6):nwag047. doi: 10.1093/nsr/nwag047 (PMC13045720; doi:10.1093/nsr/nwag047)

Supplementary Information

Electrochemical Hydroxylation of Alkenes with H_2O

Guoqing Yang, Jingpei Jia, Yuan Deng and Youai Qiu*

State Key Laboratory and Institute of Elemento-Organic Chemistry, Frontiers Science Center for New Organic Matter, Haihe Laboratory of Sustainable Chemical Transformations, College of Chemistry, Academy for Advanced Interdisciplinary Studies, Nankai University, 94 Weijin Road, Tianjin, 300071, China

***qiuyouai@nankai.edu.cn**

Table of Contents

General Remarks 1
Optimization of the Reaction Conditions[a] 2
Synthesis of starting materials and cobalt catalyst 5
General Procedure of Electrochemical Hydroxylation of Alkenes with H_2O 8
Gram-Scale Reaction 10
Mechanistic Studies 10
Cyclic Voltammetry Studies 22
DFT Calculations 26
References 48
Characterization Data of Products 50
Copies of ^{1}H, ^{13}C and ^{19}F NMR Spectra for Compounds 77

General Remarks

The reactions were carried out in undivided electrochemical cells (15 mL) using pre-dried glassware, if not noted otherwise. The substrates were obtained from commercial sources (4-Phenylphenol; CAS = 92-69-3, Cat No.1046840, Leyan, Shanghai, China) and benzoic acid derivatives were purchased from J&K Scientific, TCI, Bidepharm, Energy Chemical, Alfa Aesar, and used as received) or synthesized according to literature methods.[1-2] Solvents were obtained from commercial sources. Graphite felt electrodes (10 mm × 20 mm × 5 mm, obtained from Guangjiayuan electronic materials, Jiangsu, China) and Platinum electrodes (10 mm × 15 mm × 0.25 mm, 99.9%; obtained from Chuxi, Shanghai, China) were connected using stainless steel adapters. Electrocatalysis was conducted using an HSPY-36-03 potentiostat in constant current mode. Cyclic Voltammetry studies were performed using a Shanghai Chenhua CHI760E workstation. Yields refer to isolated compounds, estimated to be >95% purity as determined by ^{1}H NMR. Flash chromatography was performed using Silica gel (200 – 300 mesh) purchased from Qingdao Haiyang Chemical Co., China. NMR spectra were recorded on Bruker AVANCE AV 400 or 600 in the solvent indicated; using $CDCl_3$ as the solvent with tetramethylsilane (TMS) as the internal standard at room temperature, chemical shifts (δ) are given in ppm relative to the residual solvent peak, coupling constants (J) are reported in Hertz (Hz). Multiplicities are recorded as: s = singlet, d = doublet, t = triplet, q = quadruplet, dd = doublet of doublets, m = multiplet. The High-resolution mass spectrometry (HRMS) data were collected on a Micro TOF mass spectrometer with ESI mass analyzer. Melting points were recorded on Shanghai ShenGuang WRS–2 apparatus. Visualization was achieved under a UV lamp (254 nm and 365 nm).

Optimization of the Reaction Conditions[a]

Supplementary Table S1: Cobalt catalyst screening

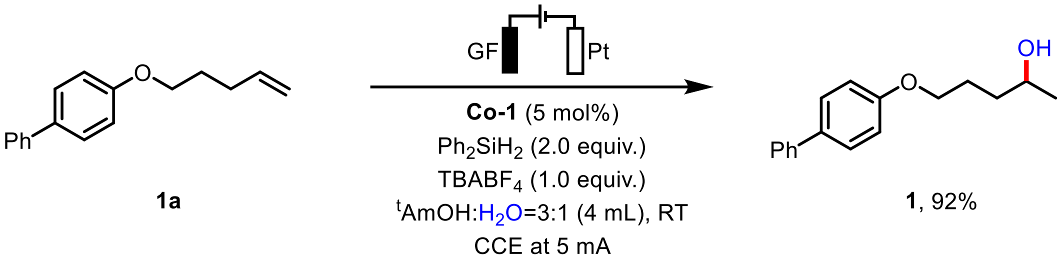

1a

1, 92%

Entry	Alteration	Yield (%)[b]
1	None	92
2	**Co-2**	16
3	**Co-3**	21
4	**Co-4**	45
5	**Co-5**	35
6	**Co-6**	28
7	**Co-7**	22
8	**Co-8/ Co-9/ Co-10**	n.d.
9	**Fe-1/Mn-1**	n.d.

[a] Reaction conditions: undivided cell, **1a** (0.2 mmol), Co-1 (0.01 mmol, 5 mol%), Ph_2SiH_2 (0.4 mmol, 2.0 equiv.), $TBABF_4$ (0.2 mmol, 1.0 equiv.) in ^{t}AmOH : H_2O = 3 : 1 (4.0 mL), room temperature, 12 h, under Ar, graphite felt as the anode, and platinum plate as the cathode, CCE = 5.0 mA, n.d. = not detected.
[b] Isolated yield.

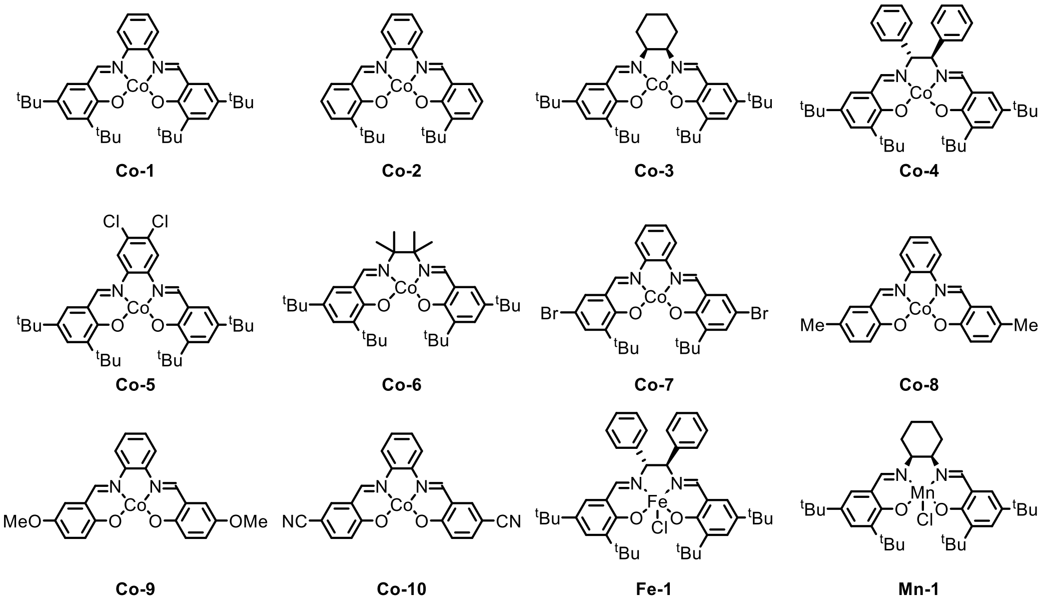

Supplementary Table S2: Other factors screening

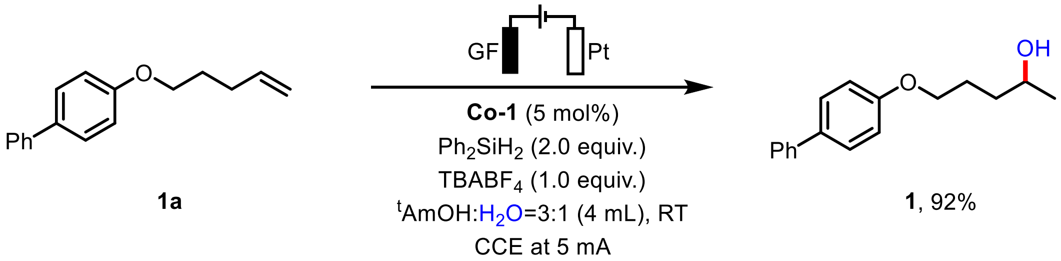

Entry	Alteration	Yield (%) of **1**[b]
1	None	92
2	$PhSiH_3$/$Ph(CH_3)_2SiH$ instead of Ph_2SiH_2	41/64
3	^{i}PrOH/HFIP/DMF instead of ^{t}AmOH	32/60/trace
4	3 mA/10 mA instead of 5 mA	88/90
5	Pt (+) instead of GF (+)	25
6	**Co-1** (1 mol%)	54
7	No H_2O	n.d.
8	No Ph_2SiH_2	n.d.
9	No Current or **Co-1**	n.d.

[a] Reaction conditions: undivided cell, **1a** (0.2 mmol), Co-1 (0.01 mmol, 5 mol%), Ph_2SiH_2 (0.4 mmol, 2.0 equiv.), $TBABF_4$ (0.2 mmol, 1.0 equiv.) in ^{t}AmOH : H_2O = 3 : 1 (4.0 mL), room temperature, 12 h, under Ar, graphite felt as the anode, and platinum plate as the cathode, CCE = 5.0 mA, n.d. = not detected. [b] Isolated yield.

Supplementary Table S3: Different catalyst amounts screening

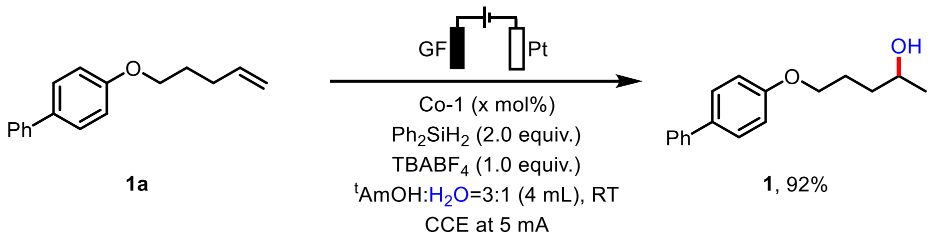

Entry	Variation (x mol%)	Yield of **1** (%)[b]
**1**	**None**	**92**
2	0.75 mol%	32 (60%)
3	1 mol%	54
4	2 mol%	57
5	3 mol%	71
6	4 mol%	69

[a] Reaction conditions: undivided cell, **1a** (0.2 mmol), Co-1 (x mol%), Ph_2SiH_2 (0.4 mmol, 2.0 equiv.), $TBABF_4$ (0.2 mmol, 1.0 equiv.) in ^{t}AmOH : H_2O = 3 : 1 (4.0 mL), room temperature, 12 h, under Ar, graphite felt as the anode, and platinum plate as the cathode, CCE = 5.0 mA. [b] Isolated yield.

Synthesis of starting materials and cobalt catalyst

General procedure for the preparation of substrates[1]:

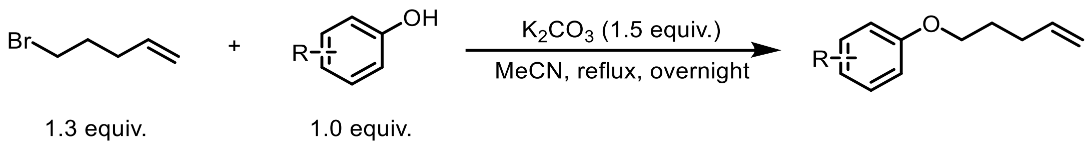

Method A: A mixture of phenol derivatives (10.0 mmol, 1.0 equiv.), 5-bromo-1-pentene (13.0 mmol, 1.3 equiv.), and K_2CO_3 (15.0 mmol, 1.5 equiv.) in anhydrous acetonitrile (30 mL) was heated to reflux in an oil bath. After refluxing overnight, the reaction mixture was cooled to room temperature, concentrated under reduced pressure, diluted with saturated NH_4Cl (20 mL) and extracted with ethyl acetate (20 mL × 3). The combined organic layers were dried with Na_2SO_4, filtered, and concentrated under reduced pressure. The residue was purified by silica gel column chromatography to afford the corresponding pure product (Supplementary Figure S1).

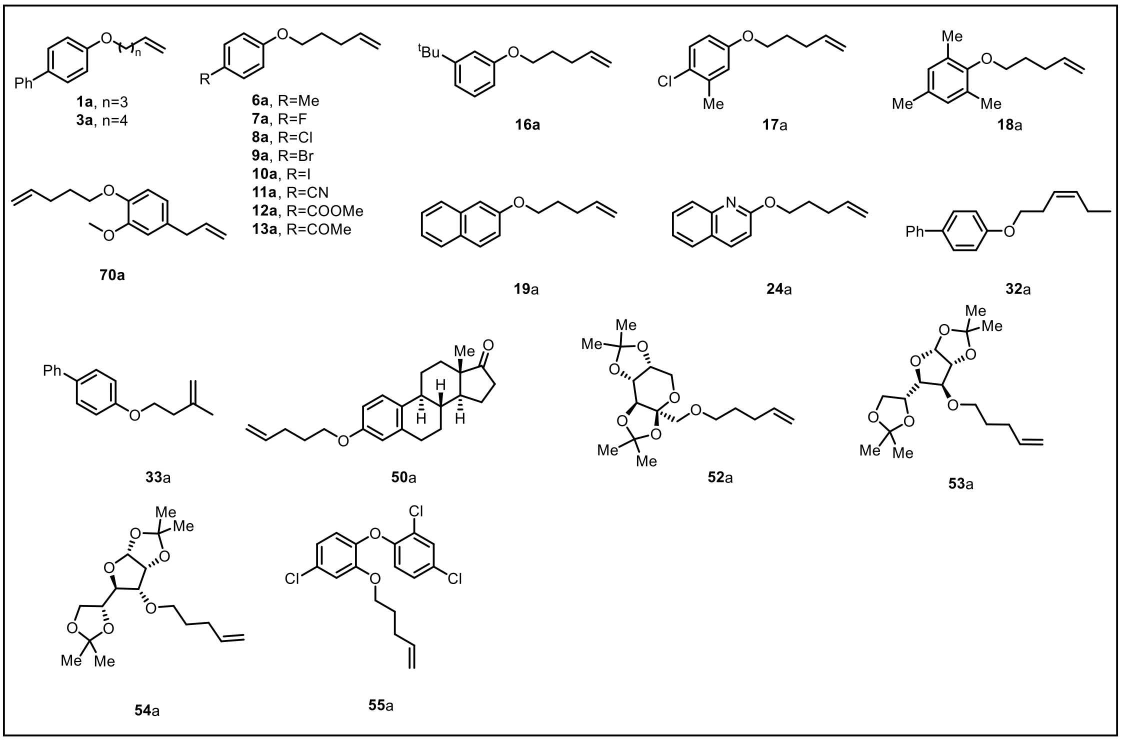

Supplementary Figure S1: Starting materials synthesized according to **Method A**.

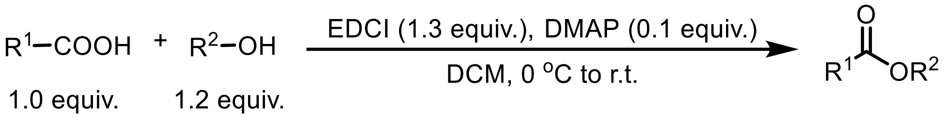

Method B[2]: The corresponding acid (10 mmol, 1.0 equiv.) was added to a solution of 1-(3-dimethylaminopropyl)-3-ethylcarbodiimide (EDCI, 1.3 equiv.) and DMAP (0.1 equiv.) in CH_2Cl_2 (25 mL) at 0 °C. Alcohol (1.2 equiv.) was then added. The reaction mixture was allowed to warm to room temperature overnight. The solution was diluted with CH_2Cl_2 (40 mL) and washed with 1N HCl (20 mL × 3), saturated $NaHCO_3$ (40 mL), brine (40 mL) sequentially. The organic layer was dried over anhydrous Na_2SO_4. After removal of solvent under reduced pressure, the crude product was purified by column chromatography on silica gel (Supplementary Figure S2).

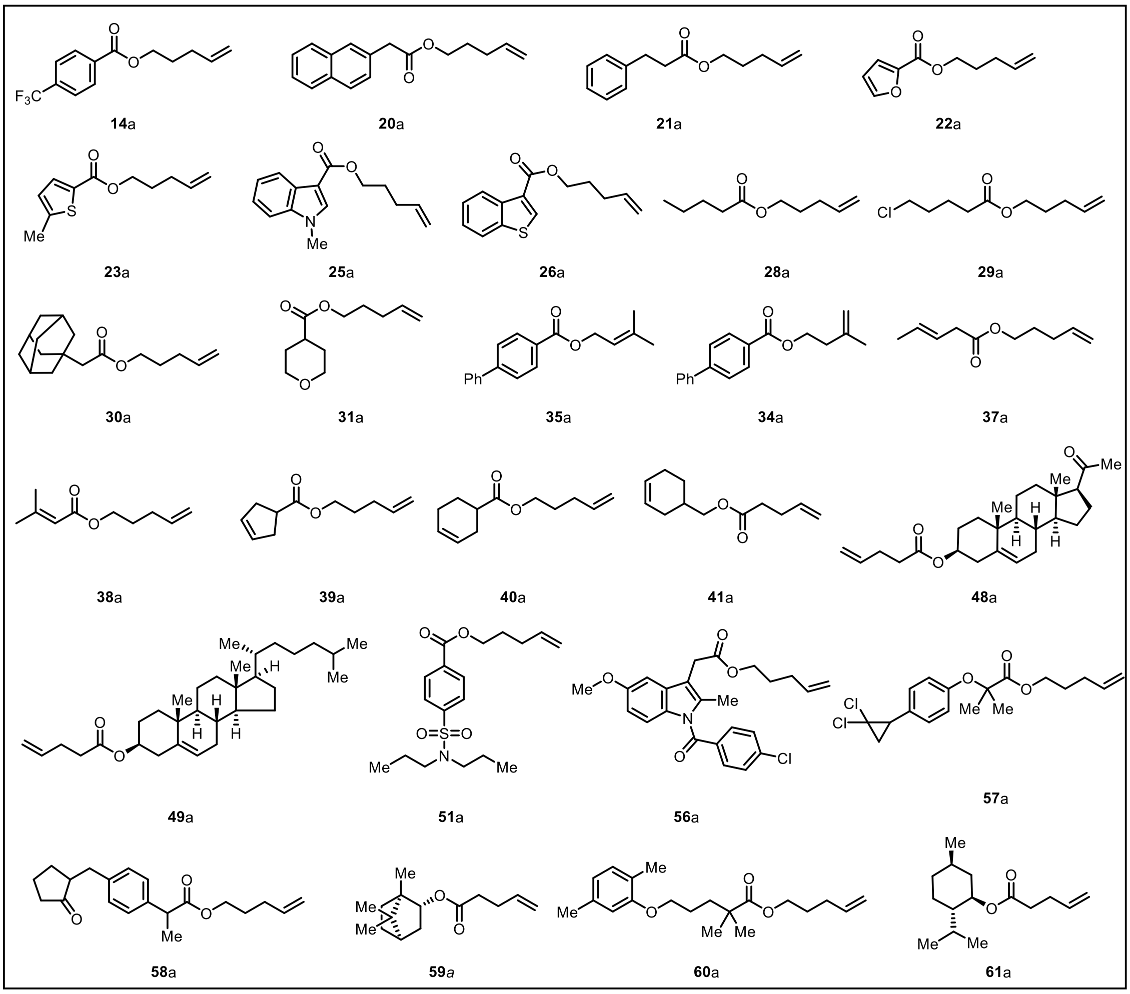

Supplementary Figure S2: Starting materials synthesized according to **Method B**.

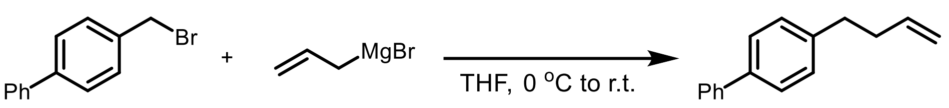

Method C: Benzyl bromide (10 mmol, 1.0 equiv.) and 20 mL anhydrous THF were added to a flame dried 100 mL round-bottom flash assembled with a constant pressure funnel which had been previously flame dried. Allyl magnesium bromide (20 mmol, 2.0 equiv.) in the constant pressure funnel were added dropwise. The reaction was stirred for 4 h at room temperature and then quenched with saturated aqueous NH_4Cl. The aqueous layer was extracted with CH_2Cl_2 (40 mL × 2) and the combined organics were dried over $MgSO_4$, filtered through celite and concentrated under reduced pressure. The crude product was purified by flash chromatography (eluent: hexane), affording the desired product as a clear colorless oil.

General procedure for the preparation of Co-1:

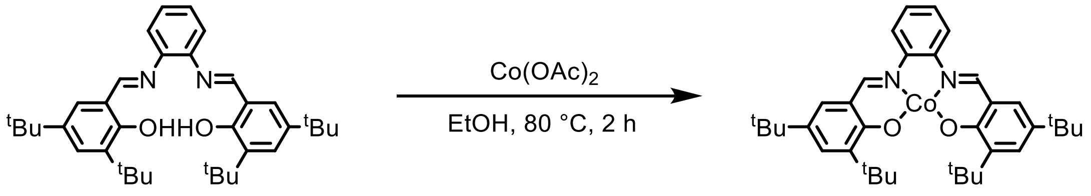

6,6'-((1E,1'E)-(1,2-phenylenebis(azaneylylidene))bis(methaneylylidene))bis(2,4-di-tert-butylphenol) (prepared according to the reported procedure,[4] 541 mg, 1.0 mmol) was dissolved in EtOH (10 mL). Then, a solution of $Co(OAc)_2$ (177 mg, 1.0 mmol) in EtOH (10 mL) was added. The mixture was stirred for 2 hours at 80°C and the precipitate was collected by filtration and washed with cold MeOH. Removal of the residual solvent in vacuo afforded **Co-1** as a dark solid. Characterization data of the obtained product were consistent with the reported one.[5] HRMS (ESI, m/z): Calculated $C_{36}H_{48}N_2O_2$ $[M+H]^+$: 598.2964 , found 598.2968.

Complexes **Co-2, Co-3, Co-4, Co-5, Co-6, Co-7, Co-8, Co-9, Co-10, Fe-1**[6] were prepared according to the representative procedure.[7-8]

General Procedure of Electrochemical Hydroxylation of Alkenes with H_2O

The reaction was carried out in an undivided cell with Graphite felt electrodes (10 mm × 20 mm × 5 mm) and Platinum electrodes (10 mm × 15 mm × 0.25 mm). To a 15.0 mL oven-dried undivided cell equipped with a magnetic bar was added substrates alkene **1a** (0.2 mmol, 1.0 equiv.), **Co-1** (0.01 mmol, 5 mol%), $TBABF_4$ (0.2 mmol, 1.0 equiv.). Then the tube was evacuated and back-filled under Ar flow (this procedure was repeated three times), and 2-Methyl-2-butanol (3.0 mL), H_2O (1.0 mL), Ph_2SiH_2 (0.4 mmol, 2.0 equiv.) was added successively via a syringe. The electrocatalysis was performed at room temperature with a constant current of 5 mA maintained for 12 h. After that, the electrodes were washed with EtOAc (5 mL × 3) in an ultrasonic bath. Then H_2O (10 mL) was added to the system, and the resulting mixture was extracted with EtOAc (10 mL × 3). The combined organic phase was washed with saturated solution of NaCl (10 mL × 3) and dried with anhydrous Na_2SO_4, filtered, and concentrated in vacuo. The crude product was purified by column chromatography to furnish the desired product.

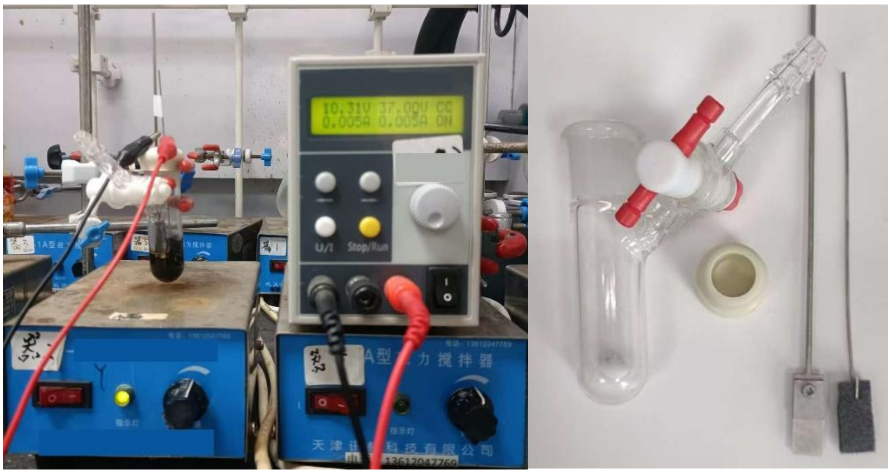

Supplementary Figure S3. Pictures of the reaction setups.

Supplementary Table S4: Faradaic efficiencies for the electrochemical reactions.

Compound	Faradaic efficiencies(%)	Compound	Faradaic efficiencies(%)	Compound	Faradaic efficiencies(%)
**1**	16.4%	**29**	8.4%	**58**	14.3%
**3**	13.0%	**30**	15.7%	**59**	11.6%
**6**	16.3%	**31**	11.4%	**60**	14.5%
**7**	11.6%	**32**	9.5%	**61**	10.9%
**8**	10.0%	**33**	9.3%	**62**	7.1%
**9**	15.0%	**34**	8.8%		
**10**	12.0%	**35**	8.9%		
**11**	11.8%	**36**	11.8%		
**12**	12.2%	**37**	11.1%		
**13**	12.7%	**38**	7.1%		
**14**	12.0%	**39**	13.0%		
**15**	14.5%	**40**	9.5%		
**16**	15.0%	**42**	16.4%		
**17**	9.8%	**43**	14.7%		
**18**	13.4%	**47**	10.5%		
**19**	10.7%	**48**	13.0%		
**20**	9.1%	**49**	9.3%		
**21**	12.9%	**50**	9.1%		
**22**	10.9%	**51**	16.3%		
**23**	12.2%	**52**	14.5%		
**24**	10.9%	**53**	11.6%		
**25**	10.9%	**54**	15.9%		
**26**	11.1%	**55**	11.6%		
**27**	15.4%	**56**	8.6%		
**28**	12.5%	**57**	11.3%		

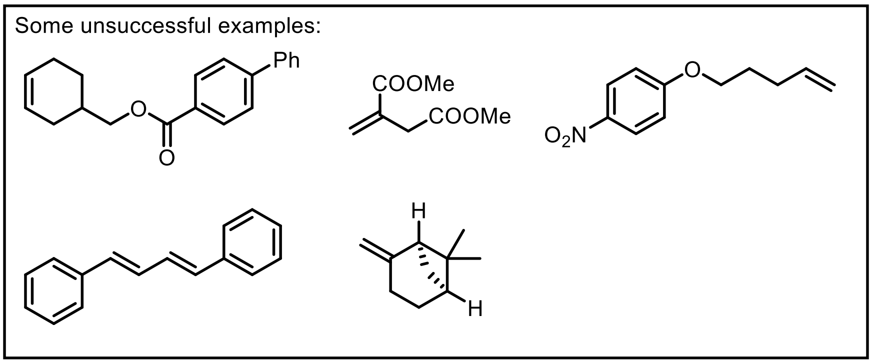

Supplementary Figure S4. Some unsuccessful examples.

Gram-Scale Reaction

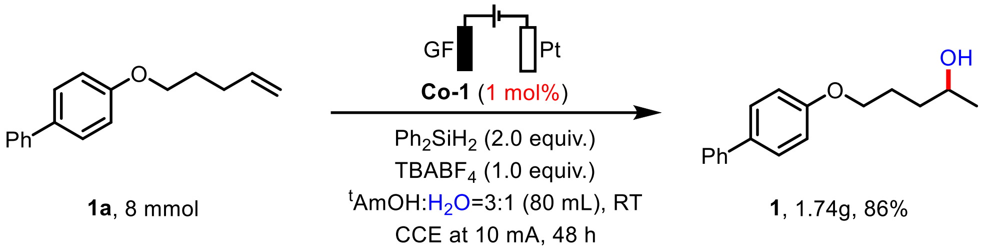

The electrocatalysis was carried out in an undivided cell with a graphite felt (GF) anode (25.0 mm × 50.0 mm × 5.0 mm) and a Pt plate cathode (25.0 mm × 50.0 mm × 0.25 mm). To an over-dried undivided electrochemical cell (diameter: 40 mm; length: 130 mm; volume: 200 mL) equipped with a magnetic bar were added substrates alkene **1a** (8 mmol, 1.0 equiv.), Co-1 (0.08 mmol, 1 mol%), $TBABF_4$ (8 mmol, 1.0 equiv.). Then the tube was evacuated and back-filled under Ar flow (this procedure was repeated three times), and 2-Methyl-2-butanol (60.0 mL), H_2O (20.0 mL), Ph_2SiH_2 (16 mmol, 2.0 equiv.) was added successively via a syringe. The electrocatalysis was performed at room temperature with a constant current of 10 mA maintained for 48 h. The electrodes were washed with EtOAc (50 mL × 3) in an ultrasonic bath. Then H_2O (100 mL) was added to the system, and the resulting mixture was extracted with EtOAc (100 mL × 3). The combined organic phase was washed with saturated solution of NaCl (100 mL × 3) and dried with anhydrous Na_2SO_4, filtered, and concentrated in vacuo. The crude product was purified by column chromatography to furnish the desired product **1** (1.74 g, 86%).

Mechanistic Studies

(a) O^{18} labeling experiment

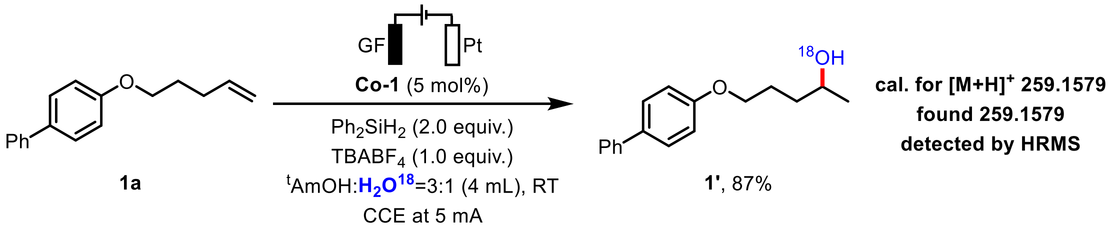

The reaction was carried out in an undivided cell with Graphite felt electrodes (10 mm × 20 mm × 5 mm) and Platinum electrodes (10 mm × 15 mm × 0.25 mm). To a 15.0

mL oven-dried undivided cell equipped with a magnetic bar was added substrates alkene **1a** (0.2 mmol, 1.0 equiv.), **Co-1** (0.01 mmol, 5 mol%), $TBABF_4$ (0.2 mmol, 1.0 equiv.). Then the tube was evacuated and back-filled under Ar flow (this procedure was repeated three times), and 2-Methyl-2-butanol (3.0 mL), H_2O^{18} (1.0 mL), Ph_2SiH_2 (0.4 mmol, 2.0 equiv.) was added successively via a syringe. The electrocatalysis was performed at room temperature with a constant current of 5 mA maintained for 12 h. After that, the electrodes were washed with EtOAc (5 mL × 3) in an ultrasonic bath. Then H_2O (10 mL) was added to the system, and the resulting mixture was extracted with EtOAc (10 mL × 3). The combined organic phase was washed with saturated solution of NaCl (10 mL × 3) and dried with anhydrous Na_2SO_4, filtered, and concentrated in vacuo. The crude product was purified by column chromatography to furnish the desired product **1'** in 87% yield.

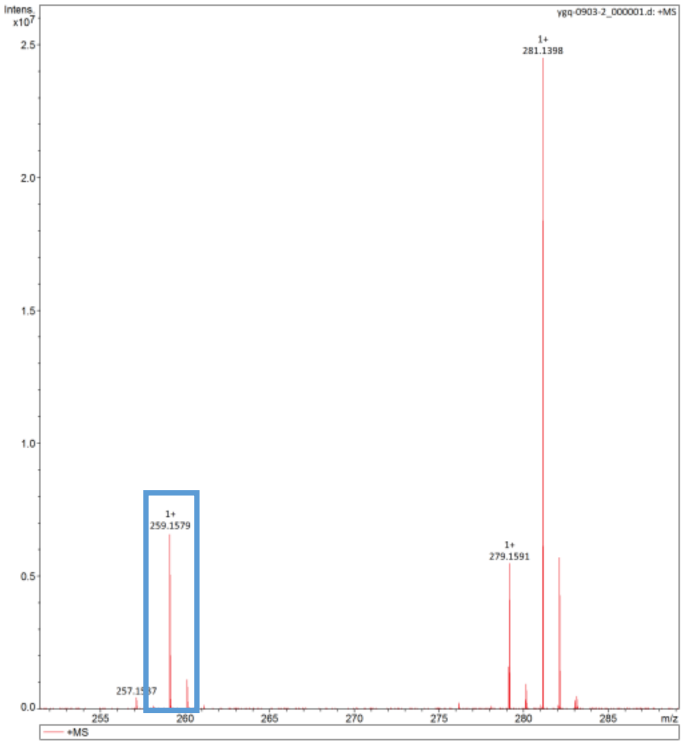

Supplementary Figure S5. The HRMS of **1'** (O^{18})

(b) Deuterium labeling experiment

Preparation of diphenylsilane-*d* (Ph_2SiD_2):

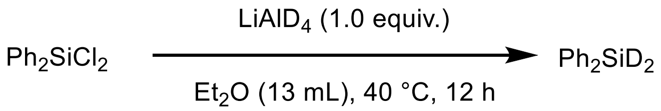

The title compound was prepared the following literature procedure[9]. To a stirring suspension of $LiAlD_4$ (9.5 mmol, 1.0 equiv.) in dry Et_2O (13 mL) was added Ph_2SiCl_2 (13 mmol, 1.36 equiv.) dropwise at ambient temperature under Ar. The reaction mixture was refluxed at 40 °C for 12 h. The reaction was cooled to room temperature. Then, the reaction was quenched by adding aqueous solution of sodium hydroxide (15 mL, 10 wt%) into the crude reaction mixture, which was subsequently extracted by diethyl ether for three times. The combined organic layers were dried over Na_2SO_4, evaporated under reduced pressure, and purified by column chromatography on silica gel to give Ph_2SiD_2 as a colorless oil in 70% yield (1.70 g, 99% D). **^{1}H NMR (400 MHz, Chloroform-d)** δ 7.63 – 7.56 (m, 4H), 7.42 – 7.30 (m, 6H).

^{1}H NMR spectrum of Ph_2SiD_2:

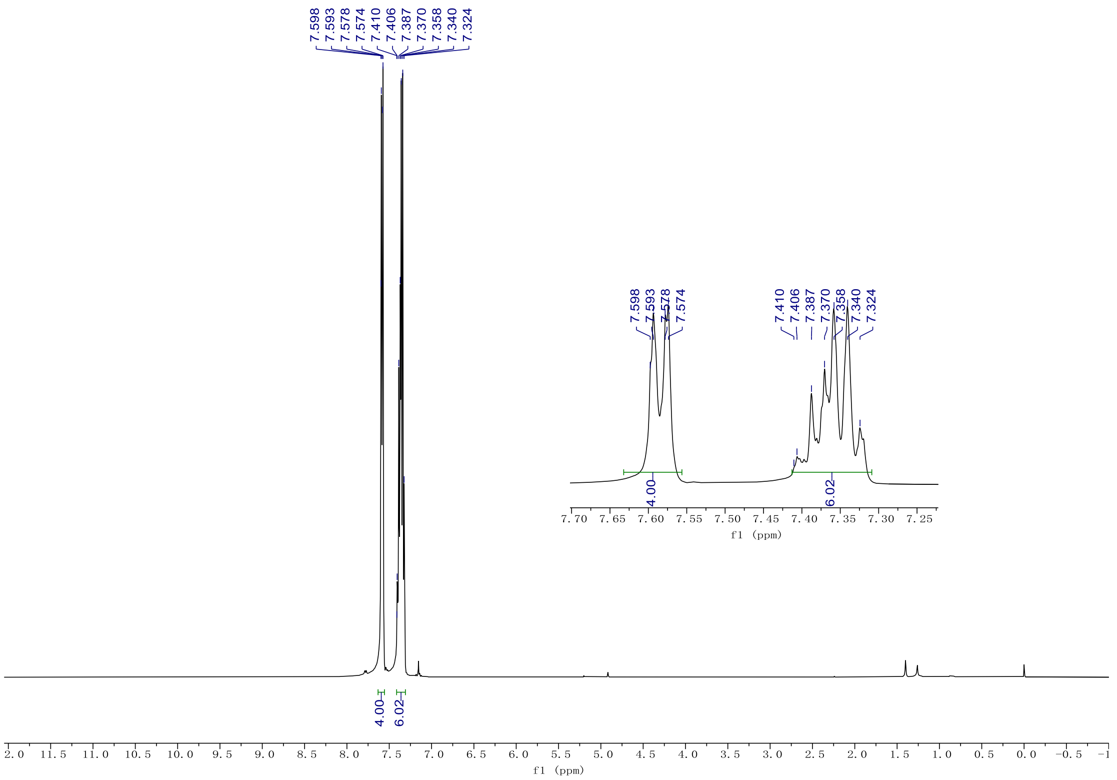

Deuterium labeling experiment using Ph_2SiD_2

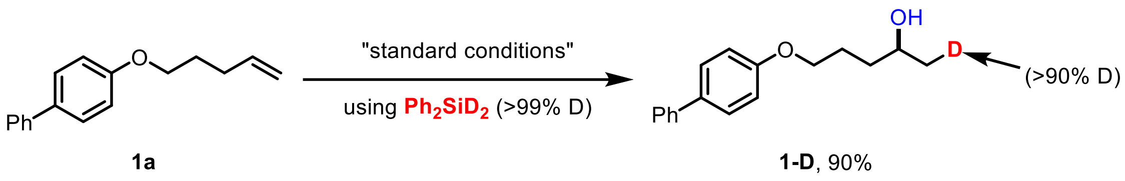

The reaction was carried out in an undivided cell with Graphite felt electrodes (10 mm × 20 mm × 5 mm) and Platinum electrodes (10 mm × 15 mm × 0.25 mm). To a 15.0 mL oven-dried undivided cell equipped with a magnetic bar was added substrates alkene **1a** (0.2 mmol, 1.0 equiv.), **Co-1** (0.01 mmol, 5 mol%), $TBABF_4$ (0.2 mmol, 1.0 equiv.). Then the tube was evacuated and back-filled under Ar flow (this procedure was repeated three times), and 2-Methyl-2-butanol (3.0 mL), H_2O (1.0 mL), Ph_2SiD_2 (0.4 mmol, 2.0 equiv.) was added successively via a syringe. The electrocatalysis was performed at room temperature with a constant current of 5 mA maintained for 12 h. After that, the electrodes were washed with EtOAc (5 mL × 3) in an ultrasonic bath. Then H_2O (10 mL) was added to the system, and the resulting mixture was extracted with EtOAc (10 mL × 3). The combined organic phase was washed with saturated solution of NaCl (10 mL × 3) and dried with anhydrous Na_2SO_4, filtered, and concentrated in vacuo. The crude product was purified by column chromatography to furnish the desired product **1-D** in 90% yield, with D incorporation (> 90% D), which demonstrated that the hydrogen came from silane.

^{1}H NMR spectrum of 1-D:

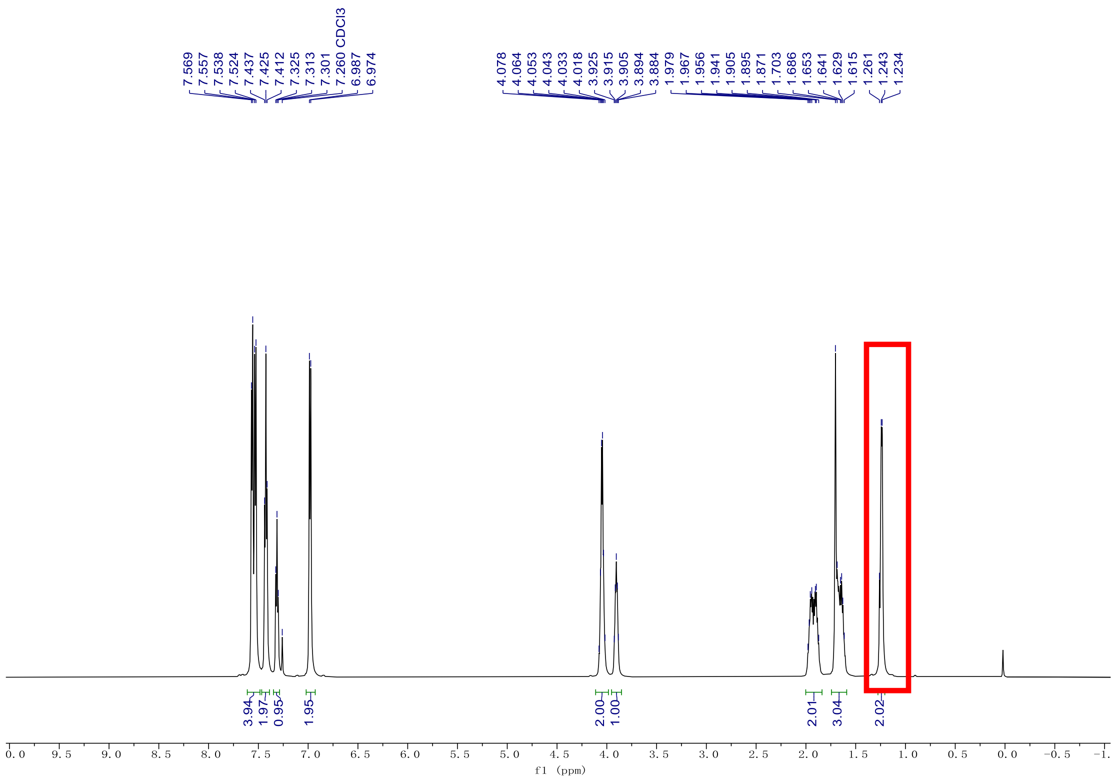

Deuterium labeling experiment using D_2O

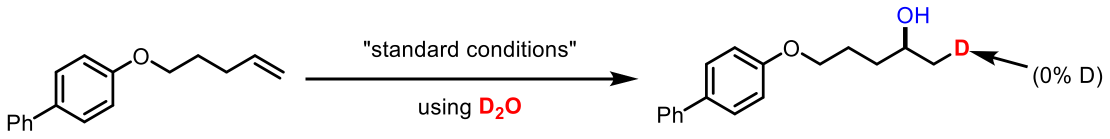

1a **1**, 89%

The reaction was carried out in an undivided cell with Graphite felt electrodes (10 mm × 20 mm × 5 mm) and Platinum electrodes (10 mm × 15 mm × 0.25 mm). To a 15.0 mL oven-dried undivided cell equipped with a magnetic bar was added substrates alkene **1a** (0.2 mmol, 1.0 equiv.), **Co-1** (0.01 mmol, 5 mol%), $TBABF_4$ (0.2 mmol, 1.0 equiv.). Then the tube was evacuated and back-filled under Ar flow (this procedure was repeated three times), and 2-Methyl-2-butanol (3.0 mL), D_2O (1.0 mL), Ph_2SiH_2 (0.4 mmol, 2.0 equiv.) was added successively via a syringe. The electrocatalysis was performed at room temperature with a constant current of 5 mA maintained for 12 h. After that, the electrodes were washed with EtOAc (5 mL × 3) in an ultrasonic bath. Then H_2O (10 mL) was added to the system, and the resulting mixture was extracted with EtOAc (10 mL × 3). The combined organic phase was washed with saturated solution of NaCl (10 mL × 3) and dried with anhydrous Na_2SO_4, filtered, and

concentrated in vacuo. The crude product was purified by column chromatography to furnish the desired product **1** in 89% yield, with D incorporation (0% D), which demonstrated that the hydrogen did not came from water. ^{1}H NMR of **1** (400 MHz, Chloroform-d) δ 7.61 – 7.52 (m, 4H), 7.45 (t, J = 7.6 Hz, 2H), 7.34 (t, J = 7.2 Hz, 1H), 7.04 – 6.97 (m, 2H), 4.07 (h, J = 6.2, 2.5 Hz, 2H), 3.93 (h, J = 5.6 Hz, 1H), 2.03 – 1.88 (m, 2H), 1.76 – 1.63 (m, 2H), 1.28 (d, J = 6.4 Hz, 3H).

(c) Radical inhibition experiment

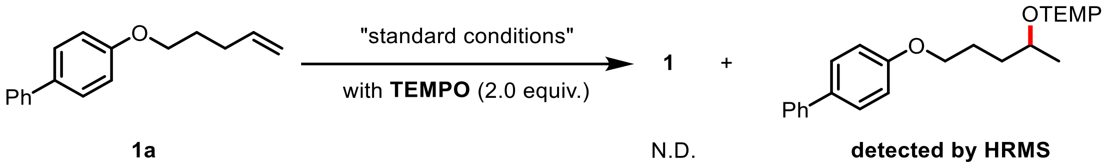

The reaction was carried out in an undivided cell with Graphite felt electrodes (10 mm × 20 mm × 5 mm) and Platinum electrodes (10 mm × 15 mm × 0.25 mm). To a 15.0 mL oven-dried undivided cell equipped with a magnetic bar was added substrates alkene **1a** (0.2 mmol, 1.0 equiv.), **Co-1** (0.01 mmol, 5 mol%), $TBABF_4$ (0.2 mmol, 1.0 equiv.), TEMPO (0.4 mmol, 2.0 equiv.). Then the tube was evacuated and back-filled under Ar flow (this procedure was repeated three times), and 2-Methyl-2-butanol (3.0 mL), H_2O (1.0 mL), Ph_2SiH_2 (0.4 mmol, 2.0 equiv.) was added successively via a syringe. The electrocatalysis was performed at room temperature with a constant current of 5 mA maintained for 12 h. After that, the electrodes were washed with EtOAc (5 mL × 3) in an ultrasonic bath. Then H_2O (10 mL) was added to the system, and the resulting mixture was extracted with EtOAc (10 mL × 3). The combined organic phase was washed with saturated solution of NaCl (10 mL × 3) and dried with anhydrous Na_2SO_4, filtered, and concentrated in vacuo. The result demonstrated that no target product was generated and the corresponding TEMPO-trapped product was detected by HRMS. HRMS (ESI, m/z): Calculated $C_{26}H_{37}NO_2$ $[M+H]^+$: 396.2897 , found 396.2886.

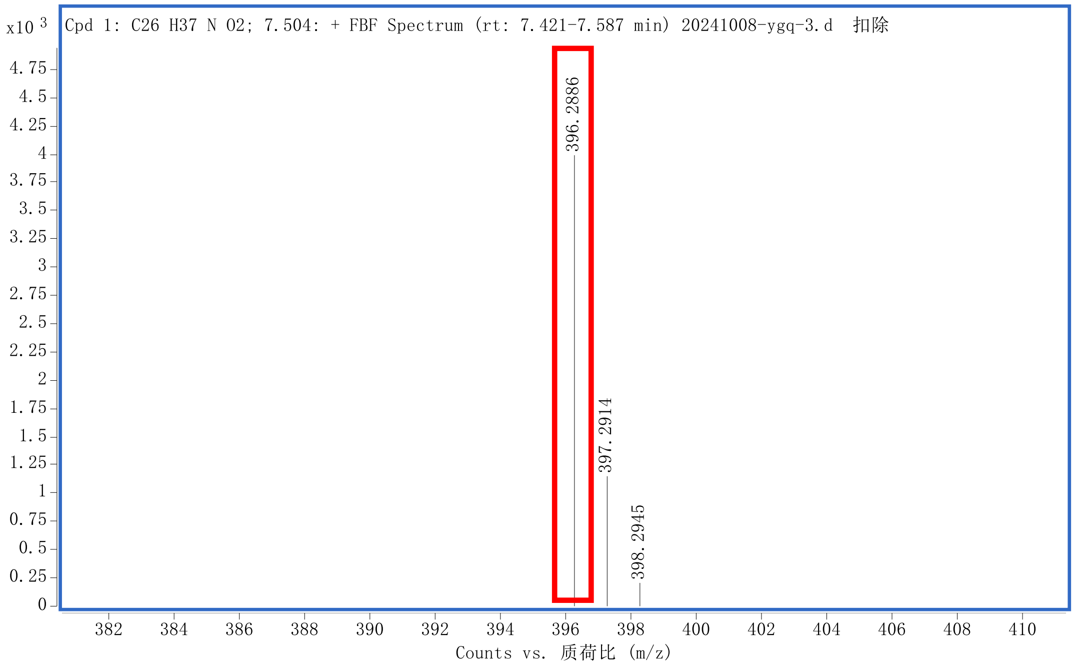

Supplementary Figure S6. The HRMS of TEMPO-trapped product

(d) Radical clock reaction

Preparation of ((1*R*,2*S*)-2-vinylcyclopropyl) benzene:

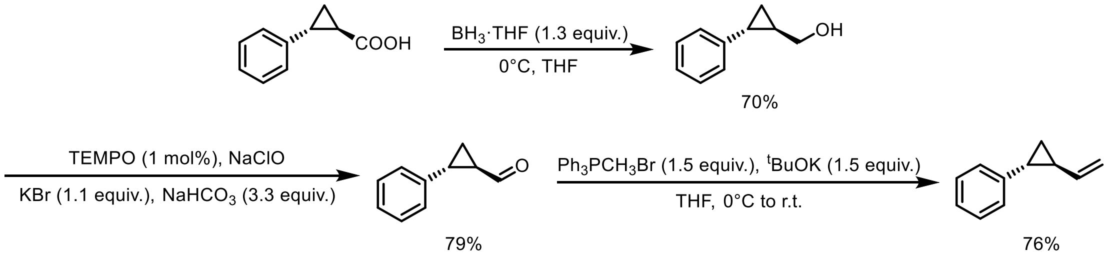

The title compound was prepared following a modified three-step literature procedure[10]. In a 100 mL dried Schlenk tube with a stir bar under argon trans-2-phenylcyclopropane 1- carboxylic acid (10.0 mmol, 1.0 equiv.) was dissolved in THF (20 mL). The solution was cooled to 0 °C and BH_3·THF (13.0 mL of a 1.0 M solution in THF, 13.0 mmol, 1.3 equiv.) was added dropwise. The reaction mixture was stirred for 1 h at 0 °C and then 1 h at rt. As TLC suggested that starting material remained, BH_3·THF (5.0 mL, 5.0 mmol, 0.5 equiv.) was added and the reaction mixture was stirred for another 1.5 h. The reaction mixture was then quenched by dropwise addition of MeOH at 0 °C and water was added until the formation of two layers could be observed. The layers were separated with Et_2O and brine and the aqueous layer

extracted with Et_2O (25 mL × 3). The combined organic layers were washed with brine (20 mL × 2), dried over $MgSO_4$, filtered and concentrated to afford trans-(3-phenylcyclopropyl) methanol (1.04 g, 7 mmol) as a colorless liquid.

The latter was transferred to a 100 mL round bottom flask with a stir bar and dissolved in CH_2Cl_2 (20 mL). After cooling to 0 °C, TEMPO (0.07 mmol, 1.0 mol%) and an aqueous solution of KBr (7.7 mmol, 1.1 equiv.) and $NaHCO_3$ (23.1 mmol, 3.3 eq.) in H_2O (40 mL) were added and the reaction mixture stirred. Next, sodium hypochlorite (10%, 10 mL) was added and, after stirring for 5 min, another 5 mL sodium hypochlorite solution was added. After the reaction mixture was stirred at 0 °C for 1 h, the reaction mixture was quenched with sat. aq. $Na_2S_2O_3$. The layers were separated, and the aqueous layer extracted with CH_2Cl_2 (25 mL × 3). The combined organic layers were dried over $MgSO_4$, filtered, concentrated and trans-2-phenylcyclopropanecarbaldehyde (5.5 mmol, 79%) was obtained.

A suspension of methyltriphenylphosphonium bromide (8.3 mmol, 1.5 equiv.) in 20 mL of THF under inert atmosphere was cooled at 0ºC with an ice bath. Then, potassium tert-butoxide (8.3 mmol, 1.5 equiv.) was added in two batches. The reaction was stirred for 10 minutes at 0 ºC and then the trans-2- phenylcyclopropanecarbaldehyde (5.5 mmol dissolved in 5 mL of THF) was added dropwise. The reaction was stirred for 12 hours at room temperature. After this time, it was quenched with a saturated aqueous NH_4Cl and extracted with Et_2O. The organic phases were collected, washed with brine and dried over $MgSO_4$. The solvent was removed under reduced pressure and the crude product was purified by flash column chromatography on silica gel, the title compound (602 mg, 4.2 mmol) was obtained as a colorless liquid. The analytical data is in accordance with the reported data[10].

Radical clock experiment

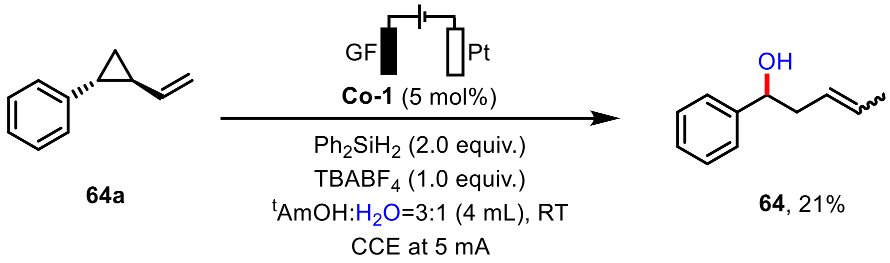

The reaction was carried out in an undivided cell with Graphite felt electrodes (10 mm × 20 mm × 5 mm) and Platinum electrodes (10 mm × 15 mm × 0.25 mm). To a 15.0 mL oven-dried undivided cell equipped with a magnetic bar was added substrates alkene **64a** (0.2 mmol, 1.0 equiv.), **Co-1** (0.01 mmol, 5 mol%), $TBABF_4$ (0.2 mmol, 1.0 equiv.). Then the tube was evacuated and back-filled under Ar flow (this procedure was repeated three times), and 2-Methyl-2-butanol (3.0 mL), H_2O (1.0 mL), Ph_2SiH_2 (0.4 mmol, 2.0 equiv.) was added successively via a syringe. The electrocatalysis was performed at room temperature with a constant current of 5 mA maintained for 12 h. After that, the electrodes were washed with EtOAc (5 mL × 3) in an ultrasonic bath. Then H_2O (10 mL) was added to the system, and the resulting mixture was extracted with EtOAc (10 mL × 3). The combined organic phase was washed with saturated solution of NaCl (10 mL × 3) and dried with anhydrous Na_2SO_4, filtered, and concentrated in vacuo. The crude product was purified by column chromatography to furnish the desired product in 21% yield. The analytical data is in accordance with the reported data[11]. ^1H NMR (400 MHz, Chloroform-d) δ 7.42 – 7.32 (m, 4H), 7.30 – 7.26 (m, 1H), 5.74 – 5.54 (m, 1H), 5.50 – 5.37 (m, 1H), 4.80 – 4.60 (m, 1H), 2.69 – 2.32 (m, 2H), 1.72 – 1.67 (m, 1H), 1.63 – 1.57 (m, 2H). ^{13}C NMR (100 MHz, Chloroform-d) δ 144.1, 144.1, 129.4, 128.4, 128.4, 127.6, 127.5, 127.4, 126.8, 125.9, 125.8, 125.7, 73.9, 73.5, 42.8, 36.9, 18.1, 13.0. HRMS (ESI, m/z): Calculated $C_{11}H_{14}O$ $[M+H]^+$: 163.1117 , found 163.1115.

^{1}H NMR spectrum of 64

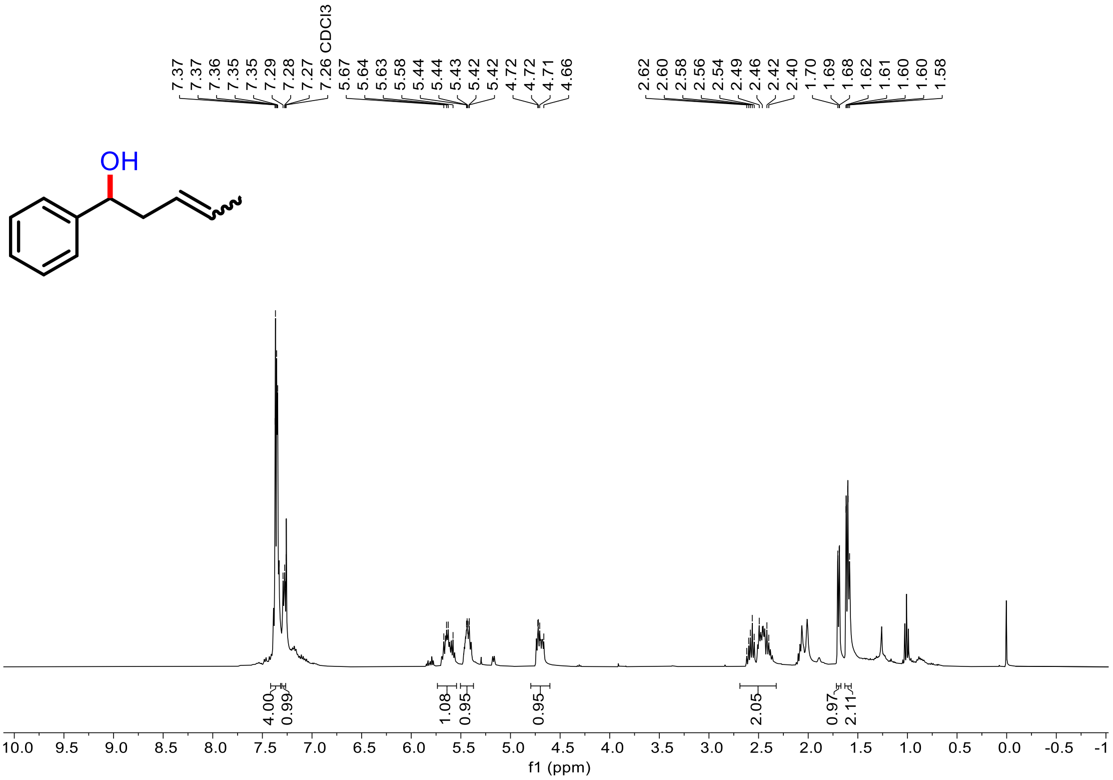

^{13}C NMR spectrum of 64

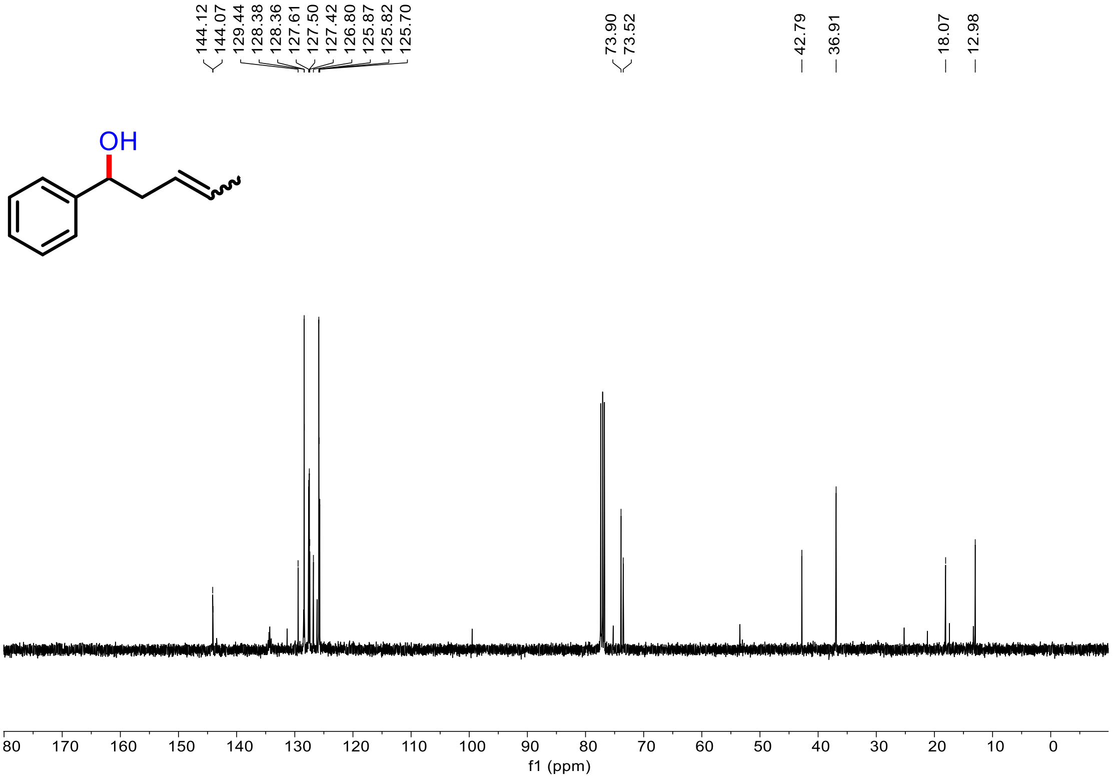

Kinetic isotope effect experiments

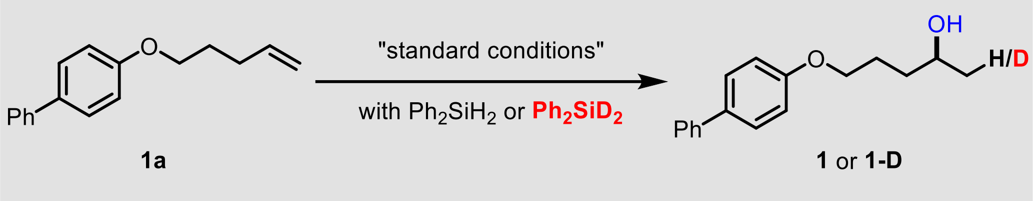

Ten parallel reactions using Ph_2SiH_2 or Ph_2SiD_2 were carried out to determine the kinetic isotope effect by comparison of the initial rates. The reaction was carried out in an undivided cell with Graphite felt electrodes (10 mm × 20 mm × 5 mm) and Platinum electrodes (10 mm × 15 mm × 0.25 mm). To a 15.0 mL oven-dried undivided cell equipped with a magnetic bar was added substrates alkene **1a** (0.2 mmol, 1.0 equiv.), Co-1 (0.01 mmol, 5 mol%), $TBABF_4$ (0.2 mmol, 1.0 equiv.). Then the tube was evacuated and back-filled under Ar flow (this procedure was repeated three times), and 2-Methyl-2-butanol (3.0 mL), H_2O (1.0 mL), Ph_2SiH_2 or Ph_2SiD_2 (0.4 mmol, 2.0 equiv.) was added successively via a syringe. The electrocatalysis was performed at room temperature with a constant current of 5 mA. After 5, 10, 15, 20 and 25 minutes, the crude mixture was analyzed by ^{1}H NMR using 1,1-dibromomethane as the internal standard. The slope of the concentration of product *vs*. time plot represented initial reaction rate. The slope ratio of two curves proves KIE of $k_H/k_D \approx 1.5$.

Time/min	5	10	15	20	25
Concentration of **1**(mol/L)	0.0028	0.0066	0.0109	0.0129	0.015
Concentration of **1-D** (mol/L)	0.0025	0.004	0.0055	0.0075	0.0095

Supplementary Table S5. The concentration of **1** and **1-D** at different times

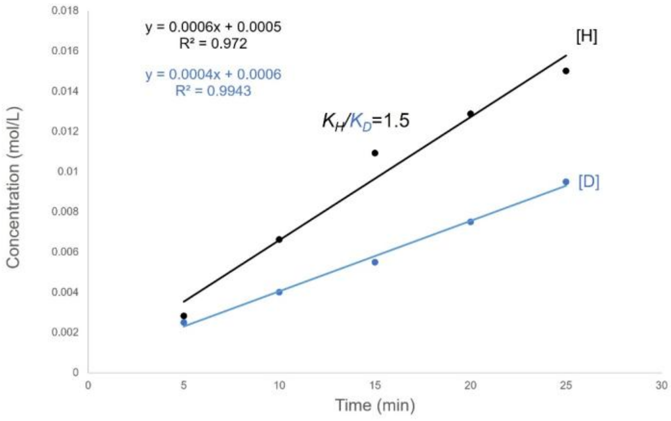

Supplementary Figure S7. Linear fit for the reaction rates of Ph_2SiH_2 or Ph_2SiD_2

The synthesis of an alkyl-Co(III) complex analog

The synthesis procedure was adapted from a literature procedure. [19-22] An oven-dried 3 mL vial equipped with a Teflon-coated magnetic stir bar was charged with (R,R)-(-)-*N*,*N'*-Bis(3,5-di-tert-Butylsalicylidene)-1,2-Cyclohexanediamno-Cobalt(II) (120.8 mg, 0.20 mmol) and Na (9.2 mg, 0.4 mmol), naphthalene (1.3 mg, 0.01 mmol). Anhydrous THF (2.0 mL) and pyridine (32 μL, 0.40 mmol) was added via syringe under Ar. The resulting red suspension was stirred at ambient temperature until a dark green solution was formed. This green solution was transferred to a separate 3 mL vial equipped with a stir bar under Ar, moved into a –30 °C refrigerator in a Ar glovebox. After 2 h, the vial was removed from the refrigerator and wrapped with aluminum foil to avoid light. 2-Bromopropane (20 μL, 0.21 mmol, 1.05 equiv.) was added. The resulting brown reaction mixture was allowed to warm to ambient temperature and stirred for another 30 minutes. 20 μL of this solution was taken up in C_6D_6 and transferred to a vacuum NMR tube for ^{1}H NMR spectroscopic analysis. The spectroscopic data was in agreement with previously reported data.

^{1}H NMR spectrum of alkyl-Co(III) complex analog

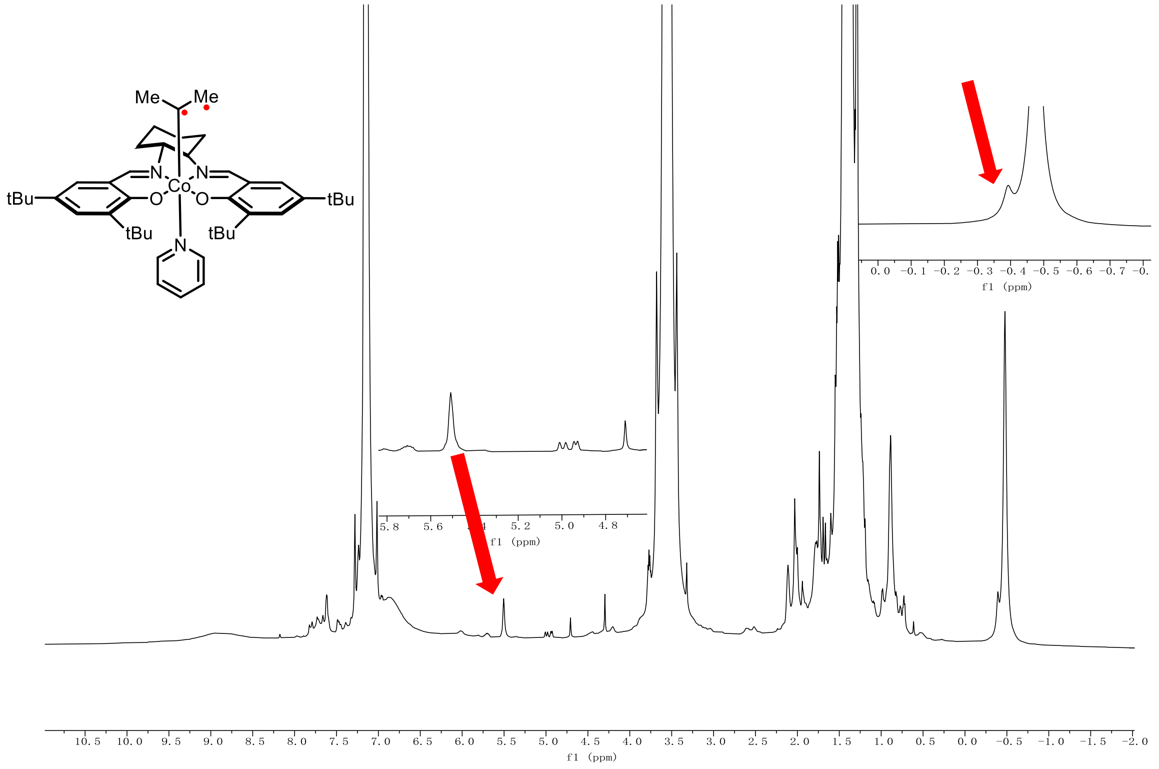

Cyclic Voltammetry Studies

The cyclic voltammetry was carried out with a Shanghai Chenhua CHI760E workstation. A glassy-carbon electrode (5 mm-diameter, disc-electrode) was used as the working electrode, a Pt plate was used as the auxiliary electrode and an Ag/AgCl electrode was used as a reference electrode. The sample should be bubbled with Ar for 5 min before testing. The measurements were carried out at a scan rate of 100 mV s^{-1} in THF:HFIP = 3:1/$^{n}Bu_4NPF_6$ (0.1 M).

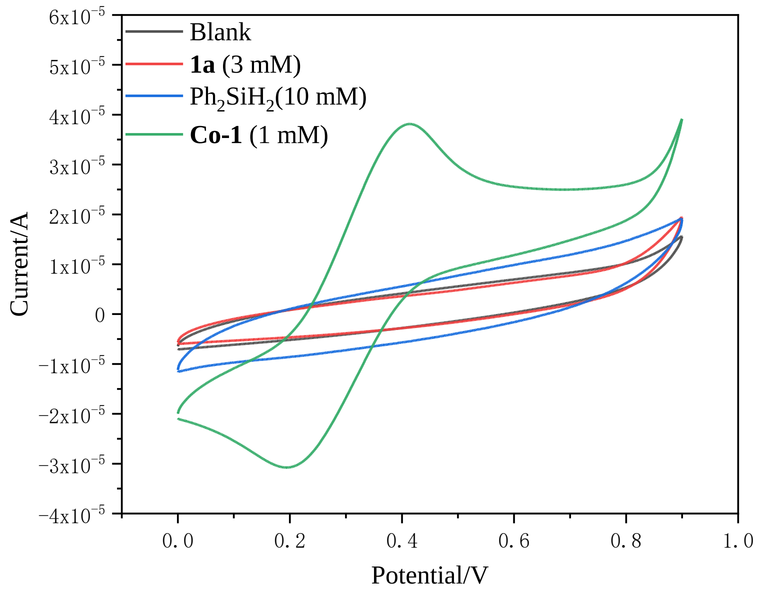

Supplementary Figure S8. Cyclic voltammograms of **1a** (3 mM), Ph_2SiH_2 (10 mM), **Co-1** (1 mM) in THF:HFIP = 3:1 with $^{n}Bu_4NPF_6$ (0.1 M).

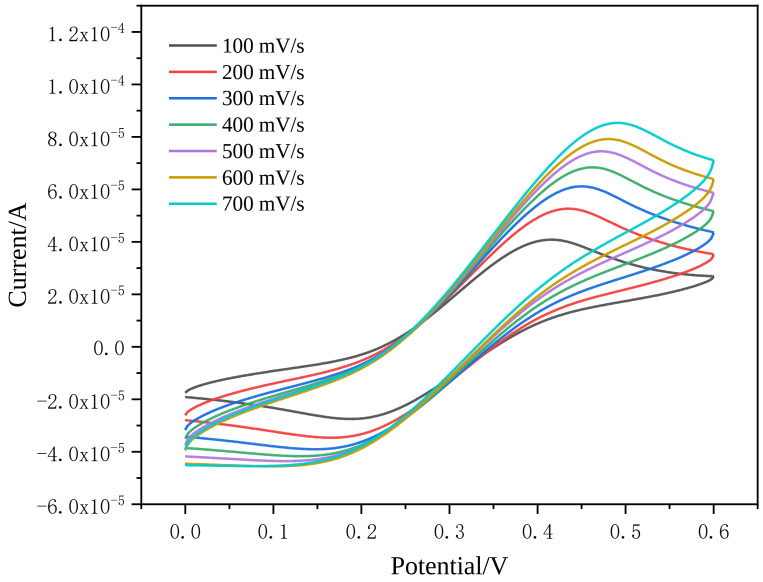

Supplementary Figure S9. Cyclic voltammograms of **Co-1** (1 mM) at different scanning speeds in THF:HFIP = 3:1 with $^{n}Bu_4NPF_6$ (0.1 M).

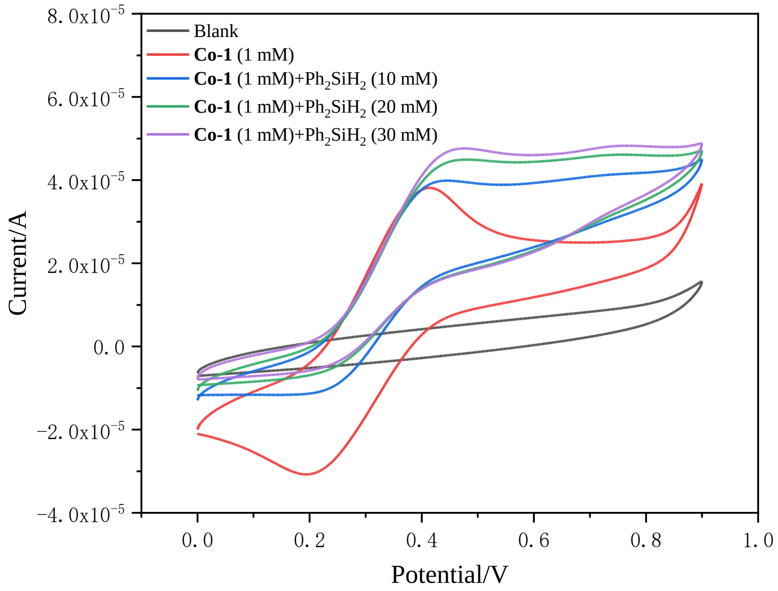

Supplementary Figure S10. Cyclic voltammograms of **Co-1** (1 mM) performed in the presence of increasing equivalents of Ph_2SiH_2 in THF:HFIP = 3:1 with $^{n}Bu_4NPF_6$ (0.1 M).

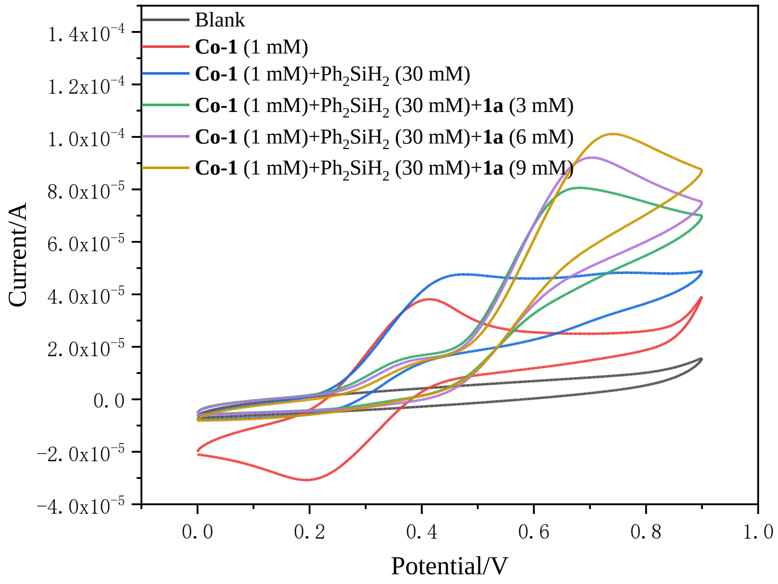

Supplementary Figure S11. Cyclic voltammograms of **Co-1** in the absence or presence of Ph_2SiH_2 and/or substrate **1a** in THF:HFIP = 3:1 with $^{n}Bu_4NPF_6$ (0.1 M).

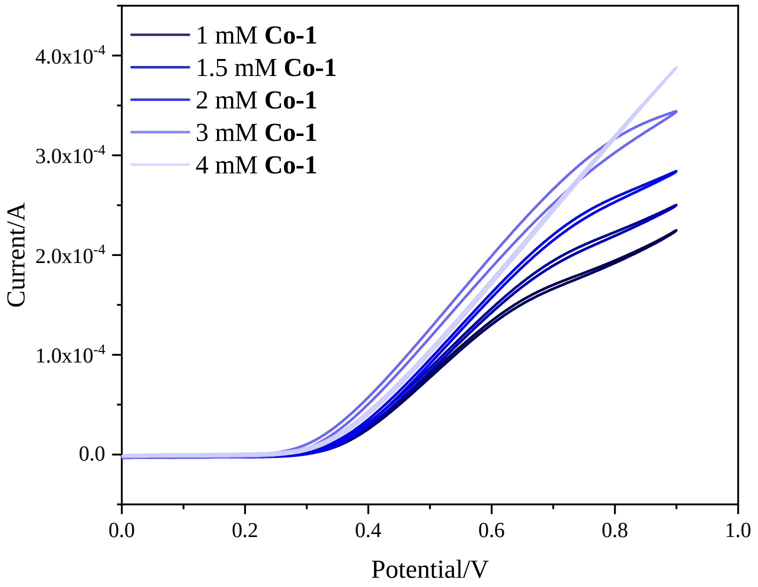

Supplementary Figure S12. Cyclic voltammograms of varying **Co-1** concentrations with 80 equiv. of Ph_2SiH_2 in THF:HFIP = 3:1 with $^{n}Bu_4NPF_6$ (0.1 M).

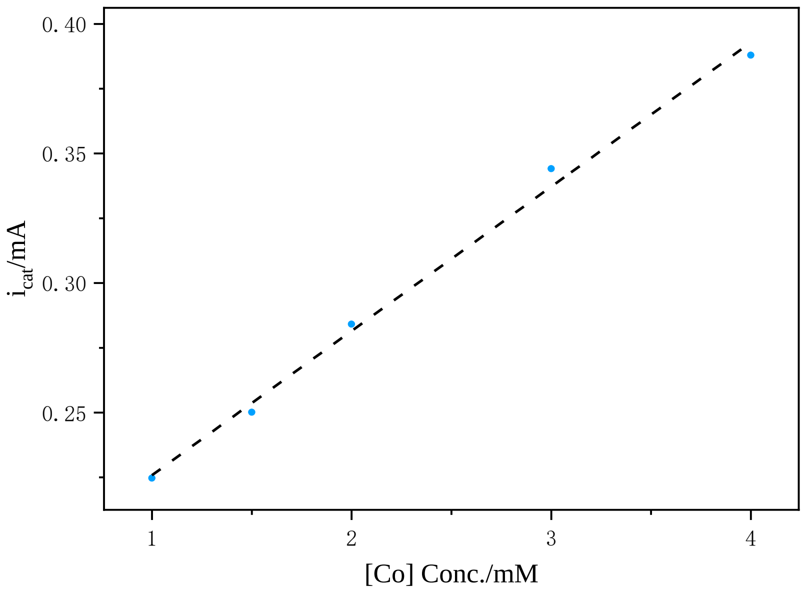

Supplementary Figure S13. Catalytic current analysis of the varying **Co-1** concentrations.

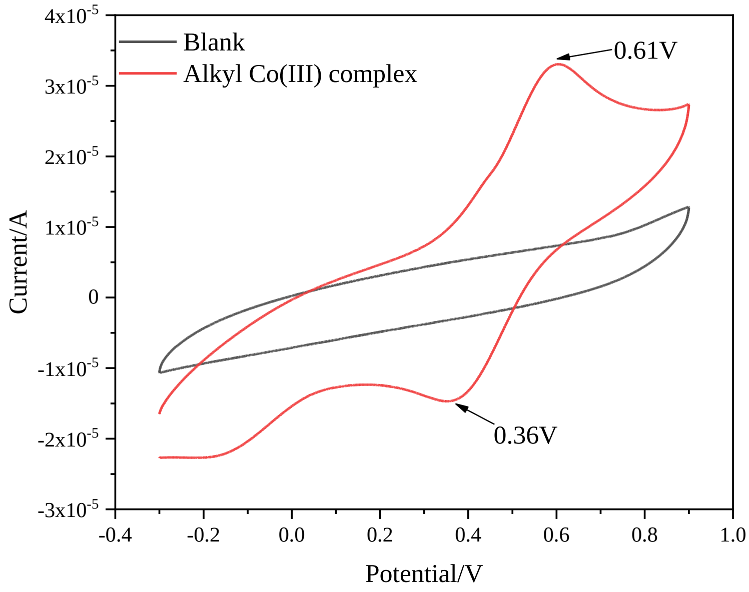

Supplementary Figure S14. Cyclic voltammograms of alkyl-Co(III) complex analog (2 mM) in CH_2Cl_2 with $^{n}Bu_4NPF_6$ (0.1 M).

DFT Calculations

General Information of DFT Computational Studies

For single-point energy (SPE) calculation of optimized geometries:

- Calculation software: Gaussian 16, Rev. A 03 [23]
- DFT functional: B3LYP-D3 (Hybrid functional B3LYP[24] with dispersion-correction DFT-D3[25])
- Basis sets: def2-TZVP[26]
- Solvation model: SMD[27], an implicit solvation model. Parameters of the SMD model were modified to find a closer fit to the actual solution environment (^{t}AmOH: H_2O = 3:1, *V*:*V*). Keyword "scrf=(solvent=generic)" was used. The input parameters are listed below:
  eps=61.69 # Static dielectric constant
  epsinf=1.917 # Square of the refractive index

For geometry optimization and frequency analysis:

- Calculation software: Gaussian 16, Rev. A 03[23]
- DFT functional: B3LYP-D3 (Hybrid functional B3LYP[24] with dispersion-correction DFT-D3[25])
- Basis sets: def2-SVP[28]
- Solvation model: IEFPCM[29], an implicit solvation model. Parameters of the IEFPCM model were modified to find a closer fit to the actual solution environment (^{t}AmOH: H_2O = 3:1, *V*:*V*). Keyword "scrf=(solvent=generic)" was used. The input parameters are listed below:
  eps=61.69 # Static dielectric constant
  epsinf=1.917 # Square of the refractive index

For the calculation of thermodynamic properties:

- Calculation software: Shermo 2.3[30], the calculated harmonic frequencies from frequency analysis are required for a certain geometry. Thermal corrections, including the thermal correction to Gibbs free energy (TCG), are the output.
- Environment parameters: T=298.15K and p=1 atm
- Treatment for low frequencies: Grimme's interpolation for entropy[31]
- Harmonic vibrational frequency scale factors for zero-point energy (ZPE), thermal energy (U), and entropy (S) were set to 0.977, 0.948, and 0.952, respectively. To see how these scale factors are obtained, please check the supporting information of Feng's work[32].
  In this work, we adopted the same method[32] to calculate the harmonic vibrational frequency scale factors. The procedure and test sets remained the same, however, while performing geometry optimization to molecules in test sets, B3LYP/def2-SVP was applied instead. To check test sets and source codes, please access:
  https://github.com/TMSCN/Computational_Chemistry_Utils/tree/main/Scale

_Factor_Generator

The level of DFT computation can be noted as SMD(^{t}AmOH/H_2O) / B3LYP-D3 / def2-TZVP // IEFPCM(^{t}AmOH/H_2O) / B3LYP -D3 / def2-SVP.

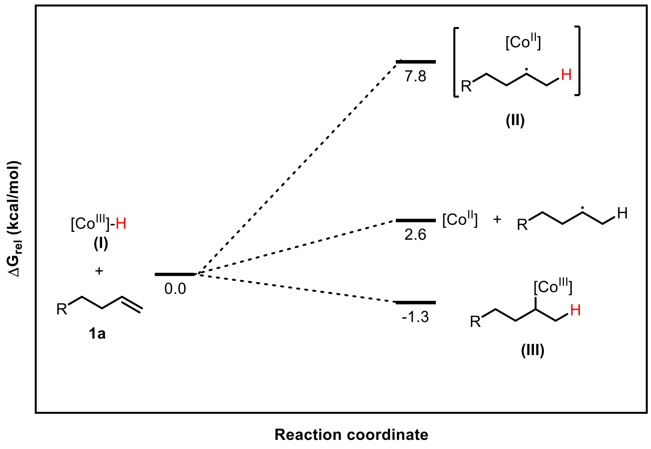

Supplementary Figure S15. Thermodynamic comparison of several possible intermediates following HAT

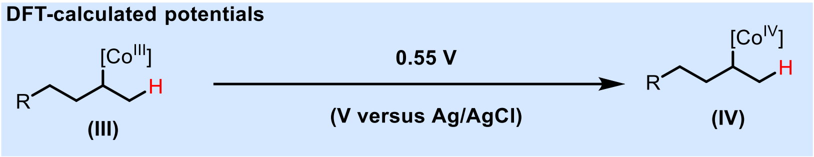

Supplementary Figure S16. DFT calculated potential

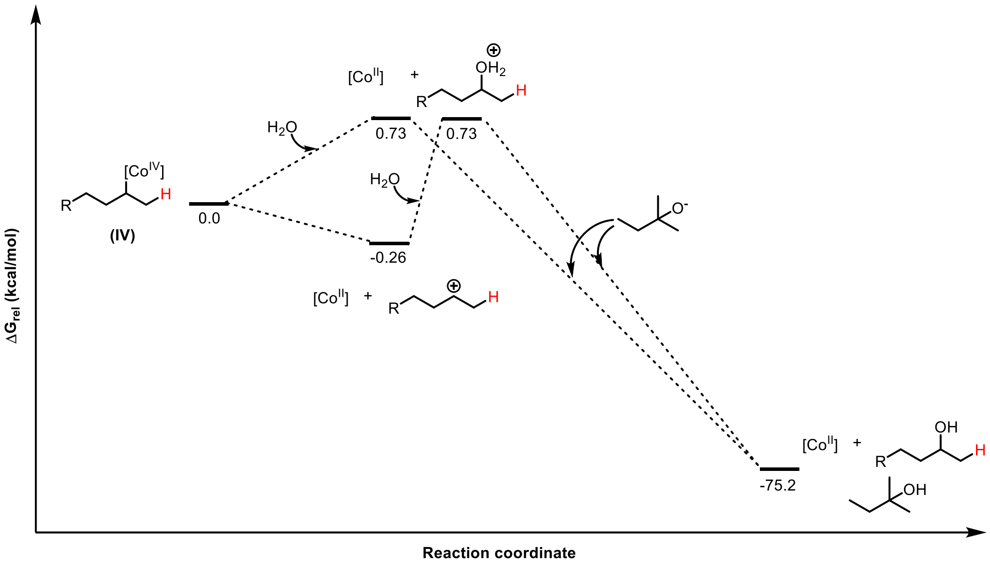

Supplementary Figure S17. Reaction paths from the alkylcobalt(IV) intermediate to product **1**

Computed Energies of Stationary Points

Supplementary Table S6. Single-point energies (SPE) and thermal corrections to

Gibbs free energies (TCG). 1 Hartree = 627.51 kcal/mol = 2625.5 kJ/mol.

Structures	SPE (Hartree)	TCG (Hartree)
**1**	-810.719	0.287175
**1a^{+}**	-734.633	0.269639
**1a.**	-734.796	0.267070
**[Co^{II}]**	-3043.32	0.679313
**H_2O**	-76.4717	0.003499
**INT-II**	-3778.14	0.975577
**INT-III**	-3778.16	0.982802
**INT-IV**	-3777.99	0.981353
**INT-V**	-811.131	0.300924
**^{t}AMO^{-}**	-272.602	0.117717
**^{t}AMOH**	-273.135	0.132381
**1a**	-734.227	0.260736
**[Co^{III}-H]**	-3043.89	0.686694

Cartesian Coordinates of Stationary Points (Unit: Å)

1

Charge = 0 Spin Multiplicity = 1

C -1.50210596 1.40345434 -0.33102874
C -0.12557827 1.58148979 -0.39775097
C 0.74511476 0.49989144 -0.17340866
C 0.20270996 -0.76162778 0.11788651
C -1.18394690 -0.92345808 0.18010396
C -2.06833206 0.14477421 -0.04048627
H -2.15368449 2.25639657 -0.53146635
H 0.30537842 2.55660365 -0.63350130
H 0.84650978 -1.61978418 0.30673149
H -1.58077455 -1.90943604 0.43068278
O 2.06960891 0.76503299 -0.25757472
C 3.00839914 -0.28556974 -0.05451033
H 2.83251676 -1.09365886 -0.78814692
H 2.87524547 -0.71875683 0.95389842
C 4.40326485 0.28827781 -0.21458063
H 4.49830912 0.73280740 -1.21697182
H 4.53694858 1.10700413 0.51322083
C 5.49109812 -0.76643511 -0.02145055
H 5.42760506 -1.19561649 0.99263405
H 5.33484553 -1.60051749 -0.72916276
C -3.53875993 -0.04168773 0.02924059
C -4.38029809 0.98763687 0.49390696

C	-4.13707514	-1.25360505	-0.36694471
C	-5.76422351	0.81286855	0.55909839
H	-3.94389264	1.93062542	0.82972545
C	-5.52046408	-1.43057553	-0.29856549
H	-3.51270142	-2.06122043	-0.75463623
C	-6.34235575	-0.39778254	0.16404233
H	-6.39409657	1.62546150	0.92955994
H	-5.95986663	-2.37883849	-0.61798938
H	-7.42492541	-0.53528134	0.21603802
C	7.97104285	-1.28294419	0.03075853
H	8.97925070	-0.86998929	-0.13622094
H	7.92418565	-1.64687553	1.06933900
H	7.83412627	-2.14060447	-0.64752843
C	6.90250788	-0.22569557	-0.23068781
H	7.05609117	0.61703504	0.47472596
O	6.97417140	0.25332877	-1.57121102
H	7.83995213	0.66518934	-1.69485873

1a⁺

Charge = 1 Spin Multiplicity = 1

C	-1.48597097	1.38888233	-0.32304583
C	-0.10614205	1.54984663	-0.36262962
C	0.74244657	0.45923591	-0.10879575
C	0.18224563	-0.79212503	0.18507560
C	-1.20745019	-0.93670248	0.21962657
C	-2.07288193	0.14012010	-0.03159844
H	-2.12269647	2.24723940	-0.54619489
H	0.34210143	2.51666584	-0.59967777
H	0.81108529	-1.65599419	0.39791808
H	-1.62155662	-1.91474962	0.47259151
O	2.07593841	0.70861685	-0.16809538
C	2.99115037	-0.34984520	0.04126204
H	2.81299826	-1.16460802	-0.68362420
H	2.87128959	-0.77177071	1.05619938
C	4.38486522	0.23137483	-0.13615697
H	4.46029247	0.68505004	-1.13635247
H	4.54467522	1.03171973	0.60228673
C	5.46703948	-0.83632447	-0.00413887
H	5.39530904	-1.32404656	1.02171942
H	5.38140621	-1.68685841	-0.69879217
C	-3.54688004	-0.02743336	0.00829132
C	-4.38413991	1.01660998	0.44660911

C	-4.15163343	-1.23494146	-0.39096134
C	-5.77127010	0.86020828	0.48327084
H	-3.94264480	1.95652570	0.78428864
C	-5.53840835	-1.39324841	-0.35152971
H	-3.52960617	-2.05378437	-0.75837156
C	-6.35626921	-0.34609046	0.08513631
H	-6.39834888	1.68373917	0.83366716
H	-5.98333550	-2.33818216	-0.67303002
H	-7.44141421	-0.46909187	0.11455642
C	6.82167863	-0.42444789	0.21607257
H	6.97993527	0.55111812	0.69978798
C	7.99343228	-1.19781774	-0.10435129
H	8.39966522	-0.62556768	-0.97762950
H	8.78284347	-1.10079329	0.65746179
H	7.79832259	-2.22837947	-0.42323968

1a.

Charge = 0   Spin Multiplicity = 2

C	-1.50117897	1.39450321	-0.32795640
C	-0.12260909	1.56290957	-0.37510798
C	0.73705518	0.47796563	-0.12638450
C	0.18200688	-0.77674907	0.16993933
C	-1.20648872	-0.92894912	0.21216364
C	-2.08019368	0.14256281	-0.03352260
H	-2.14382949	2.24957994	-0.54723725
H	0.31838370	2.53268043	-0.61429093
H	0.81704147	-1.63696328	0.37810811
H	-1.61361507	-1.90961136	0.46693541
O	2.06473241	0.73340440	-0.19354821
C	2.99262029	-0.32218421	0.02833270
H	2.81612711	-1.13715478	-0.69745705
H	2.85027118	-0.74288950	1.04073831
C	4.39375883	0.23907110	-0.12593619
H	4.49483537	0.67479175	-1.13393044
H	4.52534976	1.06666068	0.59161566
C	5.47699862	-0.81900716	0.09574317
H	5.33630649	-1.27297133	1.10326693
H	5.33100571	-1.66188591	-0.60817355
C	-3.55275251	-0.03371331	0.01503319
C	-4.39462387	1.00607172	0.45514000
C	-4.15277904	-1.24610491	-0.37709572
C	-5.78053671	0.84104833	0.50025355

H	-3.95728746	1.94981226	0.78757816
C	-5.53823132	-1.41327560	-0.32878574
H	-3.52781009	-2.06210952	-0.74583571
C	-6.36041845	-0.37012710	0.10943029
H	-6.41071773	1.66173800	0.85186186
H	-5.97885097	-2.36213993	-0.64472571
H	-7.44456836	-0.49999482	0.14574716
C	6.86822593	-0.29440896	-0.03287165
H	7.04046692	0.76743482	0.17753026
C	8.03624903	-1.21675986	-0.08830260
H	8.95791855	-0.70226806	-0.40235311
H	8.24803005	-1.67683747	0.90160784
H	7.85717987	-2.05997934	-0.77990421

[Co^{II}]

Charge = 0   Spin Multiplicity = 2

Co	0.00001156	0.86865573	-0.00039691
O	-1.28190838	-0.47872744	-0.10592344
N	-1.28569682	2.24925900	0.01525924
O	1.28190399	-0.47880048	0.10481702
N	1.28576782	2.24925670	-0.01566165
C	-3.27371328	0.83487346	0.04032627
C	2.57466458	-0.41455325	0.07689750
C	3.27378704	0.83484932	-0.03984403
C	3.36226879	-1.62206836	0.16764376
C	4.69677275	0.86920683	-0.10879532
C	4.74064354	-1.50791383	0.09162548
C	5.45016344	-0.28207020	-0.05461208
H	5.16896290	1.84749711	-0.20442000
H	5.33169013	-2.41984469	0.15119093
C	-2.57467505	-0.41448251	-0.07728533
C	-3.36233398	-1.62194420	-0.16810920
C	-4.74067201	-1.50780557	-0.09127506
C	-4.69664809	0.86922669	0.11013110
C	-5.45008872	-0.28202396	0.05589716
H	-5.33175808	-2.41970730	-0.15090163
H	-5.16877245	1.84748416	0.20641381
C	-2.58668614	2.07664031	0.06989644
H	-3.22451818	2.96479576	0.13714380
C	2.58679860	2.07661834	-0.06949244
H	3.22468541	2.96478220	-0.13610284
C	0.70626678	3.53983306	-0.00763122

C	1.40264308	4.75727765	-0.00621001
C	0.70013545	5.96190398	-0.00256558
C	-0.70008615	5.96190266	0.00075479
C	-1.40258434	4.75727279	0.00486045
C	-0.70619712	3.53983621	0.00682541
H	1.24932122	6.90561112	-0.00255837
H	2.49205425	4.77851576	-0.00569064
H	-1.24927643	6.90560662	0.00030067
H	-2.49199671	4.77849787	0.00417603
C	2.67548578	-2.98362669	0.36368380
C	1.71201830	-3.26429004	-0.80909601
H	0.93291547	-2.49808511	-0.86998893
H	2.26394400	-3.28889578	-1.76300522
H	1.22574960	-4.24381422	-0.67343786
C	3.68863001	-4.13859558	0.42393259
H	4.39546585	-4.02856940	1.26100136
H	3.14800639	-5.08652805	0.56935762
H	4.26965533	-4.22723918	-0.50736227
C	1.89995253	-2.96837122	1.69963587
H	2.59355667	-2.82660411	2.54416486
H	1.15807381	-2.16147335	1.71503240
H	1.37474514	-3.92588458	1.84633388
C	-2.67569098	-2.98345208	-0.36500133
C	-1.90061396	-2.96773632	-1.70120897
H	-1.15863451	-2.16093038	-1.71651642
H	-1.37558771	-3.92526195	-1.84848034
H	-2.59448020	-2.82552887	-2.54545041
C	-1.71183158	-3.26461362	0.80733602
H	-0.93271102	-2.49843287	0.86826117
H	-2.26342990	-3.28960672	1.76142288
H	-1.22559855	-4.24407733	0.67111152
C	-3.68892705	-4.13833356	-0.42535271
H	-4.26954051	-4.22739713	0.50615790
H	-4.39613379	-4.02785659	-1.26205091
H	-3.14842167	-5.08622645	-0.57147283
C	6.98233558	-0.30379693	-0.13373579
C	7.41893755	-1.14625191	-1.35118041
H	8.51825862	-1.18028640	-1.42130980
H	7.05652517	-2.18309960	-1.28157673
H	7.02861218	-0.71336671	-2.28579366
C	7.56760572	1.10793982	-0.28618356
H	7.21049342	1.59969186	-1.20424052

H	7.30951031	1.75059873	0.56970318
H	8.66563899	1.05218382	-0.34250730
C	7.55444699	-0.93166880	1.15506389
H	7.26039351	-0.34442210	2.03931597
H	7.19884919	-1.96297967	1.30043997
H	8.65532320	-0.96085852	1.11279713
C	-6.98221630	-0.30375854	0.13589161
C	-7.41812908	-1.14669211	1.35325211
H	-8.51740993	-1.18073723	1.42400233
H	-7.05577326	-2.18351709	1.28302187
H	-7.02725548	-0.71418767	2.28781252
C	-7.55508158	-0.93111283	-1.15282460
H	-7.19958797	-1.96237389	-1.29880896
H	-8.65593382	-0.96029751	-1.10993252
H	-7.26152843	-0.34352568	-2.03701678
C	-7.56737796	1.10792571	0.28923785
H	-7.20975347	1.59929458	1.20730048
H	-7.30974003	1.75093046	-0.56652697
H	-8.66538122	1.05216638	0.34614344

H_2O

Charge = 0 Spin Multiplicity = 1

O	-4.57467635	-0.39694893	1.98016761
H	-3.60859677	-0.33840017	1.98016761
H	-4.84196985	0.53326174	1.98016761

INT-II

Charge = 0 Spin Multiplicity = 3

Co	-0.25593172	-0.17075761	-0.80534647
O	-1.92838253	0.63581856	-0.46238891
N	-0.98288749	-1.92248861	-0.95210544
O	0.50432405	1.53490980	-0.55977223
N	1.37811706	-0.96379272	-1.35801721
C	-3.31779412	-1.30956298	-0.57965369
C	1.71892476	1.92457031	-0.74612071
C	2.72977089	1.06471446	-1.29929571
C	2.10342796	3.27189616	-0.38789751
C	4.05218629	1.54583244	-1.51301412
C	3.41613544	3.65765752	-0.60414859
C	4.42541642	2.82569905	-1.16731733
H	4.77134488	0.85084863	-1.94631806
H	3.70414357	4.66999082	-0.32606359

C	-3.10679865	0.11073486	-0.47707911
C	-4.27403629	0.96129948	-0.40629888
C	-5.52085772	0.35912175	-0.37282030
C	-4.63511499	-1.84998119	-0.53153834
C	-5.74734507	-1.04525637	-0.41882808
H	-6.39718244	1.00186563	-0.31429786
H	-4.73224020	-2.93405353	-0.59911567
C	-2.24967734	-2.22445680	-0.80183198
H	-2.54640451	-3.27625076	-0.88918747
C	2.49577688	-0.31264832	-1.56151243
H	3.36127710	-0.87284536	-1.93134421
C	1.28510798	-2.36157725	-1.50874272
C	2.34526467	-3.22578432	-1.82042152
C	2.13359112	-4.60204885	-1.86424318
C	0.86356727	-5.12993475	-1.59606204
C	-0.20053543	-4.28099538	-1.29297074
C	-0.00216483	-2.89280977	-1.25314608
H	2.96933413	-5.26729084	-2.08990974
H	3.34746087	-2.83747550	-1.99511640
H	0.70211942	-6.20968129	-1.61748884
H	-1.17917995	-4.71004066	-1.07791656
C	1.06591883	4.23299009	0.21831049
C	0.52649404	3.65601827	1.54571629
H	0.10086406	2.65637599	1.40542028
H	1.33444616	3.59264933	2.29281836
H	-0.25575575	4.31709837	1.95349768
C	1.66868259	5.61496988	0.51964064
H	2.04477512	6.10956651	-0.38962066
H	0.89120963	6.26307303	0.95289263
H	2.49457026	5.55748495	1.24577188
C	-0.09836832	4.43433825	-0.77584770
H	0.27229051	4.85357061	-1.72563748
H	-0.60701573	3.48671144	-0.98002298
H	-0.83488026	5.14010388	-0.35823948
C	-4.11747711	2.49197039	-0.42861112
C	-3.28009876	2.97384447	0.77481305
H	-2.28666360	2.51302118	0.77732071
H	-3.15593717	4.06805853	0.73315504
H	-3.79184301	2.72956280	1.71926872
C	-3.42968400	2.90403262	-1.74973302
H	-2.44455873	2.43249770	-1.84372185
H	-4.04778004	2.60848193	-2.61289586

H	-3.29642635	3.99741569	-1.78422335
C	-5.47569984	3.21016688	-0.36566179
H	-6.12129185	2.95465718	-1.22020183
H	-6.02310528	2.97597113	0.56080339
H	-5.31151125	4.29855245	-0.38808456
C	5.84909691	3.37085787	-1.33813328
C	6.40846088	3.77328928	0.04321975
H	7.43080865	4.17310611	-0.05557956
H	5.79155697	4.54778698	0.52359570
H	6.44383044	2.90271852	0.71736936
C	6.79163986	2.32554423	-1.95363531
H	6.87449442	1.42888800	-1.31988275
H	6.45330078	2.00890134	-2.95228416
H	7.80130948	2.75078455	-2.06161640
C	5.82239823	4.60766401	-2.26062934
H	5.43500458	4.34294644	-3.25720147
H	5.18593249	5.40721353	-1.85205877
H	6.83741341	5.01885932	-2.38485034
C	-7.18669248	-1.57555991	-0.37514808
C	-7.96062970	-1.04752040	-1.60217717
H	-9.00114360	-1.41083145	-1.58785452
H	-7.99072934	0.05254149	-1.62189318
H	-7.49003764	-1.38984365	-2.53740582
C	-7.88085279	-1.08649460	0.91367272
H	-7.91032447	0.01205682	0.96999205
H	-8.92047398	-1.45025698	0.95100332
H	-7.35522260	-1.45633712	1.80622610
C	-7.23251473	-3.11051235	-0.39766043
H	-6.78508600	-3.51889482	-1.31686632
H	-6.70275823	-3.54574440	0.46353009
H	-8.27776290	-3.45341144	-0.35496656
C	-4.33794312	-1.08077492	2.96832291
H	-4.52782918	-0.55184130	2.03037619
C	-5.00487907	-2.39075028	3.19965465
H	-4.39263837	-3.05422502	3.83664175
H	-5.97994954	-2.28285055	3.72355206
H	-5.21308636	-2.91209608	2.25292986
C	-3.63499844	-0.34143220	4.05847117
H	-3.35020358	-1.04426127	4.86292697
H	-4.32267935	0.38856972	4.53831934
C	-2.39050387	0.42459320	3.58665449
H	-1.92177240	0.94101456	4.44027654

H	-2.67152582	1.19708282	2.85504893
C	-1.36603243	-0.50272427	2.96028560
H	-1.72089867	-0.88222248	1.98742107
H	-1.18574981	-1.37294068	3.61767868
O	-0.15099794	0.21751521	2.78124318
C	0.93070919	-0.44372390	2.30817915
C	2.12015912	0.29473628	2.18456872
C	0.92921021	-1.79980356	1.94597557
C	3.27091386	-0.30725561	1.69829122
H	2.11311389	1.34792524	2.46354984
C	2.10016335	-2.39209494	1.47101777
H	0.02281338	-2.39872330	2.00956255
C	3.29185677	-1.66632952	1.31958059
H	4.17392536	0.29781558	1.60685456
H	2.05623813	-3.43613275	1.16129278
C	4.49613826	-2.28398387	0.71449930
C	5.45164419	-1.49655312	0.04202230
C	4.70347348	-3.67687559	0.74508906
C	6.55554609	-2.07540862	-0.58685481
H	5.31760398	-0.41639754	-0.01536076
C	5.80614660	-4.25770605	0.11715080
H	3.99556234	-4.31799388	1.27253896
C	6.73796980	-3.46147573	-0.55741838
H	7.27198975	-1.43748806	-1.11054643
H	5.93977271	-5.34155074	0.15737709
H	7.59861823	-3.91690824	-1.05262967

INT-III

Charge = 0 Spin Multiplicity = 1

Co	1.40128069	-0.44204612	0.07810763
O	3.07864700	0.41393123	-0.02418735
N	2.14813432	-2.13598567	0.49472597
O	0.65881835	1.29058365	0.24072499
N	-0.26067379	-1.28117053	0.49243787
C	4.47693493	-1.42946324	0.59343050
C	-0.55012765	1.66694983	0.49944133
C	-1.60973952	0.73027307	0.75919241
C	-0.87743328	3.07391897	0.54770166
C	-2.92816982	1.17720320	1.05641715
C	-2.18592891	3.43129374	0.83116745
C	-3.24261776	2.51666969	1.10152744
H	-3.68406394	0.41406733	1.24198264

H	-2.42852127	4.49183764	0.86189739
C	4.25945653	-0.07834180	0.14521665
C	5.42701915	0.73950215	-0.10184892
C	6.67361395	0.19792750	0.16507335
C	5.79300220	-1.90715044	0.85530254
C	6.90374849	-1.11797820	0.65632668
H	7.54810897	0.82048230	-0.01449006
H	5.88942527	-2.93430148	1.20857540
C	3.41097336	-2.35349642	0.76315650
H	3.69461736	-3.34879015	1.12706603
C	-1.38566577	-0.67064925	0.77278669
H	-2.24733130	-1.28437873	1.05932302
C	-0.15233447	-2.68512061	0.53219116
C	-1.21665150	-3.59819398	0.53910723
C	-0.95325202	-4.96788613	0.56539552
C	0.36820253	-5.43468472	0.57332715
C	1.43416010	-4.53504793	0.55380332
C	1.18043726	-3.15585226	0.54245478
H	-1.78432545	-5.67598737	0.56181499
H	-2.24889808	-3.24920621	0.50829565
H	0.56924558	-6.50790409	0.57456702
H	2.45745351	-4.91043219	0.52546956
C	0.21968791	4.13341449	0.35009068
C	0.95670437	3.92694397	-0.99159659
H	1.44602982	2.94870444	-1.02868518
H	0.25156660	4.00165793	-1.83510113
H	1.72270872	4.70862860	-1.12054195
C	-0.35081587	5.56156916	0.34845884
H	-0.83954600	5.81766012	1.30100785
H	0.47024688	6.27905390	0.19686599
H	-1.07987131	5.71154969	-0.46342046
C	1.21254987	4.02190805	1.52801716
H	0.70250892	4.24175214	2.47958890
H	1.64090793	3.01420766	1.58441600
H	2.03713226	4.74251727	1.40666865
C	5.27455854	2.17021172	-0.64611900
C	4.53020498	2.13457363	-1.99828577
H	3.53615110	1.68952451	-1.88308166
H	4.41199358	3.15669271	-2.39333520
H	5.09578952	1.54651048	-2.73905032
C	4.47997654	3.02647685	0.36054438
H	3.48229154	2.60761971	0.52187617

H	5.00587129	3.07798543	1.32788587
H	4.36688978	4.05492764	-0.01963043
C	6.63447390	2.84911691	-0.87740383
H	7.21060650	2.95136546	0.05536316
H	7.25301624	2.30122176	-1.60540187
H	6.46948265	3.86165873	-1.27677573
C	-4.64001411	3.05290703	1.43978253
C	-5.19532493	3.83674171	0.23231390
H	-6.19091419	4.24862891	0.46445539
H	-4.54006721	4.67769880	-0.04193424
H	-5.29149073	3.18075664	-0.64529370
C	-5.61692472	1.91721057	1.77893854
H	-5.73137750	1.21776820	0.93975679
H	-5.28330684	1.34534832	2.65882829
H	-6.61020817	2.33377166	2.00749752
C	-4.54756599	3.99314013	2.66037558
H	-4.14676521	3.45850832	3.53610995
H	-3.89664020	4.85870985	2.46505113
H	-5.54589681	4.37968826	2.92190667
C	8.34156943	-1.58464851	0.91835271
C	8.99696523	-0.65965043	1.96586542
H	10.03449701	-0.97505039	2.16247335
H	9.02492756	0.38681866	1.62627667
H	8.44230447	-0.69192239	2.91695408
C	9.14896074	-1.52266048	-0.39571654
H	9.18165161	-0.50259204	-0.80749996
H	10.18817112	-1.84751760	-0.22499106
H	8.70430491	-2.18126858	-1.15844794
C	8.38769342	-3.02550937	1.44803624
H	7.84132203	-3.12733547	2.39848413
H	7.95792884	-3.73841587	0.72747808
H	9.43205089	-3.32300893	1.62919938
C	1.30873797	-0.85101235	-1.86811377
H	0.52212862	-1.61979676	-1.90415888
C	2.60593570	-1.46241772	-2.38461660
H	2.47786598	-1.73832899	-3.44780823
H	3.44342674	-0.75196958	-2.33883021
H	2.89772927	-2.37398853	-1.85057939
C	0.93519213	0.34798208	-2.74278579
H	1.13718437	0.02859165	-3.78469412
H	1.63799697	1.17433143	-2.55711657
C	-0.49309816	0.89668177	-2.70369986

H	-0.65111954	1.54214653	-3.58363503
H	-0.64728621	1.53349826	-1.82680619
C	-1.56124701	-0.17833911	-2.68619731
H	-1.44507971	-0.79598965	-1.78886432
H	-1.49502725	-0.84776808	-3.56392304
O	-2.83502018	0.46235610	-2.66852513
C	-3.89317861	-0.15253984	-2.09530048
C	-5.07875255	0.59798773	-2.01623465
C	-3.88229108	-1.45829507	-1.57994068
C	-6.20490117	0.07631448	-1.39333593
H	-5.08550657	1.60155639	-2.44484691
C	-5.01971474	-1.96501250	-0.94596084
H	-2.99462405	-2.08457875	-1.65494261
C	-6.20010313	-1.21387307	-0.82438390
H	-7.11175152	0.68260490	-1.35205896
H	-4.97185279	-2.96926250	-0.51949529
C	-7.38994569	-1.74873124	-0.11865474
C	-8.24360228	-0.89526613	0.60735439
C	-7.69718228	-3.12286608	-0.14444874
C	-9.35977571	-1.39600394	1.28068700
H	-8.01766544	0.17103473	0.66614824
C	-8.81149610	-3.62449976	0.53107055
H	-7.06714675	-3.80544535	-0.71851296
C	-9.64972004	-2.76373459	1.24724904
H	-10.00195396	-0.71339147	1.84272667
H	-9.03125481	-4.69423265	0.49015567
H	-10.52218936	-3.15597800	1.77498779

INT-IV

Charge = 1   Spin Multiplicity = 2

Co	1.39376002	-0.45312864	0.11590906
O	3.03919568	0.37999704	-0.02984325
N	2.13426719	-2.11803023	0.55136807
O	0.66277526	1.21799650	0.31420056
N	-0.27306576	-1.29910348	0.47651832
C	4.46540180	-1.41794283	0.63182208
C	-0.55790608	1.62586807	0.54102346
C	-1.62717031	0.70146691	0.76454795
C	-0.84696412	3.03057040	0.59033650
C	-2.93887917	1.15650749	1.05851809
C	-2.15610007	3.40337907	0.86686814
C	-3.22518277	2.50439211	1.12431844

H	-3.70825538	0.40452880	1.22895164
H	-2.38157358	4.46665444	0.90652354
C	4.23776025	-0.08387307	0.14323231
C	5.38315123	0.74493139	-0.13726424
C	6.63447202	0.21522903	0.13124475
C	5.78434654	-1.88284613	0.89382288
C	6.88112662	-1.08596533	0.65483960
H	7.50189876	0.83936989	-0.07091057
H	5.89399426	-2.89778737	1.27402344
C	3.40426547	-2.33097550	0.82085819
H	3.67944632	-3.32308528	1.19401458
C	-1.40389450	-0.69782622	0.75301637
H	-2.26183372	-1.32349278	1.01939309
C	-0.15468128	-2.70765799	0.52477653
C	-1.21106026	-3.62513622	0.49650386
C	-0.92824997	-4.99116425	0.53124481
C	0.39718492	-5.44083110	0.58019869
C	1.45498442	-4.53067189	0.59266338
C	1.17692381	-3.15894757	0.57665958
H	-1.74912642	-5.70980731	0.50302247
H	-2.24564223	-3.28898106	0.43227260
H	0.61096063	-6.51105829	0.59038535
H	2.48179207	-4.89556549	0.59660715
C	0.26492374	4.07550376	0.40815393
C	1.01149834	3.85986493	-0.92655176
H	1.50111983	2.88191896	-0.96214915
H	0.31526019	3.93790128	-1.77651997
H	1.78405306	4.63467124	-1.04981362
C	-0.29265668	5.50881090	0.39740637
H	-0.78664571	5.77215818	1.34490745
H	0.53763616	6.21623417	0.25259308
H	-1.01103646	5.66381989	-0.42265792
C	1.23639499	3.95341117	1.60312343
H	0.71128944	4.17631955	2.54518508
H	1.65957774	2.94397437	1.67207036
H	2.06590323	4.66954446	1.49529960
C	5.21854290	2.16779062	-0.69418740
C	4.44826081	2.12214618	-2.03066062
H	3.44733981	1.70054209	-1.89396970
H	4.33866133	3.13954088	-2.43762967
H	4.98747653	1.51305776	-2.77333771
C	4.45107212	3.02848074	0.32924217

H	3.46164581	2.60932373	0.53355346
H	5.00859592	3.09403075	1.27708266
H	4.31767294	4.05044161	-0.05941695
C	6.57501916	2.84073489	-0.96071106
H	7.16982671	2.95331165	-0.04142749
H	7.17596051	2.28449026	-1.69675609
H	6.40134232	3.84865296	-1.36657062
C	-4.60972676	3.05981353	1.47907279
C	-5.15464614	3.87217516	0.28586659
H	-6.14154270	4.29533408	0.53184292
H	-4.48764959	4.70722891	0.02275792
H	-5.26672441	3.23311617	-0.60187666
C	-5.60343700	1.93661866	1.80995956
H	-5.73805661	1.25131970	0.96238851
H	-5.27434862	1.34764335	2.68006166
H	-6.58648581	2.36882356	2.05119842
C	-4.48593405	3.97887329	2.71330745
H	-4.08431481	3.42439668	3.57592973
H	-3.82574402	4.83855665	2.52420809
H	-5.47600636	4.37432991	2.99029143
C	8.32554161	-1.53090065	0.90787311
C	8.98436874	-0.56475203	1.91567354
H	10.02580055	-0.86851289	2.10678252
H	9.00248527	0.47000065	1.54174850
H	8.44317401	-0.57038844	2.87472953
C	9.10584658	-1.49937832	-0.42377577
H	9.12397252	-0.49163960	-0.86535155
H	10.14965644	-1.81085418	-0.25978187
H	8.65399798	-2.18425736	-1.15827445
C	8.39277233	-2.95412119	1.48048452
H	7.86361777	-3.03159760	2.44278810
H	7.96065618	-3.69324807	0.78849129
H	9.44281466	-3.23433288	1.65354956
C	1.31454694	-0.91242261	-1.96931036
H	0.51938177	-1.65996464	-1.87552945
C	2.62485165	-1.52349495	-2.38791898
H	2.50107177	-1.84251724	-3.43957099
H	3.45424216	-0.80440164	-2.36998979
H	2.89697288	-2.41640826	-1.81741926
C	0.94471561	0.30970472	-2.77552183
H	1.12995543	-0.01728704	-3.81910989
H	1.68012782	1.10986003	-2.60846402

C	-0.46981343	0.88066777	-2.70128716
H	-0.61567172	1.55413609	-3.56002758
H	-0.59544437	1.50056718	-1.80773506
C	-1.56320702	-0.17027273	-2.70332477
H	-1.44734476	-0.82719128	-1.83244129
H	-1.52656819	-0.80790202	-3.60548822
O	-2.81217926	0.50293349	-2.63348453
C	-3.88436076	-0.11524864	-2.08342489
C	-5.05176098	0.65730274	-1.97042772
C	-3.89767979	-1.43983869	-1.62101874
C	-6.18695349	0.13425507	-1.36512120
H	-5.03852090	1.67614780	-2.36067267
C	-5.04337097	-1.94725270	-1.00126749
H	-3.02778416	-2.08558457	-1.73237844
C	-6.20680681	-1.17680602	-0.84614184
H	-7.08160557	0.75606142	-1.29907105
H	-5.01524150	-2.96858688	-0.61599737
C	-7.40628338	-1.71461994	-0.15870355
C	-8.24011752	-0.87311301	0.60303477
C	-7.74160781	-3.07987464	-0.23904080
C	-9.36465493	-1.37741198	1.25955237
H	-7.99292966	0.18534680	0.70322478
C	-8.86456493	-3.58502411	0.41925464
H	-7.12743646	-3.75167926	-0.84220491
C	-9.68271821	-2.73642644	1.17214134
H	-9.99133093	-0.70486761	1.85038454
H	-9.10677827	-4.64738382	0.33607898
H	-10.56185143	-3.13143251	1.68654873

INT-V

Charge = 1 Spin Multiplicity = 1

C	-1.56752199	1.38024743	-0.49483556
C	-0.19768553	1.60031827	-0.57492623
C	0.70687784	0.57796906	-0.24018362
C	0.21019372	-0.66689393	0.17358055
C	-1.17041154	-0.87202603	0.24646130
C	-2.08983578	0.13711201	-0.08188779
H	-2.24812923	2.18507889	-0.77974247
H	0.20033318	2.56222333	-0.90400208
H	0.88281393	-1.47840860	0.44906937
H	-1.53410637	-1.84187742	0.59202324
O	2.02442793	0.88253706	-0.34526270

C	2.99482831	-0.09081939	0.00425915
H	2.88554638	-0.98863339	-0.63064784
H	2.86059811	-0.40523631	1.05513210
C	4.36296824	0.54027151	-0.19091113
H	4.43754719	0.85496469	-1.24629788
H	4.42353751	1.45287786	0.42390810
C	5.49423615	-0.41883152	0.17958223
H	5.38056300	-0.73929969	1.22872737
H	5.45252115	-1.33969357	-0.42644767
C	6.88065193	0.17970159	0.09548543
C	8.02132091	-0.72753350	0.47541081
H	8.98998831	-0.23568448	0.31434733
H	7.92634586	-0.96071788	1.54585456
C	-3.55331447	-0.09297608	0.00355808
C	-4.42983755	0.94368628	0.37793655
C	-4.10839628	-1.35427013	-0.28644469
C	-5.80718007	0.72817146	0.45863224
H	-4.02592372	1.92621026	0.63097492
C	-5.48527737	-1.57138169	-0.20318832
H	-3.45548416	-2.17035634	-0.60295677
C	-6.34263304	-0.53100676	0.16923683
H	-6.46528173	1.54752573	0.75830199
H	-5.89171248	-2.55777749	-0.43999393
H	-7.42005440	-0.70021088	0.23313123
O	7.16915362	0.61369303	-1.36168261
H	7.24589709	-0.13385133	-1.99183626
H	6.52605254	1.25779459	-1.72308133
H	7.98758273	-1.67657564	-0.08105393
H	6.94171470	1.14941077	0.60565629

^{t}AMO^{-}

Charge = -1 Spin Multiplicity = 1

C	-5.39631604	1.05154886	0.05396112
H	-5.22263111	-0.03415130	0.14820776
H	-4.87117954	1.54881261	0.88543204
H	-6.48197390	1.22288017	0.14914117
C	-4.81494010	1.61484161	-1.23852068
H	-5.17981437	2.65224681	-1.36316483
H	-5.17735646	1.04560624	-2.11714467
C	-3.23798762	1.69746770	-1.20381319
C	-2.81180692	2.43700555	-2.51346311
H	-3.18689529	3.47482941	-2.47634263

H	-1.70956304	2.48937782	-2.55906089
H	-3.17806737	1.96682661	-3.44637802
C	-2.67751037	0.23773233	-1.31486173
H	-2.97300948	-0.34442019	-0.42566614
H	-3.00463563	-0.31541087	-2.21604411
H	-1.57454565	0.28613441	-1.31599435
O	-2.78721093	2.32502699	-0.10750113

^{t}AMOH

Charge = 0 Spin Multiplicity = 1

C	-5.37911945	1.02158811	0.04158571
H	-5.16421595	-0.05767983	0.08239757
H	-4.95682590	1.48400629	0.94614706
H	-6.47340404	1.14106770	0.07422218
C	-4.80024043	1.67415387	-1.21237243
H	-5.14020022	2.72249743	-1.26672167
H	-5.18645366	1.17369738	-2.11601770
C	-3.26395993	1.69118936	-1.29316988
C	-2.82026597	2.43222724	-2.56016056
H	-3.22143906	3.45739693	-2.56237776
H	-1.72040845	2.49259842	-2.60859698
H	-3.17040912	1.91793459	-3.46885708
C	-2.67015478	0.27750312	-1.27013811
H	-2.91803486	-0.23794802	-0.33117789
H	-3.04553565	-0.32703804	-2.11065804
H	-1.57153498	0.32292043	-1.35252024
O	-2.82197901	2.41865212	-0.13846611
H	-1.85465494	2.41391517	-0.14153179

1a

Charge = 0 Spin Multiplicity = 1

C	-5.12623579	3.23945967	-1.47060887
C	-3.77033368	3.45772248	-1.25864975
C	-2.88948751	3.58064428	-2.34800802
C	-3.39955781	3.47942434	-3.65161833
C	-4.76598724	3.26107541	-3.84696636
C	-5.66057208	3.13614552	-2.77180961
H	-5.78973169	3.17015230	-0.60619860
H	-3.36540657	3.54729007	-0.24863802
H	-2.74519591	3.55917388	-4.51884486
H	-5.13502076	3.16394845	-4.87005573
O	-1.58625281	3.79263054	-2.04796186
C	-0.64055628	3.93799885	-3.10029560

H	-0.92692752	4.78414350	-3.75149578
H	-0.62821467	3.02649949	-3.72562897
C	0.72255164	4.17997915	-2.47796429
H	0.66608463	5.07819464	-1.83993486
H	0.96948938	3.33409040	-1.81520285
C	1.82219976	4.35263243	-3.53435880
H	1.88336907	3.44952294	-4.16497973
H	1.54089477	5.18774632	-4.20289860
C	3.16570911	4.64128662	-2.92784571
H	3.22564584	5.55250112	-2.31752124
C	4.25507829	3.87946756	-3.06371903
H	5.20371063	4.14588491	-2.58884483
H	4.23795731	2.96014460	-3.65948395
C	-7.10929723	2.90450601	-2.99418820
C	-7.86736976	2.12999770	-2.09474750
C	-7.76972839	3.45206636	-4.11117139
C	-9.23101296	1.91266777	-2.30269728
H	-7.37843450	1.67310588	-1.23185756
C	-9.13245488	3.23220280	-4.32189245
H	-7.21370819	4.07710339	-4.81301006
C	-9.87136618	2.46181301	-3.41819300
H	-9.79481066	1.30246850	-1.59271241
H	-9.62213198	3.67391055	-5.19333011
H	-10.93796671	2.29107350	-3.58183696

[Co^{III}-H]

Charge = 0 Spin Multiplicity = 1

Co	-0.00306694	0.87698102	0.05685428
O	1.27718655	-0.49352168	0.01551494
N	1.28365158	2.25945001	-0.03717443
O	-1.28219573	-0.47905351	-0.13448983
N	-1.28935232	2.26284621	-0.01609732
C	3.26454914	0.83478965	-0.06779021
C	-2.57169404	-0.41238969	-0.07742242
C	-3.26970781	0.83899602	0.03638618
C	-3.36386010	-1.61905803	-0.15409666
C	-4.69166768	0.87577928	0.11859517
C	-4.74102071	-1.50165019	-0.06412627
C	-5.44780695	-0.27445000	0.08168024
H	-5.16167480	1.85556235	0.20973306
H	-5.33384170	-2.41316290	-0.11114901
C	2.56657738	-0.41858599	0.02753035

C	3.36036340	-1.62342999	0.12241160
C	4.73877544	-1.50306165	0.06433418
C	4.68799085	0.87407253	-0.12107420
C	5.44593803	-0.27402760	-0.06565521
H	5.33259452	-2.41292431	0.12860926
H	5.15748916	1.85484742	-0.20392819
C	2.57995119	2.07802816	-0.10731122
H	3.21972530	2.96276003	-0.20060457
C	-2.58685874	2.08419263	0.03149713
H	-3.22928555	2.97134658	0.06090140
C	-0.70831437	3.54818513	-0.01582490
C	-1.40244669	4.76871681	-0.00789394
C	-0.69833060	5.97060182	-0.00078309
C	0.70352753	5.96841587	0.00237856
C	1.40386186	4.76432692	-0.00619475
C	0.70583702	3.54605066	-0.02325078
H	-1.24546590	6.91547442	0.00525026
H	-2.49190567	4.79144599	-0.00847127
H	1.25355759	6.91155170	0.01433955
H	2.49336463	4.78322311	0.00474480
C	-2.68331811	-2.98390234	-0.34967212
C	-1.70357142	-3.25730359	0.81087370
H	-0.92465144	-2.49001483	0.85588880
H	-2.24170123	-3.27681556	1.77272717
H	-1.21815475	-4.23705271	0.67385878
C	-3.69987659	-4.13686880	-0.38655527
H	-4.41697129	-4.03302772	-1.21566888
H	-3.16318705	-5.08730257	-0.53014984
H	-4.26926679	-4.21571614	0.55277030
C	-1.92641683	-2.98167333	-1.69626058
H	-2.63026810	-2.83991895	-2.53227665
H	-1.17913797	-2.18026419	-1.72608328
H	-1.40981531	-3.94366888	-1.84436753
C	2.67824438	-2.98787689	0.31514328
C	1.89291817	-2.96890491	1.64534303
H	1.15120566	-2.16177028	1.65320515
H	1.36616789	-3.92562376	1.79127186
H	2.58072783	-2.82476476	2.49413811
C	1.72541997	-3.28062564	-0.86336787
H	0.93927971	-2.52234061	-0.93184647
H	2.28396415	-3.30247262	-1.81352891
H	1.24823223	-4.26475144	-0.72815718

C	3.69649946	-4.13762241	0.38898280
H	4.28567852	-4.22749360	-0.53709960
H	4.39577763	-4.02065849	1.23140487
H	3.15922638	-5.08749518	0.53410065
C	-6.97899783	-0.29352994	0.17623539
C	-7.40500816	-1.12998635	1.40153265
H	-8.50364295	-1.16179068	1.48250662
H	-7.04522641	-2.16782012	1.33283120
H	-7.00480550	-0.69385321	2.33042790
C	-7.56041251	1.11984376	0.32811785
H	-7.19273391	1.61534484	1.23997609
H	-7.31050411	1.75802497	-0.53352923
H	-8.65786877	1.06612742	0.39629986
C	-7.56454720	-0.92624770	-1.10418610
H	-7.27859683	-0.34309728	-1.99378880
H	-7.21144122	-1.95856241	-1.24863557
H	-8.66497956	-0.95407619	-1.05092078
C	6.97896224	-0.28906497	-0.12434676
C	7.43530543	-1.12129834	-1.34155975
H	8.53560504	-1.15131569	-1.39669453
H	7.07532829	-2.15977357	-1.28436386
H	7.05652750	-0.68296645	-2.27838282
C	7.53648887	-0.92341898	1.16771065
H	7.18369293	-1.95730173	1.30118117
H	8.63795899	-0.94750163	1.14019194
H	7.22783701	-0.34366846	2.05192690
C	7.56017083	1.12614639	-0.25909396
H	7.21294004	1.62308242	-1.17815597
H	7.28825534	1.76149642	0.59798403
H	8.65906898	1.07538835	-0.30148624
H	-0.02547680	0.94860775	1.47873098

References

[1] Zhao Y, Ge S. Synergistic hydrocobaltation and borylcobaltation enable regioselective migratory triborylation of unactivated alkenes. *Angew Chem Int Ed* 2022; **61**: e202116133.

[2] Liu M, Feng T, Wang Y et al. Metal-free electrochemical dihydroxylation of unactivated alkenes. *Nat Commun* 2023; **14**: 6467.

[3] Tortajada A, Ninokata R, Martin R. Ni-catalyzed site-selective dicarboxylation of 1,3-dienes with CO_2. *J Am Chem Soc* 2018; **140**: 2050−3.

[4] Abel BA, Lidston CAL, Coates GW. Mechanism-inspired design of bifunctional catalysts for the alternating ring-opening copolymerization of epoxides and cyclic anhydrides. *J Am Chem Soc* 2019; **141**: 12760−9.

[5] Kamei Y, Seino Y, Yamaguchi Y et al. Silane- and peroxide-free hydrogen atom transfer hydrogenation using ascorbic acid and cobalt-photoredox dual catalysis. *Nat Commun* 2021; **12**: 966.

[6] Kobayashi Y, Obayashi R, Watanabe Y et al. Unprecedented asymmetric epoxidation of isolated carbon–carbon double bonds by a chiral fluorous Fe(III) salen complex: exploiting fluorophilic effect for catalyst design. *Eur J Org Chem* 2019; **13**: 2401−8.

[7] Discolo CA, Touney EE, Pronin SV. Catalytic asymmetric radical–polar crossover hydroalkoxylation. *J Am Chem Soc* 2019; **141**: 17527−32.

[8] Gaspar B, Carreira EM, Catalytic Hydrochlorination of unactivated olefins with para-toluenesulfonyl chloride. *Angew Chem Int Ed* 2008; **47**: 5758 –60.

[9] Gandhamsetty N, Park S, Chang S. Selective silylative reduction of pyridines leading to structurally diverse azacyclic compounds with the formation of sp3 C–Si bonds. *J Am Chem Soc* 2015; **137**: 15176−84.

[10] Zhang Z-Q, Meng X-Y, Sheng J et al. Enantioselective copper-catalyzed 1,5-cyanotrifluoromethylation of vinylcyclopropanes. *Org Lett* 2019; **21**: 8256−60.

[11] Zhao L-M, Wan L-J, Jin H-S et al. High regiocontrol in the zinc-mediated crotylation of aldehydes and ketones: a straightforward and facile approach to linear homoallylic alcohols in DMPU. *Eur J Org Chem* 2012; **13**: 2579–84.

[12] Dang H, Cox N, Lalic G. Copper-catalyzed reduction of alkyl triflates and iodides: an efficient method for the deoxygenation of primary and secondary alcohols. *Angew Chem Int Ed* 2014; **53**: 752 –6.

[13] Hao H-Y, Mao Y-J, Xu Z-Y et al. Synthesis and characterization of Rh^{III}–M^{II} (M = Pt, Pd) heterobimetallic complexes based on a bisphosphine ligand: tandem reactions using ethanol. *Organometallics* 2020; **39**: 3879−91.

[14] Paolillo JM, Duke AD, Gogarnoiu ES et al. Anaerobic hydroxylation of C(sp^3)–H bonds enabled by the synergistic nature of photoexcited nitroarenes. *J Am Chem Soc* 2023; **145**: 2794–99.

[15] Yasukawa T, Kobayashi S. Oxygenation of styrenes catalyzed by *N*-doped carbon incarcerated cobalt nanoparticles. *Bull Chem Soc Jpn* 2019; **92**: 1980–5.

[16] Du W, Wang L, Wu P et al. A versatile ruthenium(II)–NNC complex catalyst for

transfer hydrogenation of ketones and oppenauer-type oxidation of alcohols. *Chem Eur J* 2012; **18**: 11550 –4.
[17] Ramachandran PV, Alawaed AA, Hamann HJ. $TiCl_4$-catalyzed hydroboration of ketones with ammonia borane. *J Org Chem* 2022; **87**: 13259–69.
[18] Albarran-Velo J, Lavandera I, Gotor-Fernandez V, Sequential two-step stereoselective amination of allylic alcohols through the combination of laccases and amine transaminases. *ChemBioChem* 2020; **21**: 200 –11.
[19] Sun H-L, Yang F, Ye W-T et al. Dual cobalt and photoredox catalysis enabled intermolecular oxidative hydrofunctionalization. *ACS Catal* 2020; **10**: 4983–9.
[20] Shevick SL, Obradors C, Shenvi RA, Mechanistic interrogation of Co/Ni-dual catalyzed hydroarylation. *J Am Chem Soc* 2018; **140**: 12056−60.
[21] Wilson CV, Kim D, Sharma A et al. Cobalt–carbon bonding in a salen-supported cobalt(IV) alkyl complex postulated in oxidative MHAT catalysis. *J Am Chem Soc* 2022; **144**: 10361−7.
[22] Li W-T, Guan M-H, He P et al. Iron-catalyzed transfer hydroalumination of alkynes. *J Am Chem Soc* 2025; **147**: 15545–53.
[23] Gaussian 16, Revision A.03, M. J. Frisch. *et.al*., Gaussian, Inc., Wallingford CT, **2016**.
[24] Y. Zhao, D. G. Truhlar. *Theor Chem Acc* **2008**, 120, 215
[25] S. Grimme, J. Antony, S. Ehrlich, and H. Krieg. *J Chem Phys* **2010**, 132, 154104
[26] J. Zheng, X. Xu, and D. G. Truhlar. *Theor Chem Acc* **2011**, 128, 295.
[27] A. V. Marenich, C. J. Cramer, D. G. Truhlar, *J Phys Chem* **2009**, 113, 6378.
[28] F. Weigend and R. Ahlrichs, *Phys. Chem Chem Phys* **2005**, 7, 3297.
[29] J.Tomasia, B.Mennuccia, and E.Cancèsb, *J Mol Struct (Theochem)* **1999**, 464, 211.
[30] T. Lu, Q. Chen, *Comput Theor Chem* **2021**, 1200, 113249
[31] S. Grimme, *Chem Eur J* **2012**, 18, 9955.
[32] T. Feng, Z. Zhu, D. Zhang, S. Wang, R. Li, Z. Zhu, X. Zhang, Y. Qiu. *Green Chem* **2023**, 25, 2681.

Characterization Data of Products

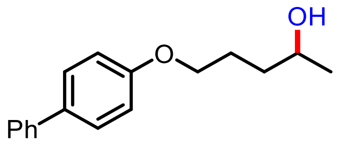

5-([1,1'-biphenyl]-4-yloxy) pentan-2-ol (1)

Compound **1** was prepared following the general procedure, purification by column chromatography on silica gel (petroleum ether/EtOAc = 5:1) yielded **1** (47.0 mg, 92 %) as a white solid. M.p.: 87 – 88 °C. ^{1}H NMR (400 MHz, Chloroform-d) δ 7.63 – 7.50 (m, 4H), 7.43 (t, J = 7.6 Hz, 2H), 7.32 (t, J = 7.4 Hz, 1H), 7.03 – 6.94 (m, 2H), 4.10 – 4.00 (m, 2H), 3.96 – 3.86 (m, 1H), 2.03 – 1.84 (m, 2H), 1.78 (s, 1H), 1.74 – 1.59 (m, 2H), 1.26 (d, J = 6.2 Hz, 3H). ^{13}C NMR (150 MHz, Chloroform-d) 158.5, 140.8, 133.8, 128.8, 128.2, 126.8, 126.7, 114.8, 68.1, 67.8, 35.9, 25.7, 23.7. HRMS (ESI, m/z): Calculated $C_{17}H_{20}O_2$ $[M+Na]^+$: 279.1356, found 279.1356.

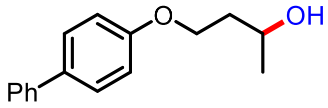

4-([1,1'-biphenyl]-4-yloxy) butan-2-ol (2)

Compound **2** was prepared following the general procedure, purification by column chromatography on silica gel (petroleum ether/EtOAc = 5:1) yielded **2** (42.0 mg, 87%) as a green solid. ^{1}H NMR (400 MHz, Chloroform-d) δ 7.58 – 7.51 (m, 4H), 7.42 (t, J = 8.0 Hz, 2H), 7.35 - 7.28 (m, 1H), 7.02 - 6.95 (m, 2H), 4.26 – 4.18 (m, 1H), 4.18 – 4.08 (m, 2H), 2.00 - 1.91 (m, 2H), 1.29 (d, J = 6.0 Hz, 3H). ^{13}C NMR (100 MHz, Chloroform-d) δ 158.3, 140.8, 134.4, 134.0, 128.7, 128.2, 126.8, 126.7, 114.8, 66.3, 66.0, 38.1, 23.7. Spectroscopic data match those previously reported in the literature.[12]

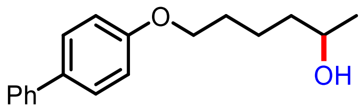

6-([1,1'-biphenyl]-4-yloxy) hexan-2-ol (3)

Compound **3** was prepared following the general procedure, purification by column chromatography on silica gel (petroleum ether/EtOAc = 5:1) yielded **3** (39.2 mg, 73%) as a light yellow solid. M.p.: 88 – 91 °C. ^{1}H NMR (400 MHz, Chloroform-d) δ 7.62 –

7.48 (m, 4H), 7.42 (t, J = 7.6 Hz, 2H), 7.30 (t, J = 7.4 Hz, 1H), 7.04 – 6.92 (m, 2H), 4.02 (t, J = 6.4 Hz, 2H), 3.91 – 3.70 (m, 1H), 1.91 – 1.77 (m, 2H), 1.69 – 1.49 (m, 4H), 1.36 (s, 1H), 1.22 (d, J = 6.1 Hz, 3H). ^{13}C NMR (100 MHz, Chloroform-d) δ 158.6, 140.9, 133.7, 128.7, 128.1, 126.7, 126.6, 114.8, 68.1, 67.9, 39.0, 29.3, 23.6, 22.4. HRMS (ESI, m/z): Calculated $C_{18}H_{22}O_2$ [M+Na]$^+$: 293.1512, found 293.1512.

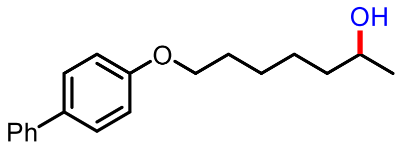

7-([1,1'-biphenyl]-4-yloxy) heptan-2-ol (4)

Compound **4** was prepared following the general procedure, purification by column chromatography on silica gel (petroleum ether/EtOAc = 5:1) yielded **4** (29.0 mg, 51%) as a light yellow solid. ^{1}H NMR (400 MHz, Chloroform-d) δ 7.58 (dd, J = 14.0, 8.0 Hz, 4H), 7.45 (t, J = 7.6 Hz, 2H), 7.40 – 7.30 (m, 1H), 7.01 (d, J = 8.0 Hz, 2H), 4.04 (t, J = 6.4 Hz, 2H), 3.91 - 3.81 (m, 1H), 1.86 (p, J = 6.4 Hz, 2H), 1.60 – 1.45 (m, 7H), 1.24 (d, J = 6.4 Hz, 3H). ^{13}C NMR (100 MHz, Chloroform-d) δ 158.7, 140.9, 134.4, 133.6, 128.7, 128.1, 126.7, 126.6, 114.8, 68.1, 68.0, 39.3, 29.3, 26.1, 25.6, 23.6. Spectroscopic data match those previously reported in the literature.[12]

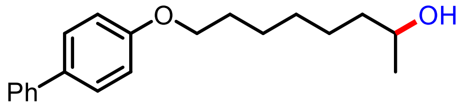

8-([1,1'-biphenyl]-4-yloxy) octan-2-ol (5)

Compound **5** was prepared following the general procedure, purification by column chromatography on silica gel (petroleum ether/EtOAc = 5:1) yielded **5** (17.9 mg, 30 %) as a light yellow solid. ^{1}H NMR (400 MHz, Chloroform-*d*) δ 7.58 – 7.50 (m, 4H), 7.45 – 7.39 (m, 2H), 7.35 – 7.28 (m, 1H), 7.01 – 6.94 (m, 2H), 4.00 (t, *J* = 6.4 Hz, 2H), 3.86 – 3.76 (m, 1H), 1.87 – 1.78 (m, 2H), 1.55 – 1.35 (m, 9H), 1.20 (d, *J* = 6.0 Hz, 3H). ^{13}C NMR (100 MHz, Chloroform-*d*) δ 158.7, 140.9, 134.4, 133.6, 128.7, 128.1, 126.7, 126.6, 114.8, 68.2, 68.0, 39.3, 29.4, 29.2, 26.1, 25.7, 23.5. Spectroscopic data match those previously reported in the literature.[12]

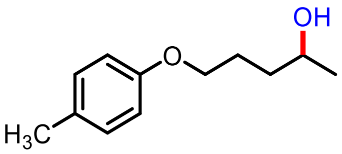

5-(*p*-tolyloxy) pentan-2-ol (6)

Compound **6** was prepared following the general procedure, purification by column chromatography on silica gel (petroleum ether/EtOAc = 5:1) yielded **6** (35.3 mg, 91%) as a yellow liquid. ^{1}H NMR (400 MHz, Chloroform-d) δ 7.07 (d, J = 8.4 Hz, 2H), 6.80 (d, J = 8.8 Hz, 2H), 4.00 - 3.93 (m, 2H), 3.91 – 3.84 (m, 1H), 2.28 (s, 3H), 1.95 – 1.81 (m, 2H), 1.66 – 1.56 (m, 2H), 1.23 (d, J = 6.0 Hz, 3H). ^{13}C NMR (100 MHz, Chloroform-d) δ 156.7, 129.9, 129.9, 114.4, 68.0, 67.8, 36.0, 25.7, 23.6, 20.5. HRMS (ESI, m/z): Calculated $C_{12}H_{18}O_2$ [M+H]$^+$: 195.1380, found 195.1379.

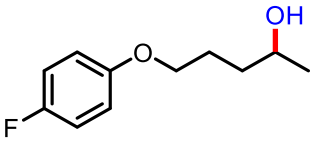

5-(4-fluorophenoxy) pentan-2-ol (7)

Compound **7** was prepared following the general procedure, purification by column chromatography on silica gel (petroleum ether/EtOAc = 5:1) yielded **7** (22.1 mg, 56 %) as a yellow liquid. ^{1}H NMR (400 MHz, Chloroform-*d*) δ 7.00 – 6.91 (m, 2H), 6.86 – 6.78 (m, 2H), 3.99 – 3.91 (m, 2H), 3.91 – 3.83 (m, 1H), 1.95 – 1.78 (m, 2H), 1.72 – 1.54 (m, 3H), 1.23 (d, J = 6.0 Hz, 3H). ^{13}C NMR (100 MHz, Chloroform-*d*) δ 157.2 (d, *J* = 238.2 Hz), 155.0 (d, *J* = 1.9 Hz), 134.4, 127.8, 115.8 (d, *J* = 23.0 Hz), 115.4 (d, *J* = 8.0 Hz), 68.6, 67.8, 35.8, 25.7, 23.6. ^{19}F NMR (375 MHz, Chloroform-*d*) δ -124.13. HRMS (ESI, m/z): Calculated $C_{11}H_{15}FO_2$ [M+Na]$^+$: 221.0948, found 221.0946.

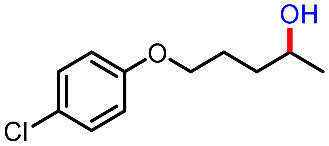

5-(4-chlorophenoxy) pentan-2-ol (8)

Compound **8** was prepared following the general procedure, purification by column chromatography on silica gel (petroleum ether/EtOAc = 5:1) yielded **8** (36.0 mg, 84%) as a yellow liquid. ^{1}H NMR (400 MHz, Chloroform-d) δ 7.22 (d, J = 8.8 Hz, 2H), 6.81

(d, J = 8.8 Hz, 2H), 4.00 - 3.92 (m, 2H), 3.91 – 3.82 (m, 1H), 1.97 – 1.78 (m, 2H), 1.66 – 1.53 (m, 2H), 1.23 (d, J = 6.4 Hz, 3H). ^{13}C NMR (100 MHz, Chloroform-d) δ 157.5, 129.3, 125.5, 115.8, 68.3, 67.8, 35.8, 25.6, 23.7. Spectroscopic data match those previously reported in the literature.[12]

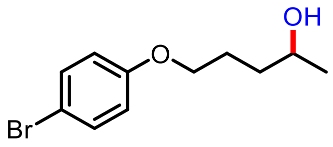

5-(4-bromophenoxy) pentan-2-ol (9)

Compound **9** was prepared following the general procedure, purification by column chromatography on silica gel (petroleum ether/EtOAc = 5:1) yielded **9** (34.9 mg, 67%) as a yellow liquid. ^{1}H NMR (400 MHz, Chloroform-d) δ 7.39 – 7.32 (m, 2H), 6.80 – 6.73 (m, 2H), 3.99 - 3.91 (m, 2H), 3.91 – 3.82 (m, 1H), 1.97 – 1.77 (m, 2H), 1.72 (s, 1H), 1.68 – 1.53 (m, 2H), 1.22 (d, J = 6.1 Hz, 3H). ^{13}C NMR (100 MHz, Chloroform-d) δ 158.1, 132.3, 116.3, 112.8, 68.2, 67.8, 35.8, 25.6, 23.7. HRMS (ESI, m/z): Calculated $C_{11}H_{15}BrO_2$ [M+Na]$^+$: 281.0148, found 281.0160.

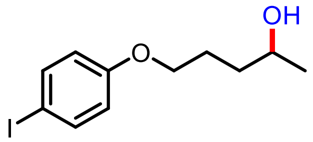

5-(4-iodophenoxy) pentan-2-ol (10)

Compound **10** was prepared following the general procedure, purification by column chromatography on silica gel (petroleum ether/EtOAc = 5:1) yielded **10** (40.4 mg, 66%) as a yellow liquid. ^{1}H NMR (400 MHz, Chloroform-d) δ 7.57 – 7.49 (m, 2H), 6.73 – 6.62 (m, 2H), 3.98 – 3.91 (m, 2H), 3.91 – 3.82 (m, 1H), 1.95 – 1.78 (m, 2H), 1.65 – 1.55 (m, 2H), 1.22 (d, J = 6.0 Hz, 3H).^{13}C NMR (100 MHz, Chloroform-d) δ 158.8, 138.2, 116.9, 82.7, 68.1, 67.7, 35.8, 25.6, 23.7. HRMS (ESI, m/z): Calculated $C_{11}H_{15}IO_2$ [M+Na]$^+$: 329.0009, found 329.0011.

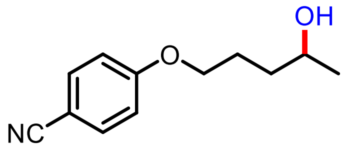

4-((4-hydroxypentyl) oxy) benzonitrile (11)

Compound **11** was prepared following the general procedure, purification by column chromatography on silica gel (petroleum ether/EtOAc = 3:1) yielded **11** (28.0 mg, 68%) as a yellow liquid. ^{1}H NMR (400 MHz, Chloroform-*d*) δ 7.56 (d, *J* = 8.8 Hz, 2H), 6.92 (d, *J* = 8.8 Hz, 2H), 4.08 – 3.98 (m, 2H), 3.92 – 3.87 (m, 1H), 2.00 – 1.80 (m, 2H), 1.65 – 1.55 (m, 2H), 1.23 (d, *J* = 6.4 Hz, 3H). ^{13}C NMR (100 MHz, Chloroform-d) δ 162.3, 134.0, 119.3, 115.2, 103.8, 68.3, 67.6, 35.5, 25.4, 23.7. HRMS (ESI, m/z): Calculated $C_{12}H_{15}NO_2$ $[M+H]^+$: 206.1176, found 206.1175.

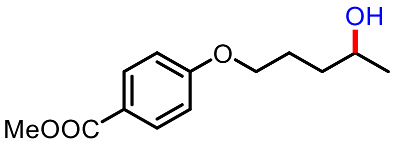

Methyl 4-((4-hydroxypentyl) oxy) benzoate (12)

Compound **12** was prepared following the general procedure, purification by column chromatography on silica gel (petroleum ether/EtOAc = 5:1) yielded **12** (33.7 mg, 71%) as a yellow solid. ^{1}H NMR (400 MHz, Chloroform-*d*) δ 8.00 (d, *J* = 8.8 Hz, 2H), 6.92 (d, *J* = 8.8 Hz, 2H), 4.09 – 4.04 (m, 2H), 3.95 – 3.86 (m, 4H), 2.02 – 1.84 (m, 2H), 1.69 – 1.62 (m, 2H), 1.26 (d, *J* = 6.4 Hz, 3H). ^{13}C NMR (100 MHz, Chloroform-*d*) δ 166.9, 162.8, 131.6, 122.5, 114.1, 68.1, 67.7, 51.9, 35.7, 25.5, 23.7. HRMS (ESI, m/z): Calculated $C_{13}H_{18}O_4$ $[M+Na]^+$: 261.1097, found 261.1092.

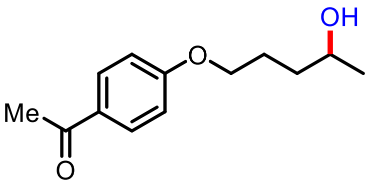

1-(4-((4-hydroxypentyl) oxy) phenyl) ethan-1-one (13)

Compound **13** was prepared following the general procedure, purification by column chromatography on silica gel (petroleum ether/EtOAc = 5:1) yielded **13** (29.8 mg, 67%) as a yellow liquid. ^{1}H NMR (400 MHz, Chloroform-*d*) δ 7.96 – 7.85 (m, 2H), 6.95 – 6.86 (m, 2H), 4.10 – 4.00 (m, 2H), 3.92 – 3.83 (m, 1H), 2.54 (s, 3H), 2.02 – 1.81 (m, 2H), 1.69 – 1.56 (m, 2H), 1.23 (d, *J* = 6.4 Hz, 3H). ^{13}C NMR (100 MHz, Chloroform-*d*) δ 196.9, 162.9, 130.6, 130.2, 114.1, 68.2, 67.7, 35.6, 26.3, 25.5, 23.7. HRMS (ESI, m/z): Calculated $C_{13}H_{18}O_3$ $[M+H]^+$: 223.1329, found 223.1332.

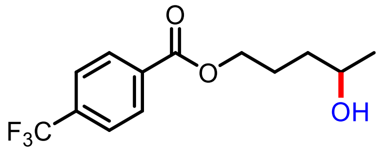

4-hydroxypentyl-4-(trifluoromethyl) benzoate (14)

Compound **14** was prepared following the general procedure, purification by column chromatography on silica gel (petroleum ether/EtOAc = 5:1) yielded **14** (33.2 mg, 60%) as a yellow liquid. ^{1}H NMR (400 MHz, Chloroform-*d*) δ 8.15 (d, *J* = 8.0 Hz, 2H), 7.71 (d, *J* = 8.4 Hz, 2H), 4.39 (t, *J* = 6.4 Hz, 2H), 3.94 – 3.84 (m, 1H), 1.99 – 1.80 (m, 2H), 1.67 – 1.57 (m, 3H), 1.25 (d, *J* = 6.4 Hz, 3H). ^{13}C NMR (150 MHz, Chloroform-*d*) δ 165.4, 134.4 (q, *J* = 32.6 Hz), 133.6, 129.6, 129.1, 125.4 (q, *J* = 3.8 Hz), 123.7 (q, *J* = 270.7 Hz), 67.6, 65.6, 35.4, 25.1, 23.7. ^{19}F NMR (376 MHz, Chloroform-*d*) δ -63.16. HRMS (ESI, m/z): Calculated $C_{13}H_{15}F_3O_3$ $[M+Na]^+$: 299.0866, found 299.0867.

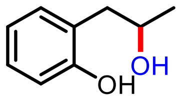

2-(2-hydroxypropyl) phenol (15)

Compound **15** was prepared following the general procedure, purification by column chromatography on silica gel (petroleum ether/EtOAc = 3:1) yielded **15** (19.8 mg, 65%) as a colorless liquid. ^{1}H NMR (400 MHz, Chloroform-d) δ 8.39 (s, 1H), 7.18 – 7.11 (m, 1H), 7.03 (d, J = 7.4 Hz, 1H), 6.93 – 6.82 (m, 2H), 4.25 – 4.16 (m, 1H), 3.17 (s, 1H), 2.85 (dd, J = 14.6, 2.9 Hz, 1H), 2.76 (dd, J = 14.5, 7.3 Hz, 1H), 1.25 (d, J = 6.2 Hz, 3H). ^{13}C NMR (100 MHz, Chloroform-d) δ 155.3, 131.8, 128.3, 125.3, 120.4, 117.0, 70.2, 40.6, 23.1. HRMS (ESI, m/z): Calculated $C_9H_{12}O_2$ $[M+Na]^+$: 175.0730, found 175.0728.

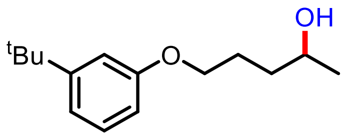

5-(3-(*tert*-butyl) phenoxy) pentan-2-ol (16)

Compound **16** was prepared following the general procedure, purification by column chromatography on silica gel (petroleum ether/EtOAc = 5:1) yielded **16** (38.3 mg, 81%)

as a yellow liquid. ^{1}H NMR (400 MHz, Chloroform-*d*) δ 7.25 (t, *J* = 8.0 Hz, 1H), 7.07 – 6.94 (m, 2H), 6.75 (dd, *J* = 8.4, 2.4 Hz, 1H), 4.08 – 3.98 (m, 2H), 3.97 – 3.87 (m, 1H), 1.99 – 1.83 (m, 3H), 1.76 – 1.59 (m, 2H), 1.35 (s, 9H), 1.27 (d, *J* = 6.4 Hz, 3H). ^{13}C NMR (100 MHz, Chloroform-*d*) δ 158.7, 153.0, 129.0, 117.9, 112.6, 110.6, 67.8, 67.8, 36.0, 34.8, 31.4, 25.8, 23.6. HRMS (ESI, m/z): Calculated $C_{15}H_{24}O_2$ $[M+Na]^+$: 259.1669, found 259.1667.

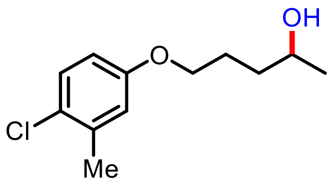

5-(4-chloro-3-methyl phenoxy) pentan-2-ol (17)

Compound **17** was prepared following the general procedure, purification by column chromatography on silica gel (petroleum ether/EtOAc = 5:1) yielded **17** (38.6 mg, 84%) as a yellow liquid. ^{1}H NMR (400 MHz, Chloroform-*d*) δ 7.20 (d, *J* = 8.8 Hz, 1H), 6.76 (d, *J* = 2.8 Hz, 1H), 6.66 (dd, *J* = 8.8, 2.8 Hz, 1H), 3.99 – 3.92 (m, 2H), 3.89 – 3.85 (m, 1H), 2.33 (s, 3H), 1.95 – 1.79 (m, 2H), 1.65 – 1.54 (m, 2H), 1.23 (d, *J* = 6.4 Hz, 3H). ^{13}C NMR (100 MHz, Chloroform-*d*) δ 157.5, 137.0, 129.6, 125.8, 117.1, 113.1, 68.2, 67.8, 35.8, 25.6, 23.6, 20.3. HRMS (ESI, m/z): Calculated $C_{12}H_{17}ClO_2$ $[M+Na]^+$: 251.0809, found 251.0816.

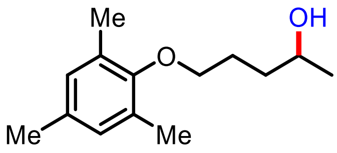

5-(mesityloxy) pentan-2-ol (18)

Compound **18** was prepared following the general procedure, purification by column chromatography on silica gel (petroleum ether/EtOAc = 5:1) yielded **18** (24.5 mg, 55%) as a yellow liquid. ^{1}H NMR (400 MHz, Chloroform-*d*) δ 6.84 (s, 2H), 3.98 – 3.88 (m, 1H), 3.83 – 3.74 (m, 2H), 2.26 (s, 9H), 1.98 – 1.85 (m, 3H), 1.79 – 1.64 (m, 2H), 1.27 (d, *J* = 6.4 Hz, 3H). ^{13}C NMR (150 MHz, Chloroform-*d*) δ 153.6, 133.1, 130.5, 129.4, 72.3, 67.9, 36.2, 26.9, 23.6, 20.7, 16.2. HRMS (ESI, m/z): Calculated $C_{14}H_{22}O_2$ $[M+Na]^+$: 245.1512, found 245.1512.

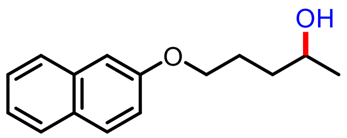

5-(naphthalen-2-yloxy) pentan-2-ol (19)

Compound **19** was prepared following the general procedure, purification by column chromatography on silica gel (petroleum ether/EtOAc = 5:1) yielded **19** (34.6 mg, 75%) as a yellow liquid. ^{1}H NMR (400 MHz, Chloroform-*d*) δ 7.81 – 7.68 (m, 3H), 7.44 (t, *J* = 7.2 Hz, 1H), 7.37 – 7.30 (m, 1H), 7.18 – 7.10 (m, 2H), 4.19 – 4.05 (m, 2H), 3.96 – 3.88 (m, 1H), 2.05 – 1.86 (m, 2H), 1.77 – 1.62 (m, 3H), 1.26 (d, *J* = 6.0 Hz, 3H). ^{13}C NMR (100 MHz, Chloroform-*d*) δ 156.9, 134.6, 129.4, 129.0, 127.7, 126.7, 126.4, 123.6, 118.9, 106.6, 68.0, 67.8, 36.0, 25.7, 23.7. HRMS (ESI, m/z): Calculated $C_{15}H_{18}O_2$ $[M+Na]^+$: 253.1199, found 253.1203.

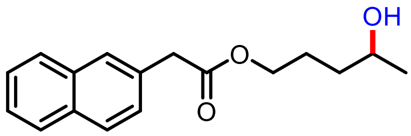

4-hydroxypentyl 2-(naphthalen-2-yl) acetate (20)

Compound **20** was prepared following the general procedure, purification by column chromatography on silica gel (petroleum ether/EtOAc = 5:1) yielded **20** (28.0 mg, 51%) as a yellow liquid. ^{1}H NMR (400 MHz, Chloroform-*d*) δ 7.85 – 7.76 (m, 3H), 7.74 (s, 1H), 7.50 – 7.40 (m, 3H), 4.13 (t, *J* = 6.6 Hz, 2H), 3.82 – 3.70 (m, 3H), 1.79 – 1.63 (m, 3H), 1.46 – 1.39 (m, 2H), 1.13 (d, *J* = 6.4 Hz, 3H). ^{13}C NMR (100 MHz, Chloroform-*d*) δ 171.7, 133.5, 132.8, 131.6, 128.2, 128.0, 127.7, 127.4, 126.2, 125.8, 67.6, 64.9, 41.7, 35.4, 25.0, 23.6. HRMS (ESI, m/z): Calculated $C_{17}H_{20}O_3$ $[M+Na]^+$: 295.1305, found 295.1307.

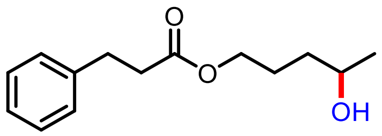

4-hydroxypentyl 3-phenylpropanoate (21)

Compound **21** was prepared following the general procedure, purification by column chromatography on silica gel (petroleum ether/EtOAc = 5:1) yielded **21** (33.8 mg, 72%)

as a yellow liquid. ^{1}H NMR (400 MHz, Chloroform-*d*) δ 7.35 – 7.28 (m, 2H), 7.26 – 7.18 (m, 3H), 4.11 (t, *J* = 6.6 Hz, 2H), 3.81 (h, *J* = 6.2 Hz, 1H), 2.97 (t, *J* = 7.8 Hz, 2H), 2.66 (t, *J* = 7.8 Hz, 2H), 1.83 – 1.61 (m, 3H), 1.51 – 1.41 (m, 2H), 1.21 (d, *J* = 6.4 Hz, 3H). ^{13}C NMR (100 MHz, Chloroform-*d*) δ 173.0, 140.5, 128.5, 128.3, 126.3, 67.6, 64.5, 35.9, 35.4, 31.0, 25.0, 23.6. HRMS (ESI, m/z): Calculated $C_{14}H_{20}O_3$ $[M+Na]^+$: 259.1305, found 259.1304.

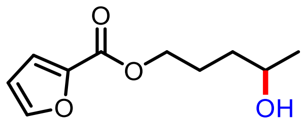

4-hydroxypentyl furan-2-carboxylate (22)

Compound **22** was prepared following the general procedure, purification by column chromatography on silica gel (petroleum ether/EtOAc = 5:1) yielded **22** (24.2 mg, 61%) as a yellow liquid. ^{1}H NMR (400 MHz, Chloroform-*d*) δ 7.57 (dd, *J* = 1.6, 0.8 Hz, 1H), 7.17 (dd, *J* = 3.6, 0.4 Hz, 1H), 6.50 (dd, *J* = 3.2, 1.6 Hz, 1H), 4.33 (t, *J* = 6.8 Hz, 2H), 3.86 (h, *J* = 6.4 Hz, 1H), 1.95 – 1.76 (m, 2H), 1.60 – 1.53 (m, 2H), 1.22 (d, *J* = 6.4 Hz, 3H). ^{13}C NMR (100 MHz, Chloroform-*d*) δ 158.8, 146.3, 144.7, 117.9, 111.8, 67.6, 65.0, 35.4, 25.1, 23.6. HRMS (ESI, m/z): Calculated $C_{10}H_{14}O_4$ $[M+Na]^+$: 221.0784, found 221.0792.

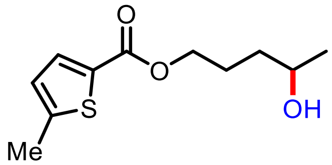

4-hydroxypentyl 5-methylthiophene-2-carboxylate (23)

Compound **23** was prepared following the general procedure, purification by column chromatography on silica gel (petroleum ether/EtOAc = 5:1) yielded **23** (31.1 mg, 68%) as a yellow solid. ^{1}H NMR (400 MHz, Chloroform-*d*) δ 7.58 (d, *J* = 3.6 Hz, 1H), 6.77 – 6.71 (m, 1H), 4.27 (t, *J* = 6.8 Hz, 2H), 3.84 (h, *J* = 6.4 Hz, 1H), 2.49 (s, 3H), 1.92 – 1.72 (m, 3H), 1.59 – 1.50 (m, 2H), 1.20 (d, *J* = 6.4 Hz, 3H). ^{13}C NMR (100 MHz, Chloroform-*d*) δ 162.4, 147.9, 133.8, 131.2, 126.4, 67.6, 64.9, 35.5, 25.1, 23.6, 15.8. HRMS (ESI, m/z): Calculated $C_{11}H_{16}O_3S$ $[M+Na]^+$: 251.0712, found 251.0713.

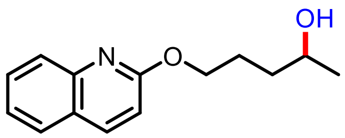

5-(quinolin-2-yloxy) pentan-2-ol (24)

Compound **24** was prepared following the general procedure, purification by column chromatography on silica gel (petroleum ether/EtOAc = 5:1) yielded **24** (28.2 mg, 61%) as a colorless liquid. ^{1}H NMR (600 MHz, Chloroform-*d*) δ 7.97 (d, *J* = 9.0 Hz, 1H), 7.84 (d, *J* = 8.4 Hz, 1H), 7.70 (d, *J* = 7.8 Hz, 1H), 7.61 (t, *J* = 7.8 Hz, 1H), 7.37 (t, *J* = 7.8 Hz, 1H), 6.89 (d, *J* = 8.4 Hz, 1H), 4.59 – 4.45 (m, 2H), 3.97 – 3.91 (m, 1H), 2.13 (s, 1H), 1.99 – 1.86 (m, 2H), 1.71 – 1.54 (m, 2H), 1.25 (d, *J* = 6.2 Hz, 3H). ^{13}C NMR (150 MHz, Chloroform-*d*) δ 162.2, 146.5, 138.8, 129.6, 127.4, 127.1, 125.0, 124.0, 113.3, 67.8, 65.7, 35.5, 25.3, 23.6. HRMS (ESI, m/z): Calculated $C_{14}H_{17}NO_2$ $[M+H]^+$: 232.1332, found 232.1328.

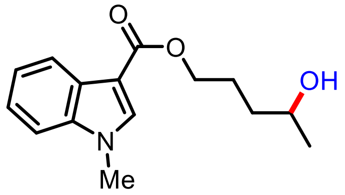

4-hydroxypentyl 1-methyl-1H-indole-3-carboxylate (25)

Compound **25** was prepared following the general procedure, purification by column chromatography on silica gel (petroleum ether/EtOAc = 5:1) yielded **25** (31.9 mg, 61%) as a yellow liquid. ^{1}H NMR (400 MHz, Chloroform-*d*) δ 8.19 (d, *J* = 9.4 Hz, 1H), 7.80 (s, 1H), 7.39 – 7.28 (m, 3H), 4.38 (t, *J* = 6.6 Hz, 2H), 3.92 (h, *J* = 6.2 Hz, 1H), 3.83 (s, 3H), 2.00 – 1.86 (m, 3H), 1.71 – 1.61 (m, 2H), 1.26 (d, *J* = 6.2 Hz, 3H). ^{13}C NMR (100 MHz, Chloroform-*d*) δ 165.2, 137.2, 135.3, 126.6, 122.8, 121.9, 121.6, 109.8, 107.0, 67.7, 63.7, 35.8, 33.4, 25.4, 23.6. HRMS (ESI, m/z): Calculated $C_{15}H_{19}NO_3$ $[M+Na]^+$: 284.1257, found 284.1256.

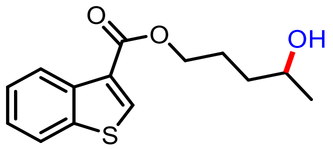

4-hydroxypentyl benzo[b]thiophene-3-carboxylate (26)

Compound **26** was prepared following the general procedure, purification by column chromatography on silica gel (petroleum ether/EtOAc = 5:1) yielded **26** (33.0 mg, 62%) as a pale-yellow liquid. ^{1}H NMR (400 MHz, Chloroform-*d*) δ 8.58 (d, *J* = 8.2 Hz, 1H), 8.37 (s, 1H), 7.86 (d, *J* = 8.2 Hz, 1H), 7.51 – 7.44 (m, 1H), 7.43 – 7.36 (m, 1H), 4.39 (t, *J* = 6.6 Hz, 2H), 3.89 (h, *J* = 6.2 Hz, 1H), 2.02 – 1.83 (m, 2H), 1.66 – 1.57 (m, 2H), 1.24 (d, *J* = 6.2 Hz, 3H). ^{13}C NMR (100 MHz, Chloroform-*d*) δ 162.9, 140.1, 136.7, 136.6, 127.3, 125.4, 125.0, 124.7, 122.5, 67.6, 64.7, 35.6, 25.2, 23.7. HRMS (ESI, m/z): Calculated $C_{14}H_{16}O_3S$ [M+Na]$^+$: 287.0712, found 287.0712.

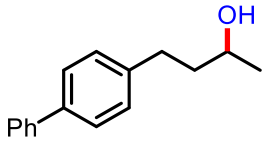

4-([1,1'-biphenyl]-4-yl) butan-2-ol (27)

Compound **27** as prepared following the general procedure, purification by column chromatography on silica gel (petroleum ether/EtOAc = 5:1) yielded **27** (39.1 mg, 86%) as a pale-yellow solid. ^{1}H NMR (400 MHz, Chloroform-d) δ 7.60 – 7.55 (m, 2H), 7.54 – 7.50 (m, 2H), 7.46 – 7.39 (m, 2H), 7.35 – 7.24 (m, 3H), 3.86 (h, J = 6.4 Hz, 1H), 2.88 – 2.62 (m, 2H), 1.90 – 1.73 (m, 2H), 1.25 (d, J = 6.0 Hz, 3H). ^{13}C NMR (100 MHz, Chloroform-d) δ 141.2, 141.1, 138.8, 128.9, 128.7, 127.2, 127.1, 127.0, 67.6, 40.8, 31.8, 23.7. Spectroscopic data match those previously reported in the literature.[13]

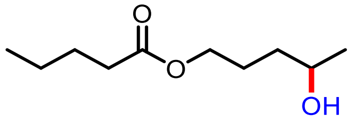

4-hydroxypentyl pentanoate (28)

Compound **28** was prepared following the general procedure, purification by column chromatography on silica gel (petroleum ether/EtOAc = 5:1) yielded **28** (26.3 mg, 70%) as a yellow liquid. ^{1}H NMR (400 MHz, Chloroform-*d*) δ 4.08 (t, *J* = 6.6 Hz, 2H), 3.82 (h, *J* = 6.2 Hz, 1H), 2.29 (t, *J* = 7.6 Hz, 2H), 1.80 – 1.65 (m, 2H), 1.64 – 1.54 (m, 3H), 1.53 – 1.46 (m, 2H), 1.39 – 1.28 (m, 2H), 1.20 (d, *J* = 6.1 Hz, 3H), 0.90 (t, *J* = 7.3 Hz, 3H). ^{13}C NMR (100 MHz, Chloroform-*d*) δ 174.0, 67.6, 64.2, 35.5, 34.1, 27.1, 25.0, 23.6, 22.3, 13.7. HRMS (ESI, m/z): Calculated $C_{10}H_{20}O_3$ [M+Na]$^+$: 211.1305, found

211.1306.

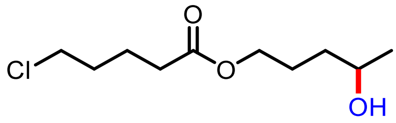

4-hydroxypentyl-5-chloropentanoate (29)

Compound **29** was prepared following the general procedure, purification by column chromatography on silica gel (petroleum ether/EtOAc = 5:1) yielded **29** (21.1 mg, 47%) as a yellow liquid. ^{1}H NMR (400 MHz, Chloroform-*d*) δ 4.08 (t, *J* = 6.7 Hz, 2H), 3.81 (h, *J* = 6.2 Hz, 1H), 3.53 (t, *J* = 6.1 Hz, 2H), 2.33 (t, *J* = 7.0 Hz, 2H), 1.81 – 1.62 (m, 7H), 1.52 – 1.44 (m, 2H), 1.19 (d, *J* = 6.2 Hz, 3H). ^{13}C NMR (100 MHz, Chloroform-*d*) δ 173.3, 67.6, 64.5, 44.4, 35.4, 33.4, 31.8, 25.0, 23.6, 22.2. HRMS (ESI, m/z): Calculated $C_{10}H_{19}ClO_3$ [M+Na]$^+$: 245.0915, found 245.0908.

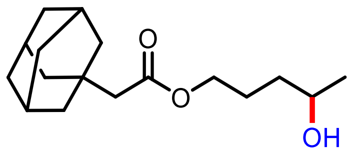

4-hydroxypentyl 2-(adamantan-1-yl) acetate (30)

Compound **30** was prepared following the general procedure, purification by column chromatography on silica gel (petroleum ether/EtOAc = 5:1) yielded **30** (49.4 mg, 88%) as a yellow liquid. ^{1}H NMR (400 MHz, Chloroform-*d*) δ 4.07 (t, *J* = 6.6 Hz, 2H), 3.83 (h, *J* = 6.2 Hz, 1H), 2.05 (s, 2H), 1.95 (d, *J* = 3.3 Hz, 3H), 1.73 – 1.55 (m, 15H), 1.55 – 1.46 (m, 2H), 1.20 (d, *J* = 6.2 Hz, 3H). ^{13}C NMR (150 MHz, Chloroform-*d*) δ 172.0, 67.6, 64.0, 49.0, 42.4, 36.7, 35.6, 32.8, 28.6, 25.1, 23.6. HRMS (ESI, m/z): Calculated $C_{17}H_{28}O_3$ [M+Na]$^+$: 303.1391, found 303.1391.

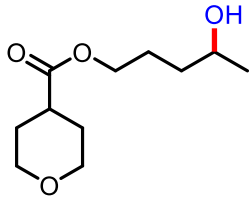

4-hydroxypentyl tetrahydro-2H-pyran-4-carboxylate (31)

Compound **31** was prepared following the general procedure, purification by column chromatography on silica gel (petroleum ether/EtOAc = 5:1) yielded **31** (27.5 mg, 64%)

as a yellow liquid. ^{1}H NMR (400 MHz, Chloroform-*d*) δ 4.11 (t, *J* = 6.6 Hz, 2H), 3.95 (dt, *J* = 11.6, 3.6 Hz, 2H), 3.82 (h, *J* = 6.2 Hz, 1H), 3.42 (td, *J* = 11.2, 3.0 Hz, 2H), 2.58 – 2.47 (m, 1H), 1.87 – 1.63 (m, 7H), 1.53 – 1.45 (m, 2H), 1.20 (d, *J* = 6.2 Hz, 3H). ^{13}C NMR (100 MHz, Chloroform-*d*) δ 174.6, 67.6, 67.1, 64.5, 40.2, 35.4, 28.7, 25.0, 23.7. HRMS (ESI, m/z): Calculated $C_{11}H_{20}O_4$ [M+Na]$^{+}$: 239.1254, found 239.1261.

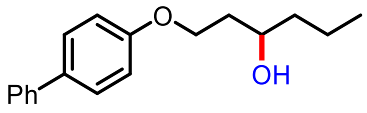

32, 53%

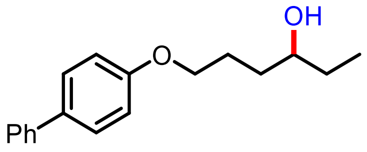

32', 7%

1-([1,1'-biphenyl]-4-yloxy) hexan-3-ol (32)

Compound **32** was prepared following the general procedure, purification by column chromatography on silica gel (petroleum ether/EtOAc = 5:1) yielded **32** (28.7 mg, 53%) as a white solid. M.p.: 83 – 85 °C. ^{1}H NMR (400 MHz, Chloroform-*d*) δ 7.60 – 7.50 (m, 4H), 7.47 – 7.38 (m, 2H), 7.35 – 7.28 (m, 1H), 7.02 – 6.95 (m, 2H), 4.11 – 4.00 (m, 2H), 3.68 – 3.56 (m, 1H), 2.05 – 1.84 (m, 2H), 1.80 – 1.68 (m, 2H), 1.65 – 1.45 (m, 3H), 0.99 (t, *J* = 7.4 Hz, 3H). ^{13}C NMR (101 MHz, Chloroform-*d*) δ 158.5, 140.9, 133.8, 128.7, 128.2, 126.7, 126.7, 114.8, 73.0, 68.1, 33.6, 30.4, 25.7, 10.0. ^{1}H NMR of **32'** (600 MHz, Chloroform-d) δ 8.11 (d, J = 8.1 Hz, 2H), 7.64 (dd, J = 22.5, 7.8 Hz, 4H), 7.47 (t, J = 7.6 Hz, 2H), 7.40 (t, J = 7.4 Hz, 1H), 4.38 (t, J = 6.6 Hz, 2H), 3.65 – 3.58 (m, 1H), 2.03 – 1.92 (m, 1H), 1.90 – 1.79 (m, 1H), 1.66 – 1.46 (m, 6H), 0.97 (t, J = 7.5 Hz, 3H). ^{13}C NMR of **32'** (150 MHz, Chloroform-d) δ 166.6, 145.6, 140.0, 130.1, 128.9, 128.2, 127.3, 127.1, 72.9, 65.1, 33.3, 30.3, 25.1, 9.9. HRMS (ESI, m/z): Calculated $C_{18}H_{22}O_2$ [M+Na]$^{+}$: 293.1512, found 293.1509.

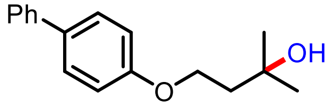

4-([1,1'-biphenyl]-4-yloxy)-2-methylbutan-2-ol (33)

Compound **33** was prepared following the general procedure, purification by column chromatography on silica gel (petroleum ether/EtOAc = 5:1) yielded **33** (20.3 mg, 40%)

as a yellow liquid. ^{1}H NMR (400 MHz, Chloroform-*d*) δ 7.61 – 7.53 (m, 4H), 7.45 (t, *J* = 7.6 Hz, 2H), 7.38 – 7.31 (m, 1H), 7.08 – 6.95 (m, 2H), 4.26 (t, *J* = 6.2 Hz, 2H), 2.05 (t, *J* = 6.0 Hz, 2H), 1.36 (s, 6H). ^{13}C NMR (100 MHz, Chloroform-*d*) δ 158.1, 140.8, 134.1, 128.8, 128.2, 126.8, 114.8, 70.4, 65.3, 41.7, 29.6. HRMS (ESI, m/z): Calculated $C_{17}H_{20}O_2$ $[M+Na]^+$: 279.1356, found 279.1356.

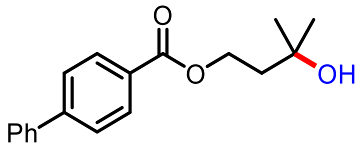

3-hydroxy-3-methylbutyl [1,1'-biphenyl]-4-carboxylate (34)

Compound **34** was prepared following the general procedure, purification by column chromatography on silica gel (petroleum ether/EtOAc = 5:1) yielded **34** (30.0 mg, 53%) as a yellow liquid. ^{1}H NMR (400 MHz, Chloroform-*d*) δ 8.14 – 8.09 (m, 2H), 7.70 – 7.61 (m, 4H), 7.52 – 7.45 (m, 2H), 7.45 – 7.39 (m, 1H), 4.57 (t, *J* = 6.8 Hz, 2H), 2.03 (t, *J* = 6.8 Hz, 2H), 1.98 (s, 1H), 1.37 (s, 6H). ^{13}C NMR (100 MHz, Chloroform-*d*) δ 166.6, 145.7, 140.0, 130.1, 129.0, 128.2, 127.3, 127.1, 70.1, 62.0, 41.8, 29.8. HRMS (ESI, m/z): Calculated $C_{18}H_{20}O_3$ $[M+Na]^+$: 307.1305, found 307.1304.

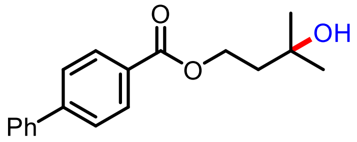

3-hydroxy-3-methylbutyl [1,1'-biphenyl]-4-carboxylate (35)

Compound **35** was prepared following the general procedure, purification by column chromatography on silica gel (petroleum ether/EtOAc = 5:1) yielded **35** (41.6 mg, 73%) a yellow liquid. ^{1}H NMR (400 MHz, Chloroform-*d*) δ 8.13 (d, *J* = 8.2 Hz, 2H), 7.70 – 7.62 (m, 4H), 7.49 (t, *J* = 7.2 Hz, 2H), 7.42 (t, *J* = 7.2 Hz, 1H), 4.57 (t, *J* = 6.8 Hz, 2H), 2.10 (s, 1H), 2.04 (t, *J* = 6.8 Hz, 2H), 1.38 (s, 6H). ^{13}C NMR (100 MHz, Chloroform-*d*) δ 166.6, 145.7, 140.0, 130.1, 129.0, 128.2, 127.3, 127.1, 70.1, 62.1, 41.8, 29.8. HRMS (ESI, m/z): Calculated $C_{18}H_{20}O_3$ $[M+Na]^+$: 307.1305, found 307.1303.

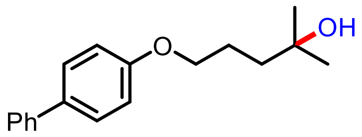

5-([1,1'-biphenyl]-4-yloxy)-2-methylpentan-2-ol (36)

Compound **36** was prepared following the general procedure, purification by column chromatography on silica gel (petroleum ether/EtOAc = 5:1) yielded **36** (29.2 mg, 54%) as a yellow solid. ^{1}H NMR (400 MHz, Chloroform-*d*) δ 7.59 – 7.50 (m, 4H), 7.45 – 7.38 (m, 2H), 7.33 – 7.28 (m, 1H), 7.00 – 6.95 (m, 2H), 4.04 (t, *J* = 6.3 Hz, 2H), 1.98 – 1.86 (m, 2H), 1.71 – 1.64 (m, 2H), 1.46 (s, 1H), 1.28 (s, 6H). ^{13}C NMR (100 MHz, Chloroform-*d*) δ 158.6, 140.9, 133.8, 128.7, 128.2, 126.7, 126.6, 114.8, 70.7, 68.5, 40.2, 29.4, 24.4.

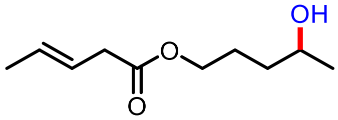

4-hydroxypentylpent-3-enoate (37)

Compound **37** was prepared following the general procedure, purification by column chromatography on silica gel (petroleum ether/EtOAc = 5:1) yielded **37** (19.5 mg, 52%) as a yellow liquid.^{1}H NMR (400 MHz, Chloroform-*d*) δ 5.62 – 5.46 (m, 2H), 4.08 (t, *J* = 6.6 Hz, 2H), 3.81 (h, *J* = 6.2 Hz, 1H), 3.06 – 2.93 (m, 2H), 1.81 – 1.60 (m, 6H), 1.53 – 1.44 (m, 2H), 1.19 (d, *J* = 6.2 Hz, 3H). ^{13}C NMR (100 MHz, Chloroform-*d*) δ 172.3, 129.4, 122.7, 67.6, 64.6, 38.1, 35.4, 25.0, 23.6, 17.9. HRMS (ESI, m/z): Calculated $C_{10}H_{18}O_3$ [M+Na]$^+$: 209.1148, found 209.1152.

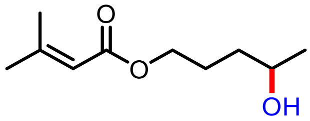

4-Hydroxypentyl 3-methylbut-2-enoate (38)

Compound **38** was prepared following the general procedure, purification by column chromatography on silica gel (petroleum ether/EtOAc = 5:1) yielded **38** (18.3 mg, 49%) as a yellow liquid. ^{1}H NMR (400 MHz, Chloroform-*d*) δ 5.67 – 5.63 (m, 1H), 4.09 (t, *J* = 6.6 Hz, 2H), 3.81 (h, *J* = 6.2 Hz, 1H), 2.14 (d, *J* = 1.5 Hz, 3H), 1.87 (d, *J* = 1.5 Hz, 3H), 1.80 – 1.64 (m, 3H), 1.54 – 1.46 (m, 2H), 1.18 (d, *J* = 6.2 Hz, 3H). ^{13}C NMR (100

MHz, Chloroform-*d*) δ 166.8, 156.7, 116.0, 67.6, 63.5, 35.6, 27.4, 25.1, 23.5, 20.2. HRMS (ESI, m/z): Calculated $C_{10}H_{18}O_3$ $[M+Na]^+$: 209.1148, found 209.1149.

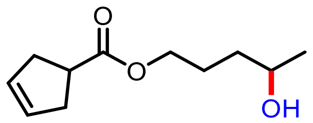

4-Hydroxypentyl cyclopent-3-ene-1-carboxylate (39)

Compound **39** was prepared following the general procedure, purification by column chromatography on silica gel (petroleum ether/EtOAc = 5:1) yielded **39** (19.8 mg, 50%) as a colorless liquid. ^{1}H NMR (400 MHz, Chloroform-*d*) δ 5.65 (s, 2H), 4.10 (t, J = 6.6 Hz, 2H), 3.82 (h, J = 6.2 Hz, 1H), 3.10 (p, J = 8.2 Hz, 1H), 2.63 (d, J = 8.2 Hz, 4H), 1.80 – 1.65 (m, 3H), 1.53 – 1.46 (m, 2H), 1.20 (d, J = 6.2 Hz, 3H). ^{13}C NMR (150 MHz, Chloroform-*d*) δ 176.3, 129.0, 67.6, 64.6, 41.6, 36.3, 35.5, 25.0, 23.6. HRMS (ESI, m/z): Calculated $C_{11}H_{18}O_3$ $[M+Na]^+$: 221.1148, found 221.1149.

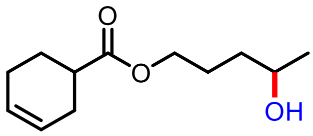

4-Hydroxypentyl cyclohex-3-ene-1-carboxylate (40)

Compound **40** was prepared following the general procedure, purification by column chromatography on silica gel (petroleum ether/EtOAc = 5:1) yielded **40** (27.9 mg, 66%) as a colorless liquid. ^{1}H NMR (400 MHz, Chloroform-*d*) δ 5.73 – 5.64 (m, 2H), 4.12 (t, J = 6.6 Hz, 2H), 3.83 (h, J = 6.2 Hz, 1H), 2.61 – 2.51 (m, 1H), 2.28 – 2.22 (m, 2H), 2.14 – 2.06 (m, 2H), 2.04 – 1.97 (m, 1H), 1.81 – 1.65 (m, 4H), 1.55 – 1.47 (m, 2H), 1.21 (d, J = 6.2 Hz, 3H). ^{13}C NMR (100 MHz, Chloroform-*d*) δ 176.0, 126.7, 125.2, 67.6, 64.3, 39.4, 35.5, 27.4, 25.1, 25.0, 24.4, 23.6. HRMS (ESI, m/z): Calculated $C_{12}H_{20}O_3$ $[M+Na]^+$: 235.1305, found 235.1303.

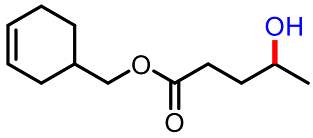

Cyclohex-3-en-1-ylmethyl 4-hydroxypentanoate (41)

Compound **41** was prepared following the general procedure, purification by column chromatography on silica gel (petroleum ether/EtOAc = 5:1) yielded **41** (26.3 mg, 62%)

as a yellow liquid.^{1}H NMR (400 MHz, Chloroform-*d*) δ 5.77 – 5.61 (m, 2H), 3.99 (d, *J* = 6.6 Hz, 2H), 3.90 – 3.77 (m, 1H), 2.46 (t, *J* = 7.2 Hz, 2H), 2.17 – 2.02 (m, 4H), 2.01 – 1.89 (m, 1H), 1.86 – 1.68 (m, 4H), 1.38 – 1.25 (m, 1H), 1.22 (d, *J* = 6.2 Hz, 3H). ^{13}C NMR (100 MHz, Chloroform-*d*) δ 174.3, 127.0, 125.5, 68.9, 67.3, 33.9, 33.0, 30.8, 28.1, 25.3, 24.4, 23.5. HRMS (ESI, m/z): Calculated $C_{12}H_{20}O_3$ $[M+Na]^+$: 235.1305, found 235.1303.

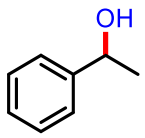

1-phenylethan-1-ol (42)

Compound **42** was prepared following the general procedure, purification by column chromatography on silica gel (petroleum ether/EtOAc = 5:1) yielded **42** (22.4 mg, 61%) as a colorless liquid. ^{1}H NMR (400 MHz, Chloroform-*d*) δ 7.43 – 7.36 (m, 4H), 7.34 – 7.28 (m, 1H), 4.91 (q, J = 6.5 Hz, 1H), 2.16 (s, 1H), 1.53 (d, J = 6.5 Hz, 3H). ^{13}C NMR (100 MHz, Chloroform-*d*) δ 145.9, 128.5, 127.5, 125.4, 70.4, 25.2. Spectroscopic data match those previously reported in the literature.[14]

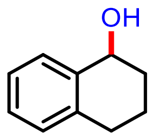

1,2,3,4-tetrahydronaphthalen-1-ol (43)

Compound **43** was prepared following the general procedure, purification by column chromatography on silica gel (petroleum ether/EtOAc = 5:1) yielded **43** (24.5 mg, 55%) as a yellow solid.^{1}H NMR (400 MHz, Chloroform-*d*) δ 7.51 – 7.35 (m, 1H), 7.28 – 7.14 (m, 2H), 7.14 – 7.03 (m, 1H), 4.75 (t, *J* = 4.6 Hz, 1H), 2.90 – 2.65 (m, 2H), 2.06 - 1.84 (m, 4H), 1.83 – 1.69 (m, 1H). ^{13}C NMR (100 MHz, Chloroform-*d*) δ 138.8, 137.1, 129.0, 128.7, 127.6, 126.2, 68.1, 32.3, 29.3, 18.8. Spectroscopic data match those previously reported in the literature.[14]

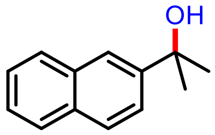

2-(naphthalen-2-yl) propan-2-ol (44)

Compound **44** was prepared following the general procedure, purification by column chromatography on silica gel (petroleum ether/EtOAc = 5:1) yielded **44** (26.1 mg, 70%) as a yellow solid.^{1}H NMR (400 MHz, Chloroform-*d*) δ 7.95 (d, *J* = 1.9 Hz, 1H), 7.85 – 7.81 (m, 2H), 7.62 (dd, *J* = 8.7, 2.0 Hz, 1H), 7.55 – 7.43 (m, 3H), 1.69 (s, 6H). ^{13}C NMR (100 MHz, Chloroform-*d*) δ 146.4, 133.2, 132.3, 128.2, 128.0, 127.7, 127.5, 126.1, 125.8, 123.6, 122.4, 72.8, 31.7. Spectroscopic data match those previously reported in the literature.[15]

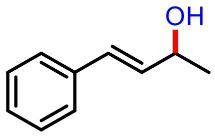

4-Phenylbut-3-en-2-ol (45)

Compound **45** was prepared following the general procedure, purification by column chromatography on silica gel (petroleum ether/EtOAc = 10:1) yielded **45** (15.1 mg, 51%) as a yellow liquid.^{1}H NMR (400 MHz, Chloroform-*d*) δ 7.45 – 7.39 (m, 2H), 7.38 – 7.32 (m, 2H), 7.31 – 7.25 (m, 1H), 6.60 (d, *J* = 16.0 Hz, 1H), 6.30 (dd, *J* = 16.0, 6.4 Hz, 1H), 4.60 – 4.42 (m, 1H), 1.78 (s, 1H), 1.41 (d, *J* = 6.4 Hz, 3H). ^{13}C NMR (100 MHz, Chloroform-*d*) δ 136.7, 133.6, 129.4, 128.6, 127.7, 126.5, 69.0, 23.4. Spectroscopic data match those previously reported in the literature.[18]

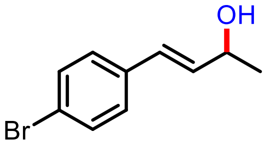

4-(4-bromophenyl) but-3-en-2-ol (46)

Compound **46** was prepared following the general procedure, purification by column chromatography on silica gel (petroleum ether/EtOAc = 5:1) yielded **46** (20.4 mg, 45%) as a yellow solid.^{1}H NMR (400 MHz, Chloroform-*d*) δ 7.49 – 7.44 (m, 2H), 7.30 – 7.25

(m, 2H), 6.53 (d, J = 16.0 Hz, 1H), 6.28 (dd, J = 16.0, 6.2 Hz, 1H), 4.56 – 4.46 (m, 1H), 1.40 (d, J = 6.4 Hz, 3H). ^{13}C NMR (150 MHz, Chloroform-*d*) δ 134.3, 131.7, 128.2, 128.0, 121.4, 68.8, 23.4. Spectroscopic data match those previously reported in the literature.[18]

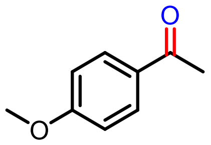

1-(4-methoxyphenyl)ethan-1-one (47)

Compound **47** was prepared following the general procedure, purification by column chromatography on silica gel (petroleum ether/EtOAc = 10:1) yielded **47** (8.4 mg, 28%) as a liquid. ^{1}H NMR (400 MHz, Chloroform-*d*) δ 7.94-7.83 (m, 2H), 7.00-6.80 (m, 2H), 3.82 (s, 3H), 2.51 (s, 3H). ^{13}C NMR (100 MHz, Chloroform-*d*) δ 196.7, 163.5, 130.5, 130.3, 113.7, 55.4, 26.3. [18]

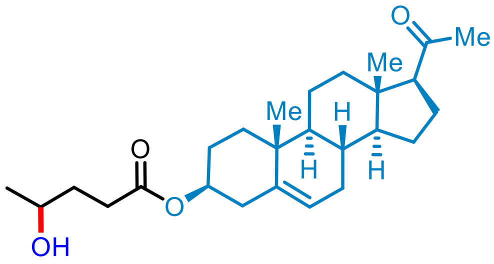

(3*S*,8*S*,9*S*,10*R*,13*S*,14*S*,17*S*)-17-acetyl-10,13-dimethyl-2,3,4,7,8,9,10,11,12,13,14,15,16,17-tetradecahydro-1H-cyclopenta[a]phenanthren-3-yl 4-hydroxypentanoate (48)

Compound **48** was prepared following the general procedure, purification by column chromatography on silica gel (petroleum ether/EtOAc = 5:1) yielded **48** (49.3 mg, 59%) as a white solid. ^{1}H NMR (400 MHz, Chloroform-*d*) δ 5.35 (d, J = 4.8 Hz, 1H), 4.68 – 4.53 (m, 1H), 3.88 – 3.77 (m, 1H), 2.52 (t, J = 8.9 Hz, 1H), 2.40 (t, J = 7.3 Hz, 2H), 2.34 – 2.27 (m, 2H), 2.21 – 2.08 (m, 4H), 2.06 – 1.93 (m, 3H), 1.88 – 1.81 (m, 2H), 1.80 – 1.50 (m, 8H), 1.49 – 1.38 (m, 3H), 1.24 – 1.10 (m, 6H), 1.00 (s, 3H), 0.61 (s, 3H). ^{13}C NMR (100 MHz, Chloroform-*d*) δ 209.6, 173.6, 139.6, 122.4, 74.0, 67.4, 63.7, 56.8, 49.9, 44.0, 38.8, 38.1, 38.0, 37.0, 36.6, 33.9, 31.8, 31.8, 31.6, 31.1, 27.7, 24.4,

23.5, 22.8, 21.0, 19.3, 13.2. HRMS (ESI, m/z): Calculated $C_{26}H_{40}O_4$ $[M+Na]^+$: 439.2819, found 439.2820.

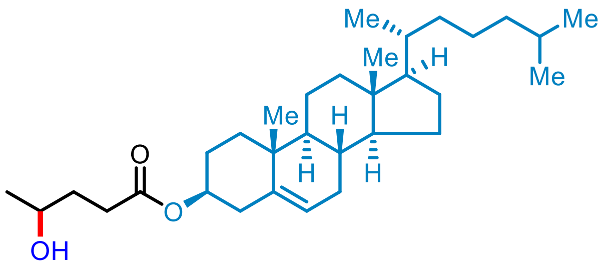

(3*S*,8*S*,9*S*,10*R*,13*R*,14*S*,17*R*)-10,13-dimethyl-17-((*R*)-6-methylheptan-2-yl)-2,3,4,7,8,9,10,11,12,13,14,15,16,17-tetradecahydro-1*H*-cyclopenta[a]phenanthren-3-yl 4-hydroxypentanoate (49)

Compound **49** was prepared following the general procedure, purification by column chromatography on silica gel (petroleum ether/EtOAc = 5:1) yielded **49** (71.3 mg, 73%) as a yellow liquid. ^{1}H NMR (400 MHz, Chloroform-*d*) δ 5.38 (d, *J* = 3.0 Hz, 1H), 4.70 – 4.56 (m, 1H), 3.90 – 3.80 (m, 1H), 2.43 (t, *J* = 7.3 Hz, 2H), 2.33 (d, *J* = 7.9 Hz, 2H), 2.06 – 1.95 (m, 3H), 1.91 – 1.71 (m, 5H), 1.63 – 1.43 (m, 7H), 1.40 – 1.31 (m, 3H), 1.29 – 1.19 (m, 5H), 1.19 – 1.08 (m, 6H), 1.03 (s, 6H), 0.93 (d, *J* = 6.6 Hz, 3H), 0.88 (dd, *J* = 6.6, 1.8 Hz, 6H), 0.69 (s, 3H). ^{13}C NMR (100 MHz, Chloroform-*d*) δ 173.6, 139.6, 122.7, 74.1, 67.4, 56.7, 56.2, 50.0, 42.3, 39.7, 39.5, 38.1, 38.1, 37.0, 36.6, 36.2, 35.8, 33.9, 31.9, 31.9, 31.2, 28.2, 28.0, 27.8, 24.3, 23.8, 23.5, 22.8, 22.6, 21.0, 19.3, 18.7, 11.8. HRMS (ESI, m/z): Calculated $C_{32}H_{54}O_3$ $[M+Na]^+$: 509.3965, found 509.3981.

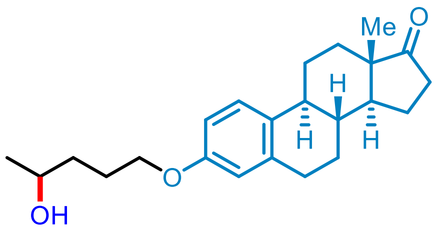

(8*R*,9*S*,13*S*,14*S*)-3-((4-hydroxypentyl)oxy)-13-methyl-6,7,8,9,11,12,13,14,15,16-decahydro-17*H*-cyclopenta[*a*]phenanthren-17-one (50)

Compound **50** was prepared following the general procedure, purification by column chromatography on silica gel (petroleum ether/EtOAc = 5:1) yielded **50** (37.1 mg, 52%) as a yellow liquid.^{1}H NMR (400 MHz, Chloroform-*d*) δ 7.19 (d, *J* = 8.6 Hz, 1H), 6.71

(dd, J = 8.6, 2.8 Hz, 1H), 6.64 (d, J = 2.8 Hz, 1H), 4.02 – 3.92 (m, 2H), 3.92 – 3.83 (m, 1H), 2.96 – 2.82 (m, 2H), 2.50 (dd, J = 18.8, 8.6 Hz, 1H), 2.27 – 1.82 (m, 9H), 1.74 – 1.51 (m, 6H), 1.48 – 1.37 (m, 2H), 1.22 (d, J = 6.2 Hz, 3H), 0.90 (s, 3H). ^{13}C NMR (100 MHz, Chloroform-*d*) δ 221.0, 156.9, 137.8, 132.1, 126.3, 114.6, 112.1, 68.0, 67.7, 50.4, 48.0, 44.0, 38.4, 36.0, 35.9, 31.6, 29.6, 26.6, 25.9, 25.8, 23.6, 21.6, 13.9. HRMS (ESI, m/z): Calculated $C_{23}H_{32}O_3$ $[M+H]^+$: 357.2424, found 357.2429.

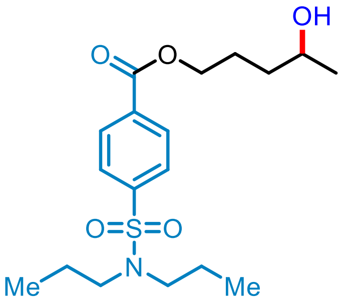

4-Hydroxypentyl 4-(*N*, *N*-dipropylsulfamoyl) benzoate (51)

Compound **51** was prepared following the general procedure, purification by column chromatography on silica gel (petroleum ether/EtOAc = 5:1) yielded **51** (37.9 mg, 51%) as a yellow liquid. ^{1}H NMR (600 MHz, Chloroform-*d*) δ 8.16 (d, J = 8.1 Hz, 2H), 7.88 (d, J = 8.1 Hz, 2H), 4.39 (t, J = 6.7 Hz, 2H), 3.89 (h, J = 6.2 Hz, 1H), 3.10 (t, J = 7.8 Hz, 4H), 1.98 – 1.90 (m, 1H), 1.89 – 1.80 (m, 2H), 1.63 – 1.58 (m, 2H), 1.58 – 1.51 (m, 4H), 1.24 (d, J = 6.2 Hz, 3H), 0.87 (t, J = 7.4 Hz, 6H). ^{13}C NMR (150 MHz, Chloroform-*d*) δ 165.3, 144.2, 133.6, 130.2, 127.0, 67.5, 65.7, 49.9, 35.4, 25.1, 23.7, 21.9, 11.1. HRMS (ESI, m/z): Calculated $C_{18}H_{29}NO_5S$ $[M+H]^+$: 372.1839, found 372.1844.

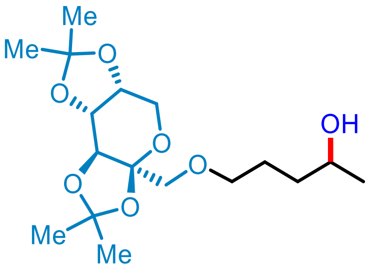

5-(((3a*S*,5a*R*,8a*R*,8b*S*)-2,2,7,7-tetramethyltetrahydro-3a*H*-bis([1,3]dioxolo)[4,5-*b*:4',5'-*d*]pyran-3a-yl)methoxy)pentan-2-ol (52)

Compound **52** was prepared following the general procedure, purification by column chromatography on silica gel (petroleum ether/EtOAc = 3:1) yielded **52** (63.2 mg, 91%)

as a yellow liquid. ^{1}H NMR (400 MHz, Chloroform-*d*) δ 4.58 (dd, *J* = 7.9, 2.6 Hz, 1H), 4.39 – 4.33 (m, 1H), 4.21 (d, *J* = 8.0 Hz, 1H), 3.88 (dd, *J* = 13.0, 2.0 Hz, 1H), 3.78 (q, *J* = 7.0 Hz, 1H), 3.70 (d, *J* = 13.0 Hz, 1H), 3.64 – 3.46 (m, 4H), 1.92 (s, 1H), 1.70 – 1.60 (m, 2H), 1.55 – 1.47 (m, 5H), 1.45 (s, 3H), 1.40 (s, 3H), 1.32 (s, 3H), 1.16 (d, *J* = 6.2 Hz, 3H). ^{13}C NMR (100 MHz, Chloroform-*d*) δ 108.9, 108.5, 108.5, 102.6, 102.6, 72.2, 72.1, 72.1, 72.0, 71.0, 71.0, 70.2, 70.1, 70.0, 67.7, 61.0, 36.2, 26.6, 26.6, 25.9, 25.9, 25.9, 25.3, 24.0, 23.5, 23.5. HRMS (ESI, m/z): Calculated $C_{17}H_{30}O_7$ $[M+Na]^+$: 369.1884, found 369.1888.

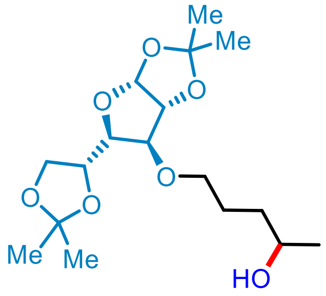

5-(((3a*R*,5*R*,6*S*,6a*R*)-5-((*R*)-2,2-dimethyl-1,3-dioxolan-4-yl)-2,2-dimethyltetrahydrofuro[2,3-*d*][1,3]dioxol-6-yl)oxy)pentan-2-ol (53)

Compound **53** was prepared following the general procedure, purification by column chromatography on silica gel (petroleum ether/EtOAc = 3:1) yielded **53** (56.1 mg, 81%) as a yellow liquid. ^{1}H NMR (400 MHz, Chloroform-*d*) δ 5.74 (d, *J* = 3.8 Hz, 1H), 4.63 (t, *J* = 4.2 Hz, 1H), 4.40 – 4.31 (m, 1H), 4.05 – 3.94 (m, 3H), 3.85 – 3.75 (m, 2H), 3.46 – 3.56 (m, 1H), 3.57 – 3.46 (m, 1H), 1.74 – 1.64 (m, 2H), 1.61 – 1.45 (m, 5H), 1.42 (d, *J* = 3.2 Hz, 3H), 1.33 (d, *J* = 9.4 Hz, 6H), 1.15 (d, *J* = 6.2 Hz, 3H). ^{13}C NMR (100 MHz, Chloroform-*d*) δ 112.9, 112.9, 109.7, 109.7, 103.9, 103.8, 78.8, 78.7, 77.8, 77.7, 77.6, 74.8, 74.7, 70.5, 70.4, 67.6, 67.4, 65.0, 65.0, 36.0, 35.6, 26.8, 26.6, 26.6, 26.4, 26.2, 26.1, 25.9, 25.1, 25.0, 23.6, 23.4. HRMS (ESI, m/z): Calculated $C_{17}H_{30}O_7$ $[M+Na]^+$: 369.1884, found 369.1888.

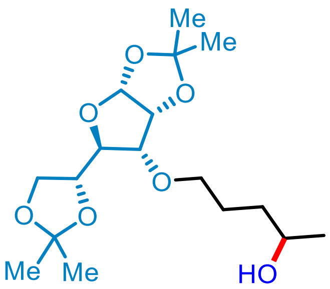

5-(((3a*R*,5*R*,6*R*,6a*R*)-5-((*R*)-2,2-dimethyl-1,3-dioxolan-4-yl)-2,2-dimethyltetrahydrofuro[2,3-*d*][1,3]dioxol-6-yl)oxy)pentan-2-ol (54)

Compound **54** was prepared following the general procedure, purification by column chromatography on silica gel (petroleum ether/EtOAc = 3:1) yielded **54** (44.0 mg, 65%) as a yellow liquid. ^{1}H NMR (600 MHz, Chloroform-*d*) δ 5.77 (d, *J* = 3.8 Hz, 1H), 4.65 (t, *J* = 4.2 Hz, 1H), 4.42 – 4.34 (m, 1H), 4.06 – 3.96 (m, 3H), 3.88 – 3.79 (m, 2H), 3.76 – 3.69 (m, 1H), 3.58 – 3.51 (m, 1H), 1.75 – 1.67 (m, 2H), 1.62 – 1.47 (m, 5H), 1.44 (d, *J* = 4.8 Hz, 3H), 1.35 (d, *J* = 14.1 Hz, 6H), 1.17 (d, *J* = 6.2 Hz, 3H). ^{13}C NMR (150 MHz, Chloroform-*d*) δ 112.9, 112.9, 109.8, 109.8, 103.9, 103.9, 78.8, 78.7, 77.8, 77.7, 74.8, 74.7, 70.5, 70.4, 67.6, 67.5, 65.1, 65.0, 36.1, 35.6, 26.8, 26.6, 26.6, 26.4, 26.2, 26.2, 25.9, 25.1, 25.0, 23.6, 23.4. HRMS (ESI, m/z): Calculated $C_{17}H_{30}O_7$ $[M+Na]^+$: 369.1884, found 369.1887.

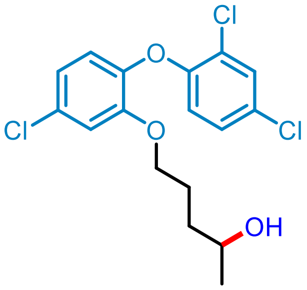

5-(5-chloro-2-(2,4-dichlorophenoxy) phenoxy) pentan-2-ol (55)

Compound **55** was prepared following the general procedure, purification by column chromatography on silica gel (petroleum ether/EtOAc = 8:1) yielded **55** (66.9 mg, 89%) as a yellow solid. M.p.: 76 – 79 °C. ^{1}H NMR (400 MHz, Chloroform-*d*) δ 7.42 (d, *J* = 2.5 Hz, 1H), 7.08 (dd, *J* = 8.8, 2.5 Hz, 1H), 6.98 – 6.90 (m, 3H), 6.63 (d, *J* = 8.8 Hz, 1H), 4.01 – 3.87 (m, 2H), 3.78 – 3.66 (m, 1H), 1.86 – 1.60 (m, 2H), 1.40 – 1.26 (m, 2H), 1.12 (d, *J* = 6.2 Hz, 3H). ^{13}C NMR (100 MHz, Chloroform-*d*) δ 152.6, 150.9, 142.9,

130.7, 130.1, 127.7, 127.6, 124.3, 122.2, 121.0, 117.7, 114.7, 69.0, 67.5, 35.4, 25.3, 23.6. HRMS (ESI, m/z): Calculated $C_{17}H_{17}Cl_3O_3$ $[M+Na]^+$: 397.0135, found 397.0127.

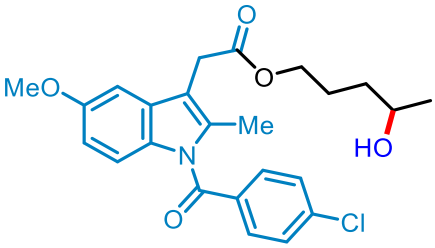

4-Hydroxypentyl-2-(1-(4-chlorobenzoyl)-5-methoxy-2-methyl-1H-indol-3-yl) acetate (56)

Compound **56** was prepared following the general procedure, purification by column chromatography on silica gel (petroleum ether/EtOAc = 5:1) yielded **56** (57.7 mg, 65%) as a yellow liquid. ^{1}H NMR (400 MHz, Chloroform-*d*) δ 7.67 (d, *J* = 8.5 Hz, 2H), 7.51 – 7.44 (m, 2H), 6.99 (d, *J* = 2.5 Hz, 1H), 6.89 (d, *J* = 9.0 Hz, 1H), 6.68 (dd, *J* = 9.0, 2.5 Hz, 1H), 4.13 (t, *J* = 6.5 Hz, 2H), 3.84 (s, 3H), 3.74 (q, *J* = 6.2 Hz, 1H), 3.67 (s, 2H), 2.40 (s, 3H), 1.82 – 1.62 (m, 3H), 1.47 – 1.38 (m, 2H), 1.14 (d, *J* = 6.2 Hz, 3H). ^{13}C NMR (100 MHz, Chloroform-*d*) δ 170.9, 168.3, 156.0, 139.3, 135.9, 133.9, 131.2, 130.9, 130.7, 129.1, 114.9, 112.7, 111.5, 101.6, 67.4, 65.0, 55.7, 35.4, 30.5, 25.0, 23.5, 13.4. HRMS (ESI, m/z): Calculated $C_{24}H_{26}ClNO_5$ $[M+H]^+$: 444.1572, found 444.1577.

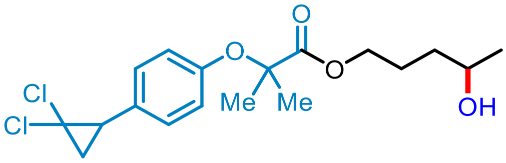

4-hydroxypentyl 2-(4-(2,2-dichlorocyclopropyl) phenoxy)-2-methylpropanoate (57)

Compound **57** was prepared following the general procedure, purification by column chromatography on silica gel (petroleum ether/EtOAc = 5:1) yielded **57** (36.0 mg, 48%) as a yellow liquid. ^{1}H NMR (400 MHz, Chloroform-*d*) δ 7.09 (d, *J* = 8.6 Hz, 2H), 6.79 (d, *J* = 8.6 Hz, 2H), 4.21 – 4.10 (m, 2H), 3.71 (h, *J* = 6.2 Hz, 1H), 2.81 (dd, *J* = 10.7, 8.4 Hz, 1H), 1.92 (dd, *J* = 10.7, 7.4 Hz, 1H), 1.79 – 1.62 (m, 4H), 1.59 (s, 6H), 1.39 – 1.31 (m, 2H), 1.11 (d, *J* = 6.2 Hz, 3H). ^{13}C NMR (100 MHz, Chloroform-*d*) δ 174.3, 155.0, 129.7, 128.1, 118.4, 67.4, 65.5, 60.9, 35.3, 34.8, 25.8, 25.4, 24.8, 23.6. HRMS

(ESI, m/z): Calculated $C_{18}H_{24}Cl_2O_4$ $[M+Na]^+$: 397.0944, found 397.0949.

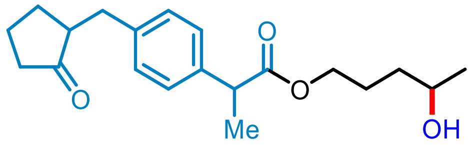

4-Hydroxypentyl 2-(4-((2-oxocyclopentyl) methyl) phenyl) propanoate (58)

Compound **58** was prepared following the general procedure, purification by column chromatography on silica gel (petroleum ether/EtOAc = 5:1) yielded **58** (41.7 mg, 63 %) as a yellow liquid. ^{1}H NMR (400 MHz, Chloroform-*d*) δ 7.19 (d, *J* = 8.2 Hz, 2H), 7.10 (d, *J* = 8.2 Hz, 2H), 4.13 – 4.00 (m, 2H), 3.76 – 3.62 (m, 2H), 3.09 (dd, *J* = 13.9, 4.2 Hz, 1H), 2.49 (dd, *J* = 13.9, 9.5 Hz, 1H), 2.37 – 2.26 (m, 2H), 2.14 – 2.02 (m, 2H), 1.99 – 1.89 (m, 1H), 1.78 – 1.51 (m, 5H), 1.46 (d, *J* = 7.2 Hz, 3H), 1.40 – 1.32 (m, 2H), 1.12 (d, *J* = 6.2 Hz, 3H). ^{13}C NMR (100 MHz, Chloroform-*d*) δ 220.2, 174.7, 138.8, 138.4, 129.1, 127.5, 67.4, 64.7, 64.7, 50.9, 45.2, 38.2, 35.3, 35.3, 35.2, 29.2, 24.9, 23.5, 20.5, 18.4. HRMS (ESI, m/z): Calculated $C_{20}H_{28}O_4$ $[M+Na]^+$: 355.1880, found 355.1877.

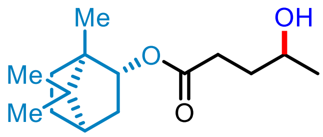

(1*R*,2*R*,4*R*)-1,7,7-trimethyl bicyclo[2.2.1]heptan-2-yl 4-hydroxypentanoate (59)

Compound **59** was prepared following the general procedure, purification by column chromatography on silica gel (petroleum ether/EtOAc = 5:1) yielded **59** (40.6 mg, 80%) as colorless liquid. ^{1}H NMR (400 MHz, Chloroform-*d*) δ 4.70 – 4.63 (m, 1H), 3.88 – 3.78 (m, 1H), 2.46 – 2.37 (m, 2H), 1.87 – 1.49 (m, 8H), 1.21 (d, *J* = 6.2 Hz, 3H), 1.18 – 1.04 (m, 2H), 0.98 (s, 3H), 0.84 (s, 6H). ^{13}C NMR (100 MHz, Chloroform-*d*) δ 173.6, 81.1, 81.1, 67.3, 48.7, 48.6, 46.9, 45.0, 38.8, 33.9, 33.7, 31.2, 31.2, 27.0, 23.5, 23.5, 20.1, 19.9, 11.4. HRMS (ESI, m/z): Calculated $C_{15}H_{26}O_3$ $[M+Na]^+$: 277.1774, found 277.1770.

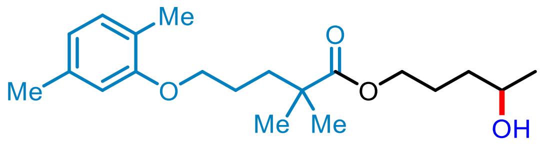

4-Hydroxypentyl 5-(2,5-dimethylphenoxy)-2,2-dimethylpentanoate (60)

Compound **60** was prepared following the general procedure, purification by column chromatography on silica gel (petroleum ether/EtOAc = 5:1) yielded **60** (43.7 mg, 65%) as a yellow liquid. ^{1}H NMR (400 MHz, Chloroform-*d*) δ 7.00 (d, *J* = 7.5 Hz, 1H), 6.66 (d, *J* = 7.5 Hz, 1H), 6.61 (s, 1H), 4.09 (t, *J* = 6.6 Hz, 2H), 3.95 – 3.89 (m, 2H), 3.86 – 3.77 (m, 1H), 2.31 (s, 3H), 2.18 (s, 3H), 1.80 – 1.64 (m, 7H), 1.55 – 1.45 (m, 2H), 1.25 - 1.16 (m, 9H). ^{13}C NMR (100 MHz, Chloroform-*d*) δ 177.9, 156.9, 136.5, 130.3, 123.6, 120.7, 112.0, 68.0, 67.6, 64.4, 42.1, 37.1, 35.5, 25.2, 25.0, 23.6, 21.4, 15.8. HRMS (ESI, m/z): Calculated $C_{20}H_{32}O_4$ [M+Na]$^+$: 359.2193, found 359.2188.

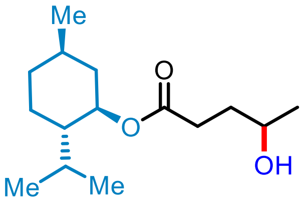

(1*R*,2*S*,5*R*)-2-isopropyl-5-methylcyclohexyl 4-hydroxypentanoate (61)

Compound **61** was prepared following the general procedure, purification by column chromatography on silica gel (petroleum ether/EtOAc = 5:1) yielded **61** (41.5 mg, 81%) as a colorless liquid. ^{1}H NMR (400 MHz, Chloroform-*d*) δ 4.67 (td, *J* = 10.9, 4.4 Hz, 1H), 3.90 – 3.77 (m, 1H), 2.41 (td, *J* = 7.3, 2.3 Hz, 2H), 2.01 – 1.93 (m, 1H), 1.90 – 1.71 (m, 4H), 1.71 – 1.61 (m, 2H), 1.53 – 1.42 (m, 1H), 1.40 – 1.32 (m, 1H), 1.20 (d, *J* = 6.2 Hz, 3H), 1.10 – 0.92 (m, 2H), 0.88 (dd, *J* = 6.8, 3.6 Hz, 6H), 0.74 (dd, *J* = 7.0, 1.4 Hz, 3H). ^{13}C NMR (100 MHz, Chloroform-*d*) δ 173.7, 67.5, 67.4, 47.0, 47.0, 40.9, 40.9, 34.2, 34.0, 31.4, 31.2, 26.3, 26.3, 23.5, 23.4, 23.4, 22.0, 20.8, 16.3, 16.3. HRMS (ESI, m/z): Calculated $C_{15}H_{28}O_3$ [M+Na]$^+$: 279.1931, found 279.1930.

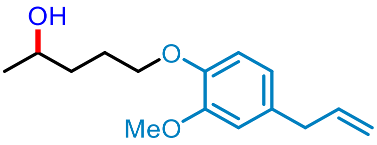

62

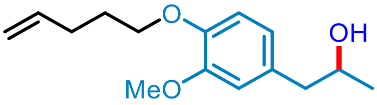

62' (10%)

Compound **62** was prepared following the general procedure, purification by column chromatography on silica gel (petroleum ether/EtOAc = 3:1) yielded **62** (30.5 mg, 61%) as a yellow liquid. ^{1}H NMR (400 MHz, Chloroform-*d*) δ 6.82 (d, *J* = 8.4 Hz, 1H), 6.75 – 6.68 (m, 2H), 5.93 – 5.77 (m, 1H), 5.11 – 4.93 (m, 2H), 4.04 – 3.93 (m, 3H), 3.85 (s,

3H), 2.73 (dd, J = 13.6, 4.5 Hz, 1H), 2.60 (dd, J = 13.6, 8.2 Hz, 1H), 2.29 – 2.18 (m, 2H), 1.98 – 1.88 (m, 2H), 1.24 (d, J = 6.2 Hz, 3H). ^{13}C NMR (100 MHz, Chloroform-*d*) δ 149.5, 147.2, 137.9, 131.2, 121.4, 115.1, 113.4, 113.1, 68.9, 68.4, 56.0, 45.4, 30.1, 28.4, 22.7. ^{1}H NMR of **61'** (600 MHz, Chloroform-*d*) δ 6.80 (d, J = 8.4 Hz, 1H), 6.71 – 6.66 (m, 2H), 6.01 – 5.89 (m, 1H), 5.15 – 5.01 (m, 2H), 4.02 (q, J = 6.5 Hz, 2H), 3.92 – 3.80 (m, 4H), 3.33 (d, J = 6.7 Hz, 2H), 1.98 – 1.90 (m, 2H), 1.71 – 1.50 (m, 2H), 1.22 (d, J = 6.2 Hz, 3H). HRMS (ESI, m/z): Calculated $C_{15}H_{22}O_3$ [M+Na]$^+$: 273.1461, found 273.1466.

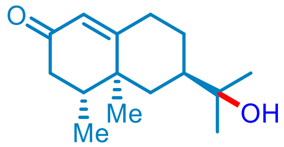

(4*R*,4a*S*,6*R*)-6-(2-hydroxypropan-2-yl)-4,4a-dimethyl-4,4a,5,6,7,8-hexahydronaphthalen-2(3*H*)-one (63)

Compound **63** was prepared following the general procedure, purification by column chromatography on silica gel (petroleum ether/EtOAc = 3:1) yielded **63** (18.9 mg, 40%) as a yellow liquid. ^{1}H NMR (400 MHz, Chloroform-*d*) δ 5.75 (s, 1H), 2.52 – 2.17 (m, 5H), 2.07 – 1.95 (m, 3H), 1.72 (tt, J = 12.5, 3.0 Hz, 1H), 1.27 – 1.14 (m, 8H), 1.07 (s, 3H), 0.96 (d, J = 6.8 Hz, 3H). ^{13}C NMR (100 MHz, Chloroform-*d*) δ 199.7, 170.8, 124.4, 72.4, 43.9, 42.1, 40.6, 39.6, 39.2, 33.0, 27.7, 27.3, 26.9, 16.9, 15.0. HRMS (ESI, m/z): Calculated $C_{15}H_{24}O_2$ [M+H]$^+$: 237.1849, found 237.1852.

Copies of ^{1}H, ^{13}C and ^{19}F NMR Spectra for Compounds

^{1}H NMR spectrum of **1** (**400 MHz, Chloroform-*d***)

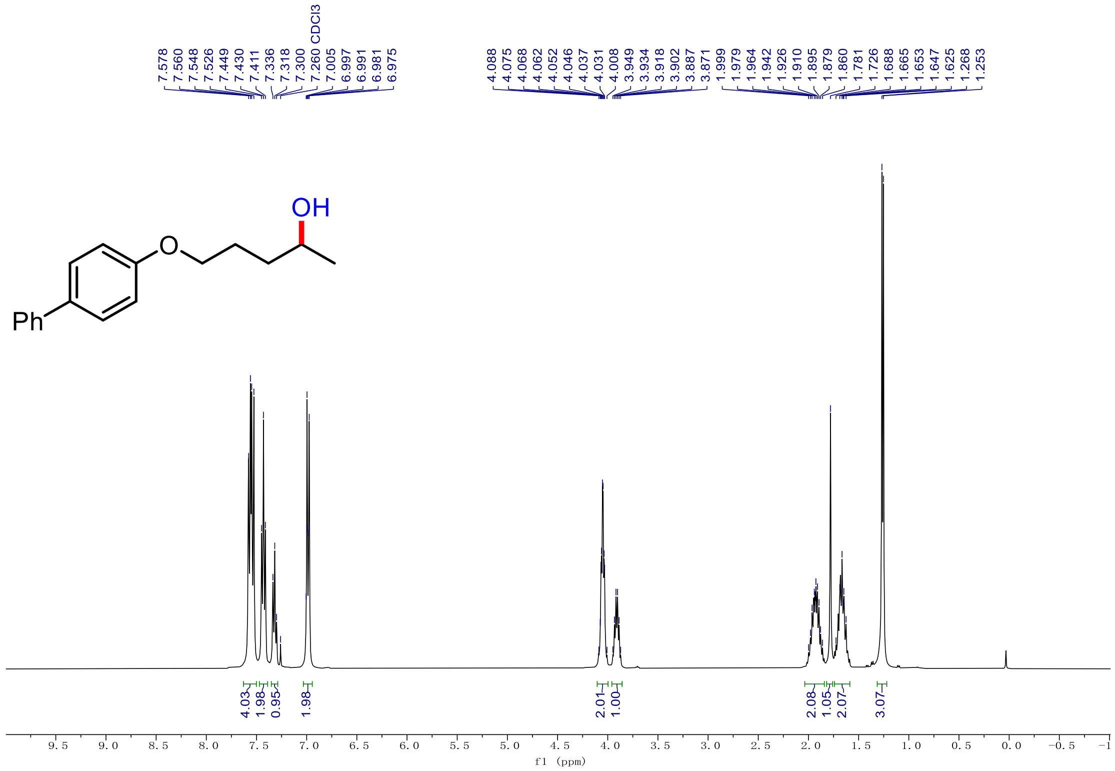

^{13}C NMR spectrum of **1** (**150 MHz, Chloroform-*d***)

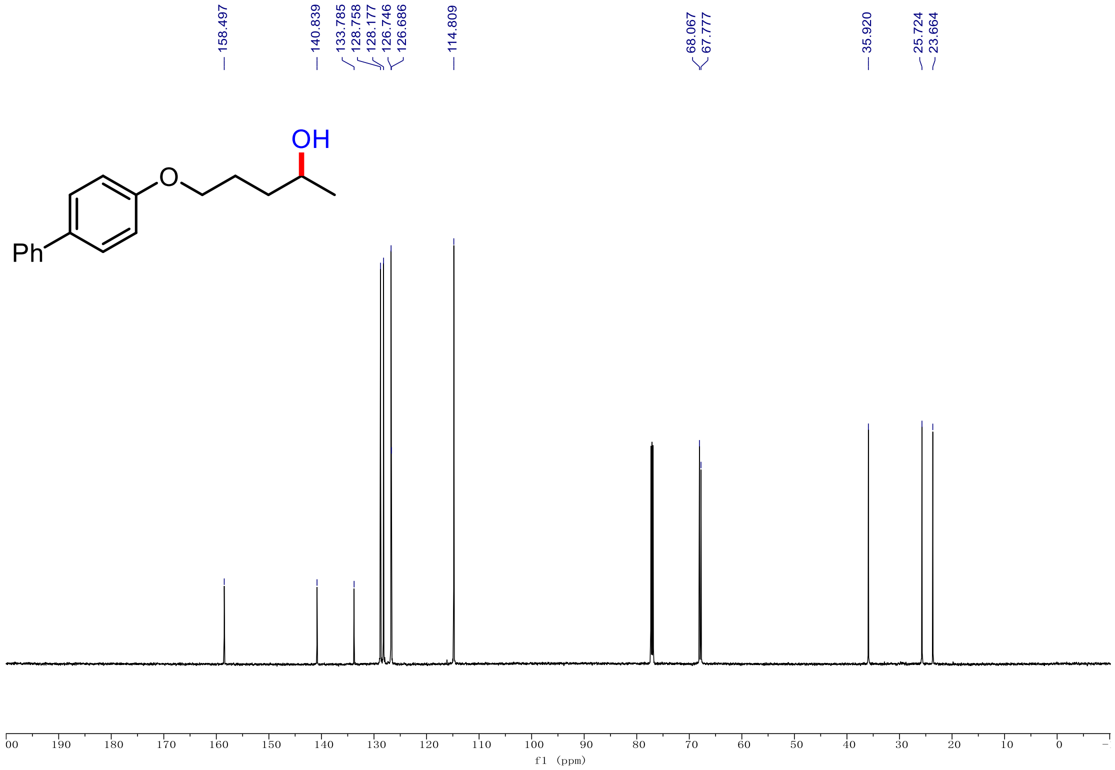

^{1}H NMR spectrum of **2** (**400 MHz, Chloroform-*d***)

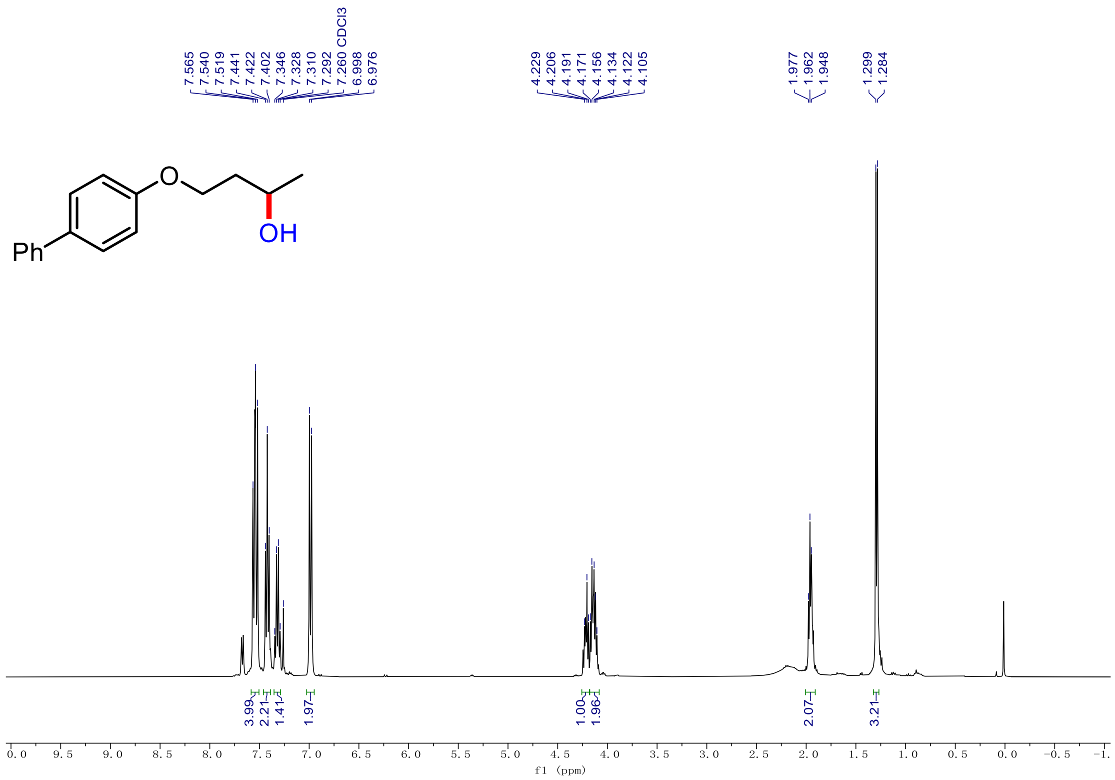

^{13}C NMR spectrum of **2** (**100 MHz, Chloroform-*d***)

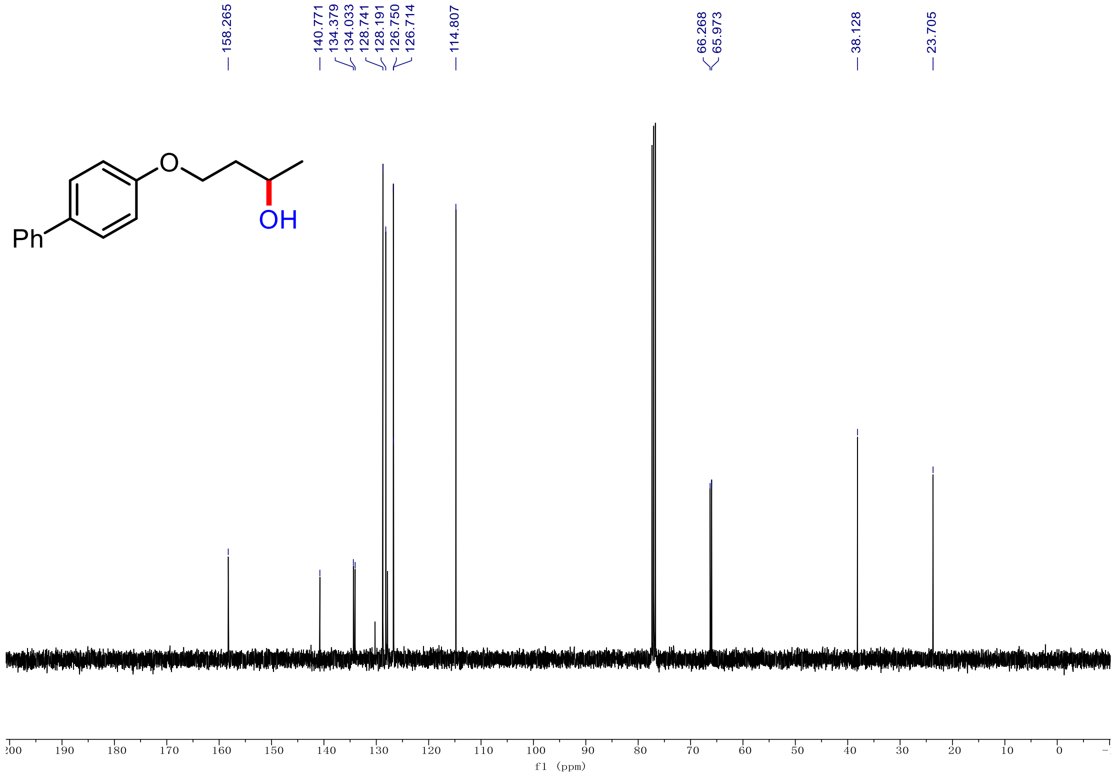

^{1}H NMR spectrum of **3** (**400 MHz, Chloroform-*d***)

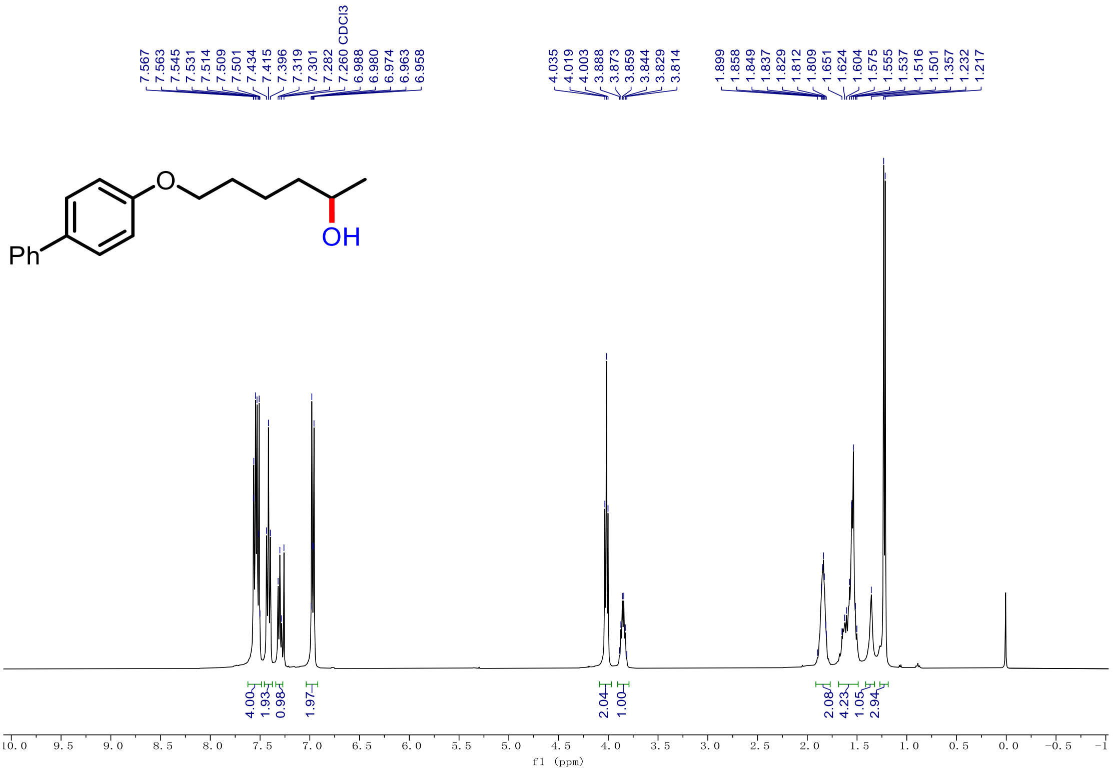

^{13}C NMR spectrum of **3** (**100 MHz, Chloroform-*d***)

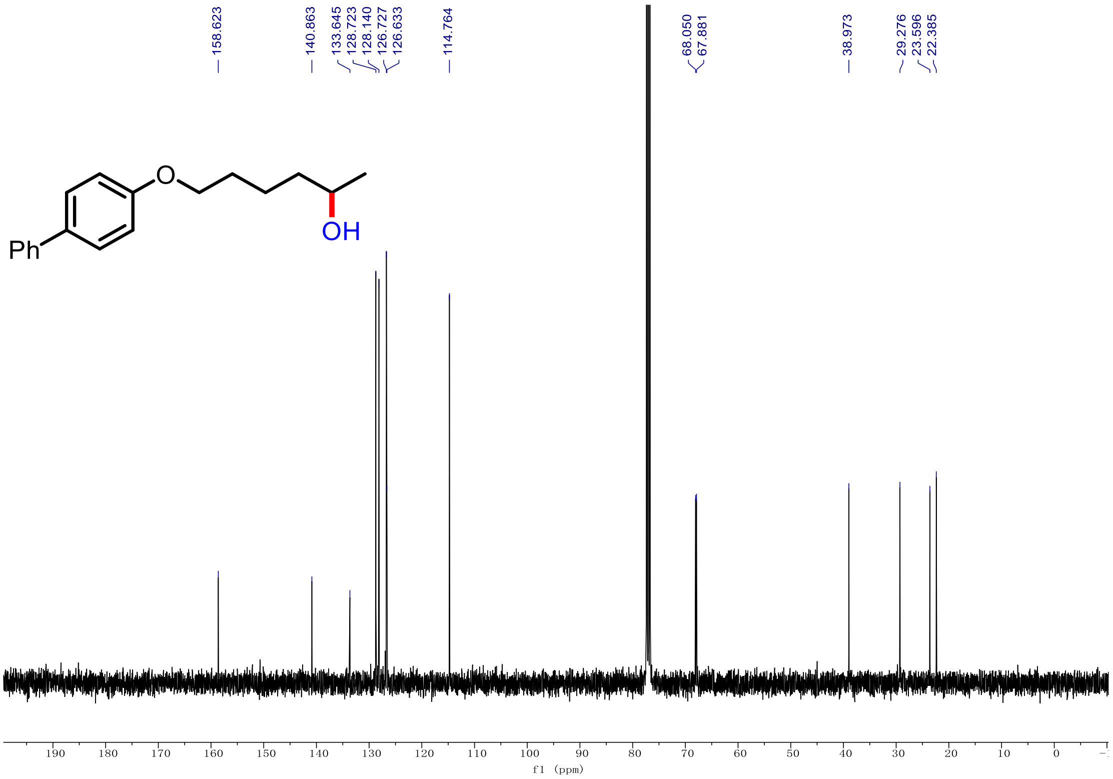

^{1}H NMR spectrum of **4** (**400 MHz, Chloroform-*d***)

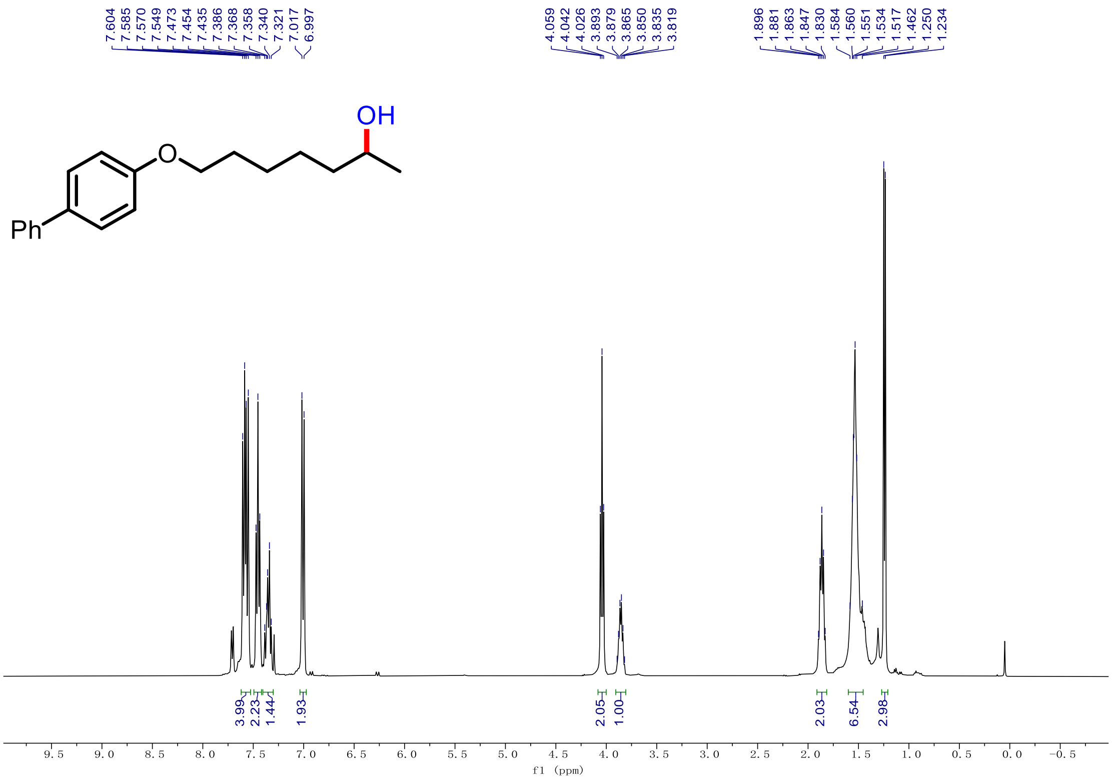

^{13}C NMR spectrum of **4** (**100 MHz, Chloroform-*d***)

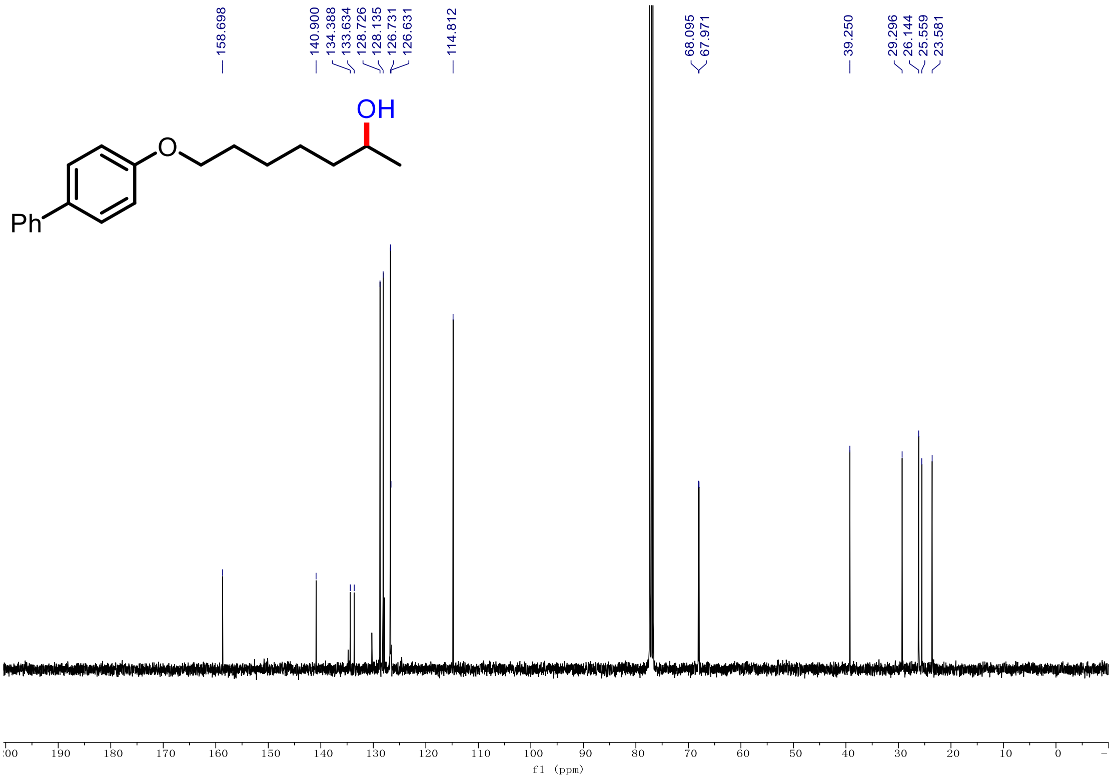

^{1}H NMR spectrum of **5** (**400 MHz, Chloroform-*d***)

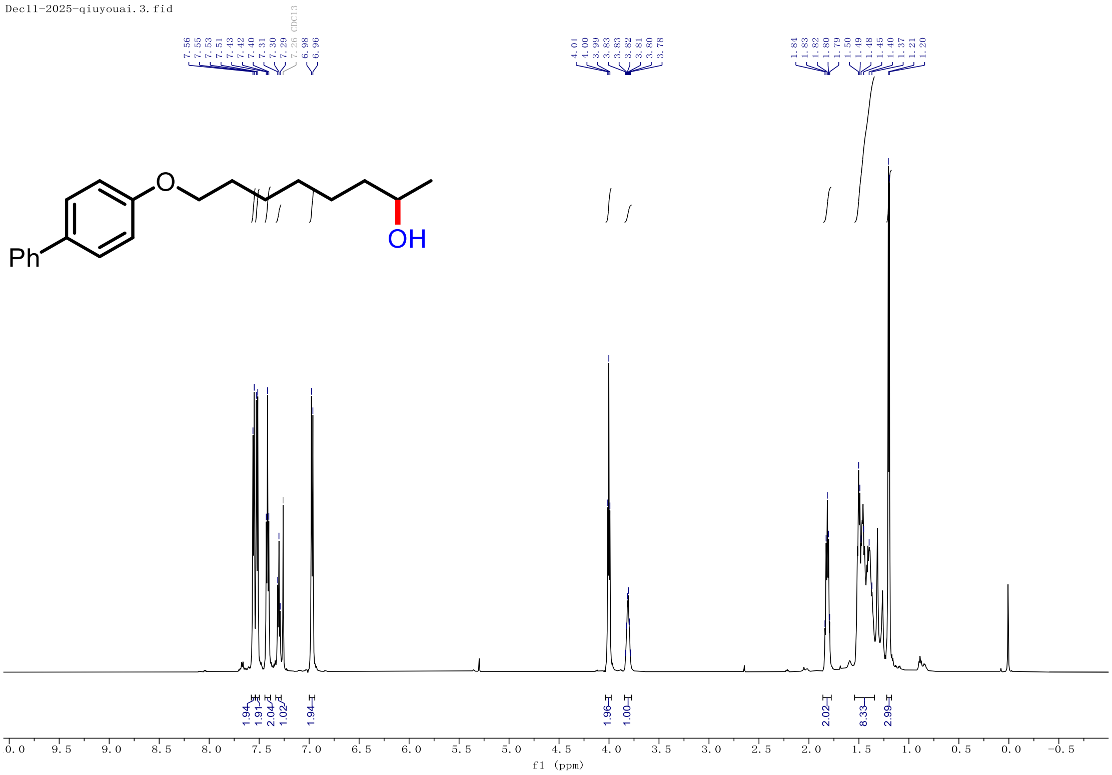

^{13}C NMR spectrum of **5** (**100 MHz, Chloroform-*d***)

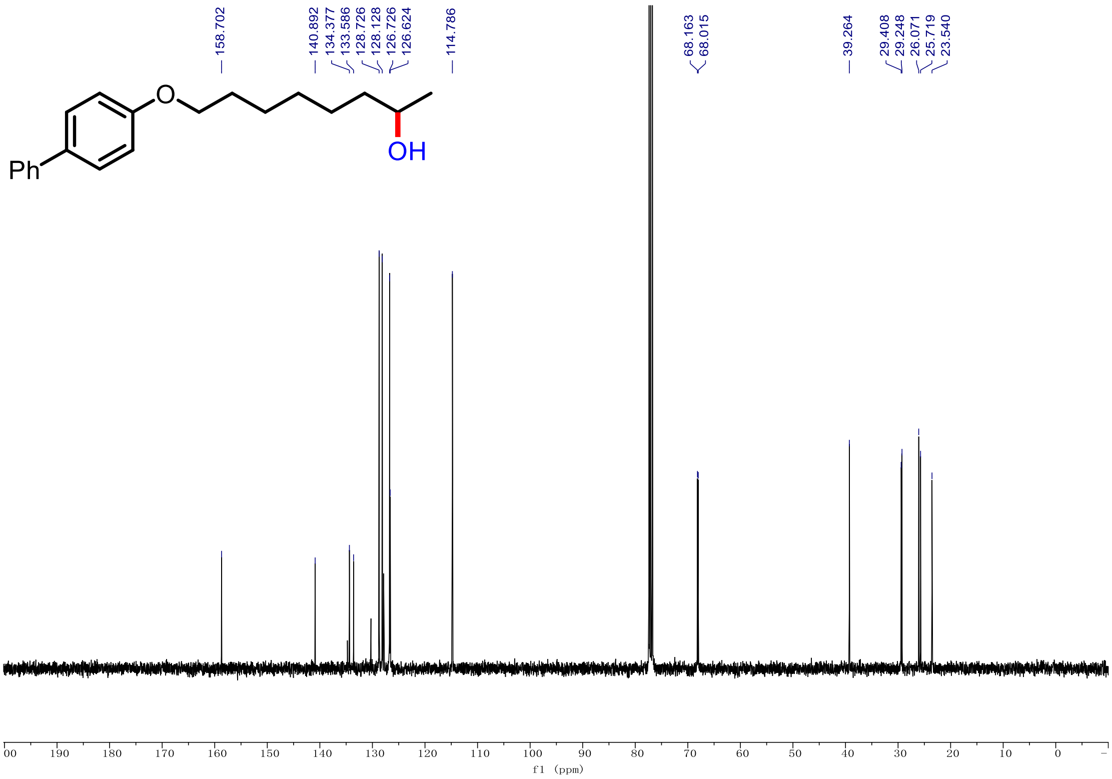

^{1}H NMR spectrum of **6** (**400 MHz, Chloroform-*d***)

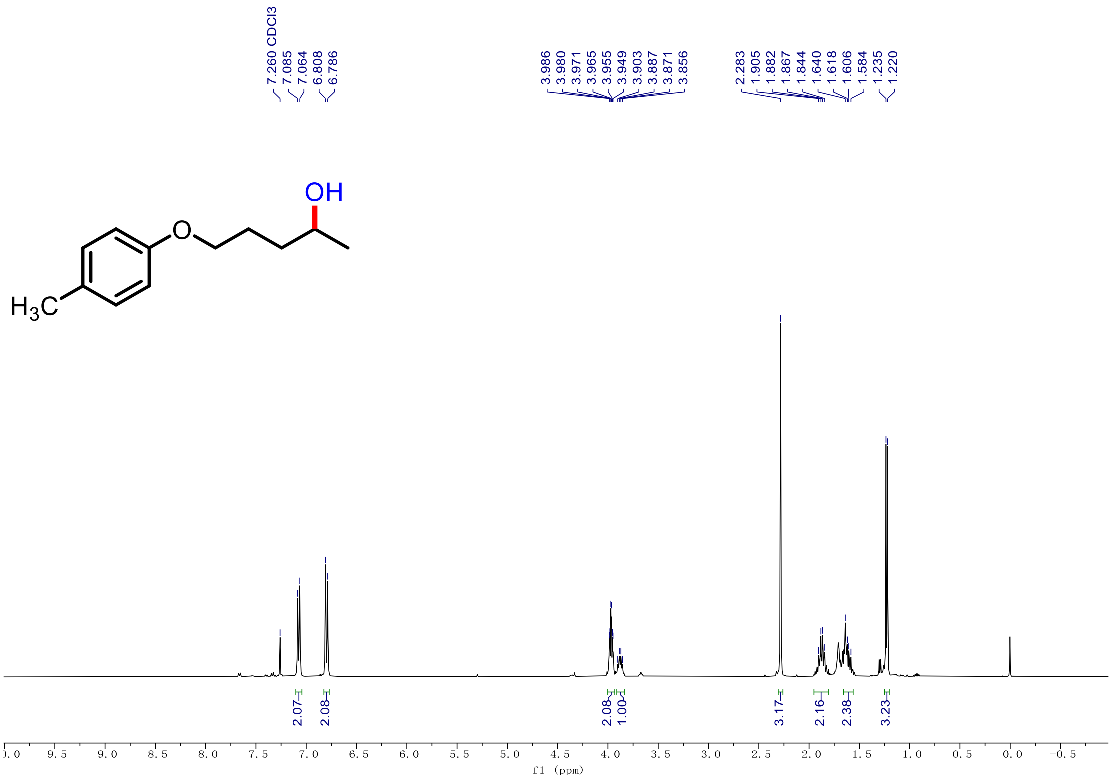

^{13}C NMR spectrum of **6** (**100 MHz, Chloroform-*d***)

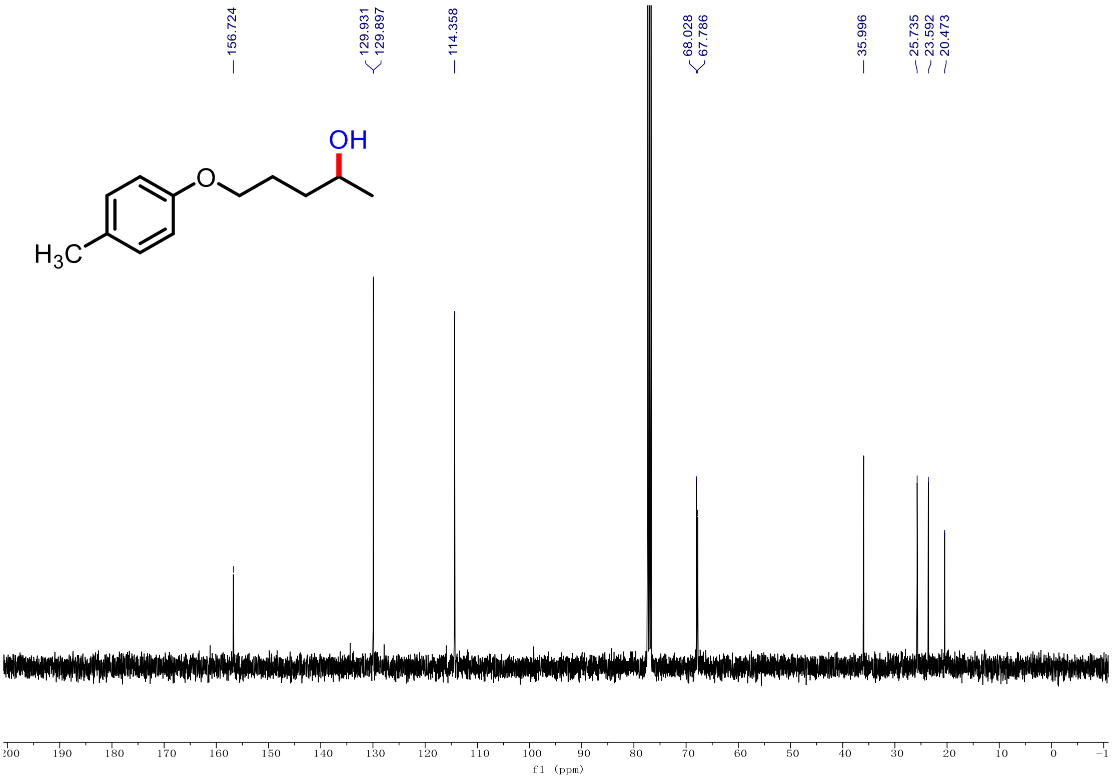

^{1}H NMR spectrum of **7** (**400 MHz, Chloroform-*d***)

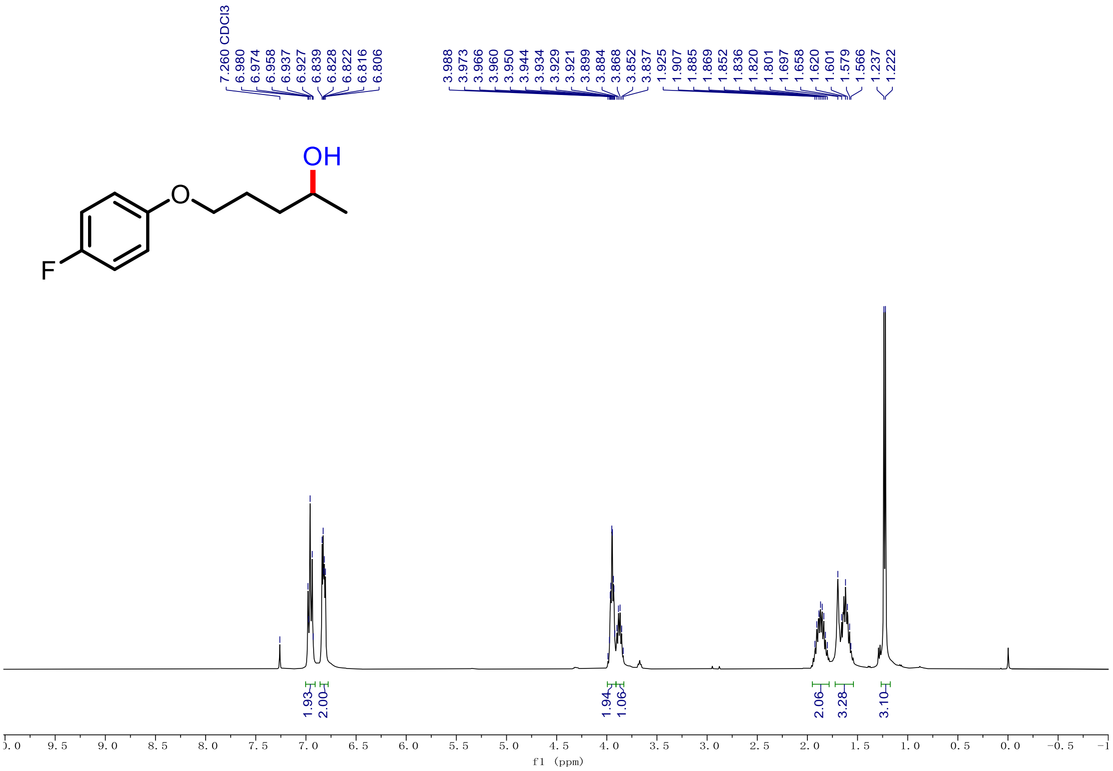

^{13}C NMR spectrum of **7** (**100 MHz, Chloroform-*d***)

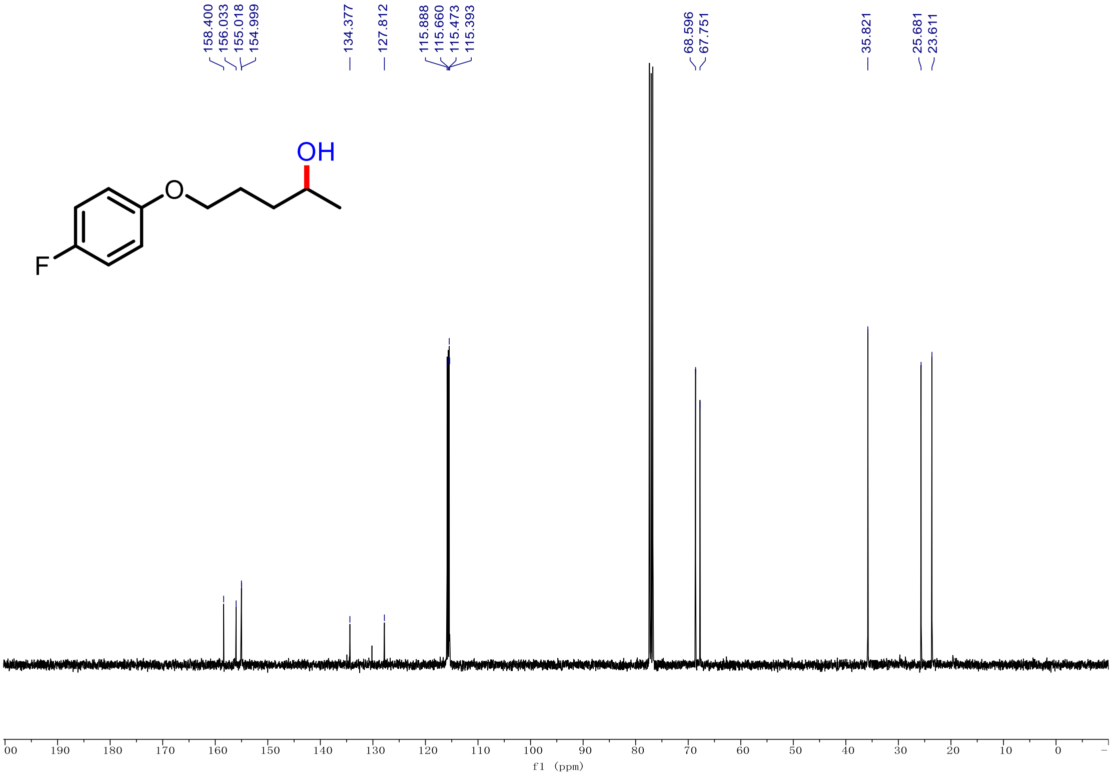

^{19}F NMR spectrum of **7** (**376 MHz, Chloroform-*d***)

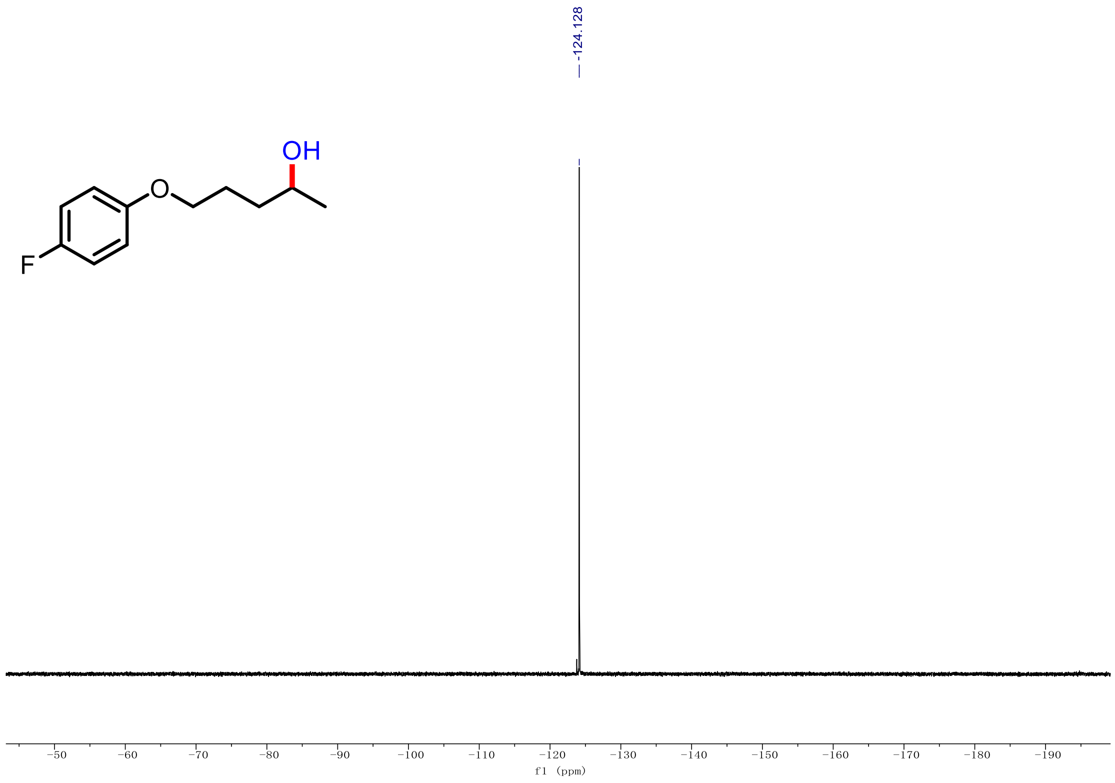

^{1}H NMR spectrum of **8** (**400 MHz, Chloroform-*d***)

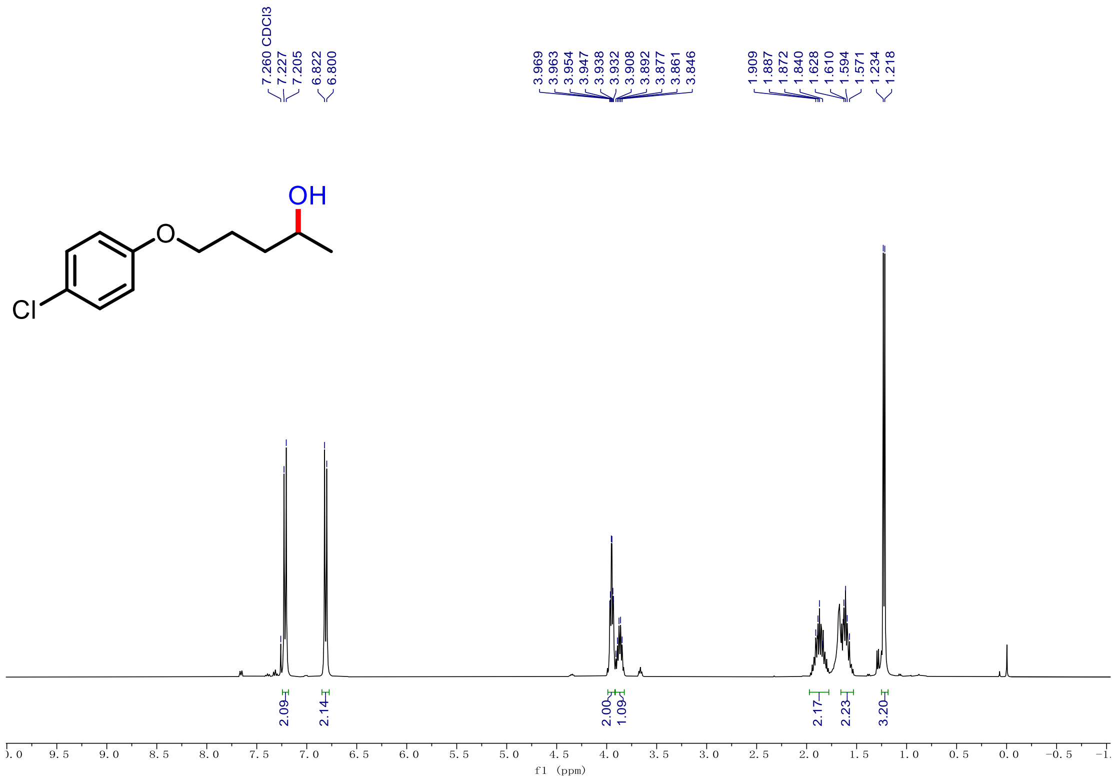

^{13}C NMR spectrum of **8** (**100 MHz, Chloroform-*d***)

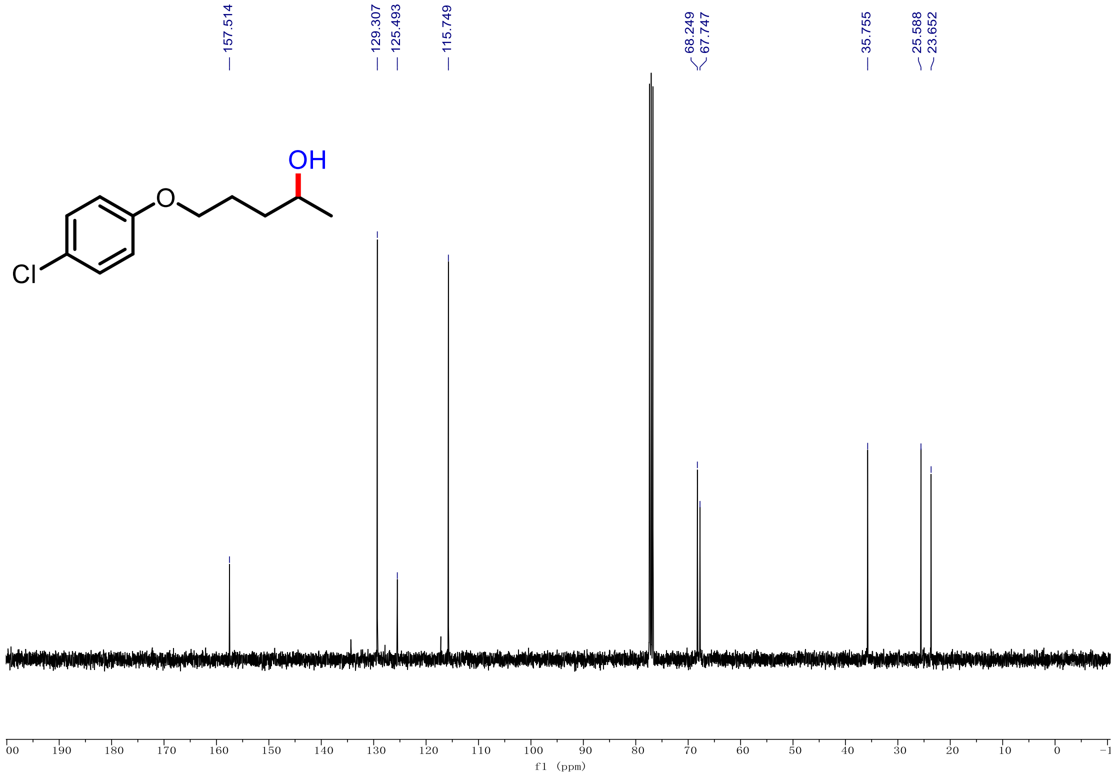

^{1}H NMR spectrum of **9** (**400 MHz, Chloroform-*d***)

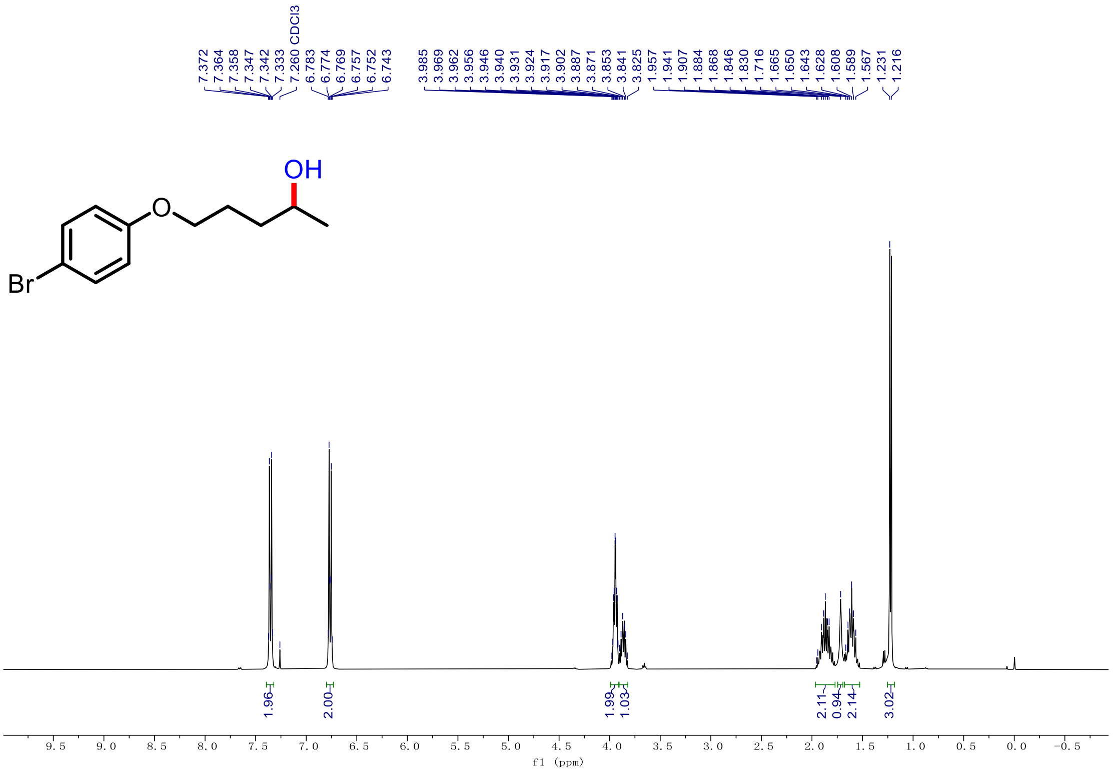

^{13}C NMR spectrum of **9** (**100 MHz, Chloroform-*d***)

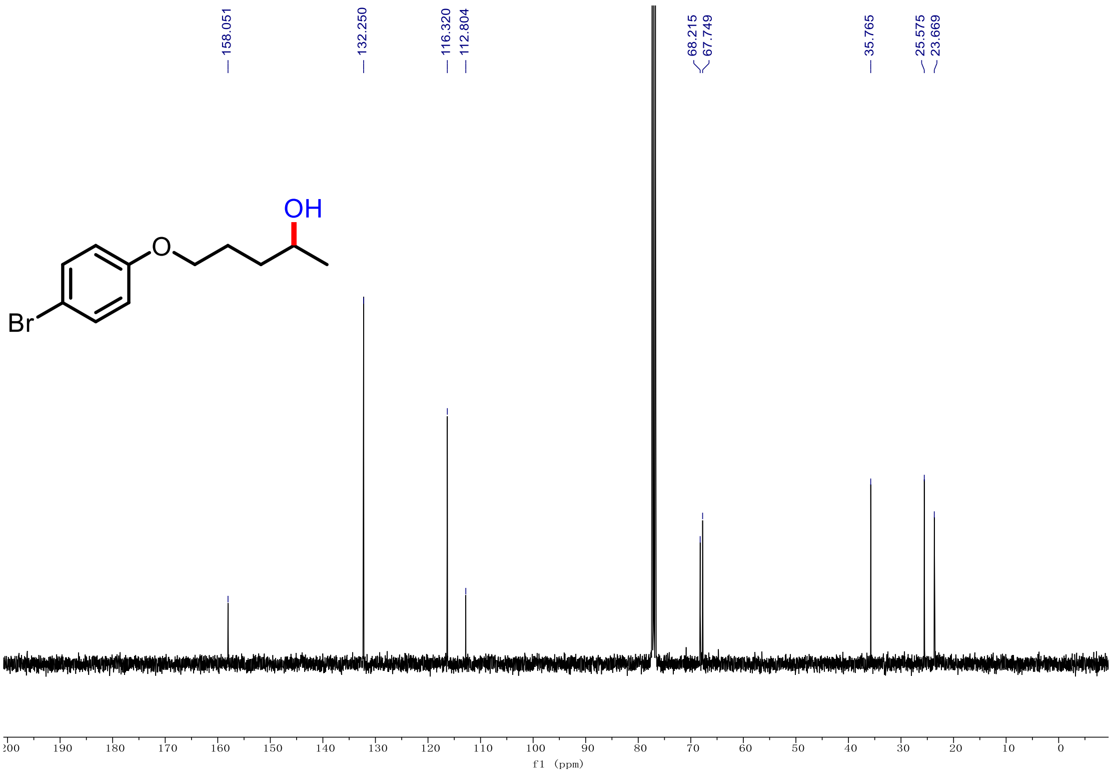

^{1}H NMR spectrum of **10** (**400 MHz, Chloroform-*d***)

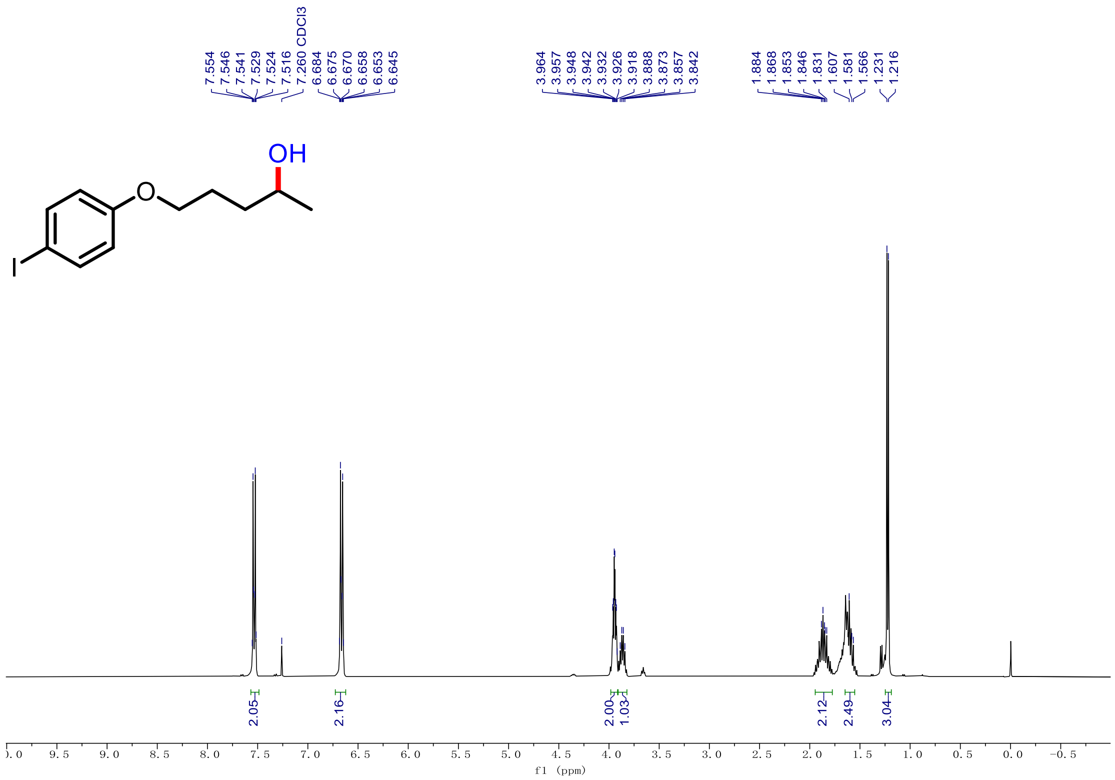

^{13}C NMR spectrum of **10** (**100 MHz, Chloroform-*d***)

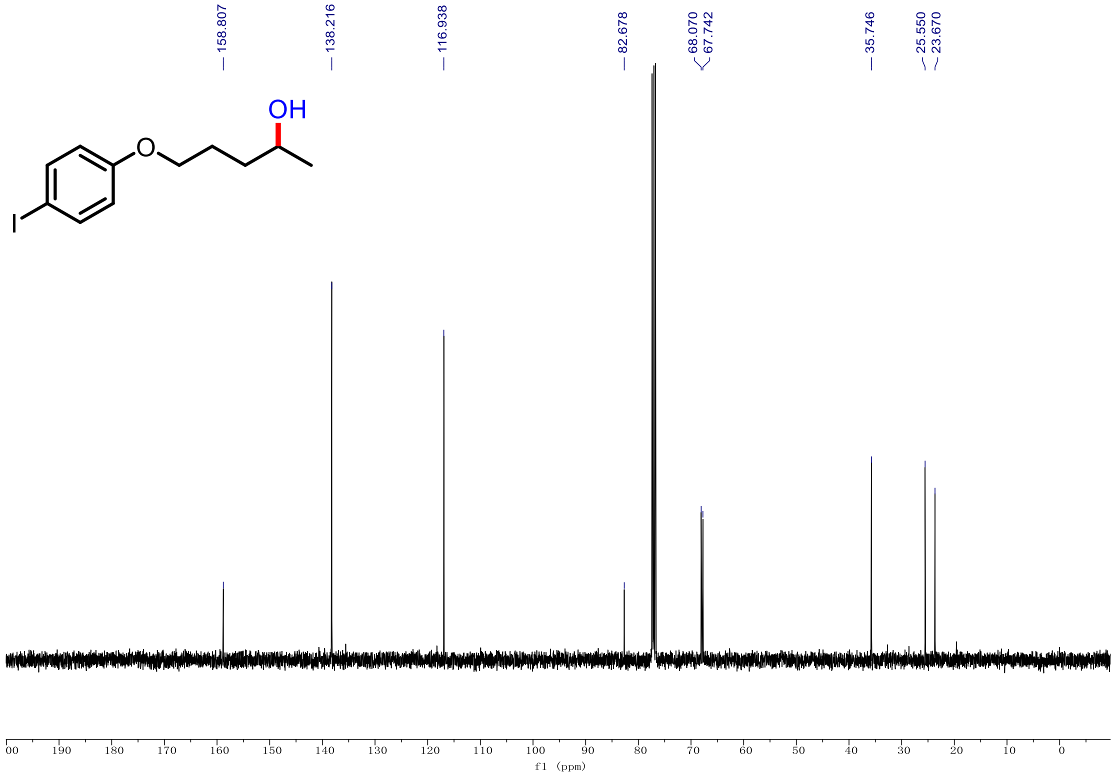

^{1}H NMR spectrum of **11** (**400 MHz, Chloroform-*d***)

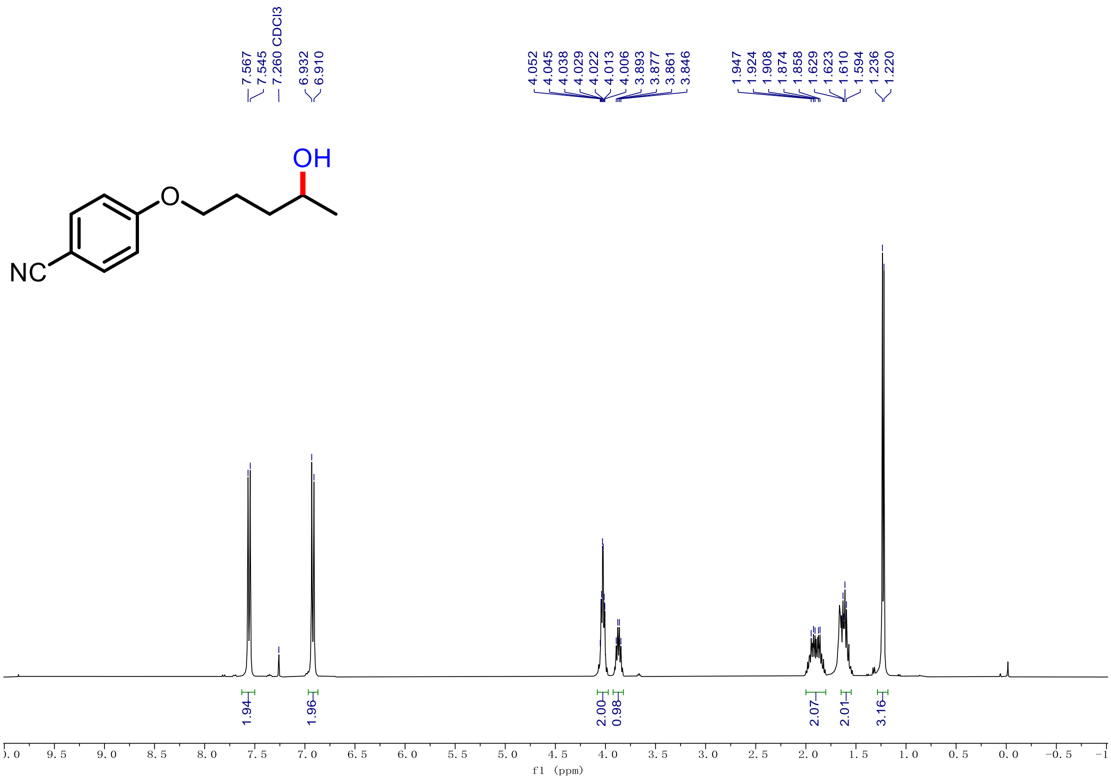

^{13}C NMR spectrum of **11** (**100 MHz, Chloroform-*d***)

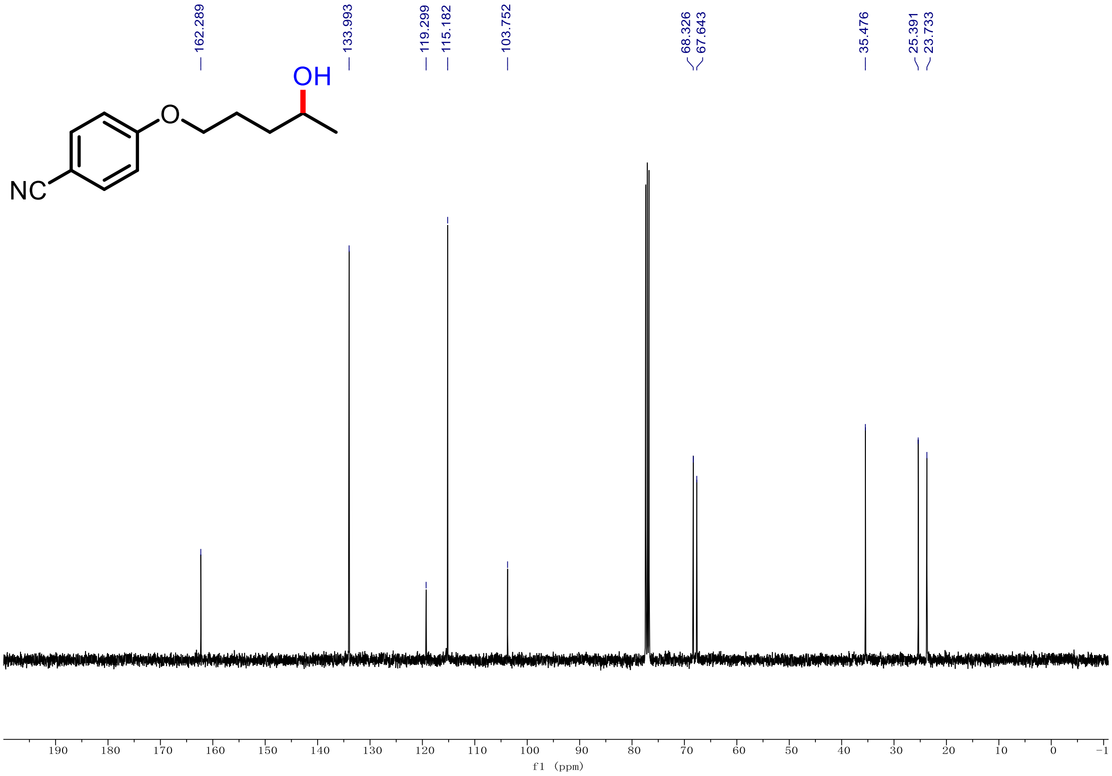

^{1}H NMR spectrum of **12** (**400 MHz, Chloroform-*d***)

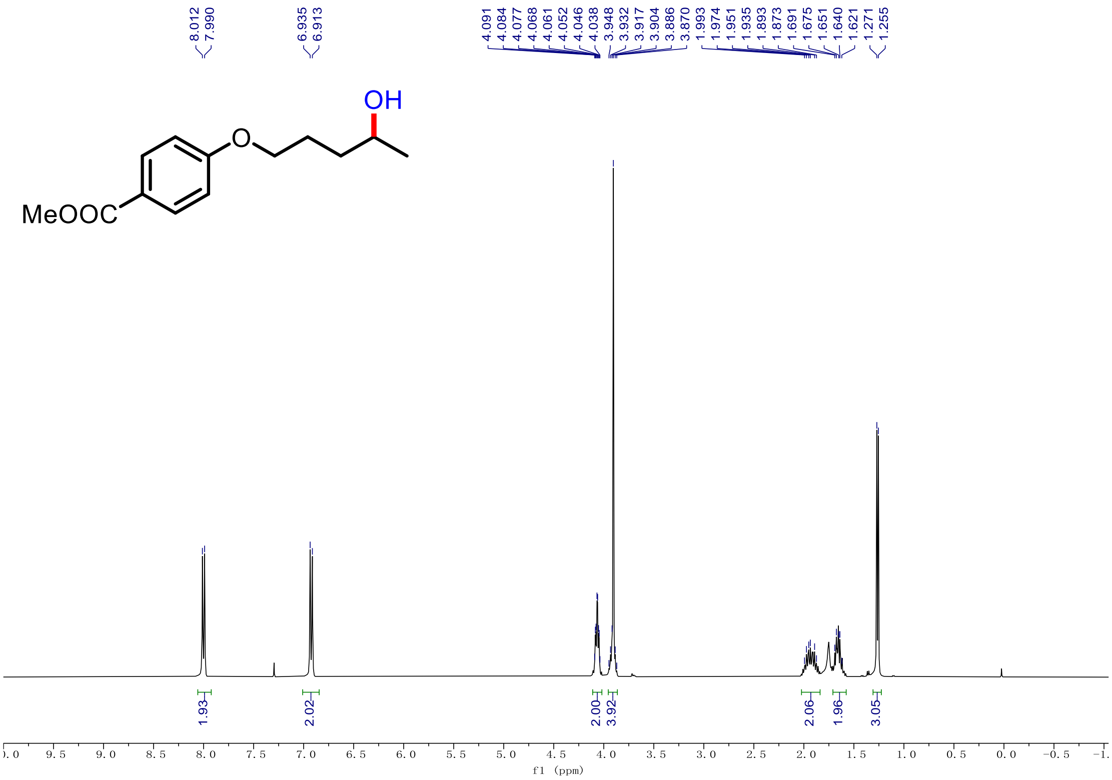

^{13}C NMR spectrum of **12** (**100 MHz, Chloroform-*d***)

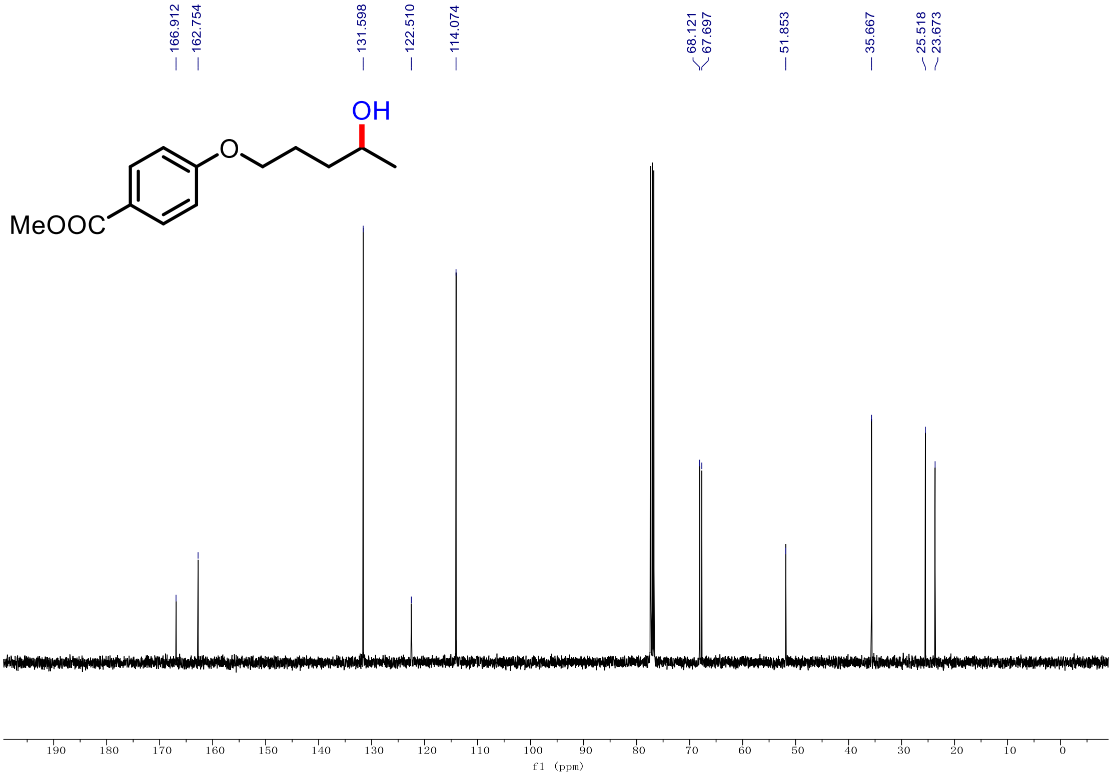

^{1}H NMR spectrum of **13** (**400 MHz, Chloroform-*d***)

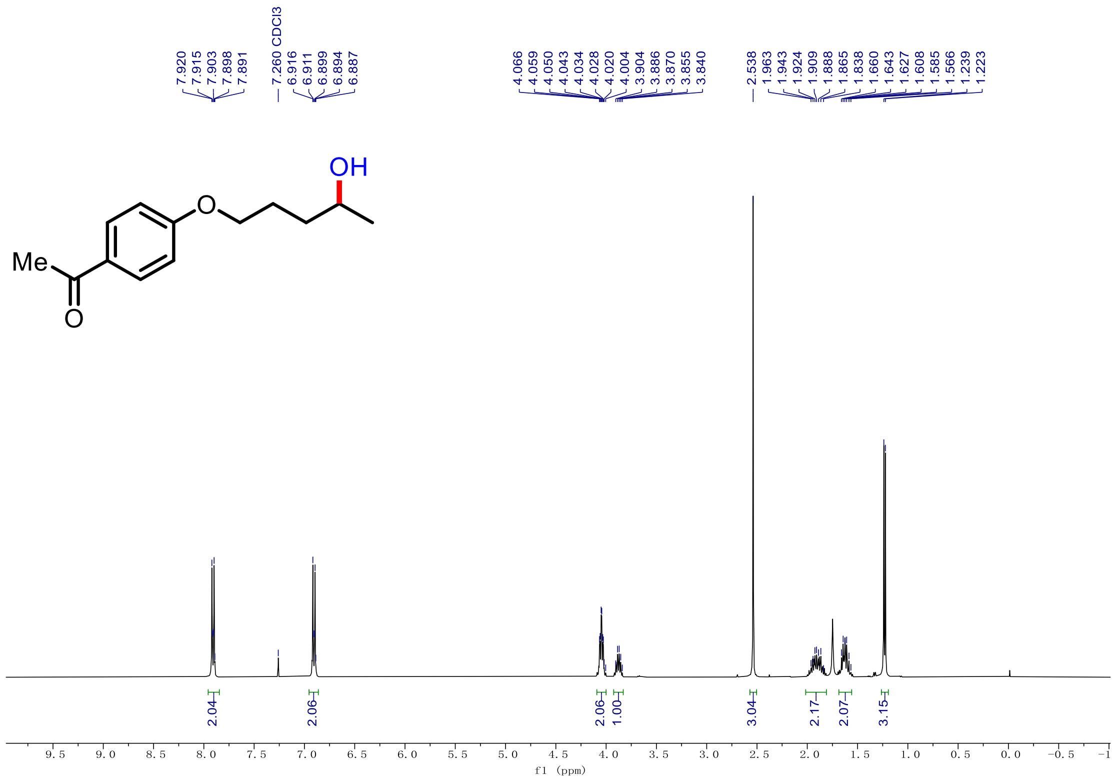

^{13}C NMR spectrum of **13** (**100 MHz, Chloroform-*d***)

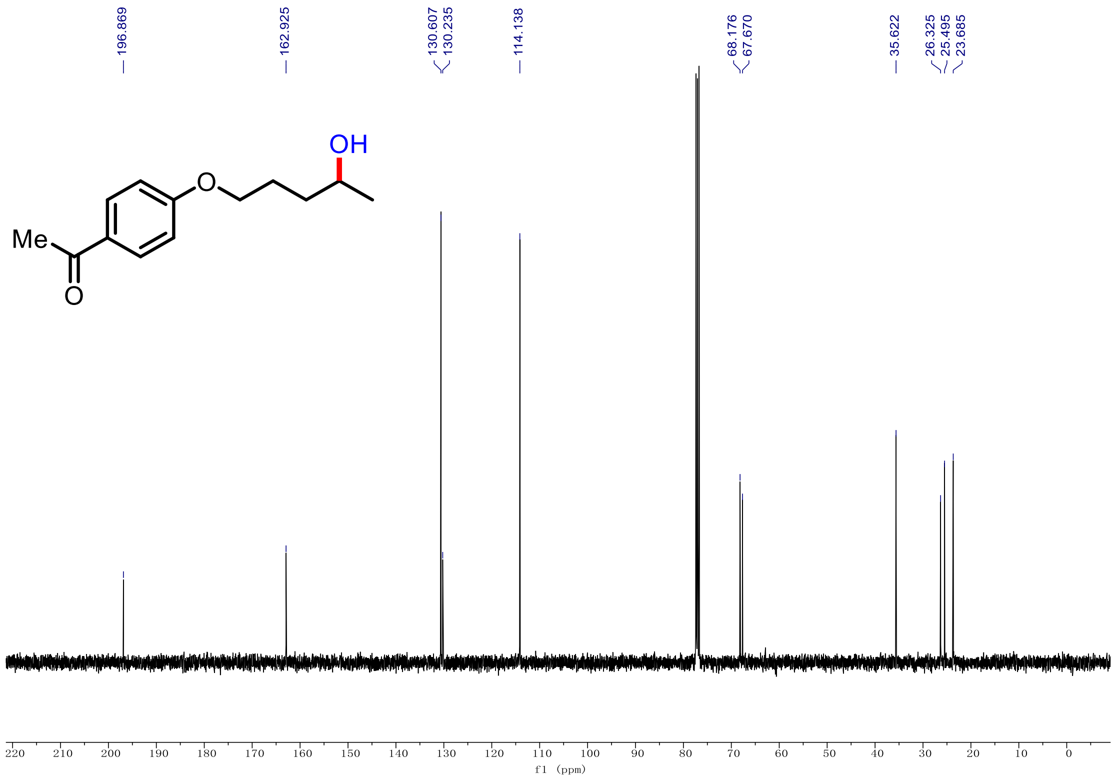

^{1}H NMR spectrum of **14** (**400 MHz, Chloroform-*d***)

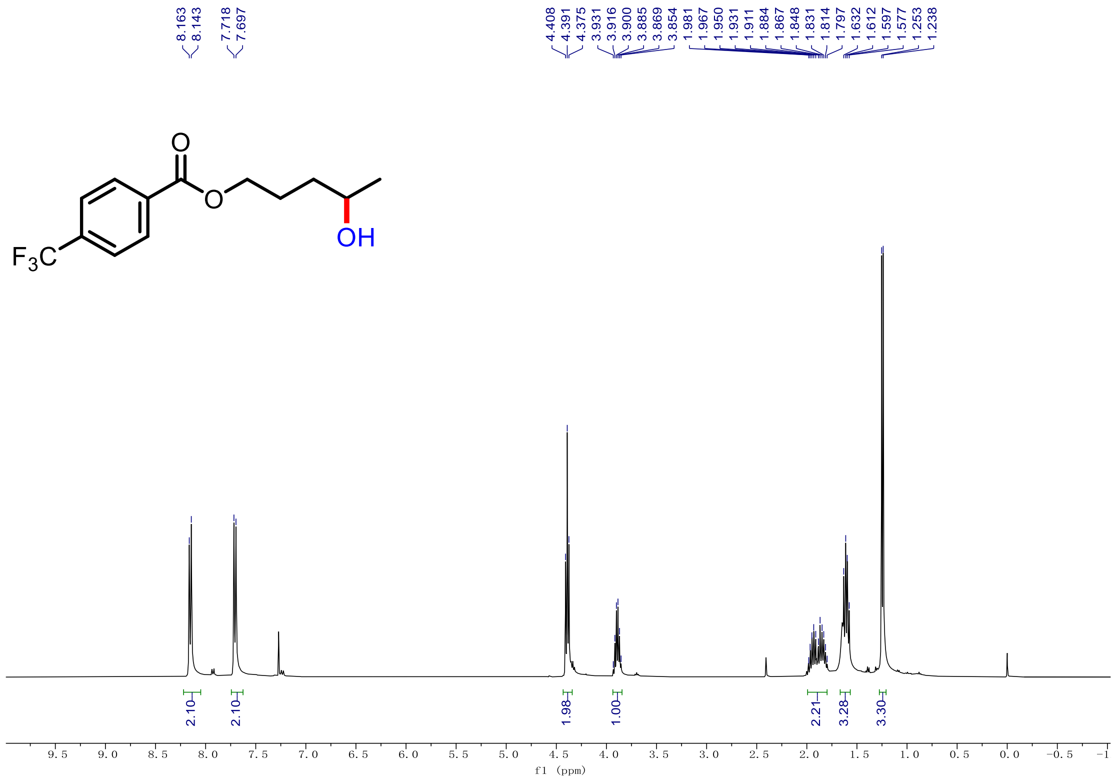

^{13}C NMR spectrum of **14** (**150 MHz, Chloroform-*d***)

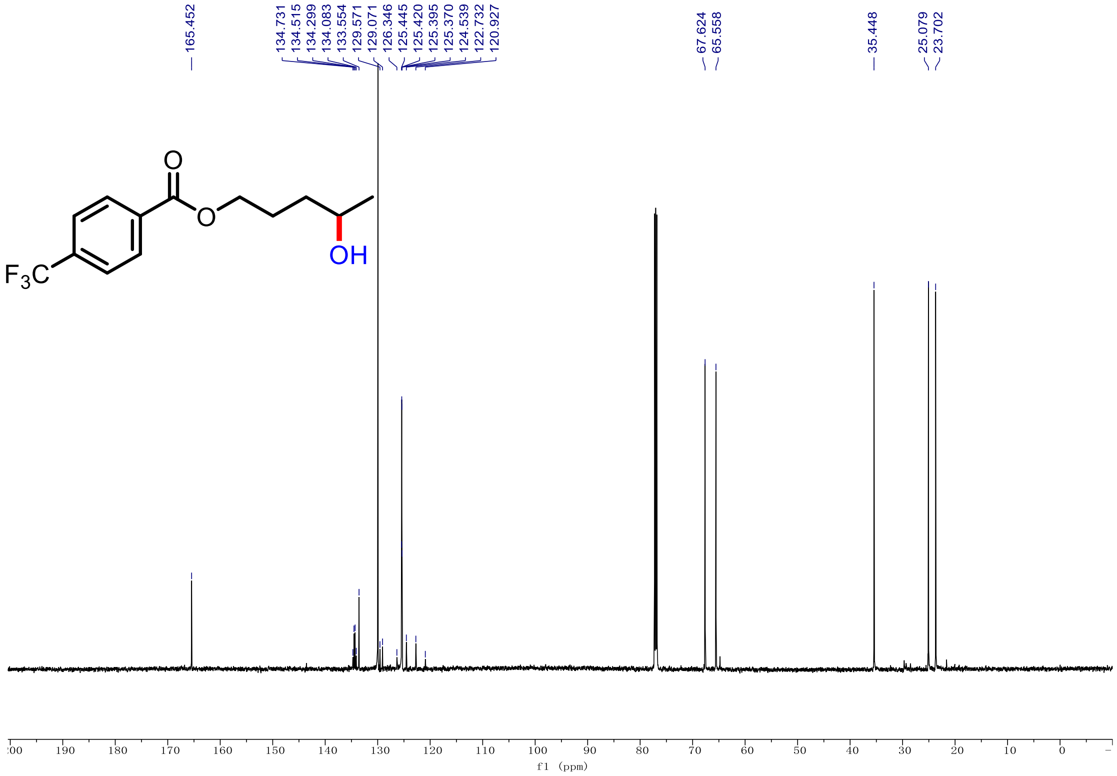

^{19}F NMR spectrum of **14** (**376 MHz, Chloroform-*d***)

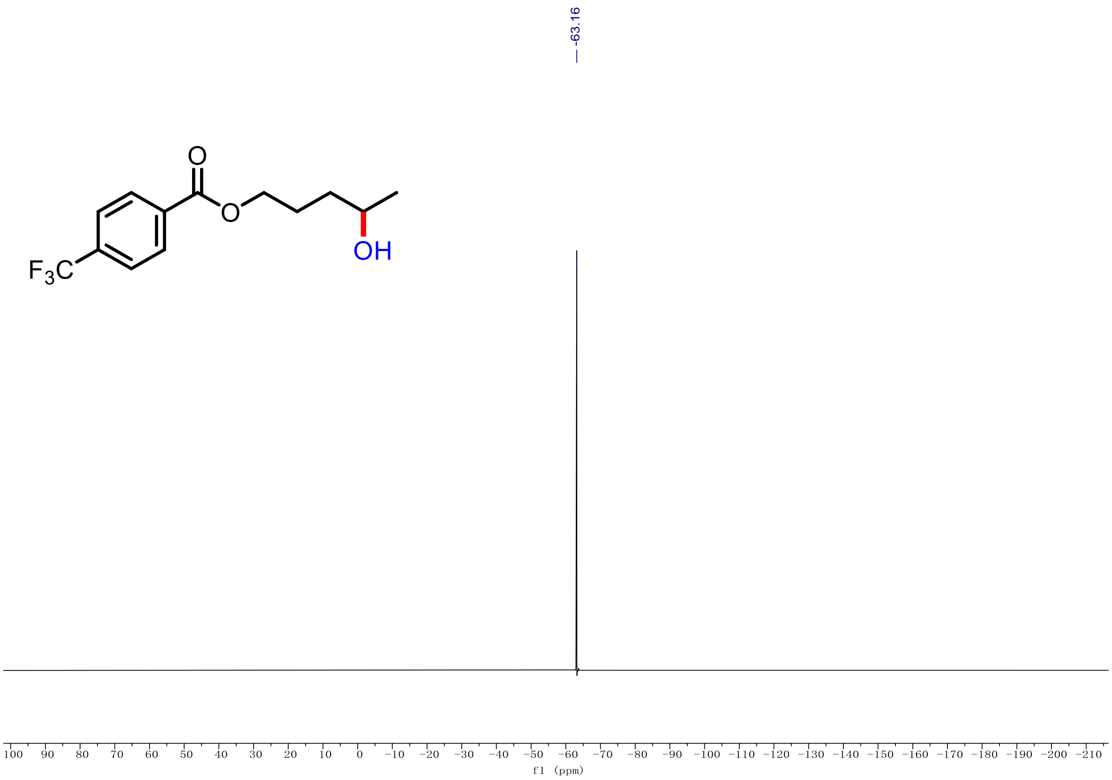

^{1}H NMR spectrum of **15** (**400 MHz, Chloroform-*d***)

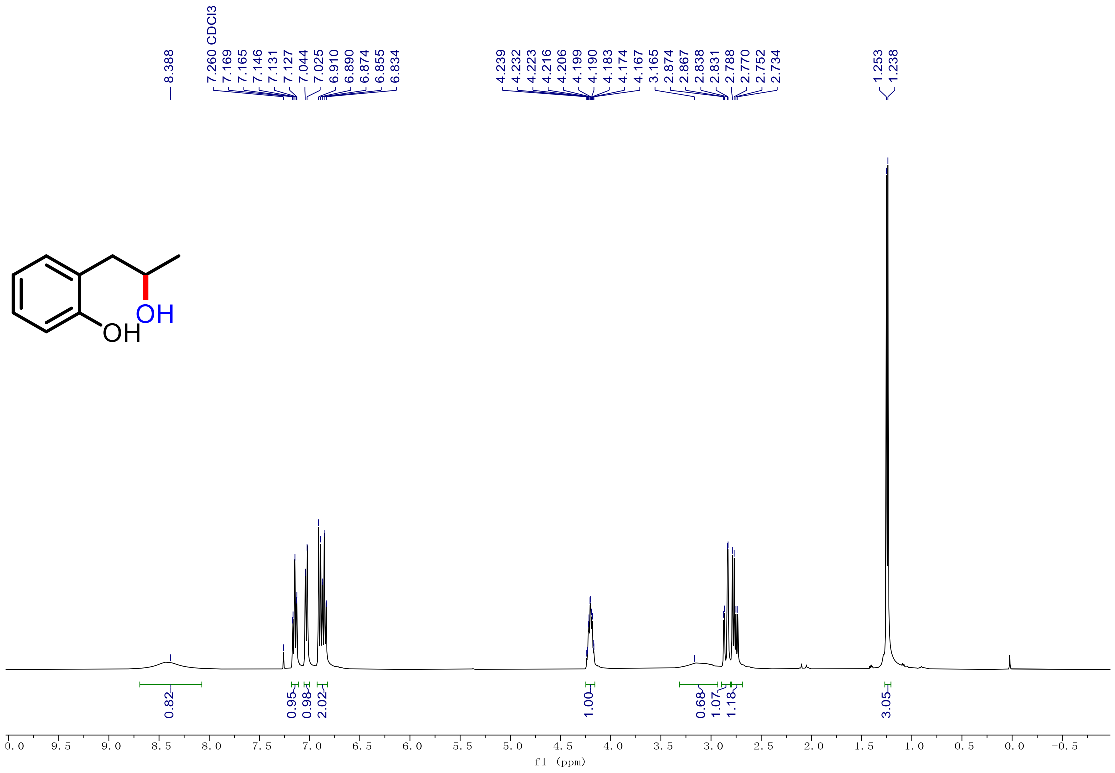

^{13}C NMR spectrum of **15** (**100 MHz, Chloroform-*d***)

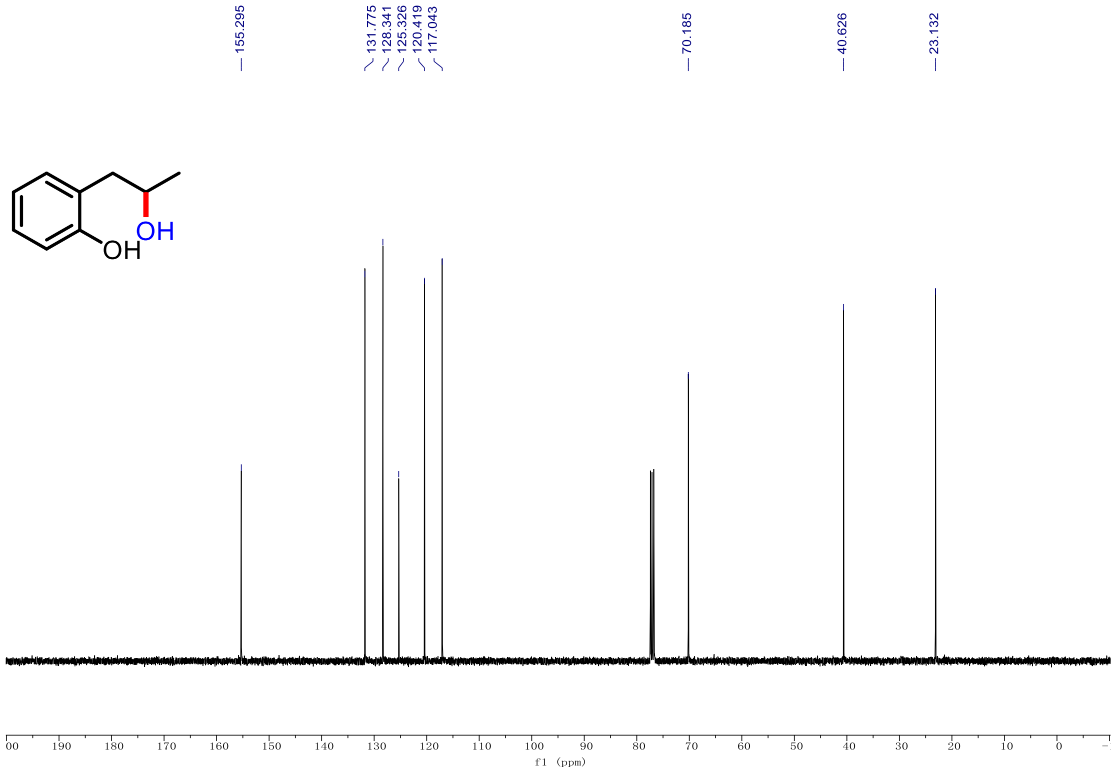

^{1}H NMR spectrum of **16** (**400 MHz, Chloroform-*d***)

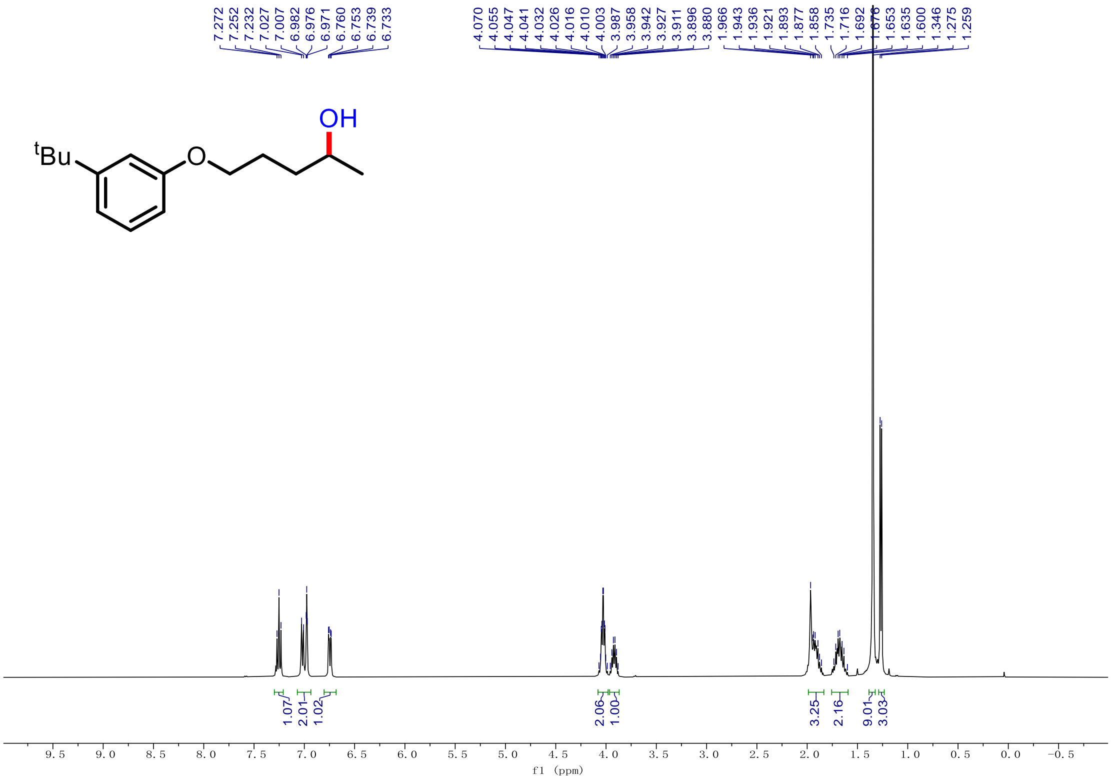

^{13}C NMR spectrum of **16** (**100 MHz, Chloroform-*d***)

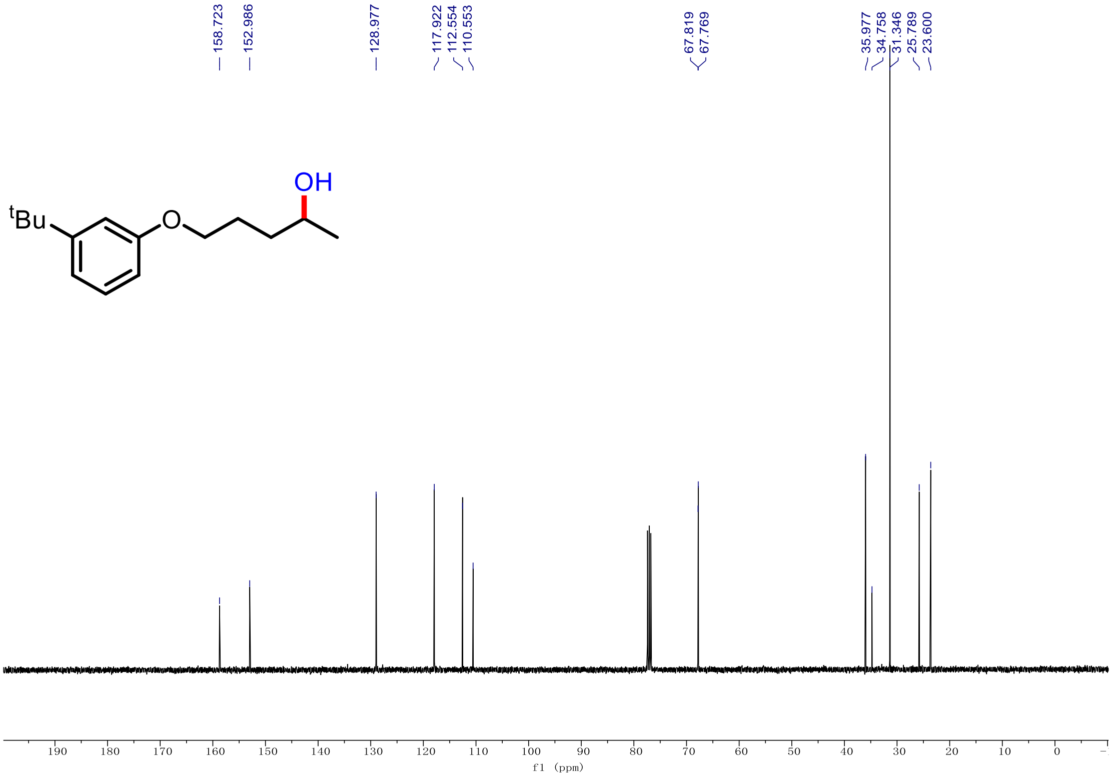

^{1}H NMR spectrum of **17** (**400 MHz, Chloroform-*d***)

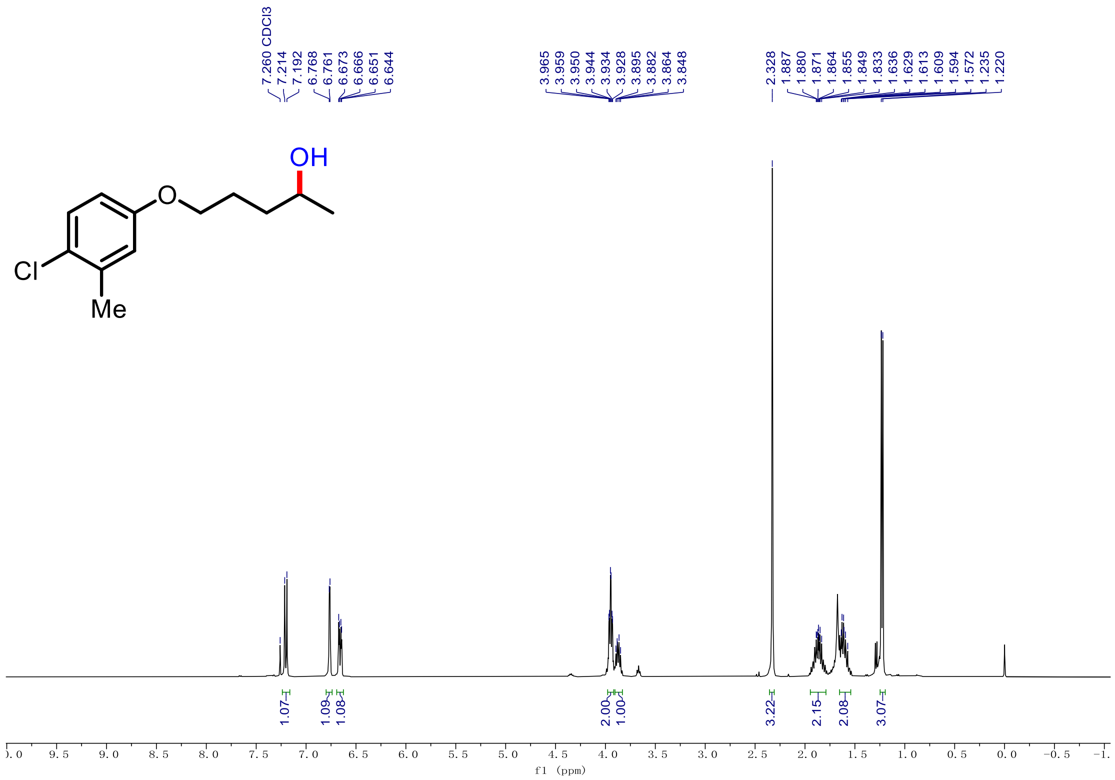

^{13}C NMR spectrum of **17** (**100 MHz, Chloroform-*d***)

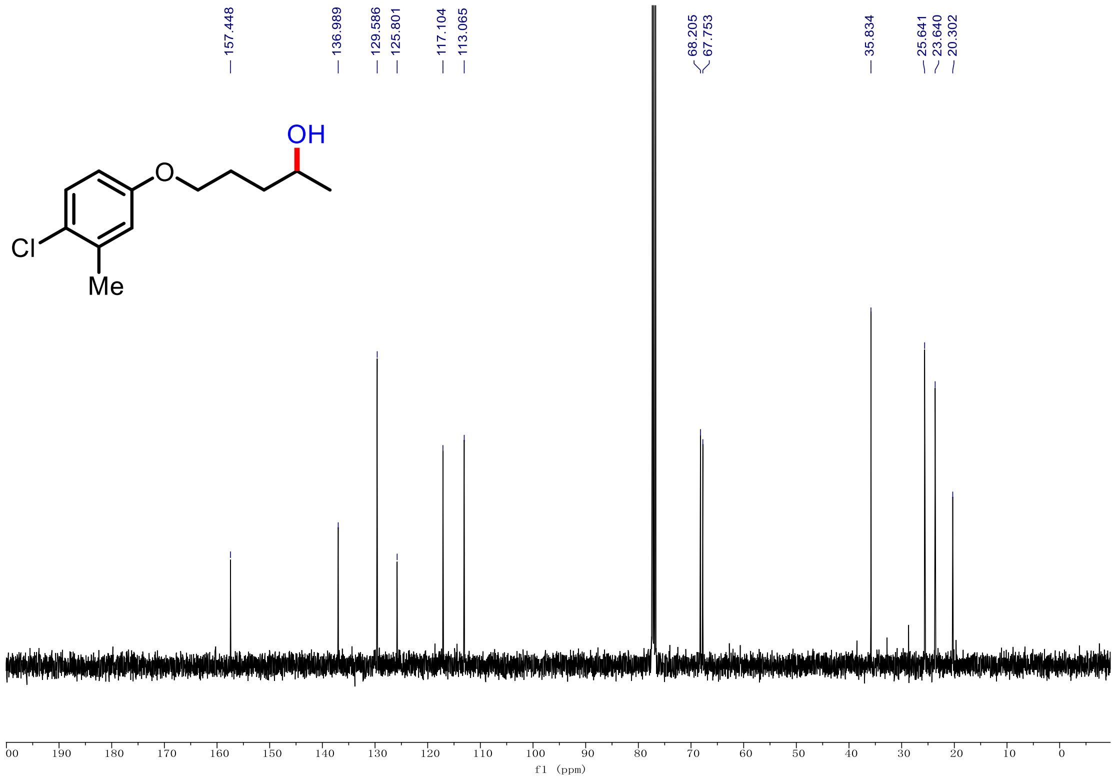

^{1}H NMR spectrum of **18** (**400 MHz, Chloroform-*d***)

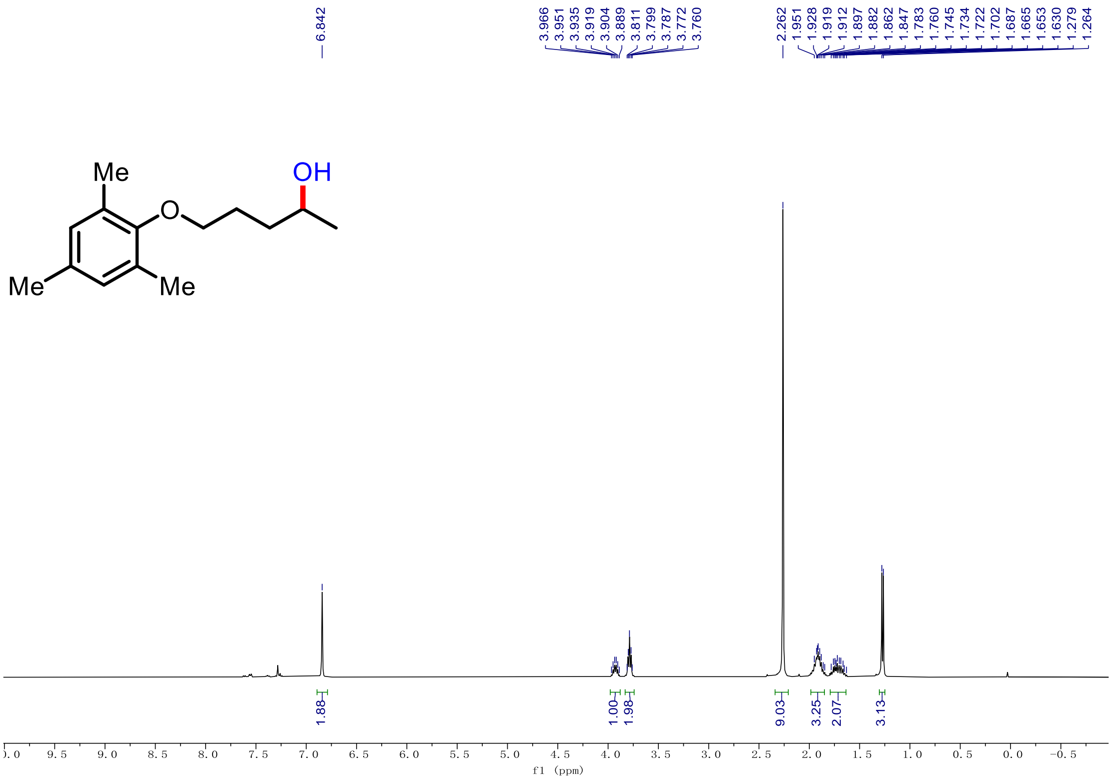

^{13}C NMR spectrum of **18** (**150 MHz, Chloroform-*d***)

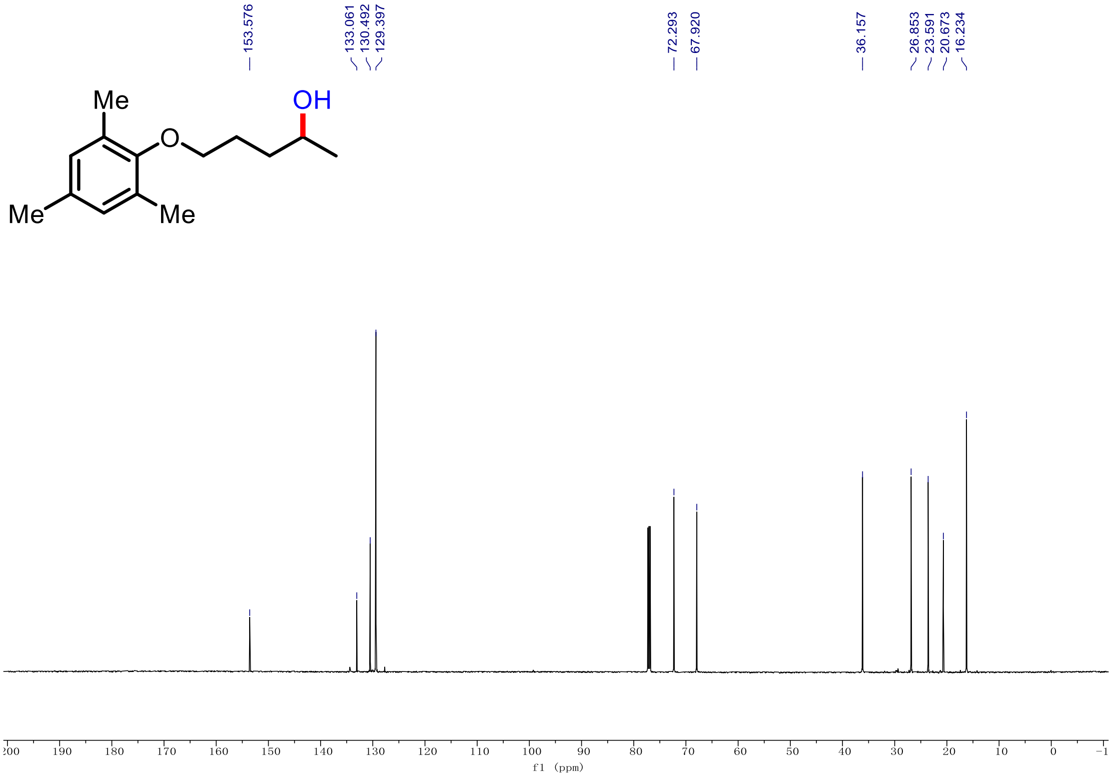

^{1}H NMR spectrum of **19** (**400 MHz, Chloroform-*d***)

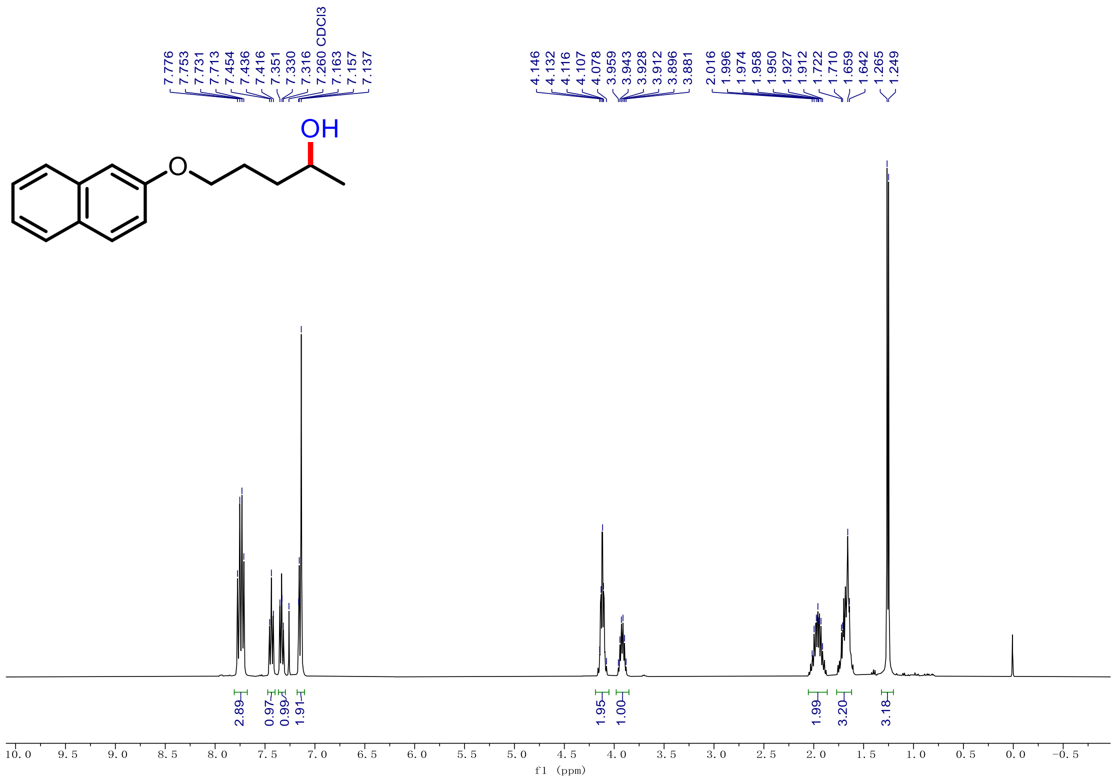

^{13}C NMR spectrum of **19** (**100 MHz, Chloroform-*d***)

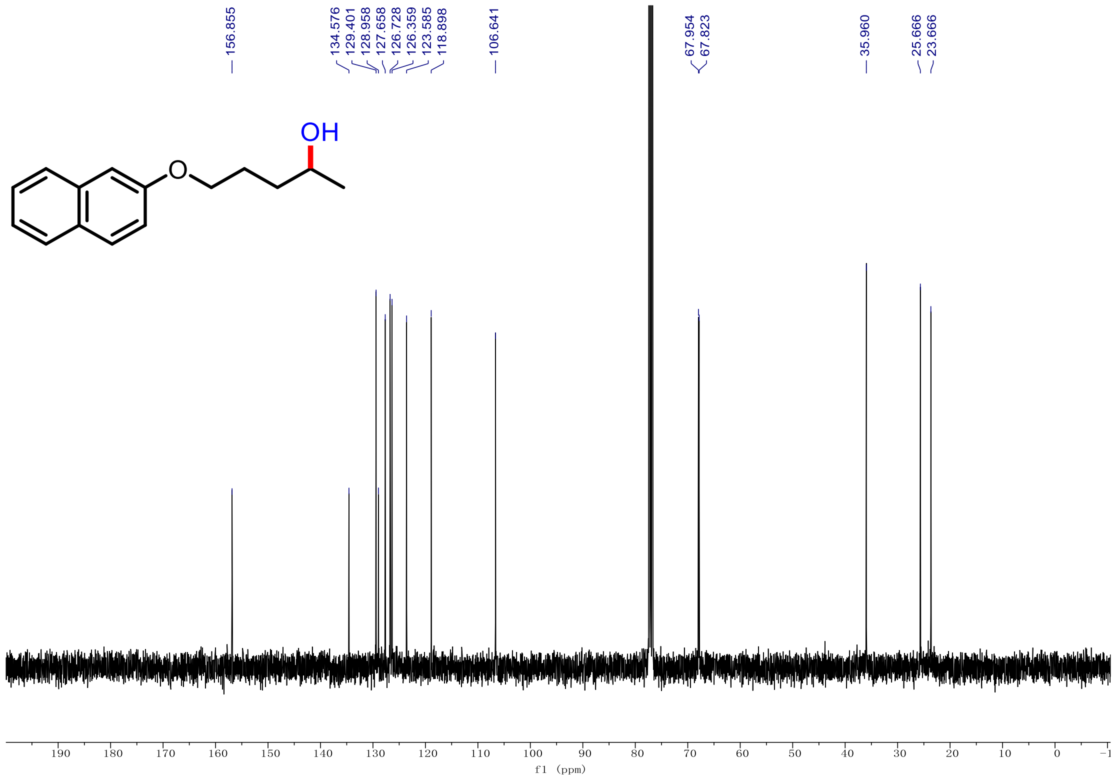

^{1}H NMR spectrum of **20** (**400 MHz, Chloroform-*d***)

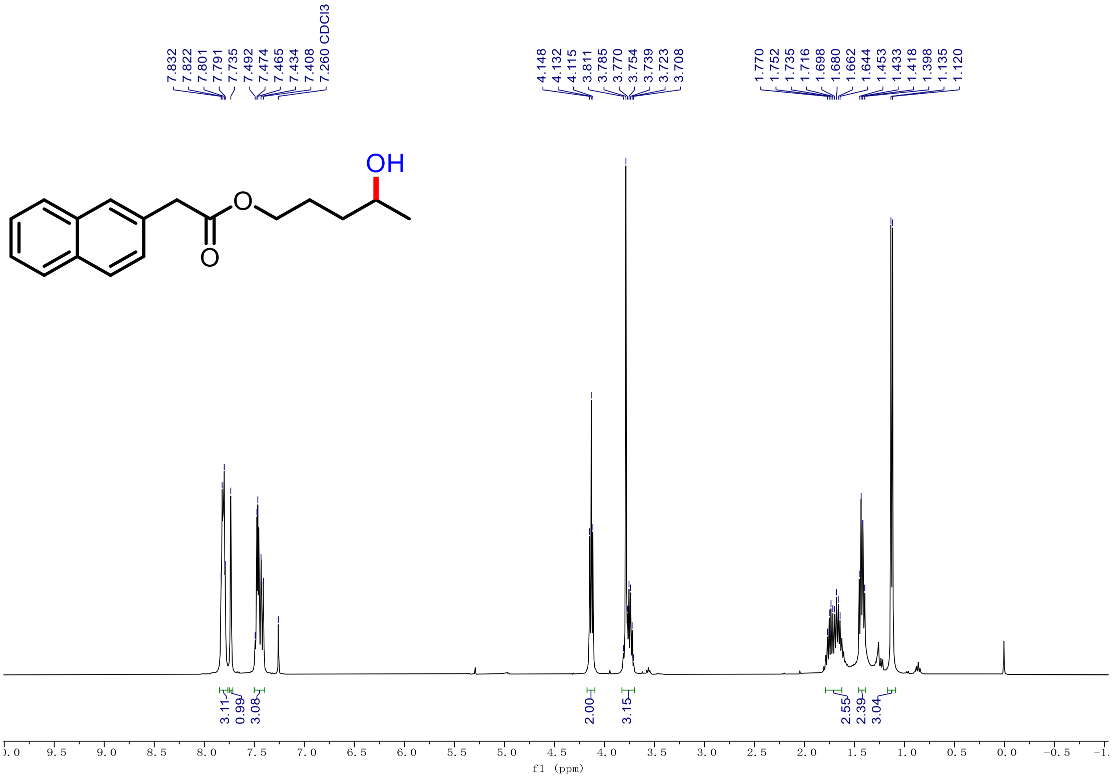

^{13}C NMR spectrum of **20** (**100 MHz, Chloroform-*d***)

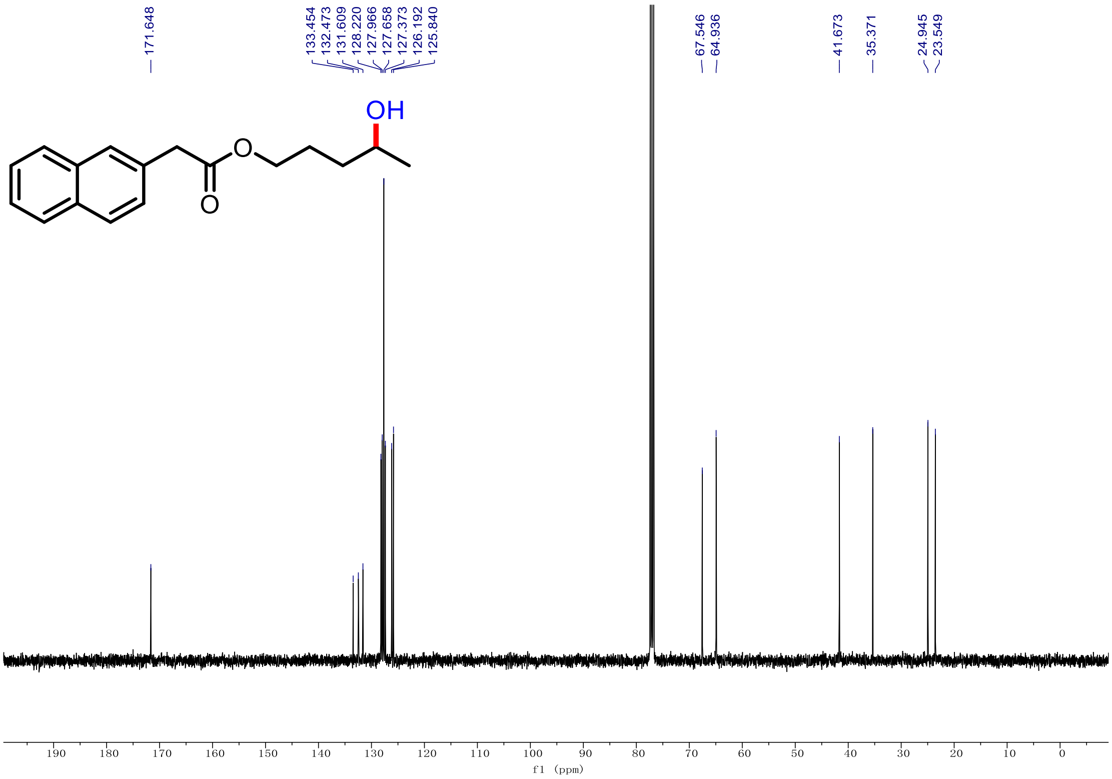

^{1}H NMR spectrum of **21** (**400 MHz, Chloroform-*d***)

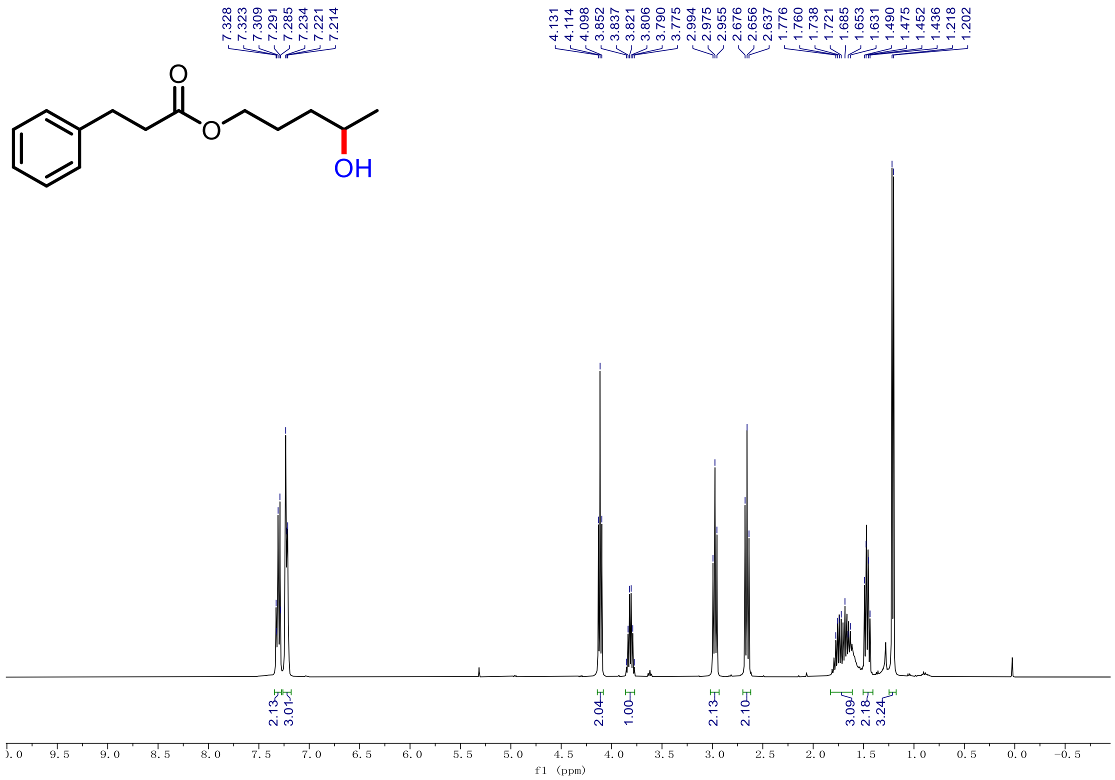

^{13}C NMR spectrum of **21** (**100 MHz, Chloroform-*d***)

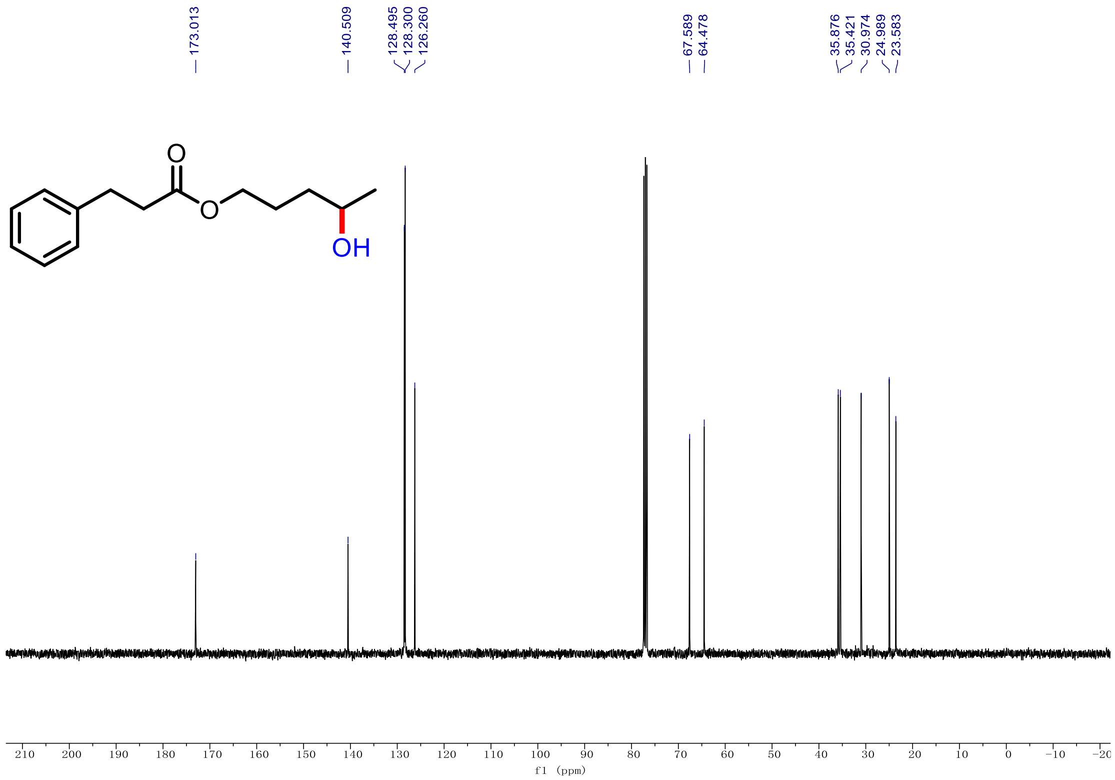

^{1}H NMR spectrum of **22** (**400 MHz, Chloroform-*d***)

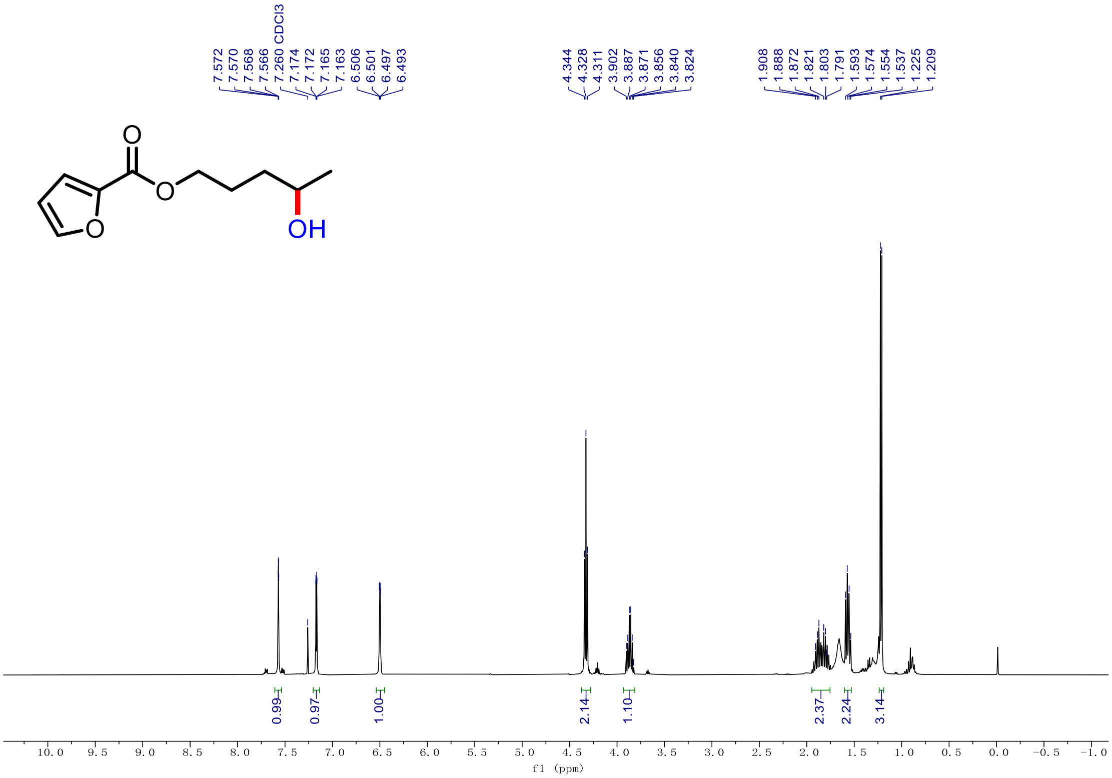

^{13}C NMR spectrum of **22** (**100 MHz, Chloroform-*d***)

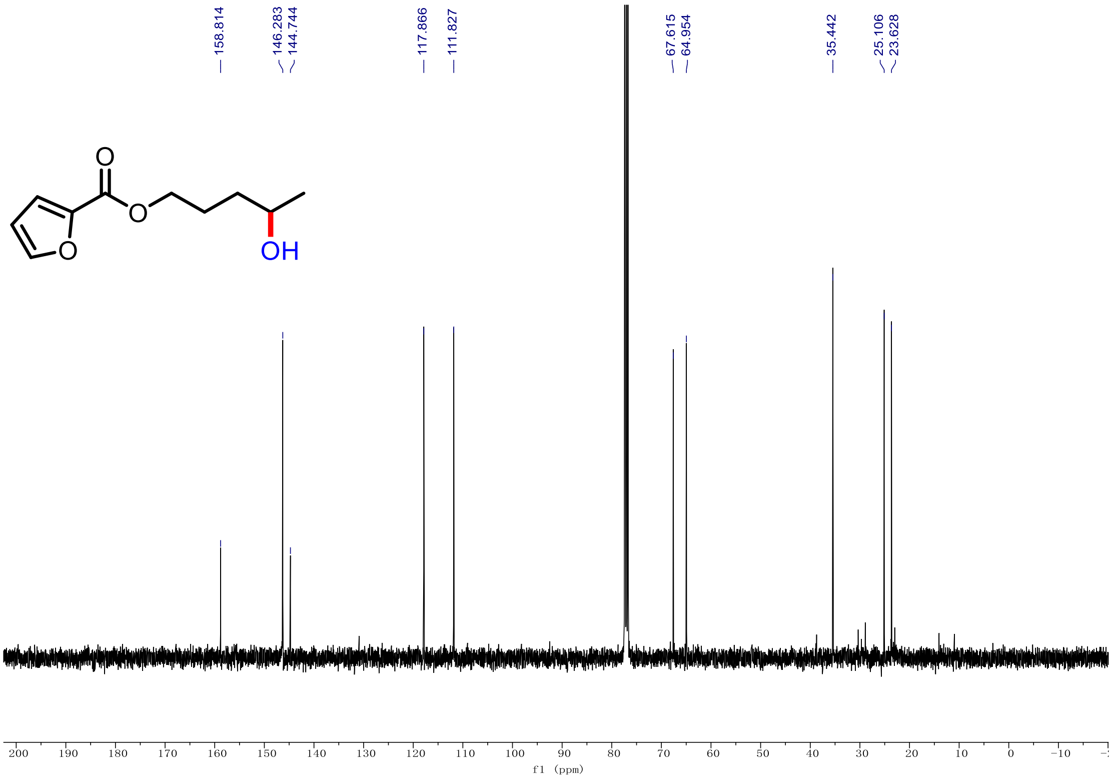

^{1}H NMR spectrum of **23** (**400 MHz, Chloroform-*d***)

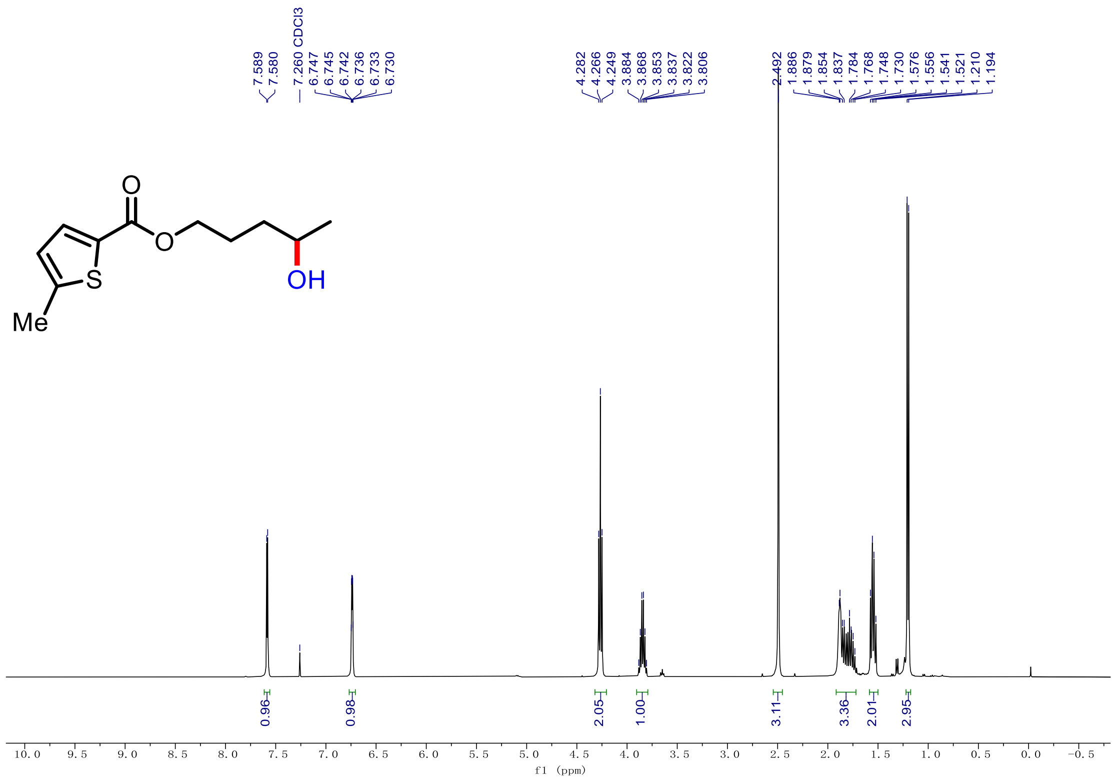

^{13}C NMR spectrum of **23** (**100 MHz, Chloroform-*d***)

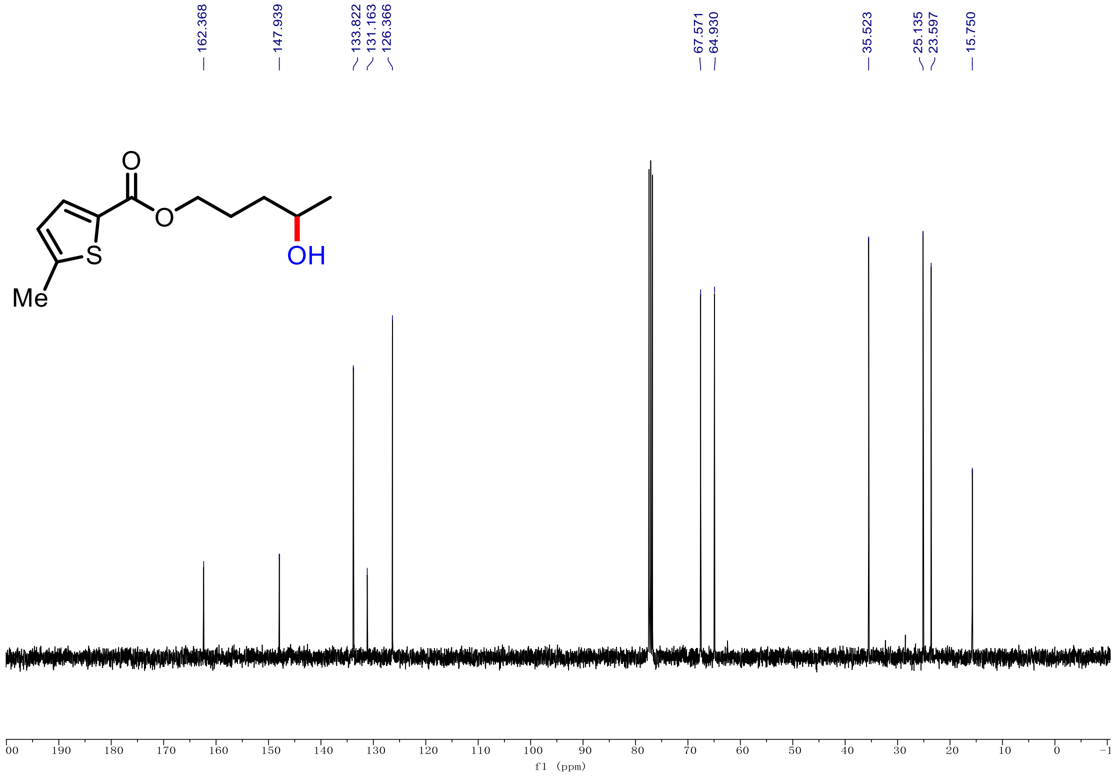

^{1}H NMR spectrum of **24** (**600 MHz, Chloroform-*d***)

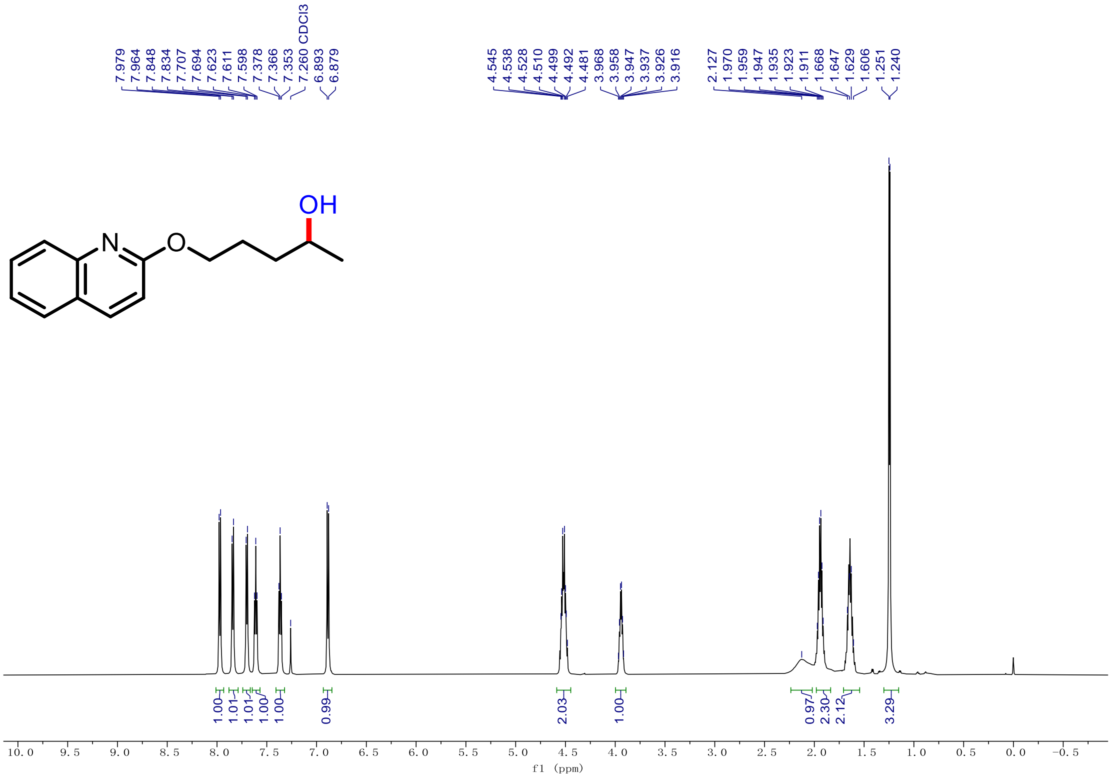

^{13}C NMR spectrum of **24** (**150 MHz, Chloroform-*d***)

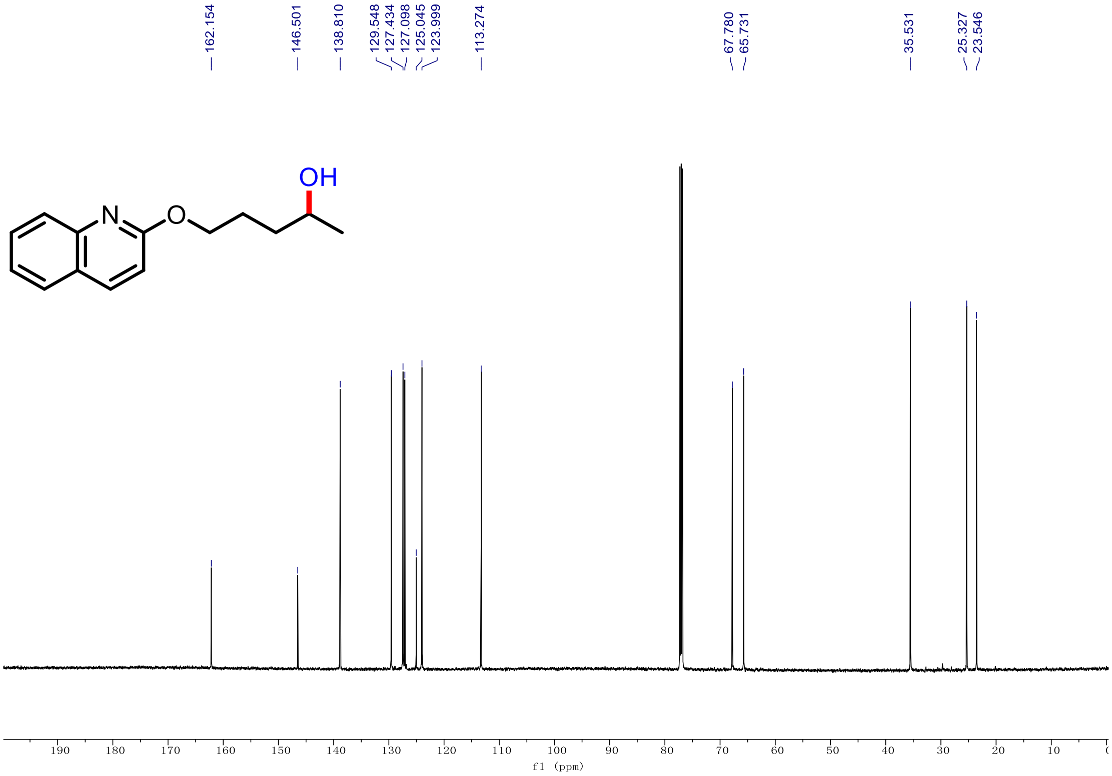

^{1}H NMR spectrum of **25** (**400 MHz, Chloroform-*d***)

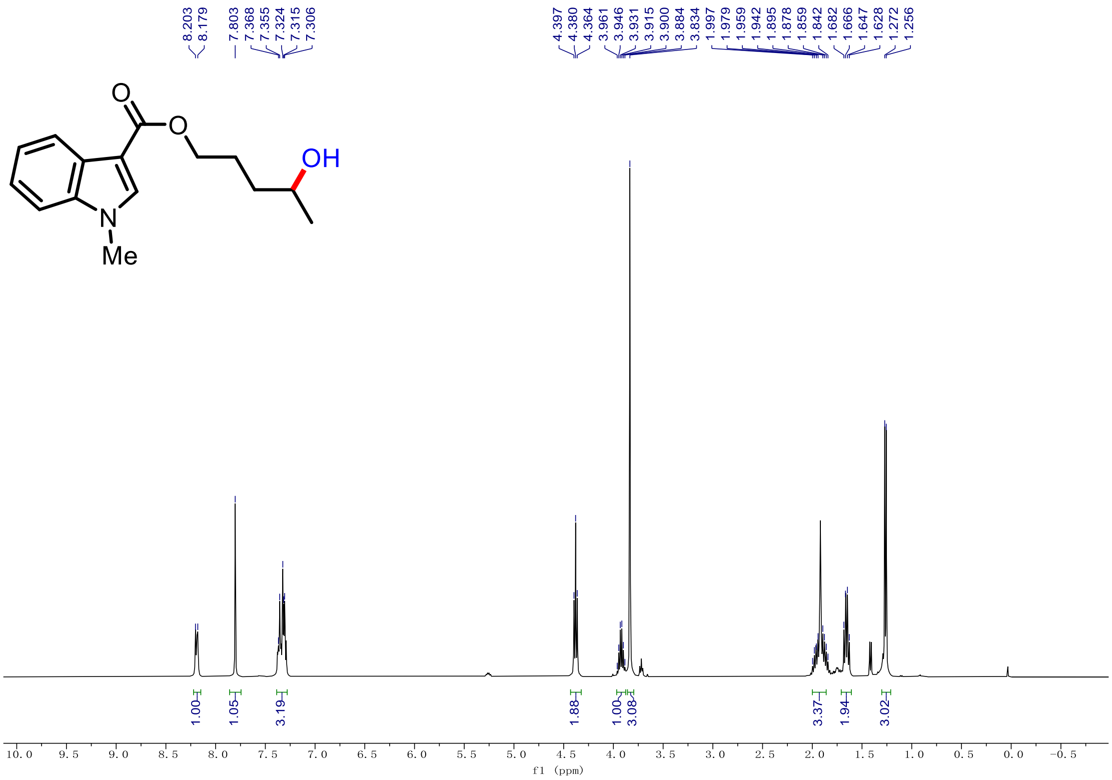

^{13}C NMR spectrum of **25** (**100 MHz, Chloroform-*d***)

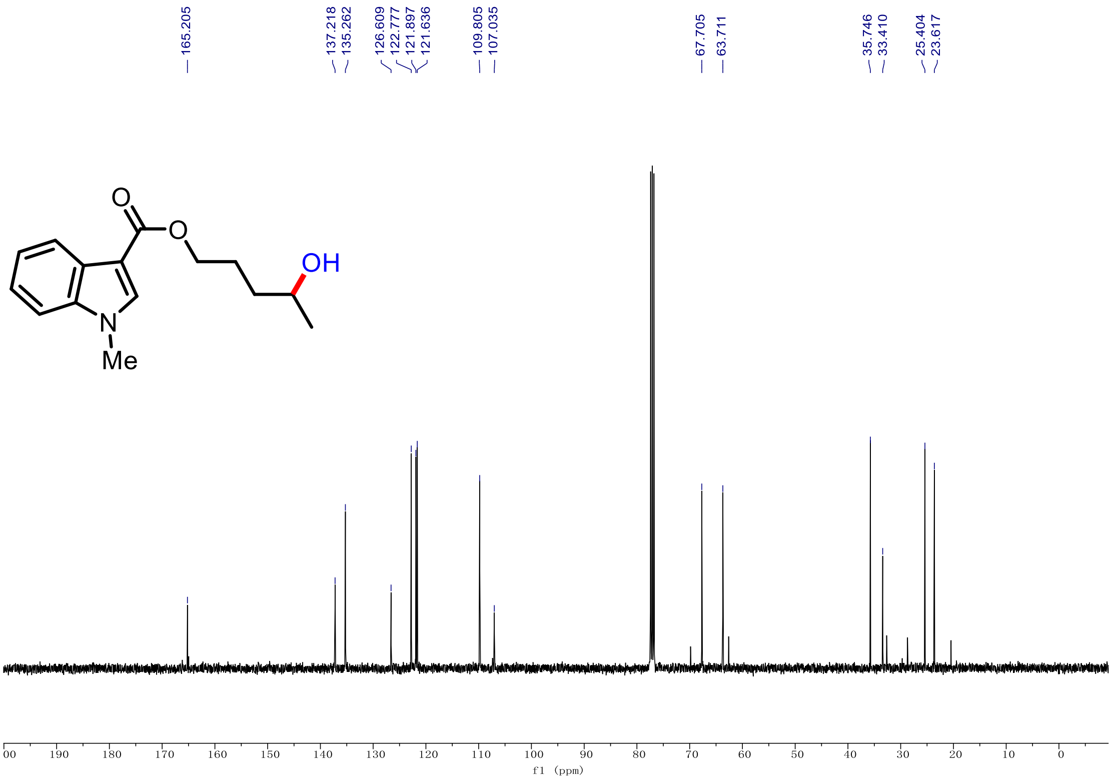

^{1}H NMR spectrum of **26** (**400 MHz, Chloroform-*d***)

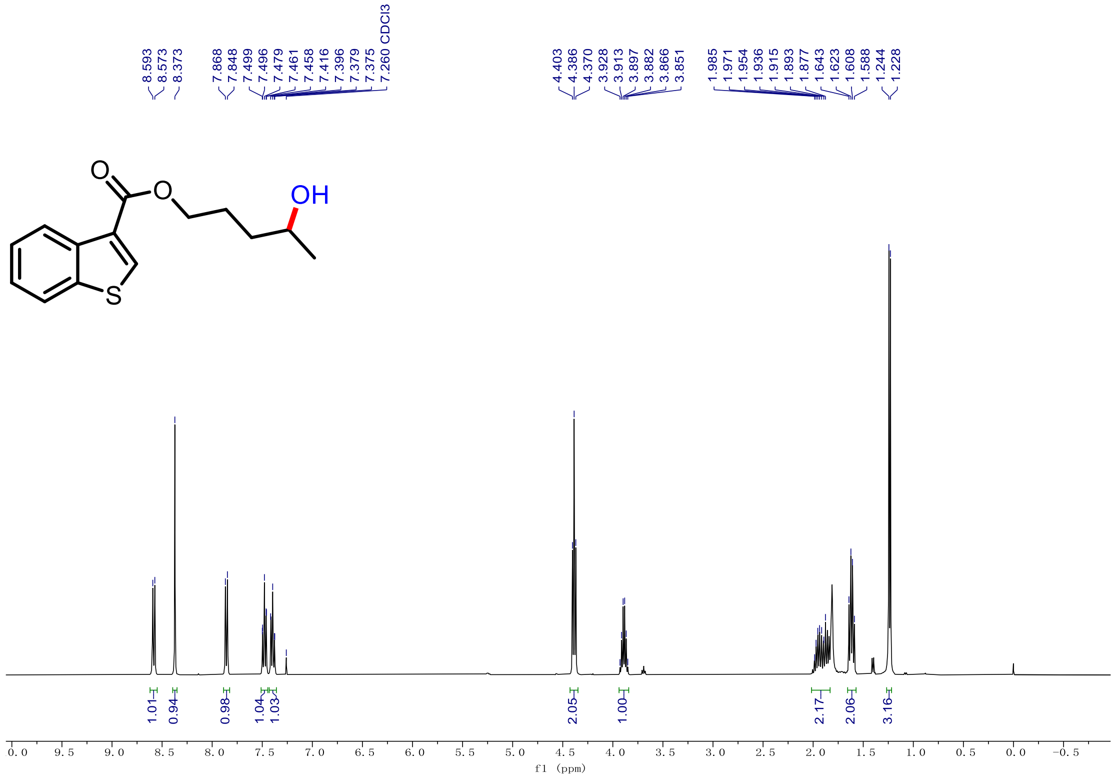

^{13}C NMR spectrum of **26** (**100 MHz, Chloroform-*d***)

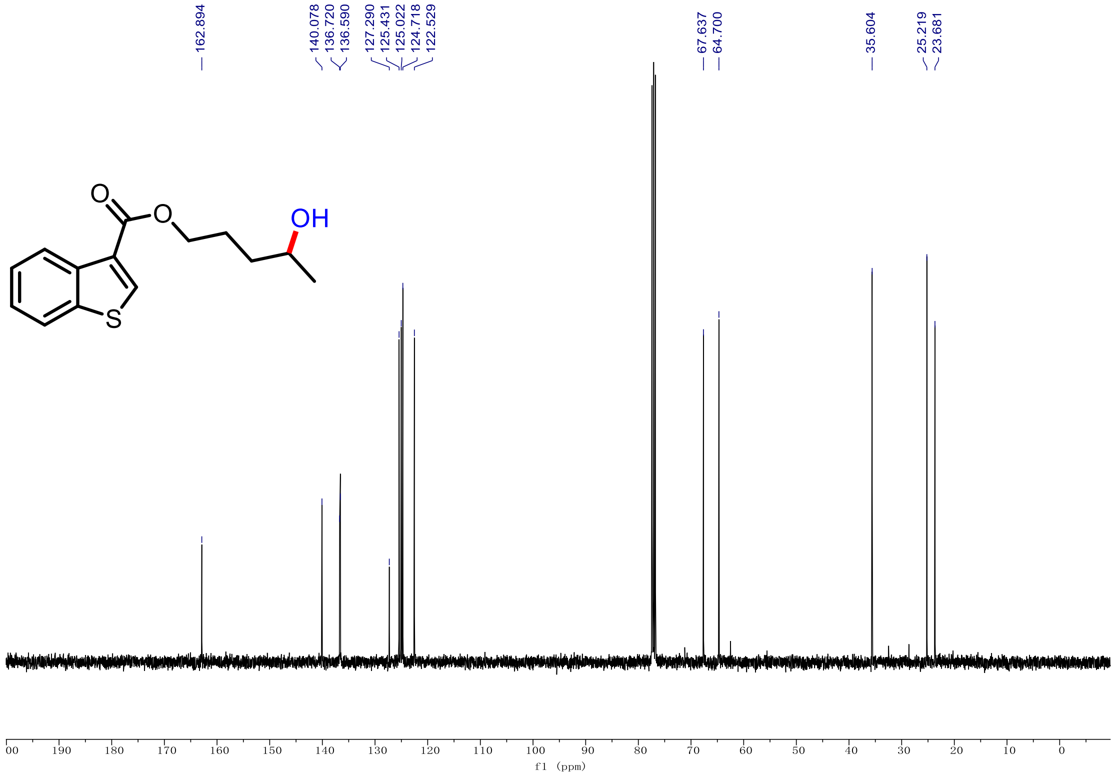

^{1}H NMR spectrum of **27** (**400 MHz, Chloroform-*d***)

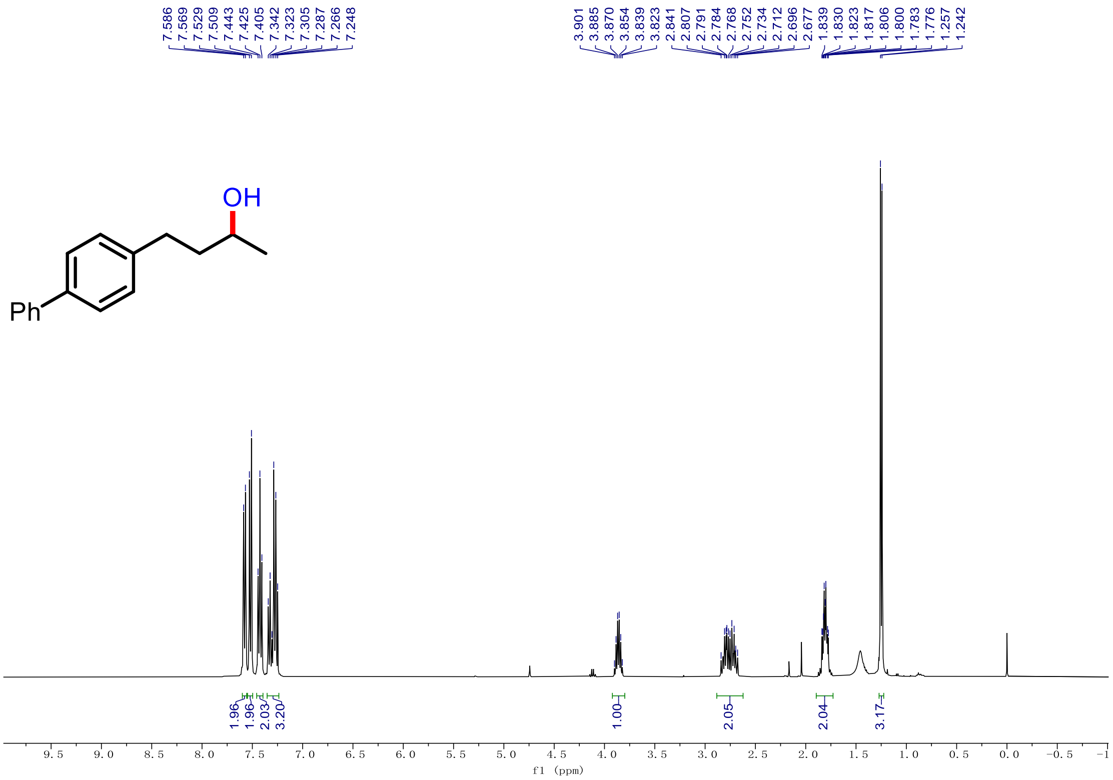

^{13}C NMR spectrum of **27** (**100 MHz, Chloroform-*d***)

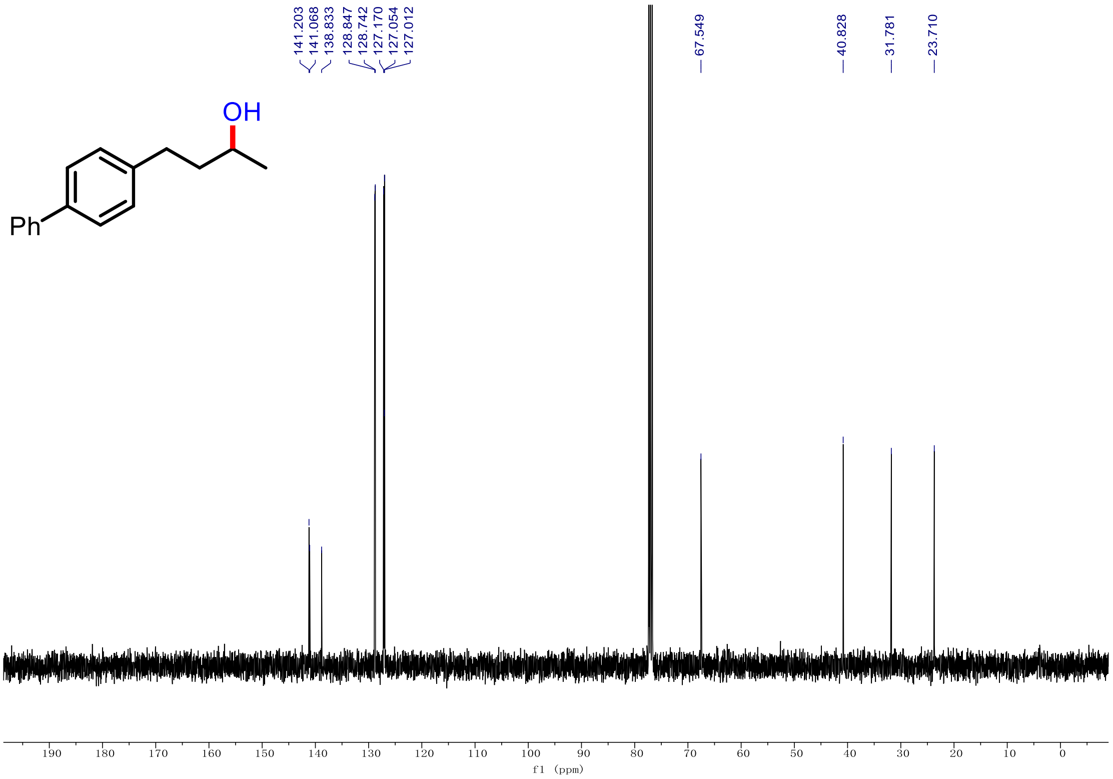

^{1}H NMR spectrum of **28** (**400 MHz, Chloroform-*d***)

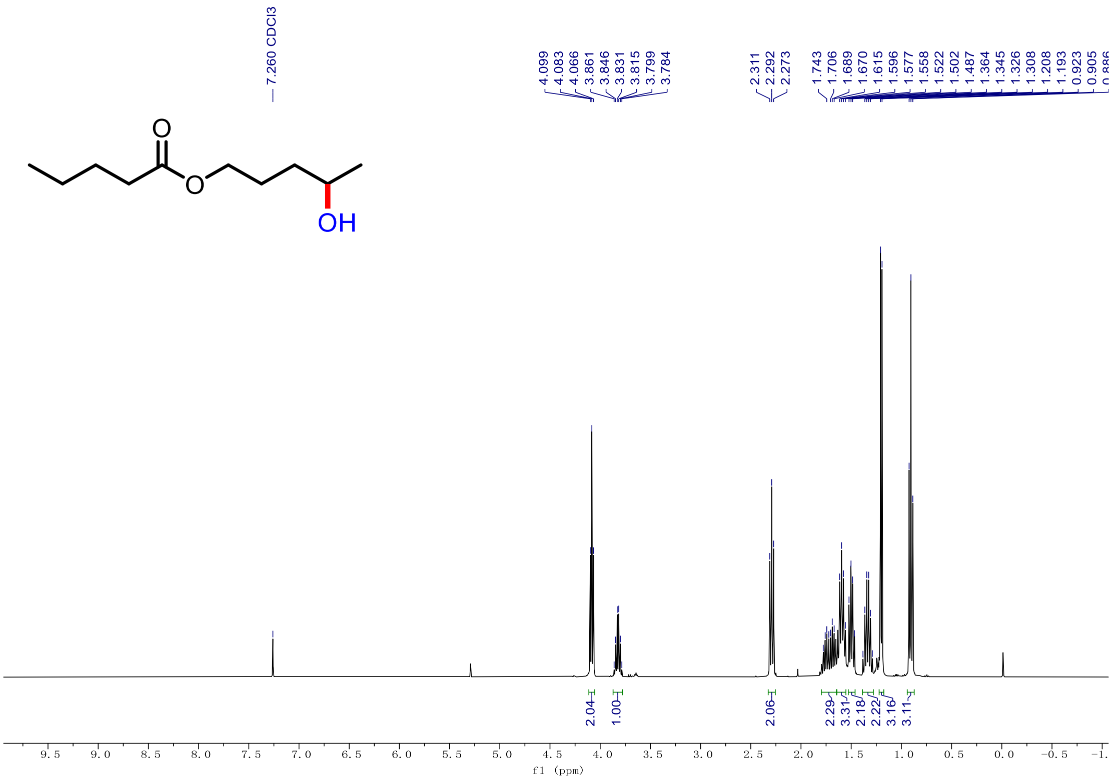

^{13}C NMR spectrum of **28** (**100 MHz, Chloroform-*d***)

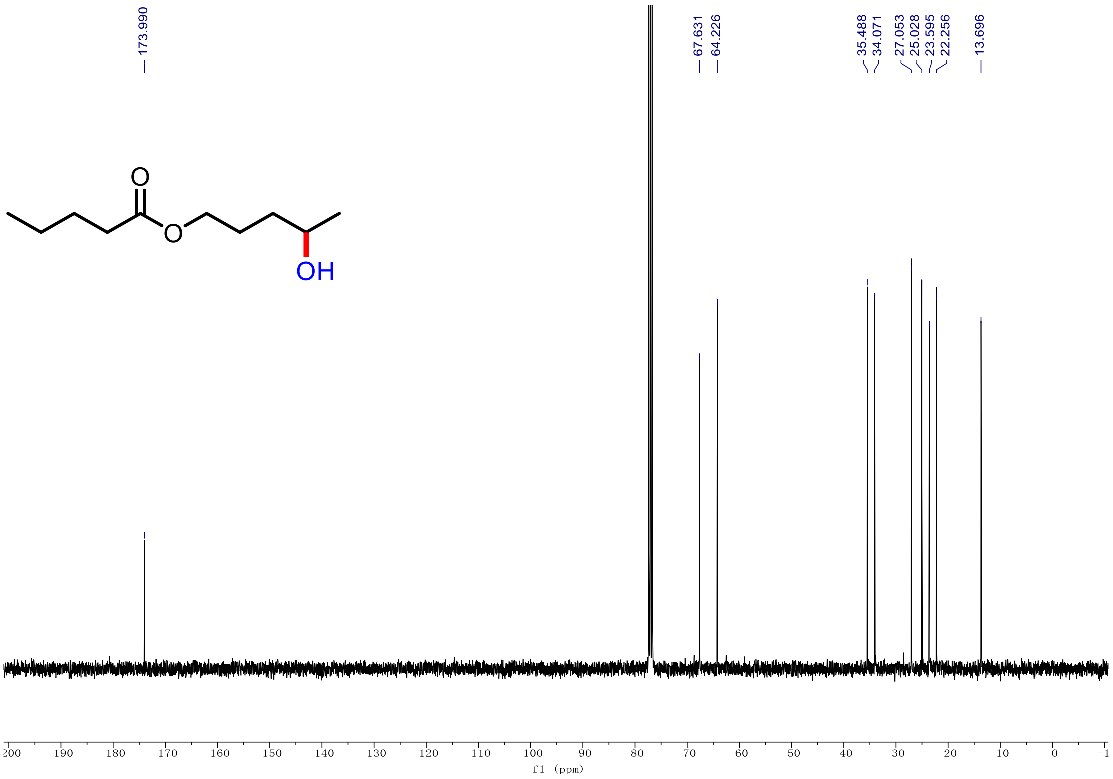

^{1}H NMR spectrum of **29** (**400 MHz, Chloroform-*d***)

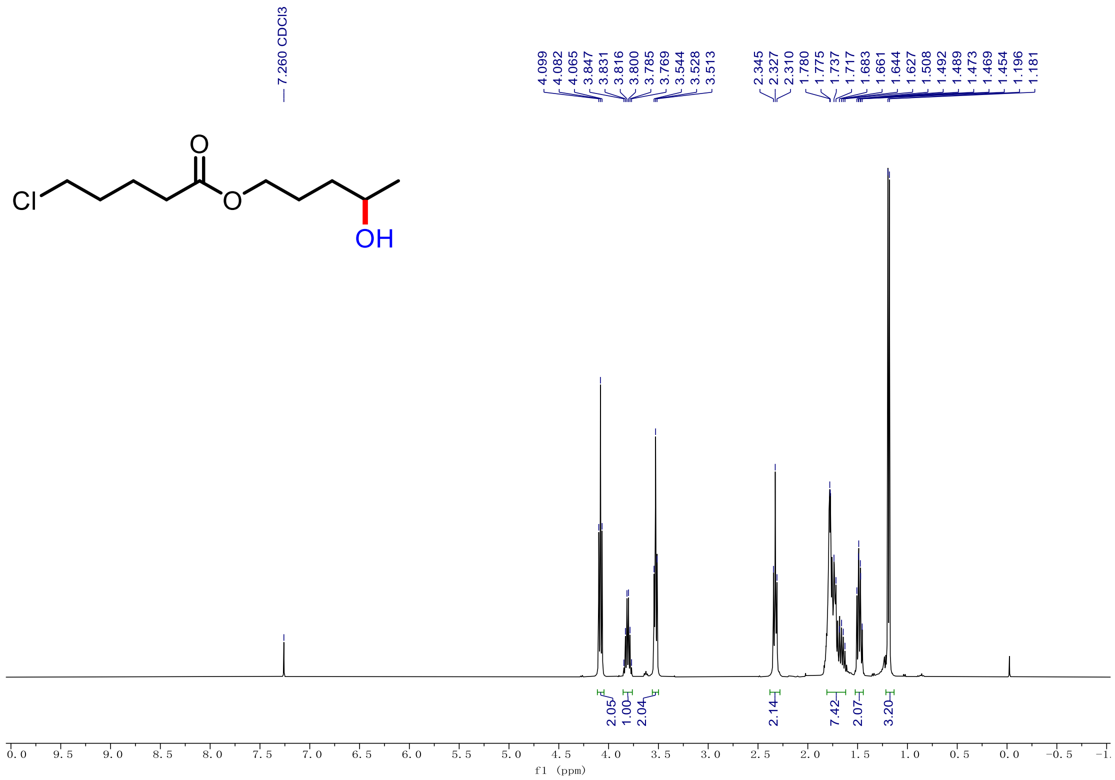

^{13}C NMR spectrum of **29** (**100 MHz, Chloroform-*d***)

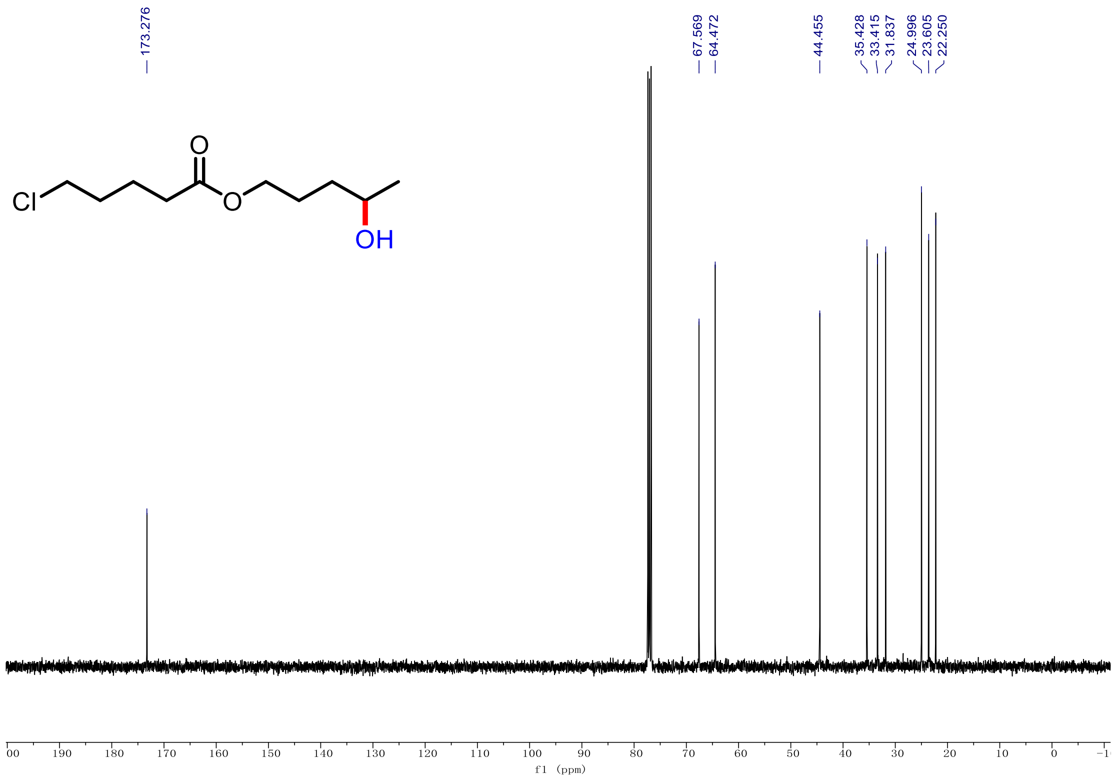

^{1}H NMR spectrum of **30** (**400 MHz, Chloroform-*d***)

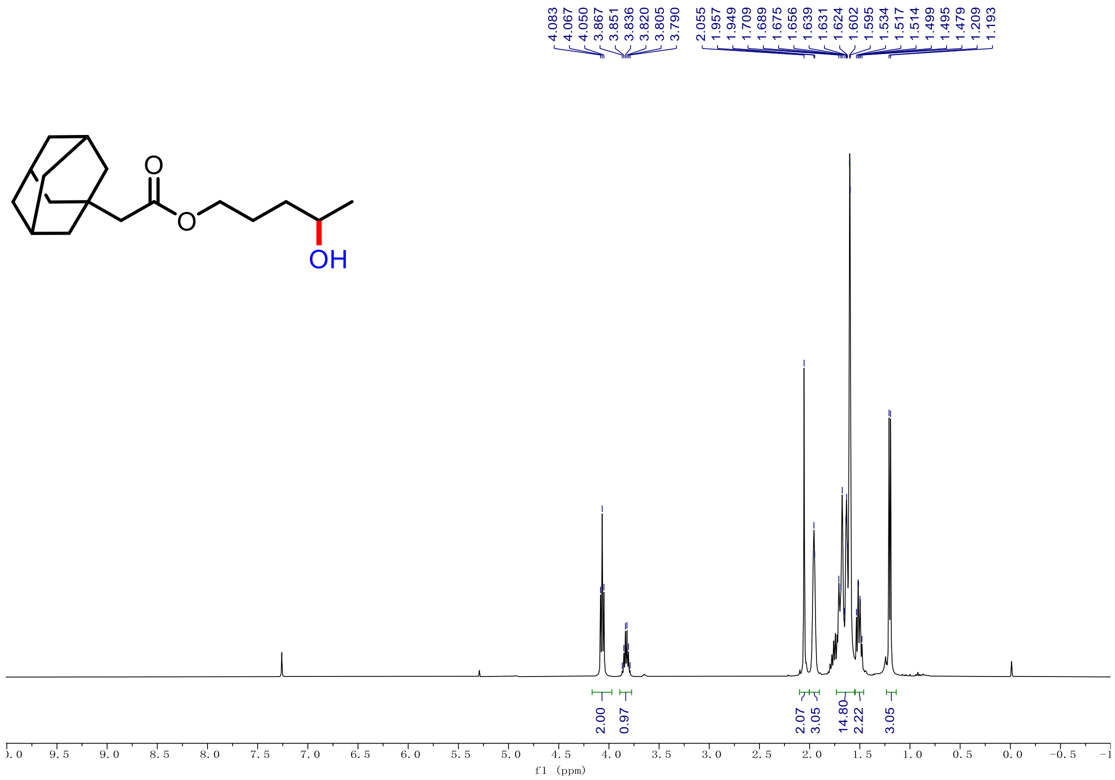

^{13}C NMR spectrum of **30** (**150 MHz, Chloroform-*d***)

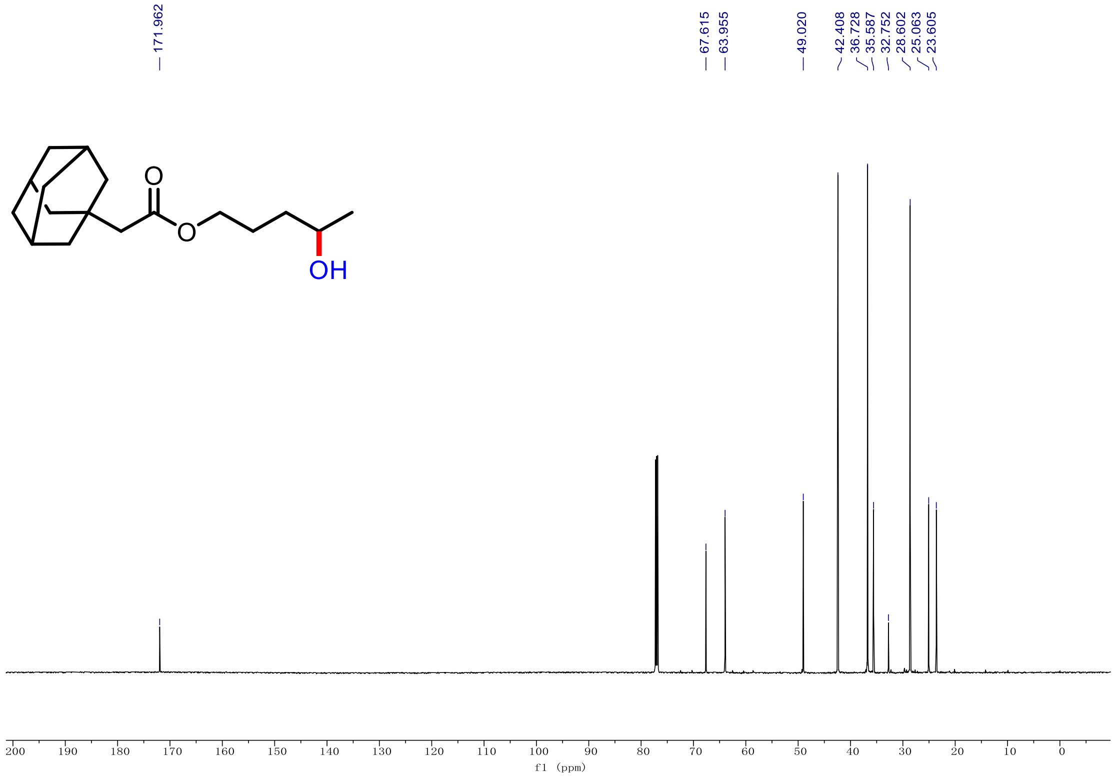

^{1}H NMR spectrum of **31** (**400 MHz, Chloroform-*d***)

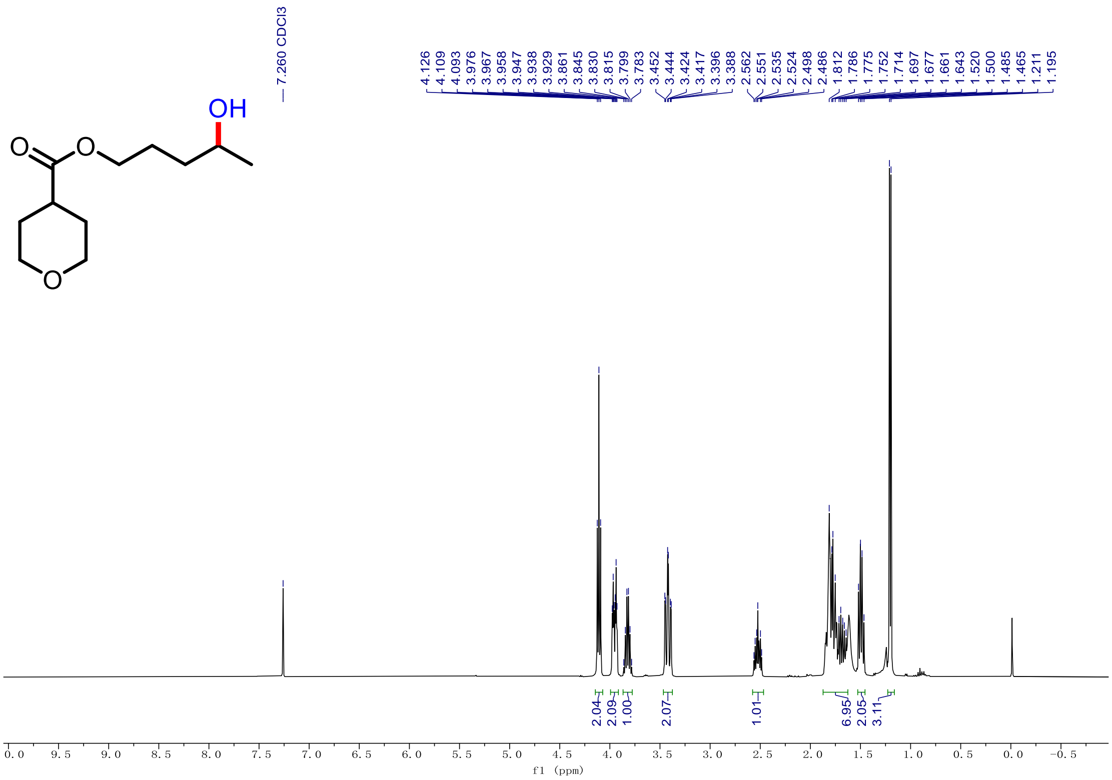

^{13}C NMR spectrum of **31** (**100 MHz, Chloroform-*d***)

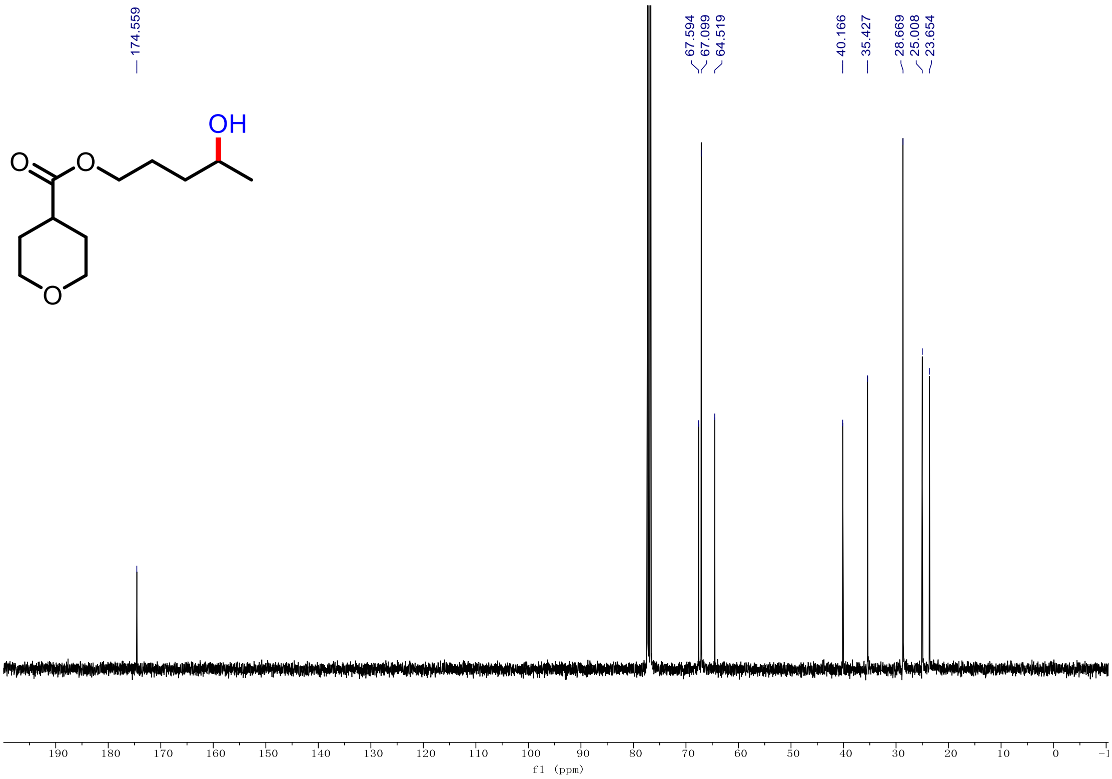

^{1}H NMR spectrum of **32** (**400 MHz, Chloroform-*d***)

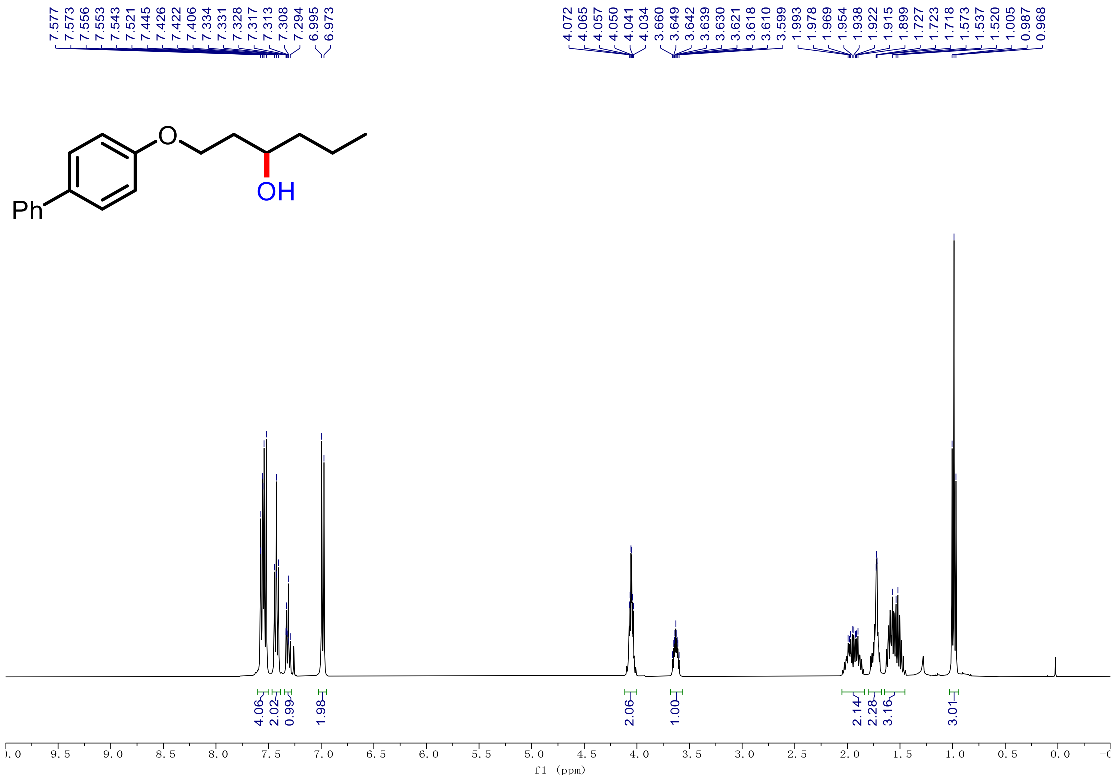

^{13}C NMR spectrum of **32** (**100 MHz, Chloroform-*d***)

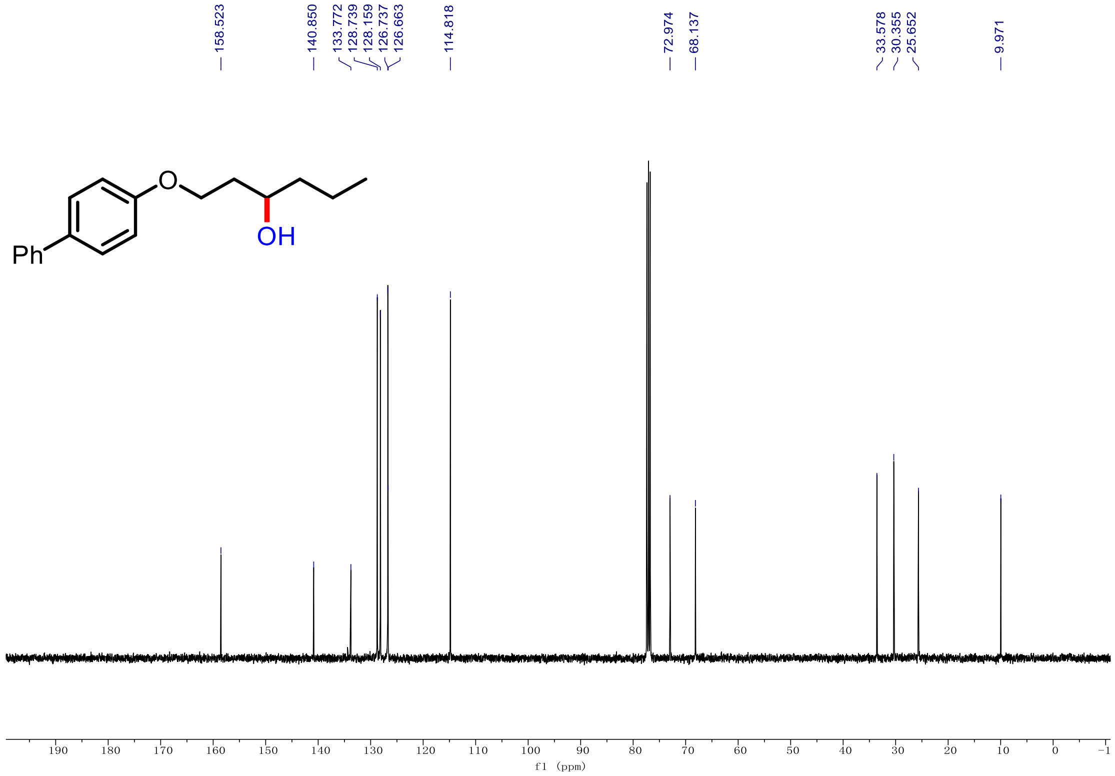

^{1}H NMR spectrum of **33** (**400 MHz, Chloroform-*d***)

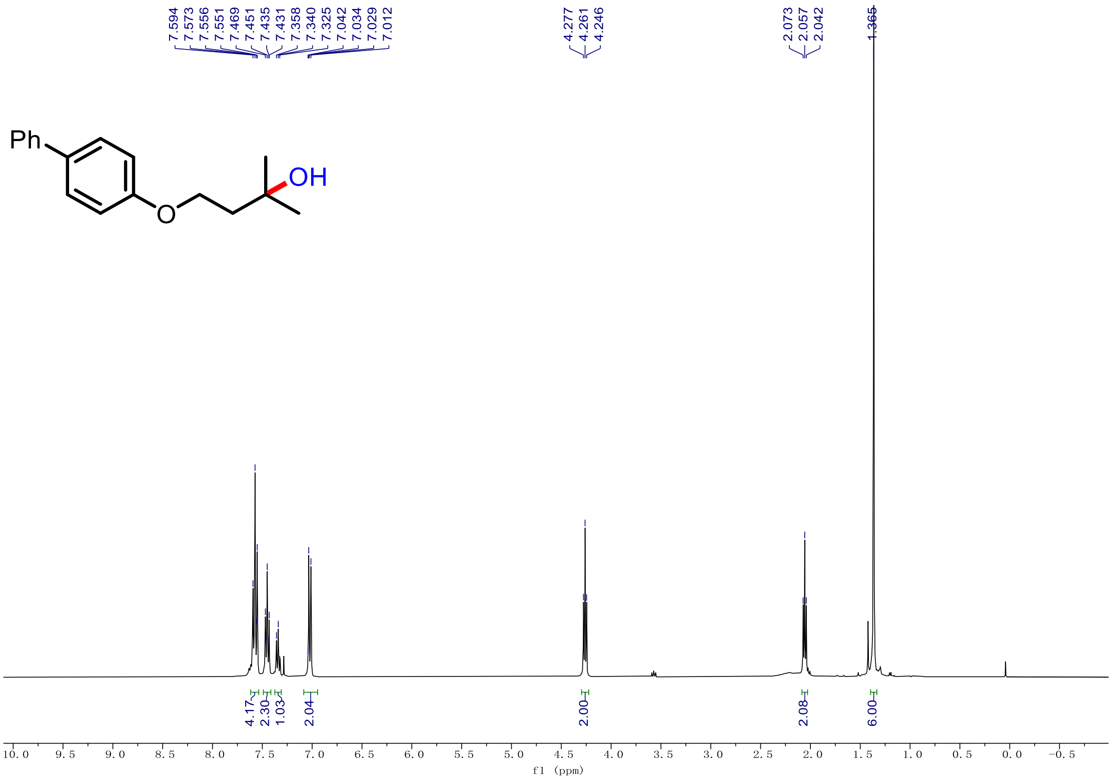

^{13}C NMR spectrum of **33** (**100 MHz, Chloroform-*d***)

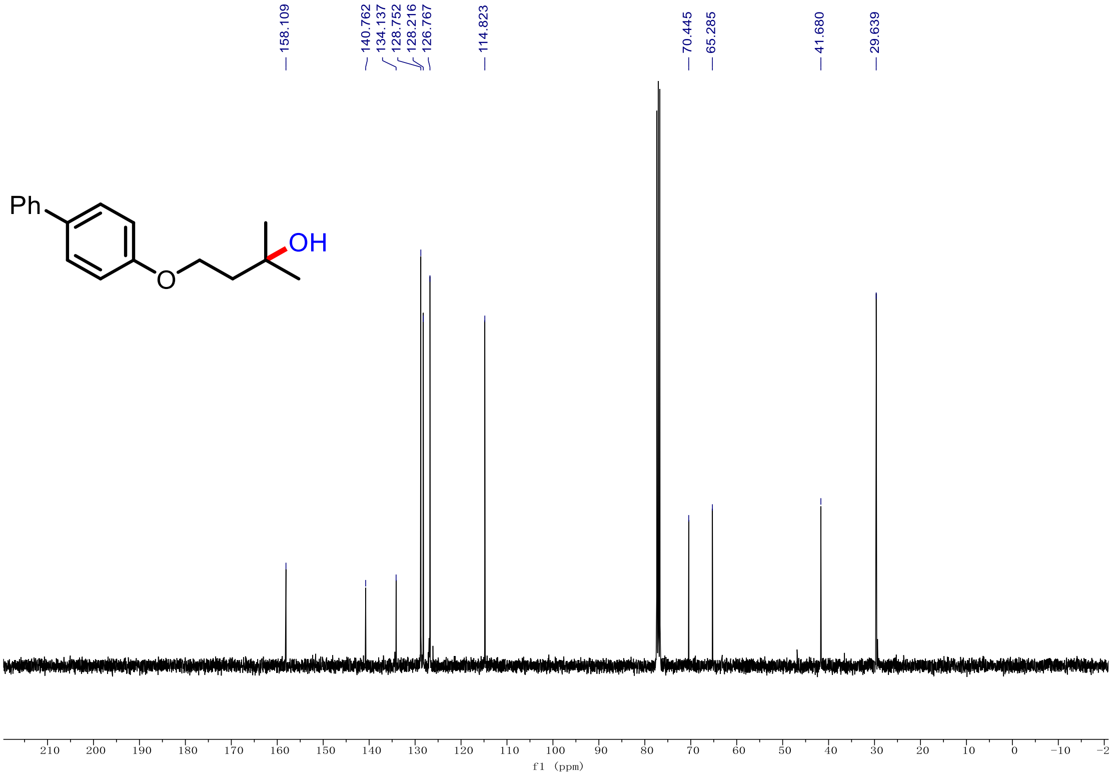

^{1}H NMR spectrum of **34** (**400 MHz, Chloroform-*d***)

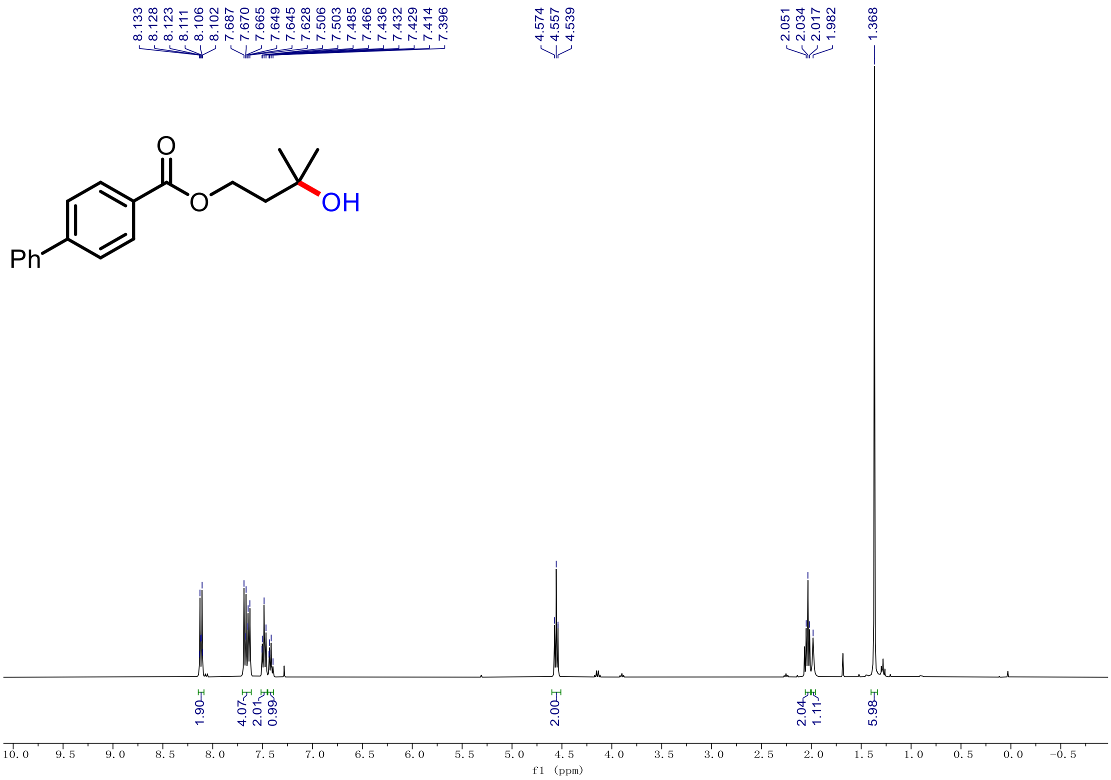

^{13}C NMR spectrum of **34** (**100 MHz, Chloroform-*d***)

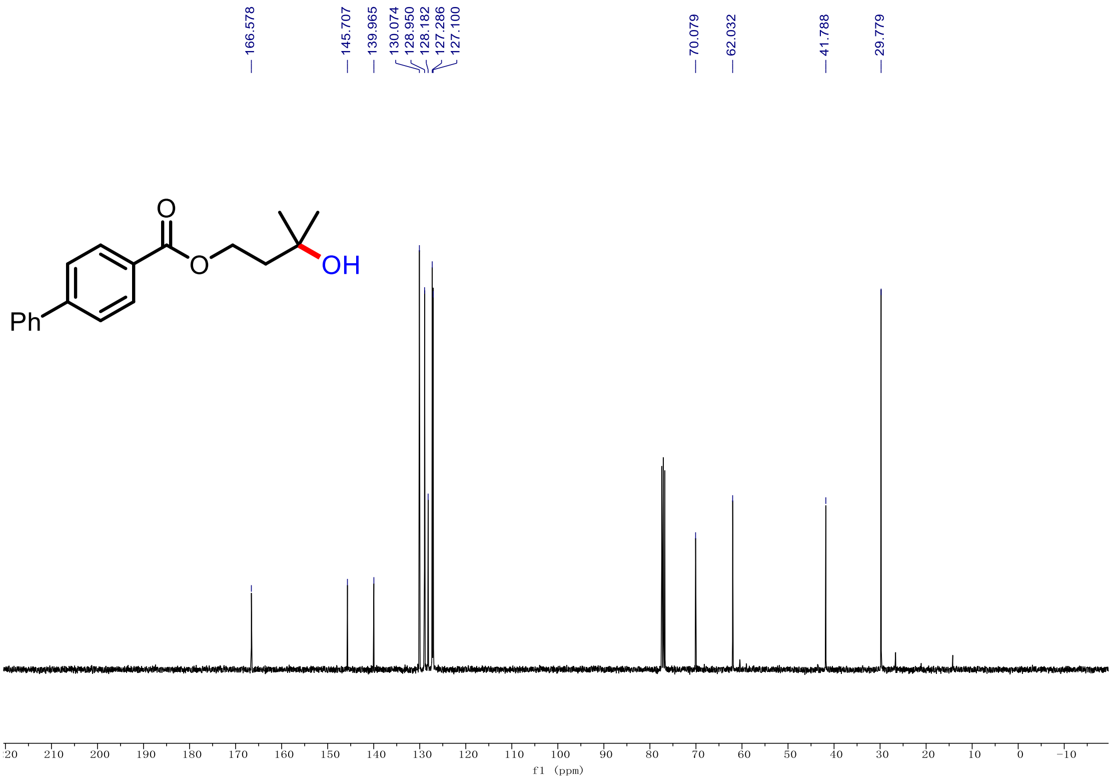

^{1}H NMR spectrum of **35** (**400 MHz, Chloroform-*d***)

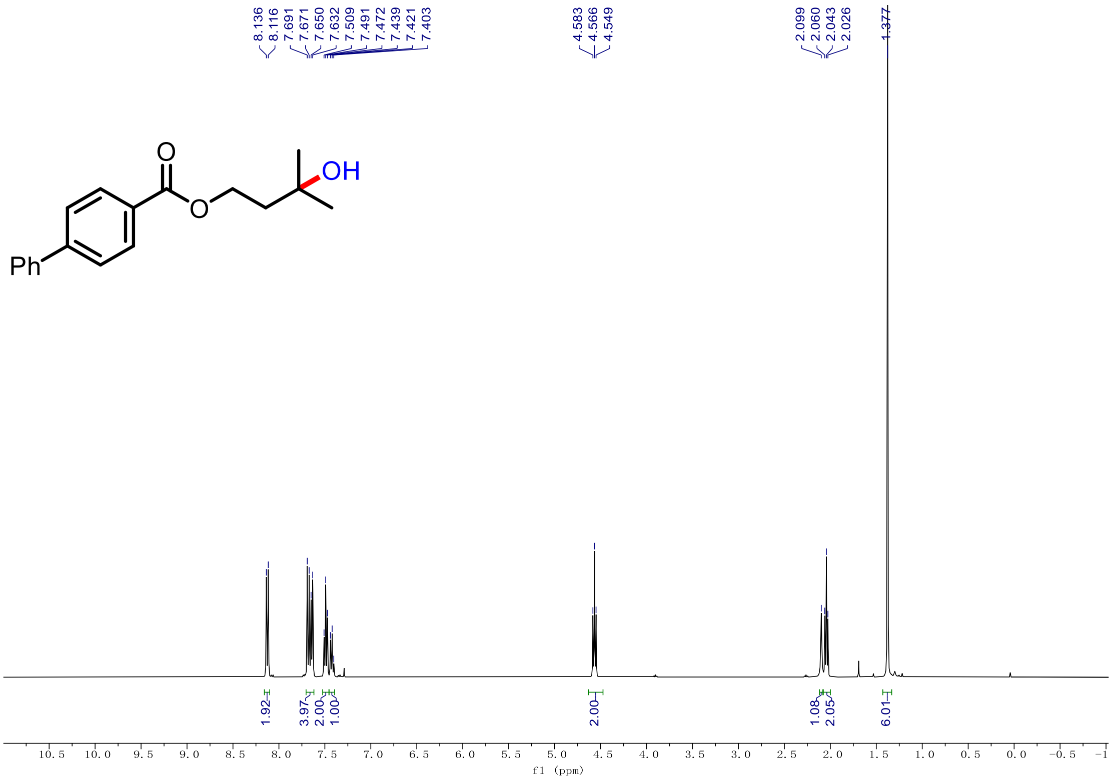

^{13}C NMR spectrum of **35** (**100 MHz, Chloroform-*d***)

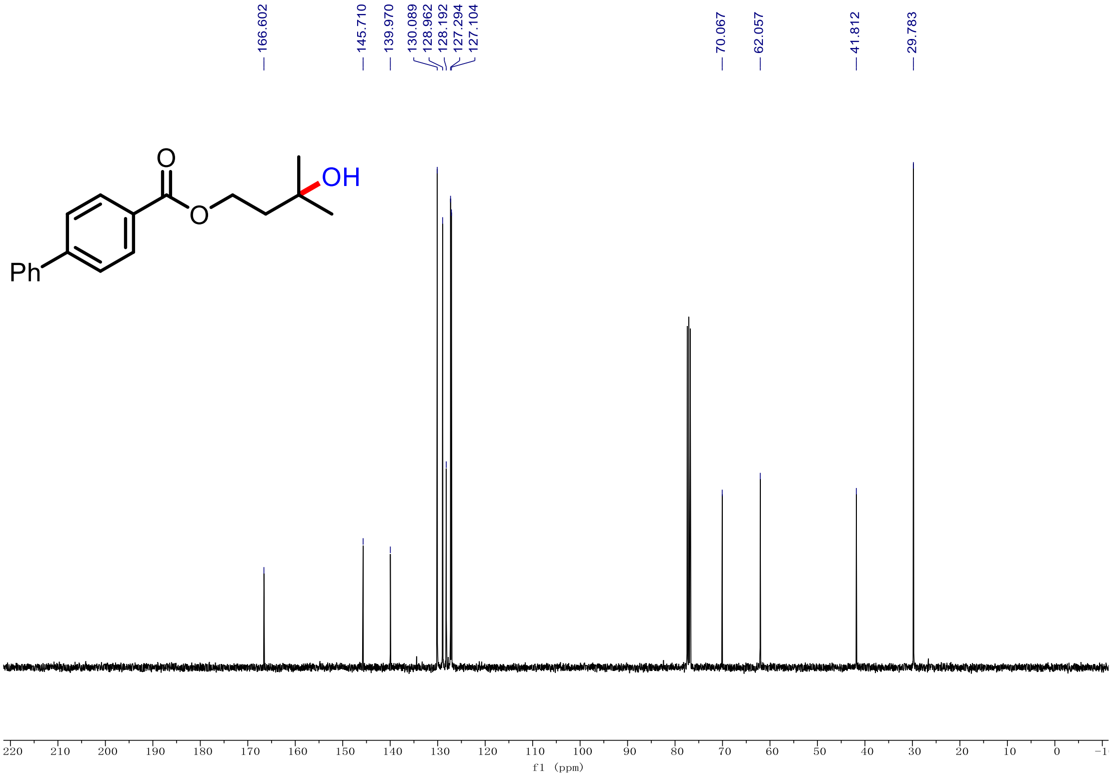

^{1}H NMR spectrum of **36** (**400 MHz, Chloroform-*d***)

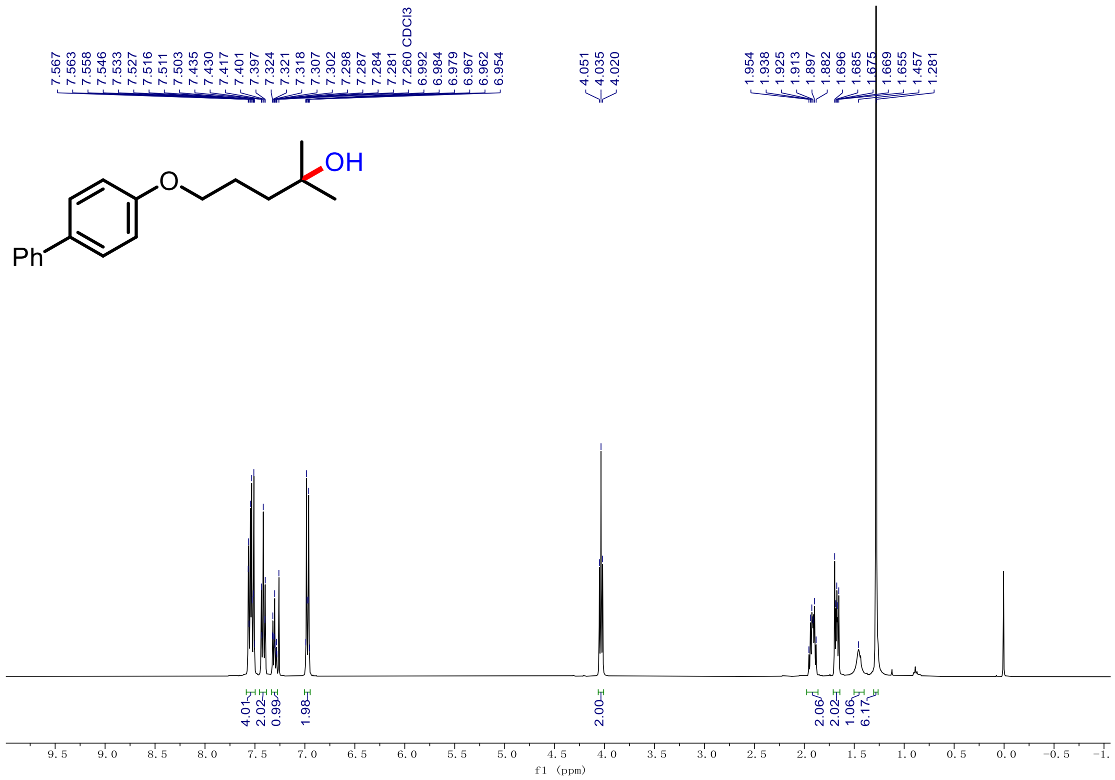

^{13}C NMR spectrum of **36** (**100 MHz, Chloroform-*d***)

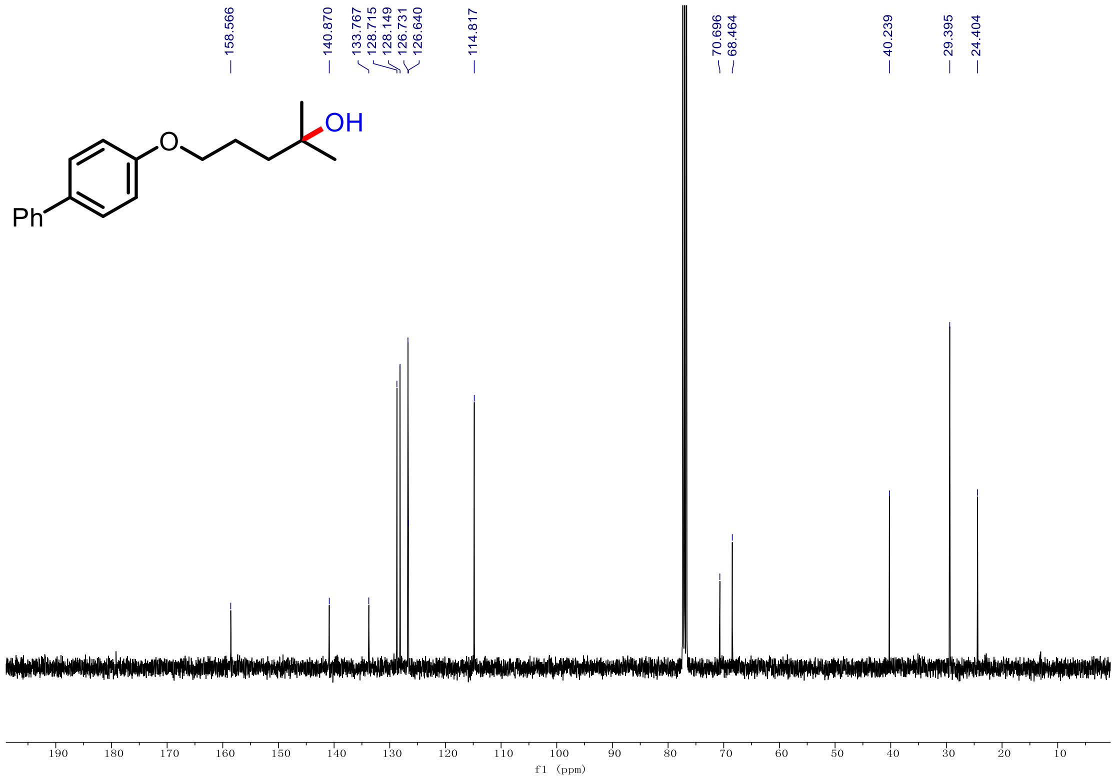

^{1}H NMR spectrum of **37** (**400 MHz, Chloroform-*d***)

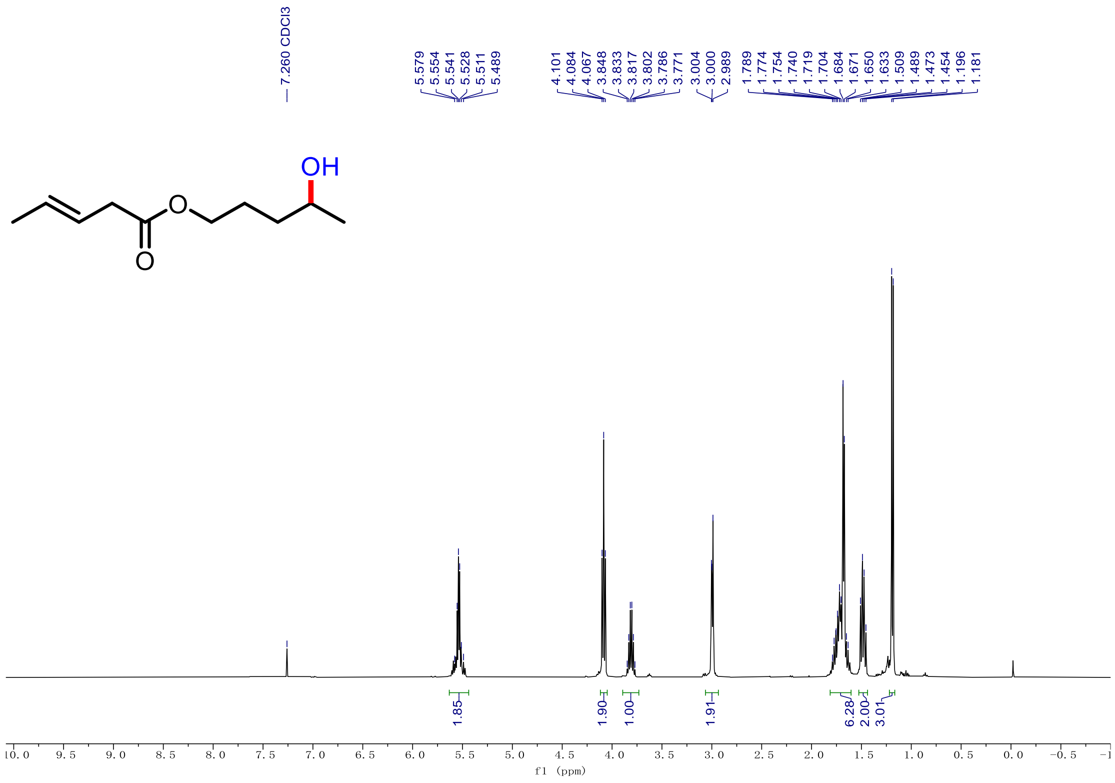

^{13}C NMR spectrum of **37** (**100 MHz, Chloroform-*d***)

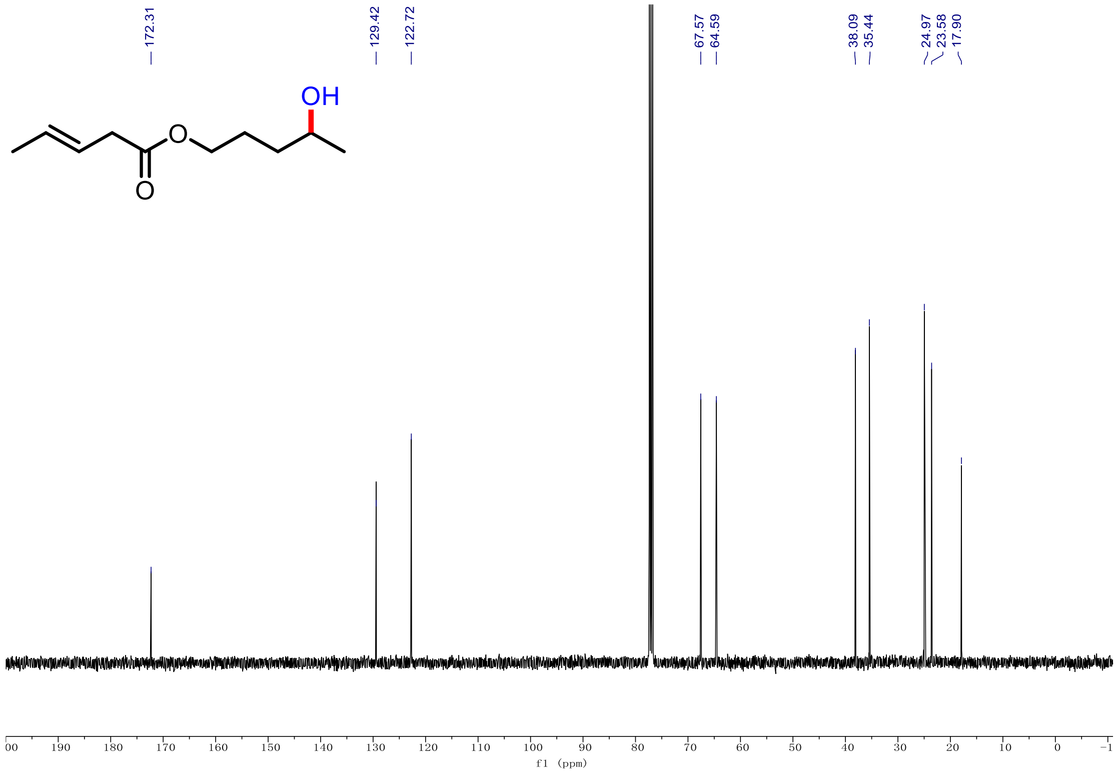

^{1}H NMR spectrum of **38** (**400 MHz, Chloroform-*d***)

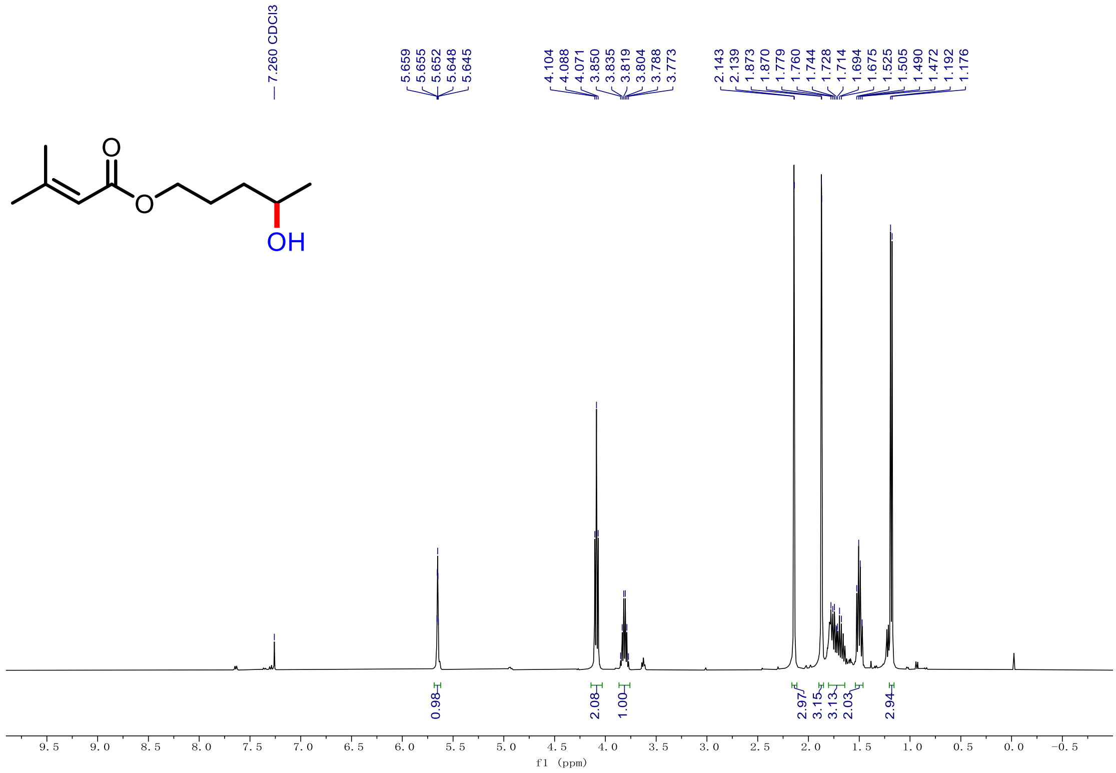

^{13}C NMR spectrum of **38** (**100 MHz, Chloroform-*d***)

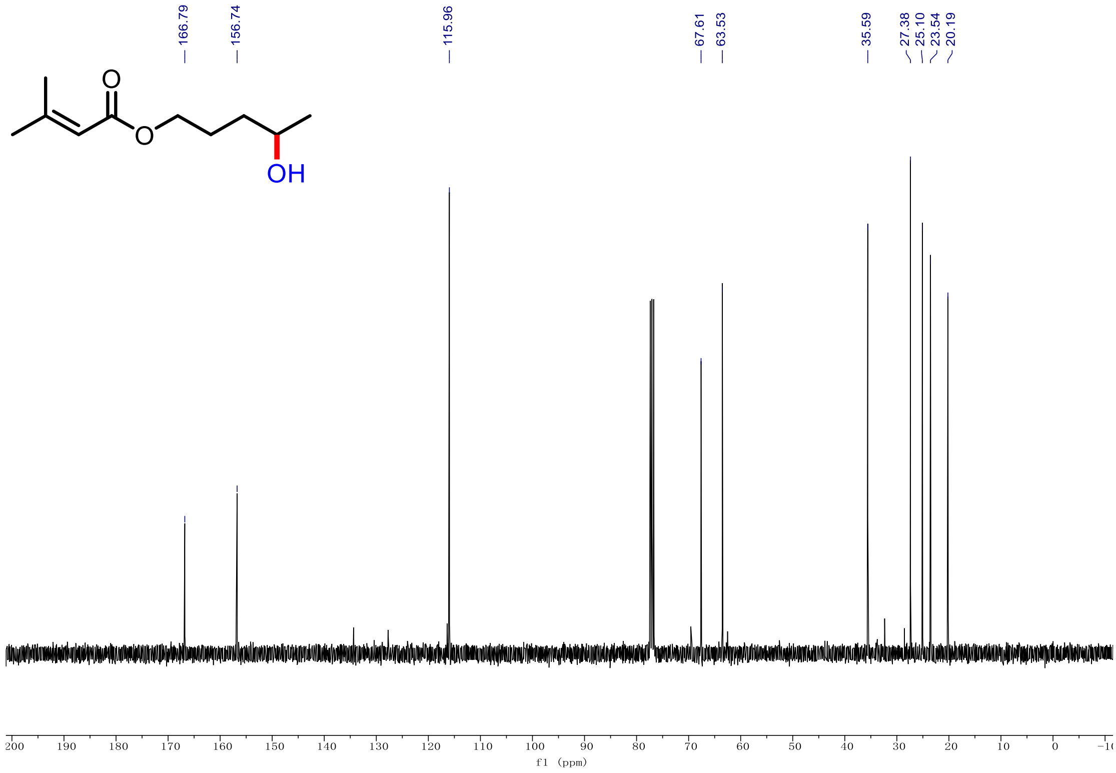

^{1}H NMR spectrum of **39** (**400 MHz, Chloroform-*d***)

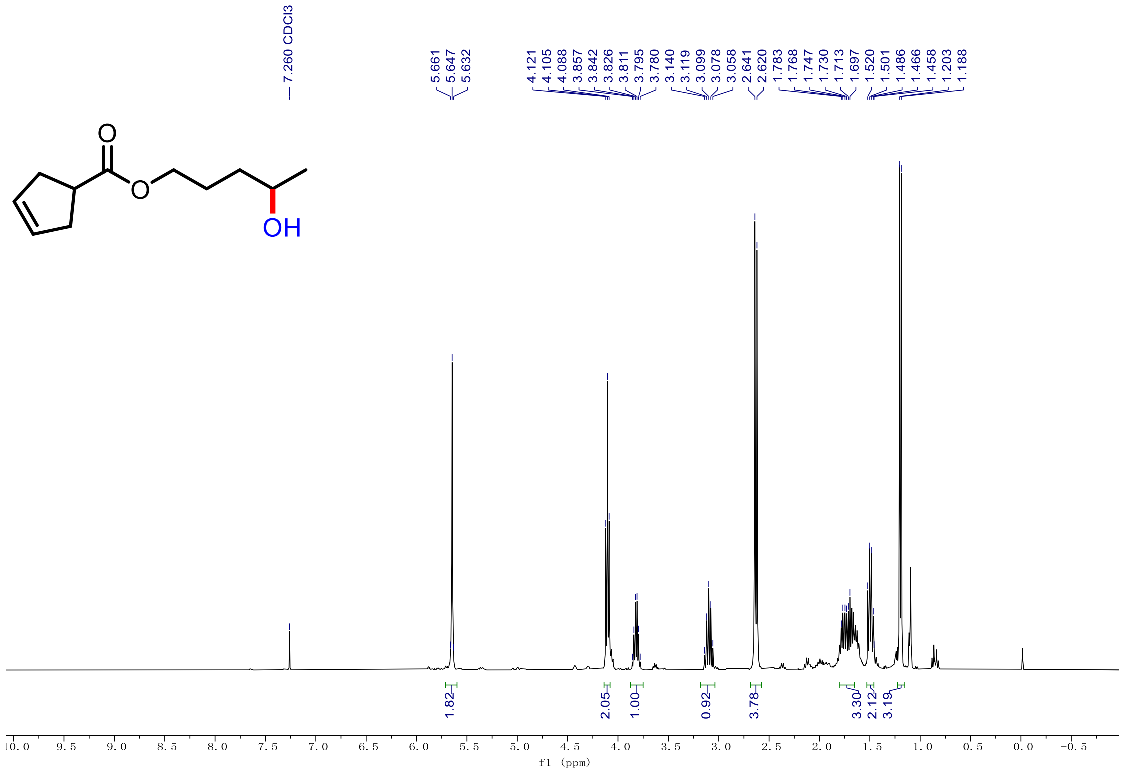

^{13}C NMR spectrum of **39** (**150 MHz, Chloroform-*d***)

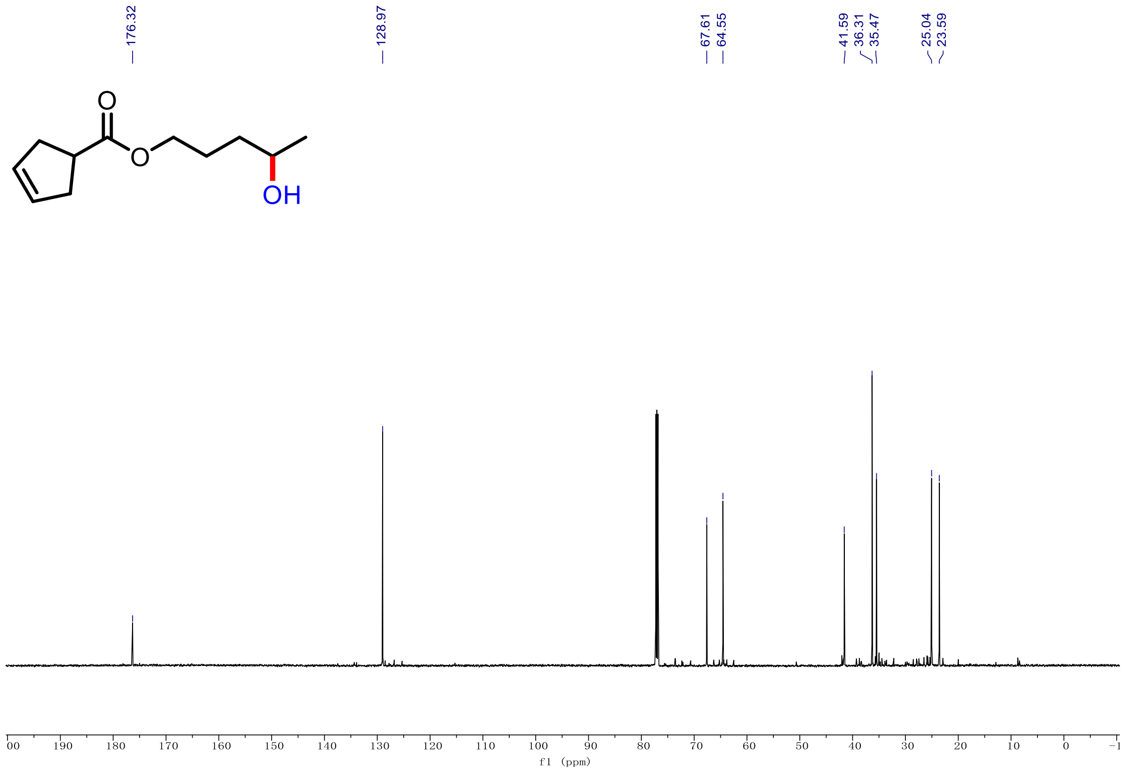

^{1}H NMR spectrum of **40** (**400 MHz, Chloroform-*d***)

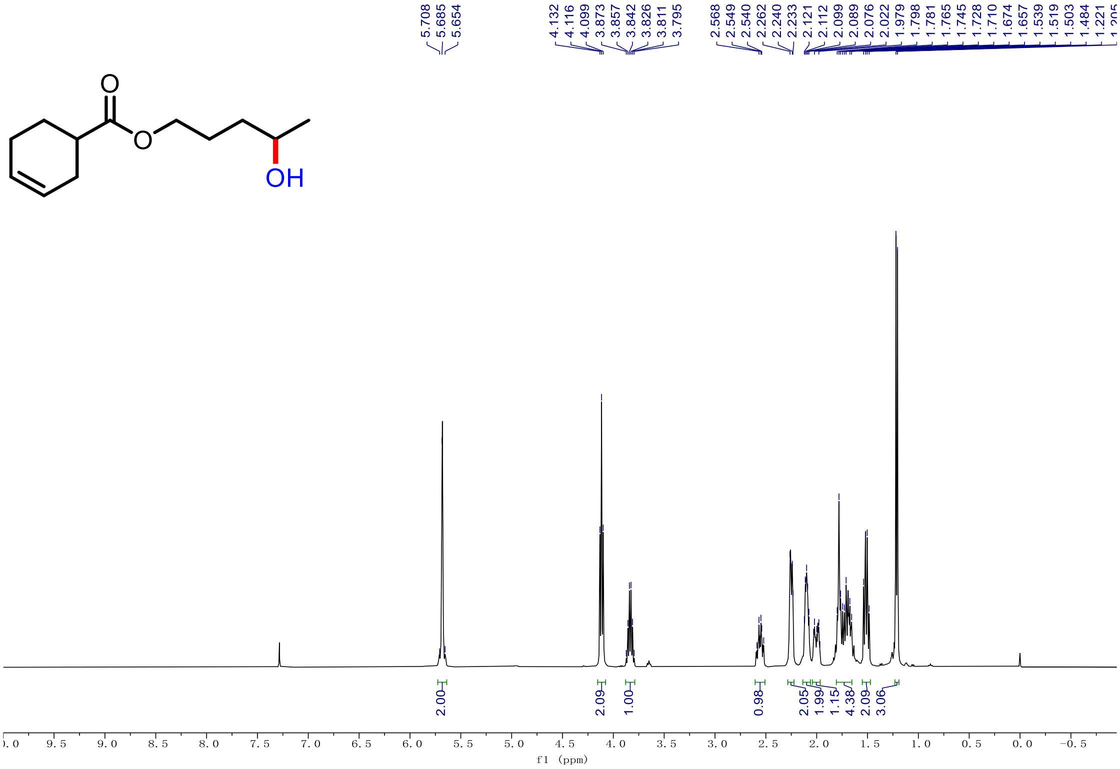

^{13}C NMR spectrum of **40** (**100 MHz, Chloroform-*d***)

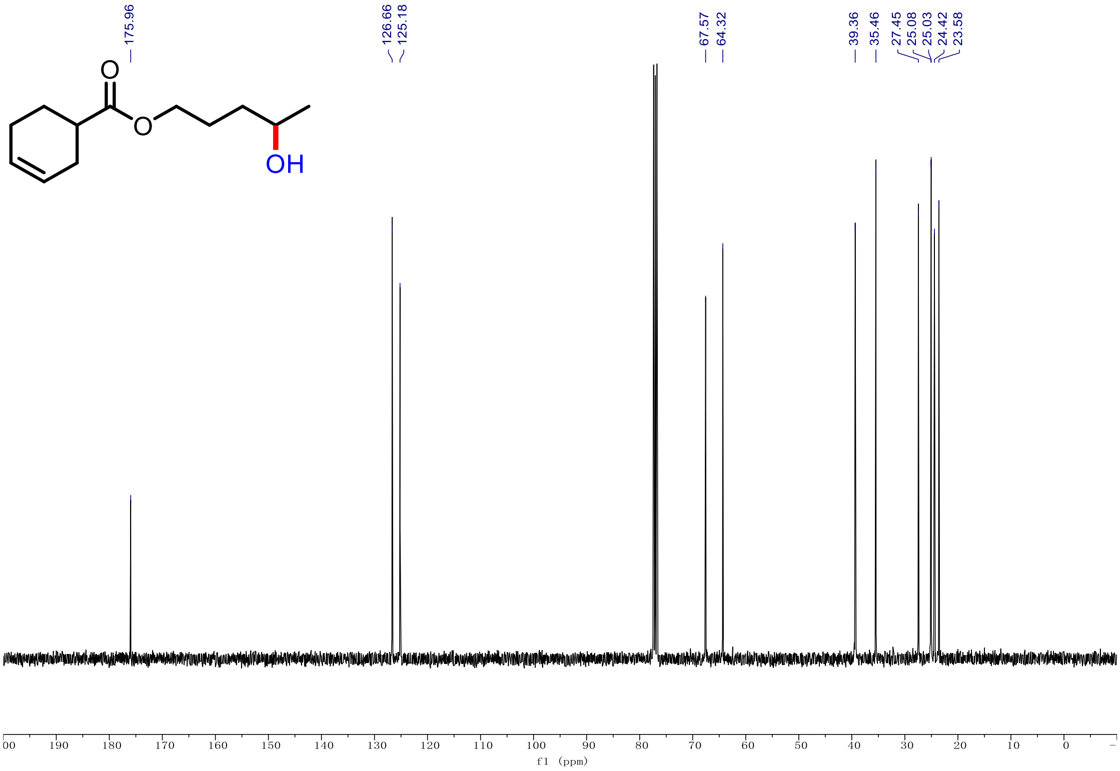

^{1}H NMR spectrum of **41** (**400 MHz, Chloroform-*d***)

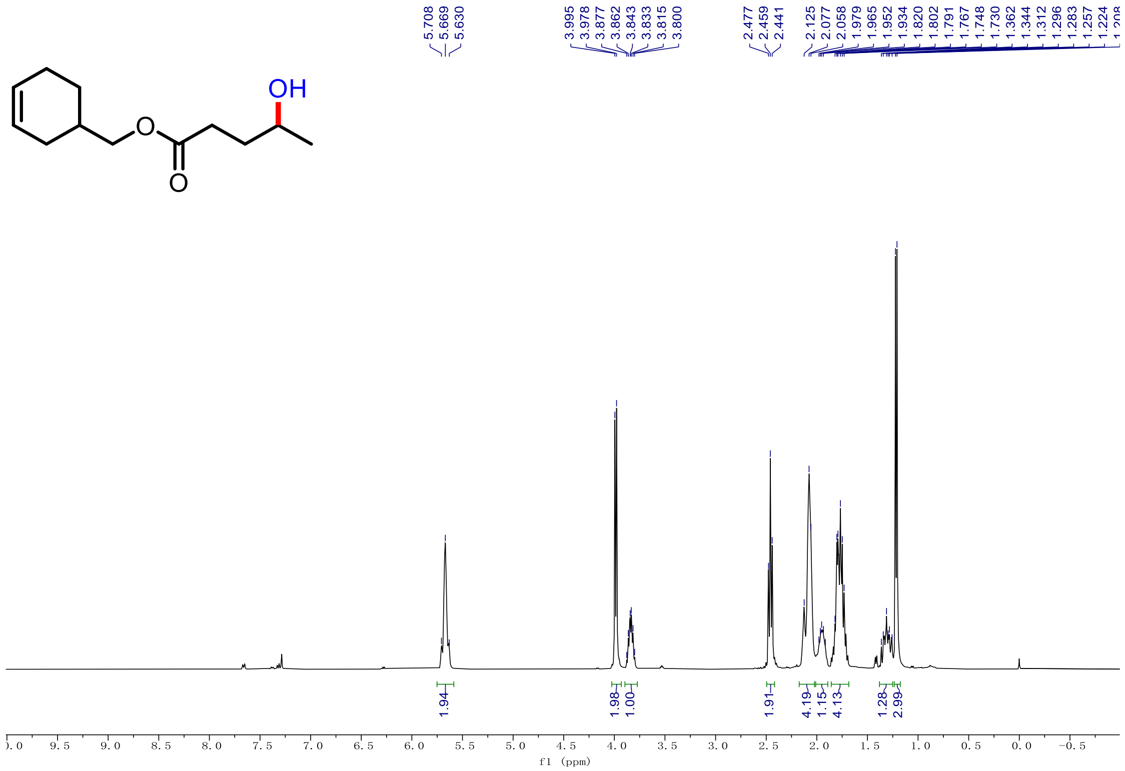

^{13}C NMR spectrum of **41** (**400 MHz, Chloroform-*d***)

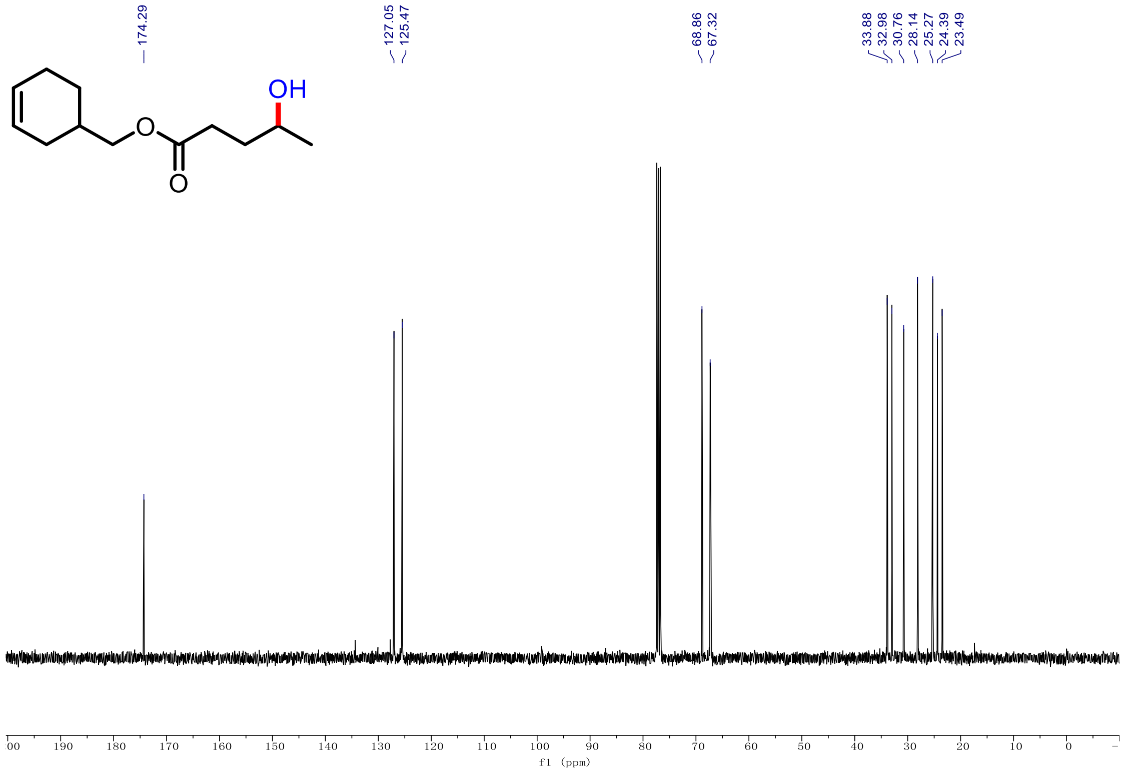

^{1}H NMR spectrum of **42** (**400 MHz, Chloroform-*d***)

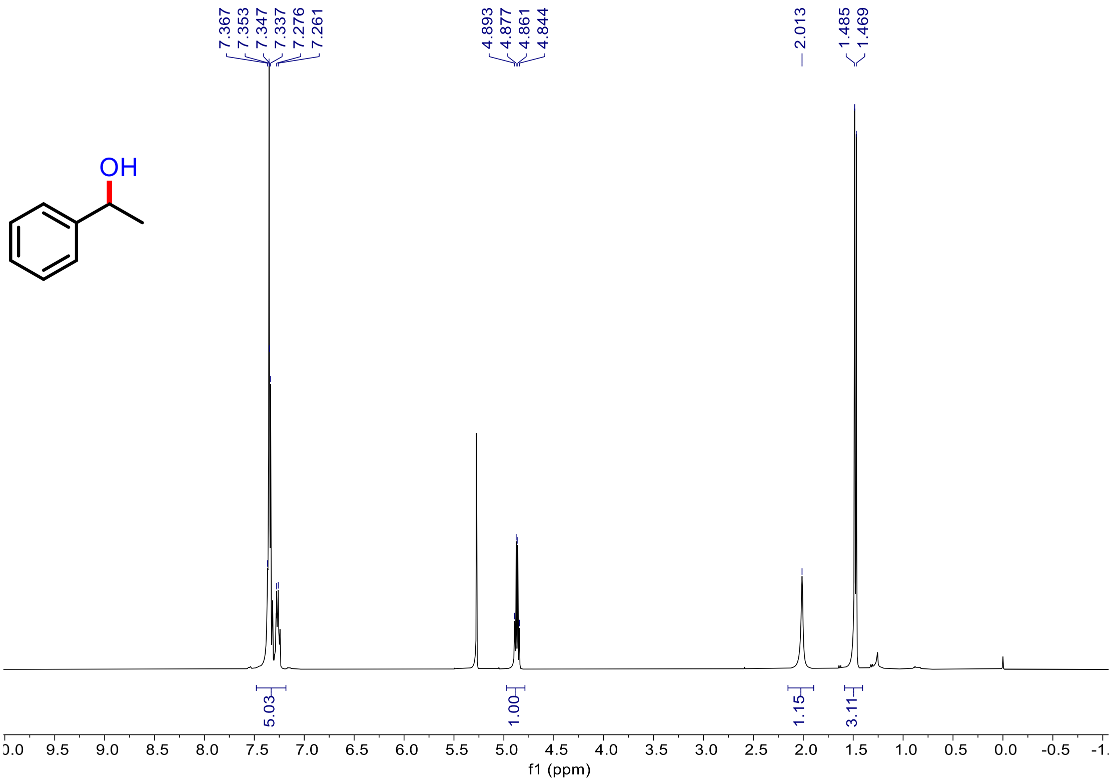

^{13}C NMR spectrum of **42** (**100 MHz, Chloroform-*d***)

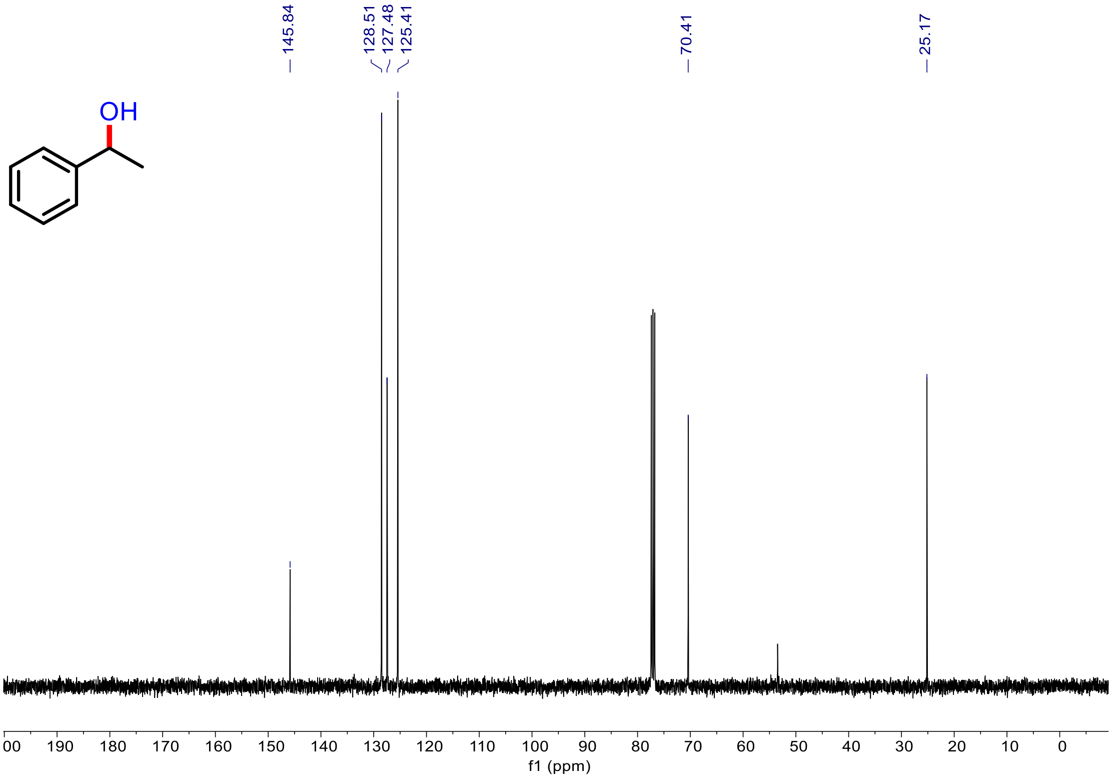

^{1}H NMR spectrum of **43** (**400 MHz, Chloroform-*d***)

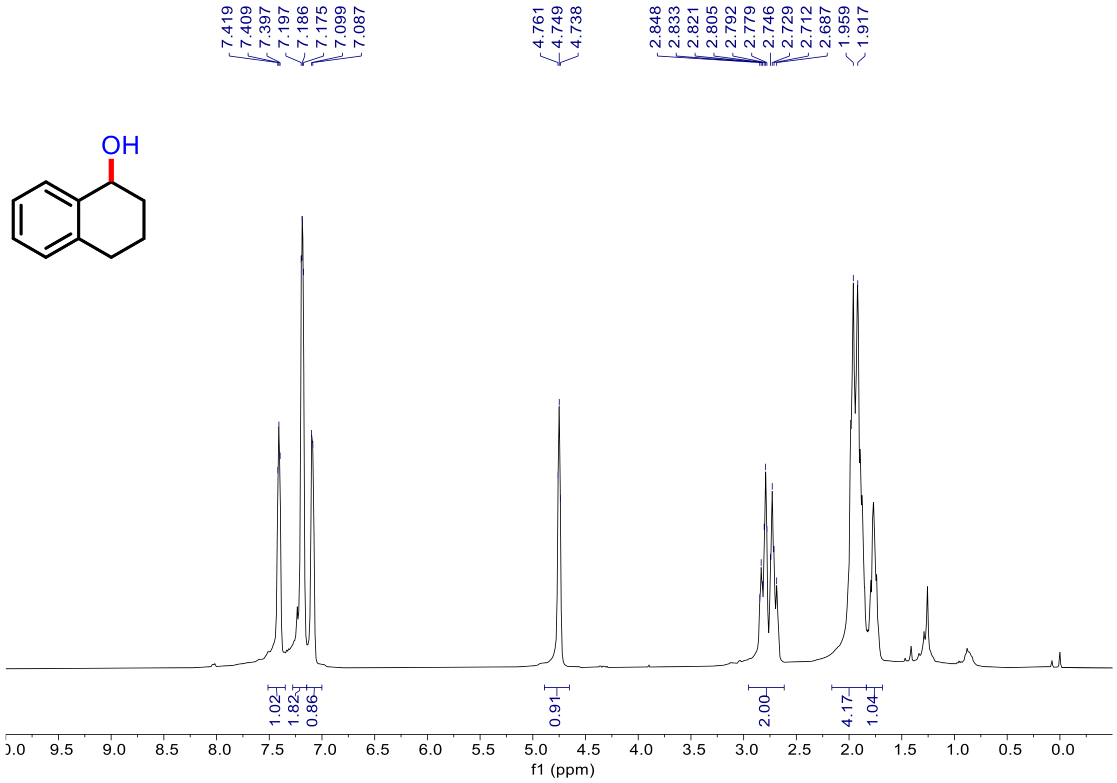

^{13}C NMR spectrum of **43** (**100 MHz, Chloroform-*d***)

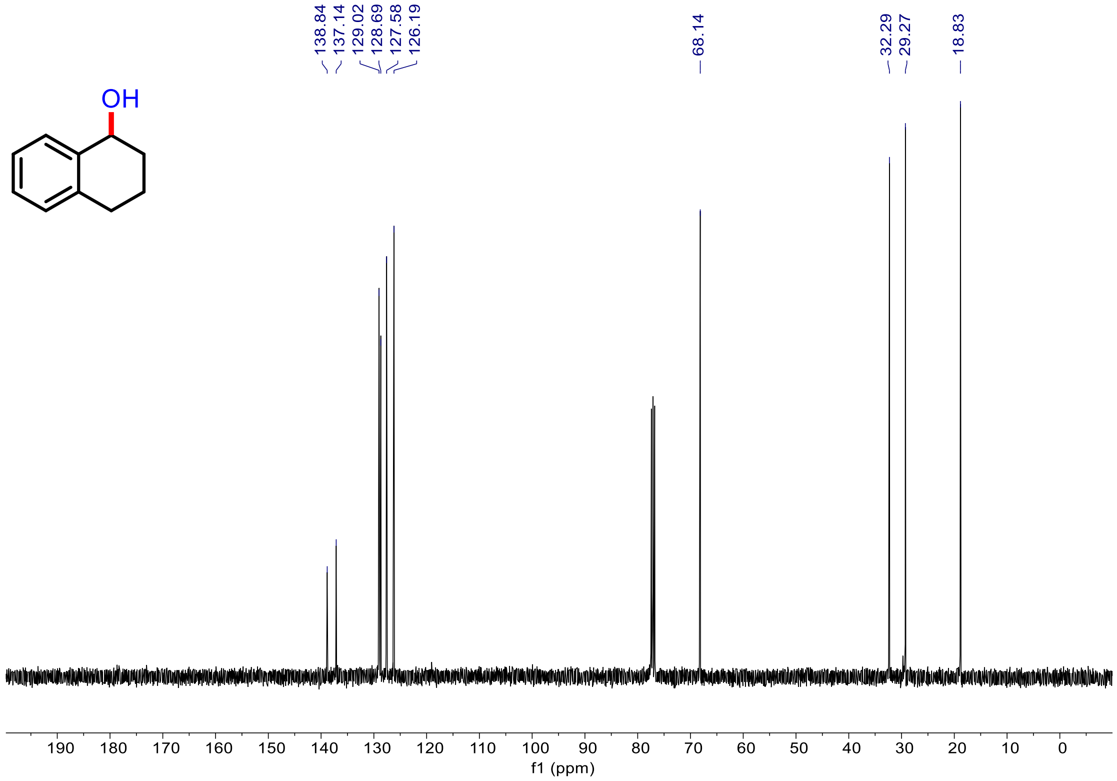

^{1}H NMR spectrum of **44** (**400 MHz, Chloroform-*d***)

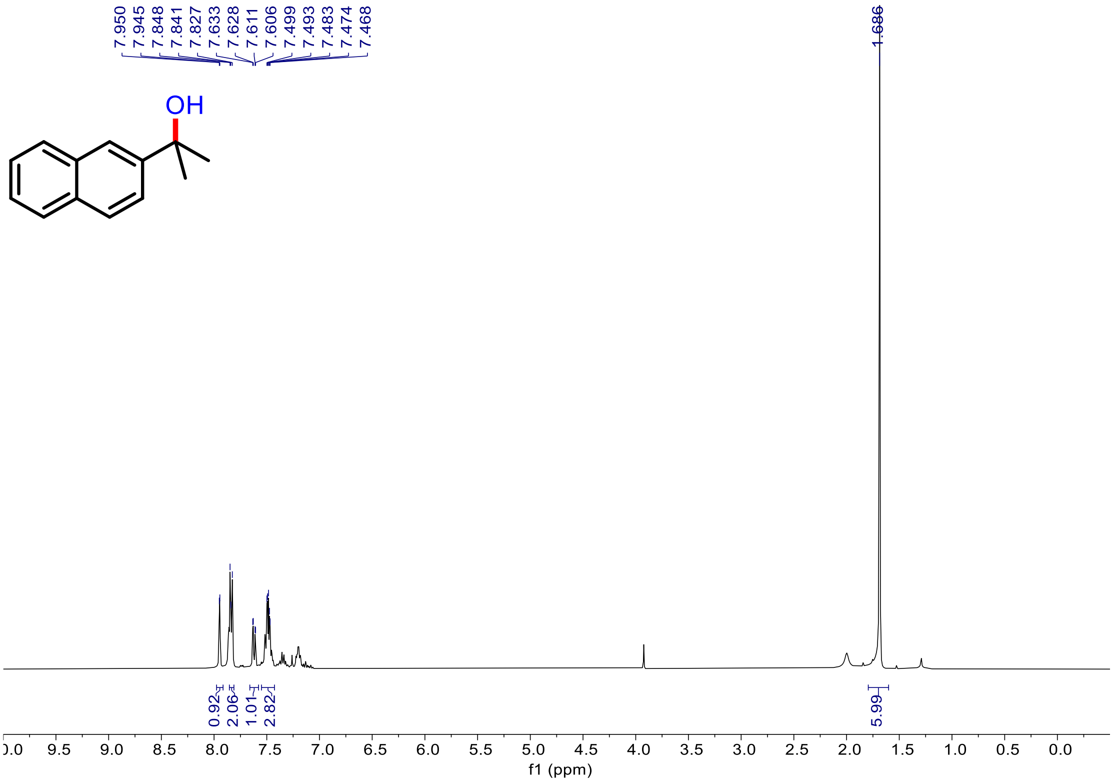

^{13}C NMR spectrum of **44** (**100 MHz, Chloroform-*d***)

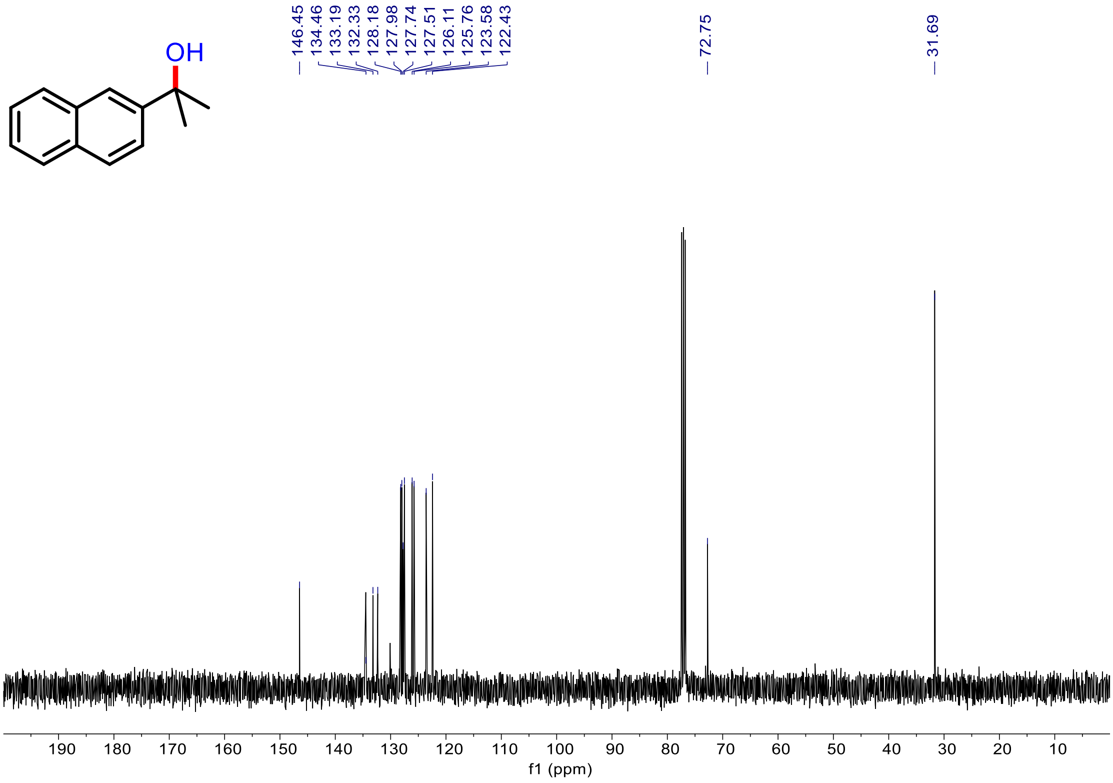

^{1}H NMR spectrum of **45** (**400 MHz, Chloroform-*d***)

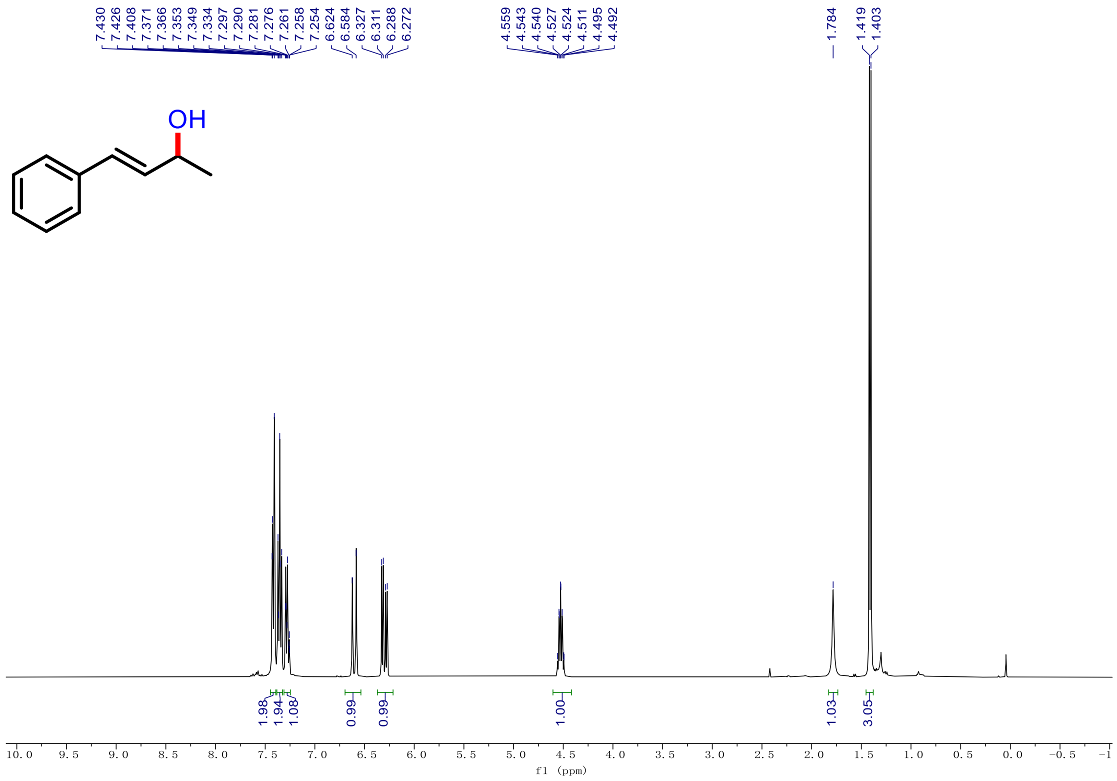

^{13}C NMR spectrum of **45** (**100 MHz, Chloroform-*d***)

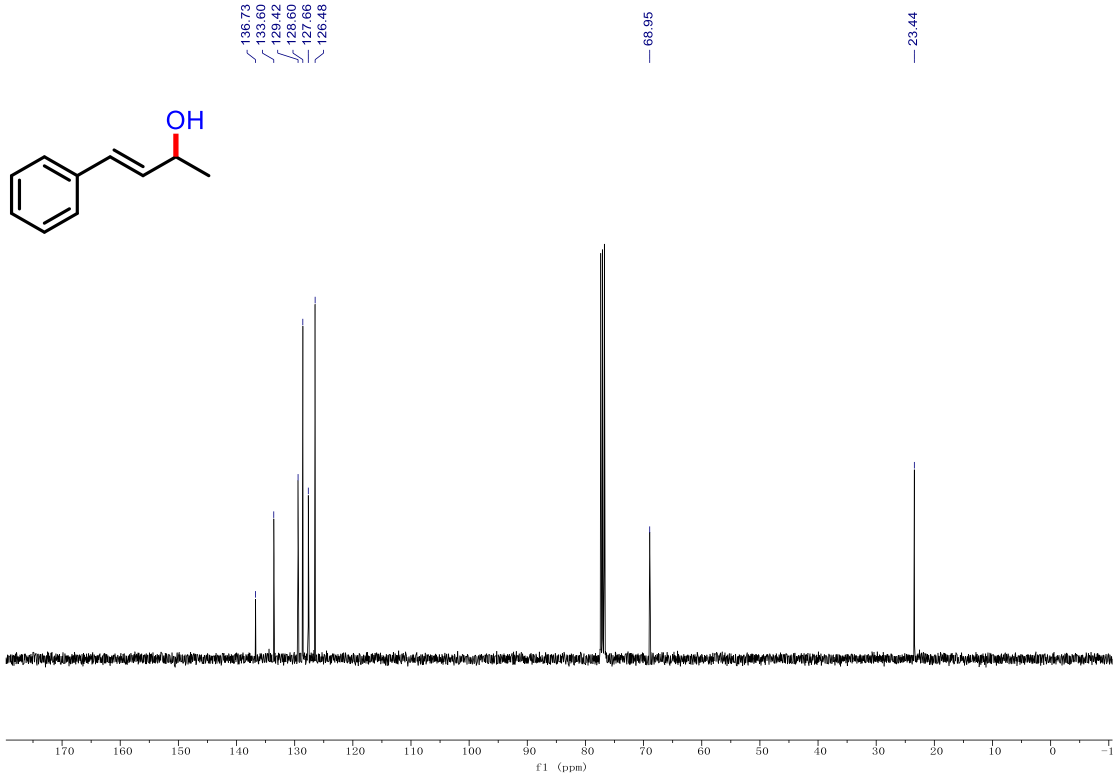

^{1}H NMR spectrum of **46** (**400 MHz, Chloroform-*d***)

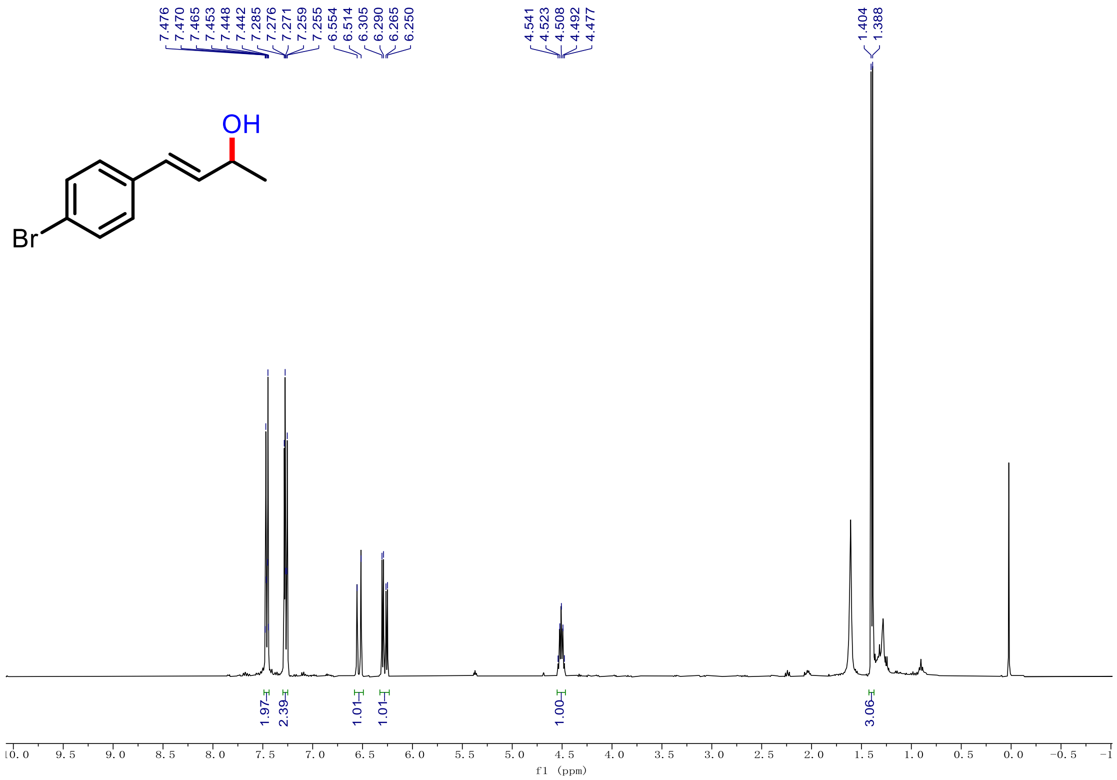

^{13}C NMR spectrum of **46** (**150 MHz, Chloroform-*d***)

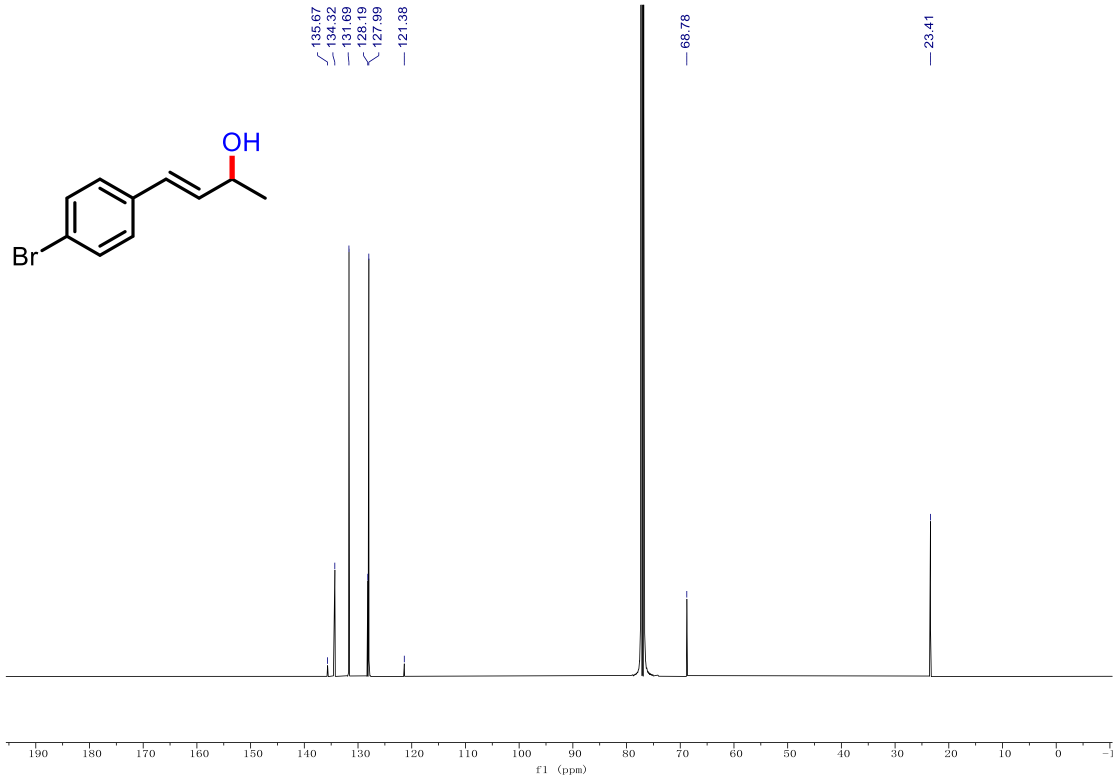

^{1}H NMR spectrum of **47** (**400 MHz, Chloroform-*d***)

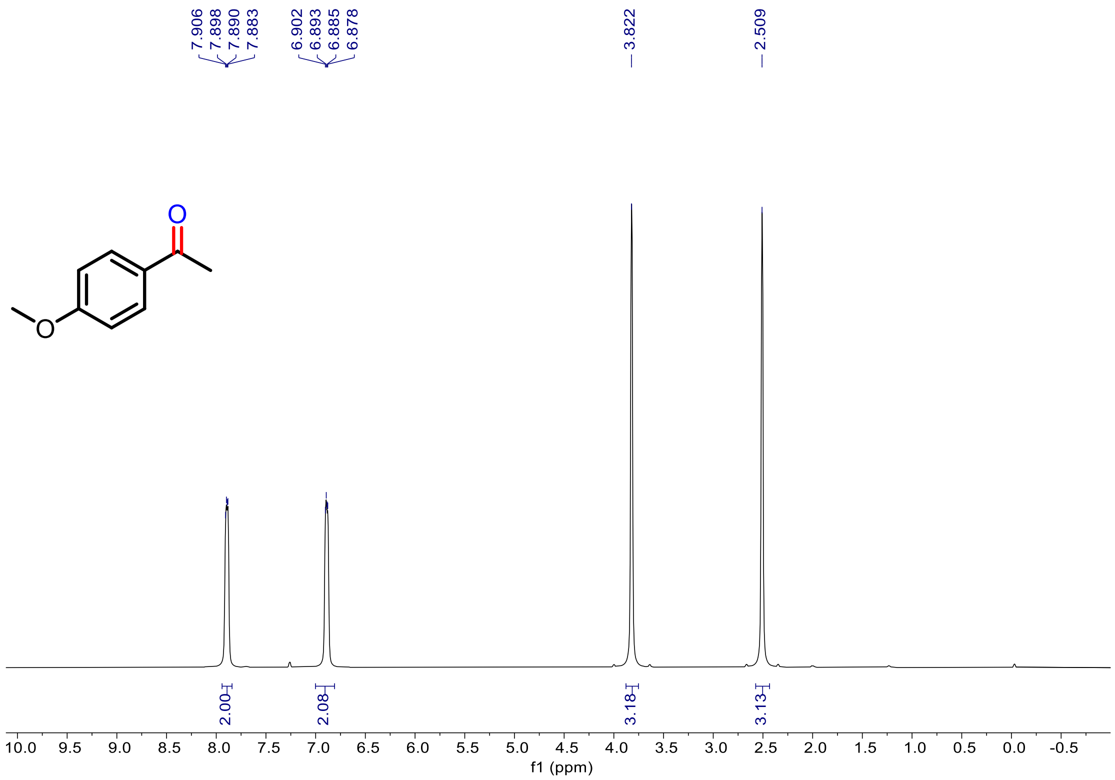

^{13}C NMR spectrum of **47** (**100 MHz, Chloroform-*d***)

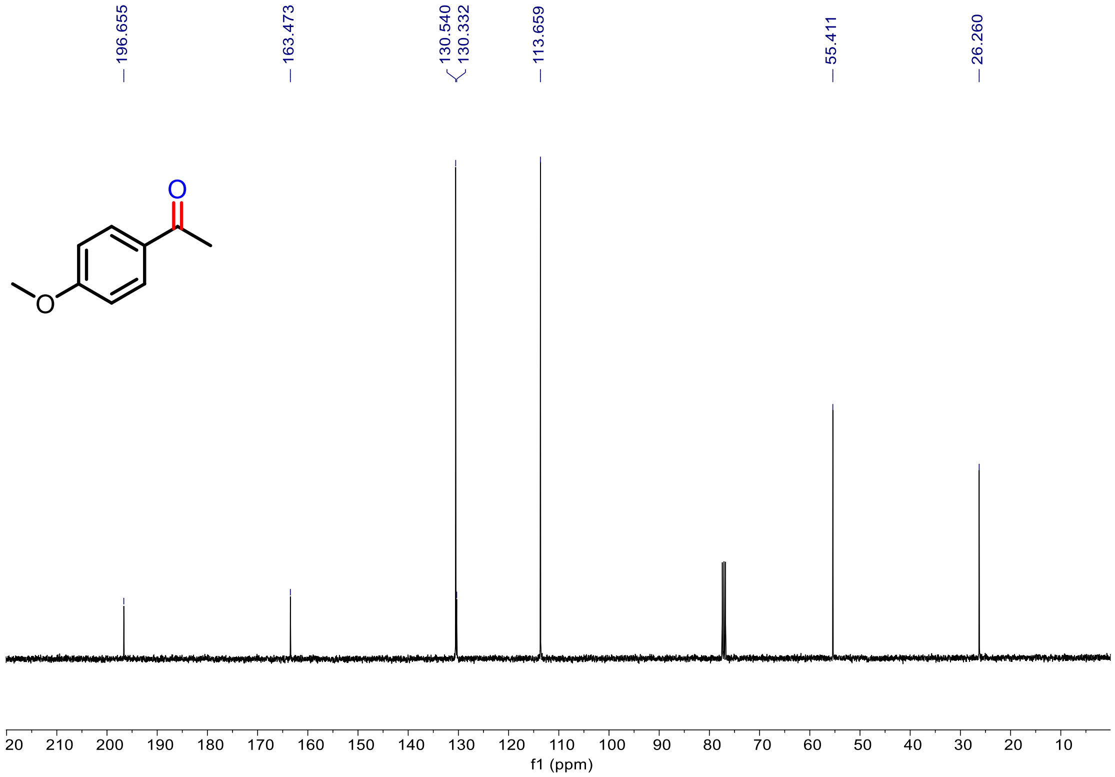

^{1}H NMR spectrum of **48** (**400 MHz, Chloroform-*d***)

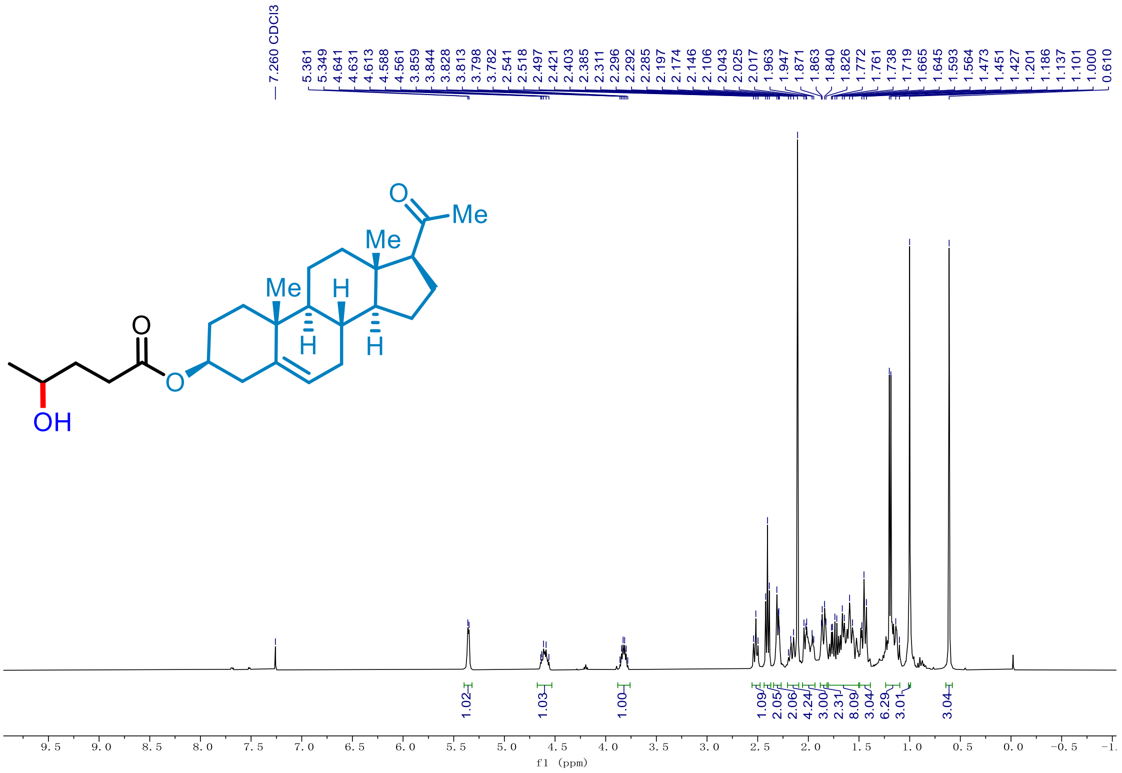

^{13}C NMR spectrum of **48** (**100 MHz, Chloroform-*d***)

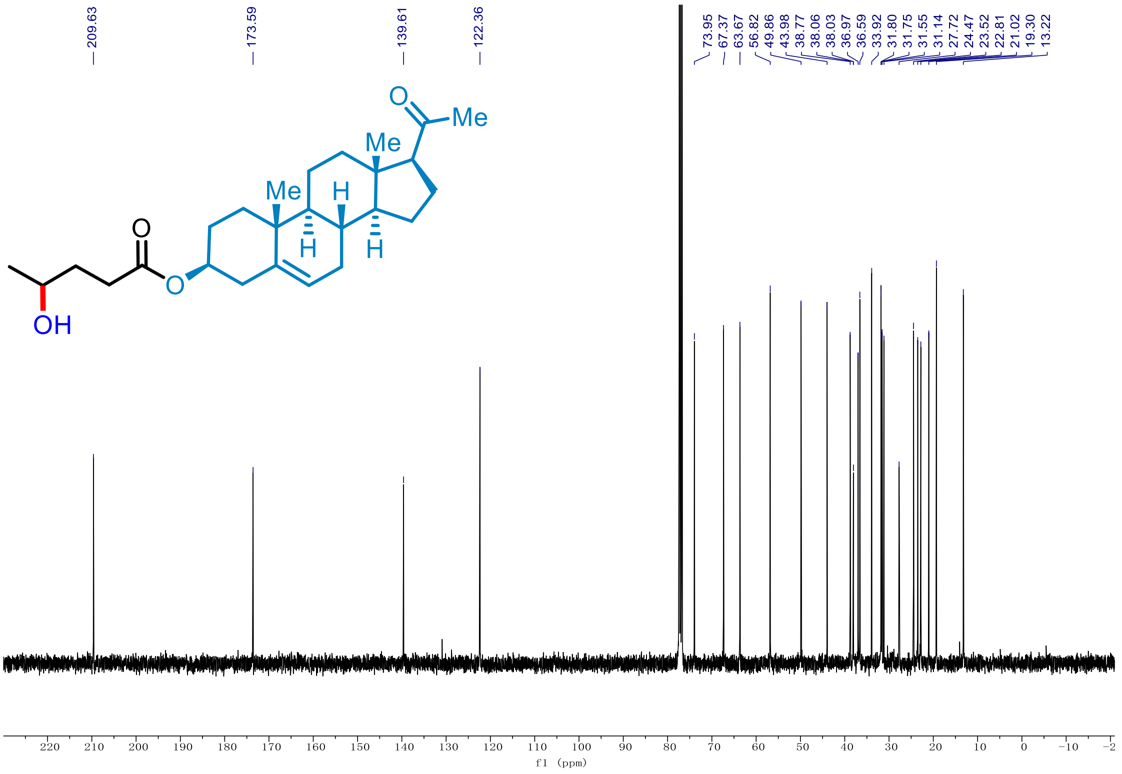

^{1}H NMR spectrum of **49** (**400 MHz, Chloroform-*d***)

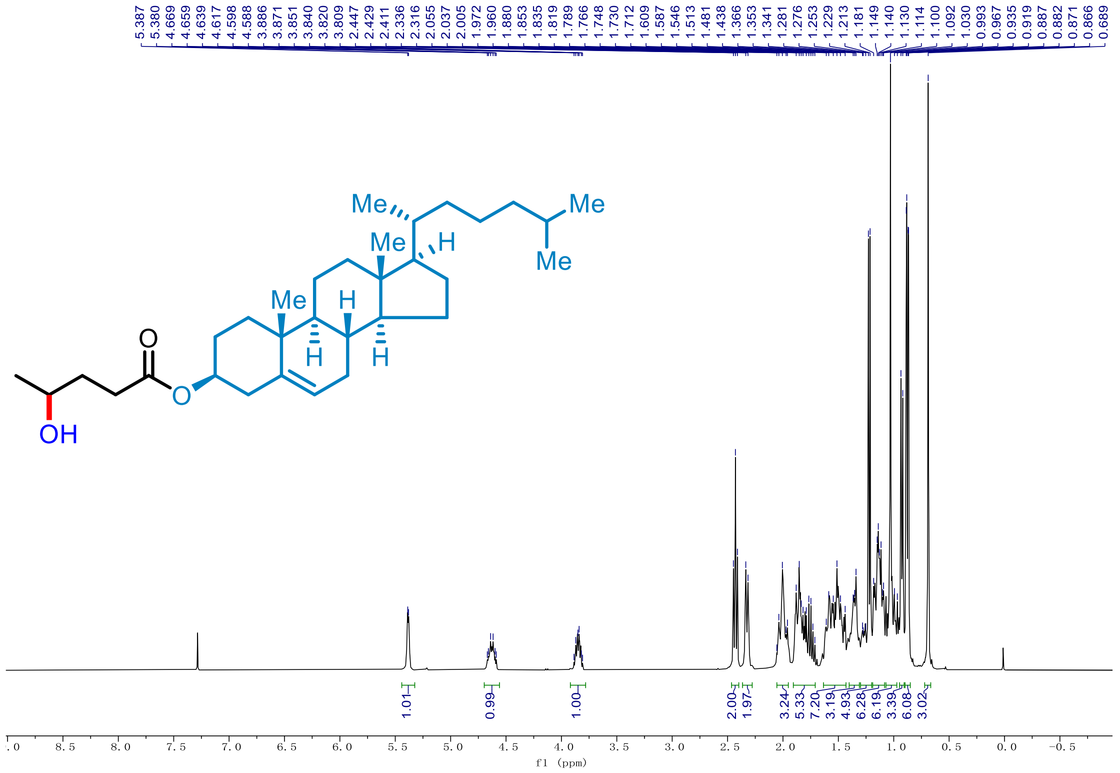

^{13}C NMR spectrum of **49** (**100 MHz, Chloroform-*d***)

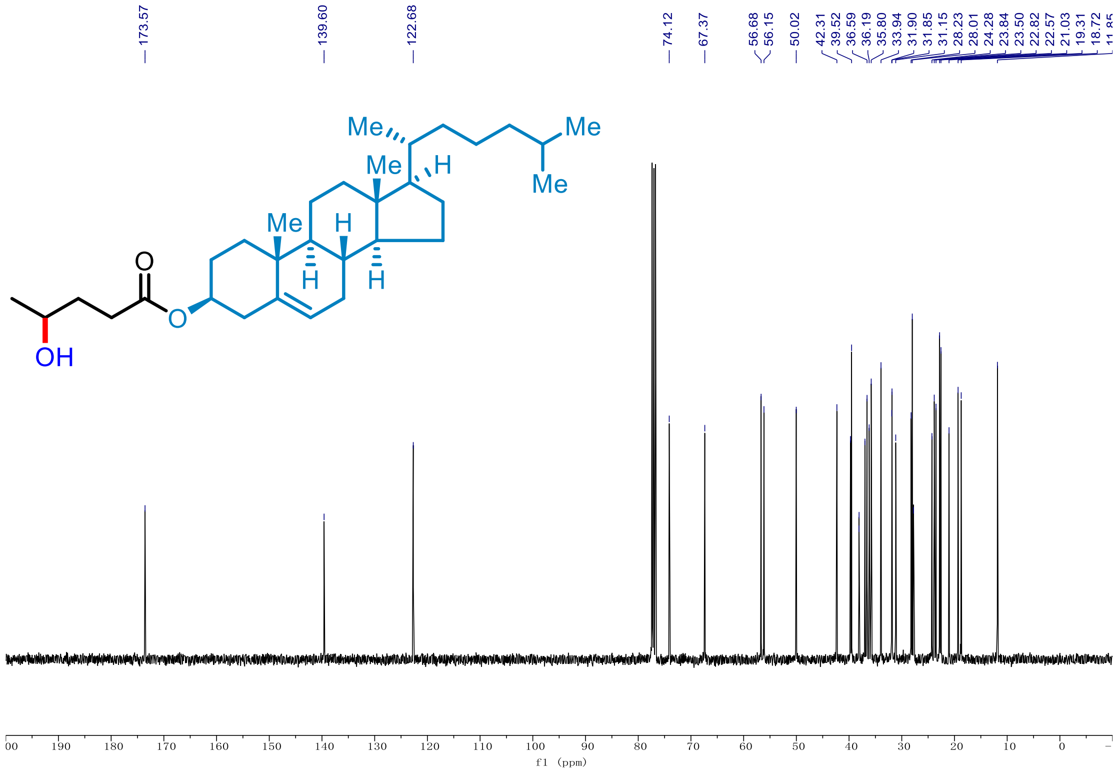

^{1}H NMR spectrum of **50** (**400 MHz, Chloroform-*d***)

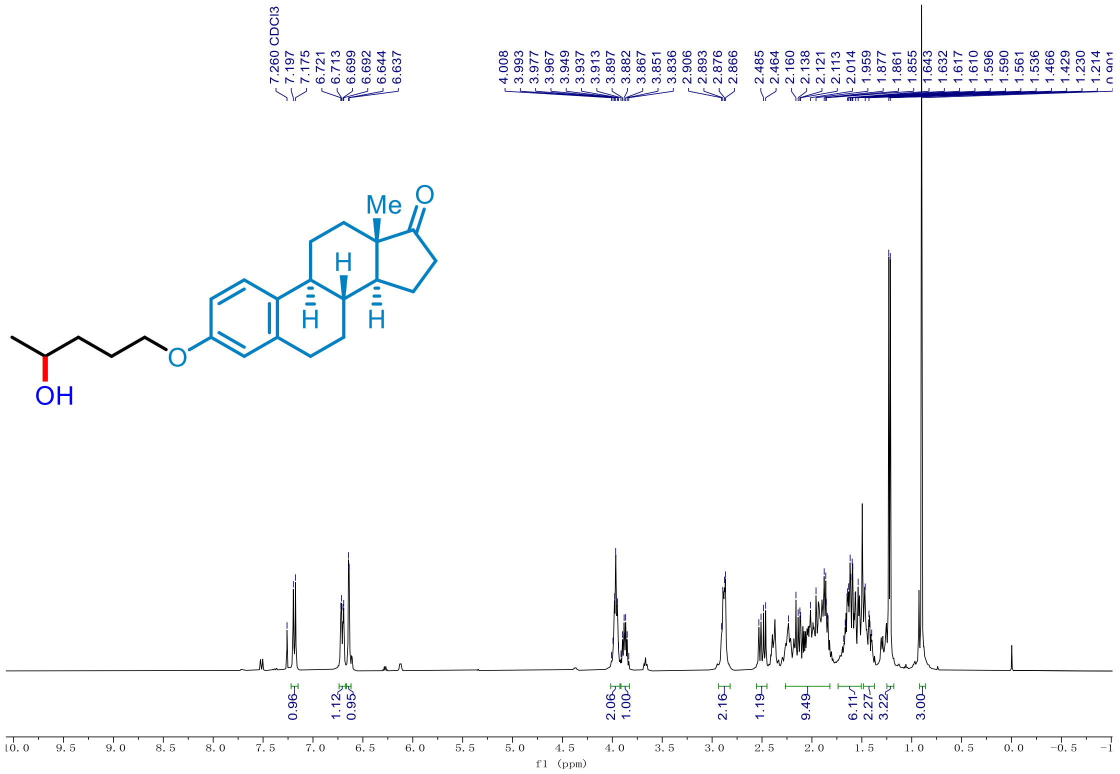

^{13}C NMR spectrum of **50** (**100 MHz, Chloroform-*d***)

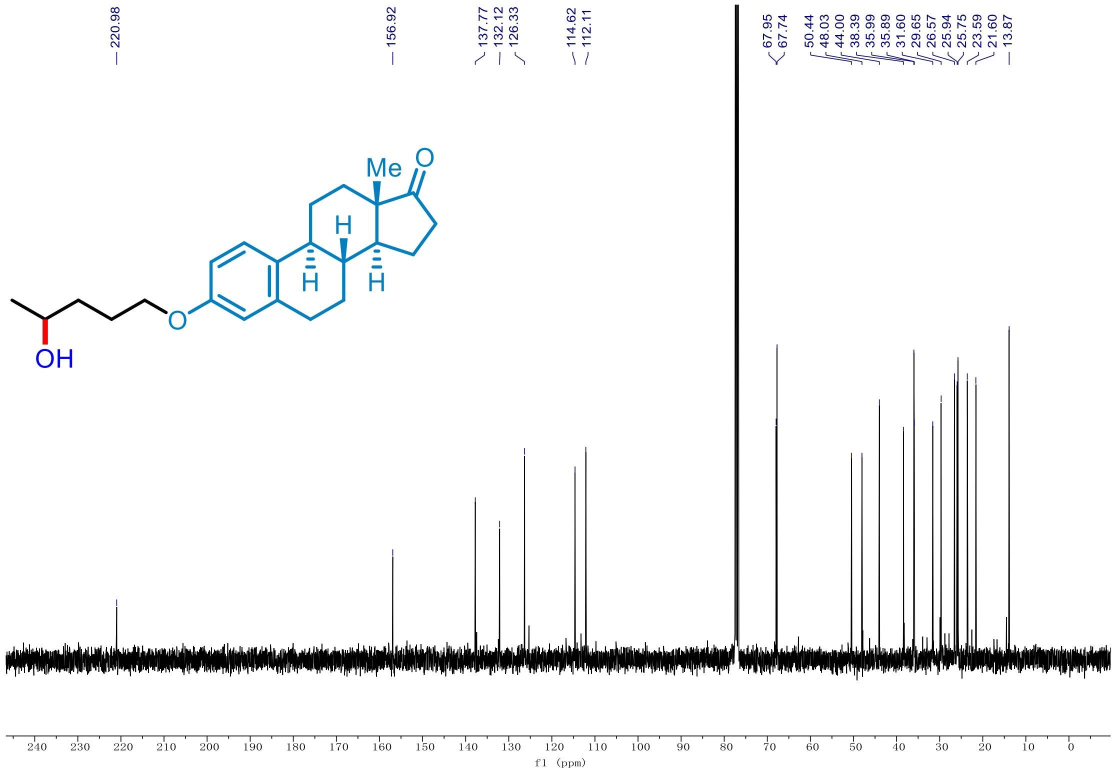

^{1}H NMR spectrum of **51** (**600 MHz, Chloroform-*d***)

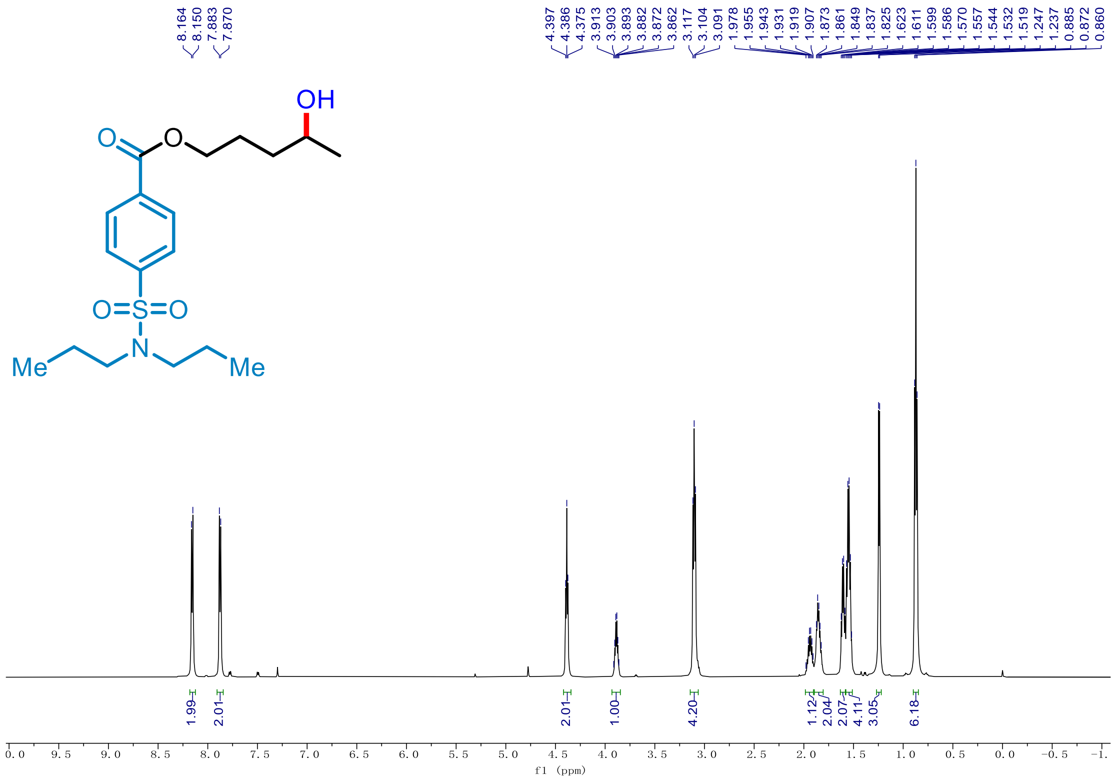

^{13}C NMR spectrum of **51** (**150 MHz, Chloroform-*d***)

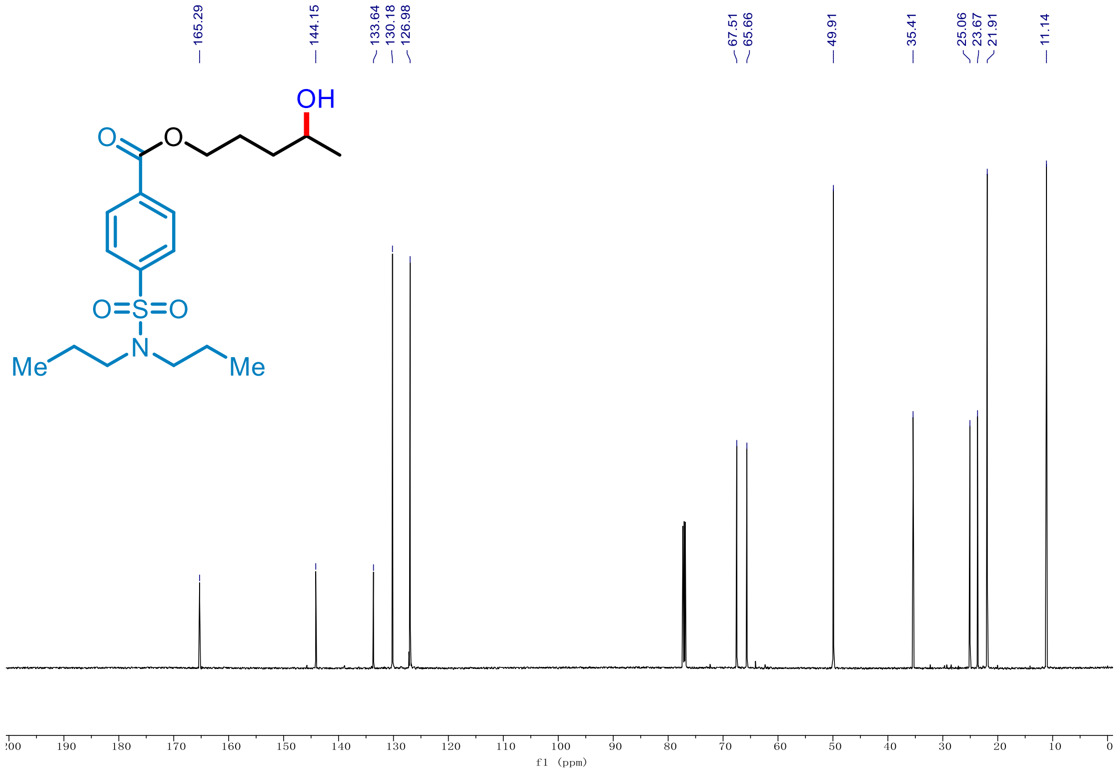

^{1}H NMR spectrum of **52** (**400 MHz, Chloroform-*d***)

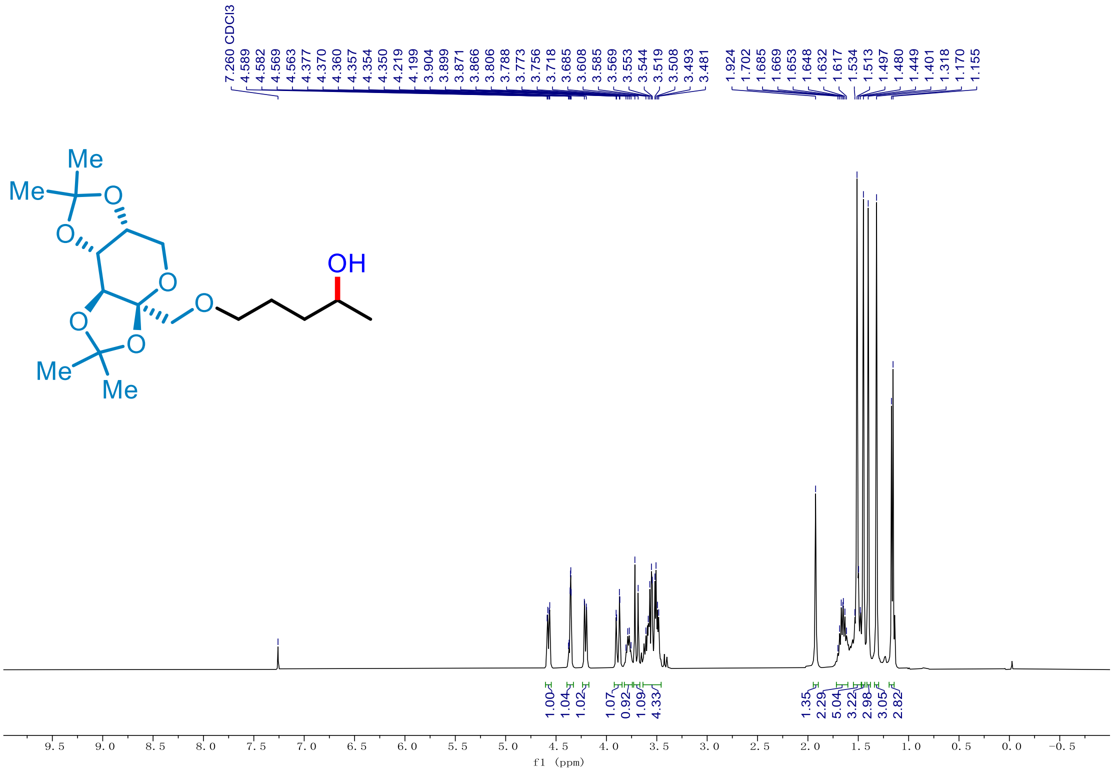

^{13}C NMR spectrum of **52** (**100 MHz, Chloroform-*d***)

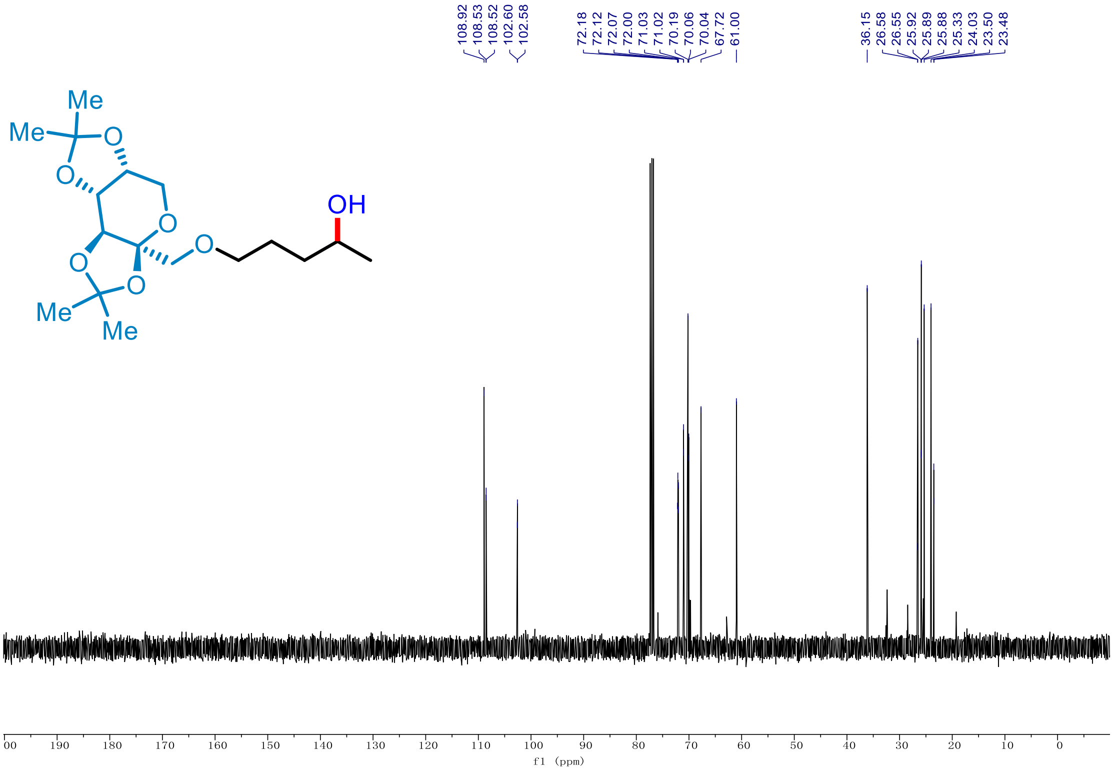

^{1}H NMR spectrum of **53** (**400 MHz, Chloroform-*d***)

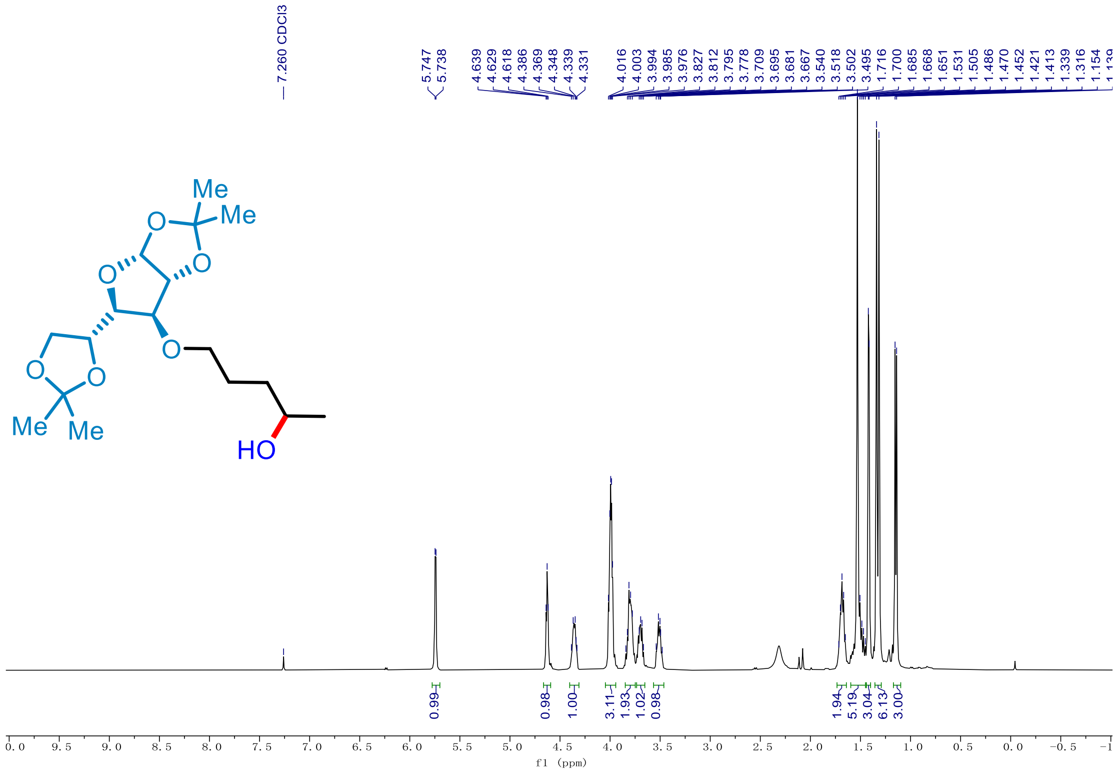

^{13}C NMR spectrum of **53** (**100 MHz, Chloroform-*d***)

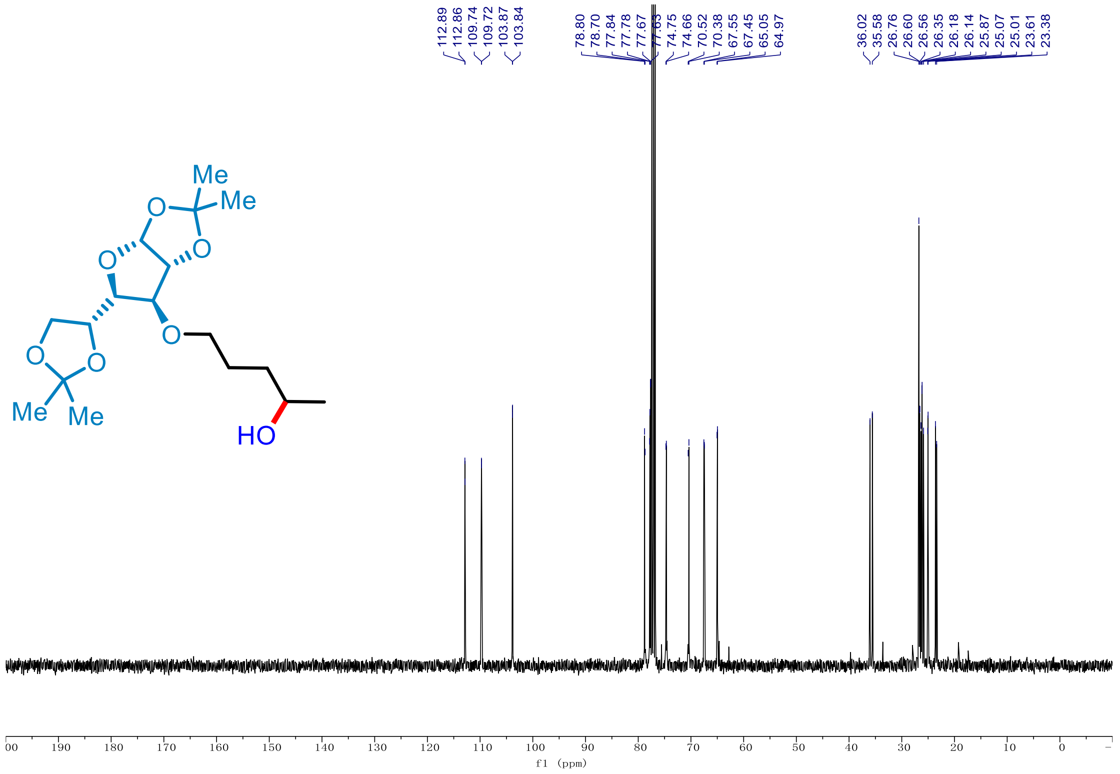

^{1}H NMR spectrum of **54** (**600 MHz, Chloroform-*d***)

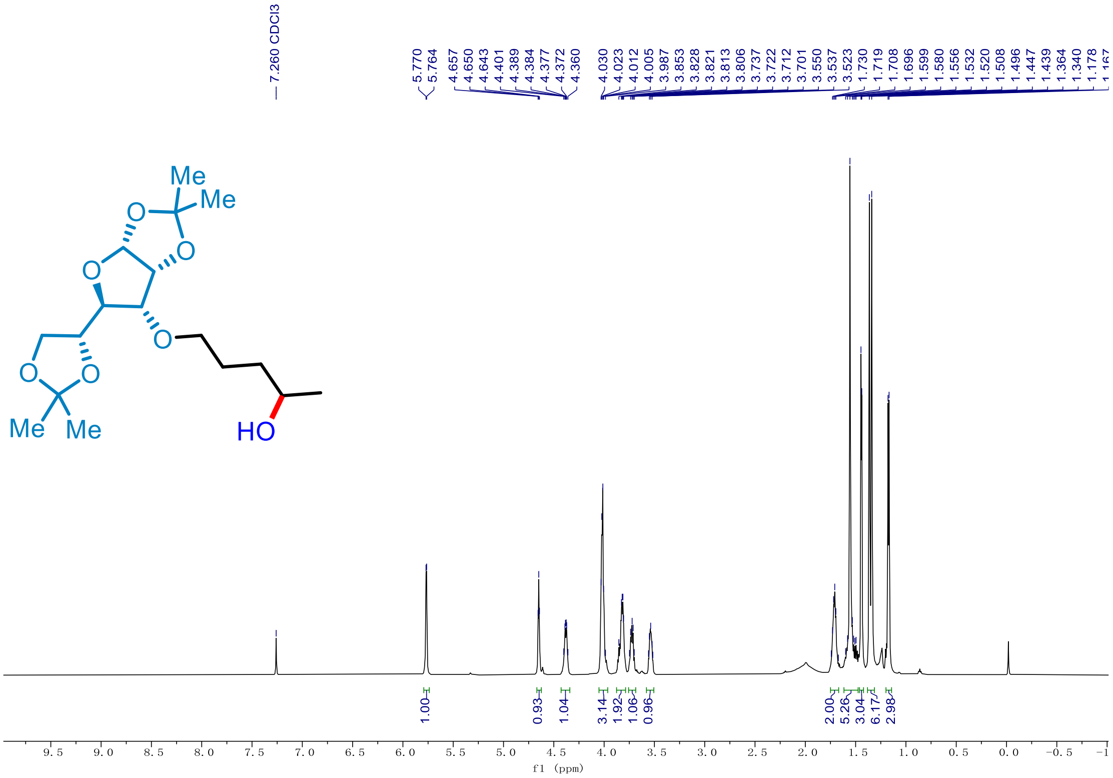

^{13}C NMR spectrum of **54** (**150 MHz, Chloroform-*d***)

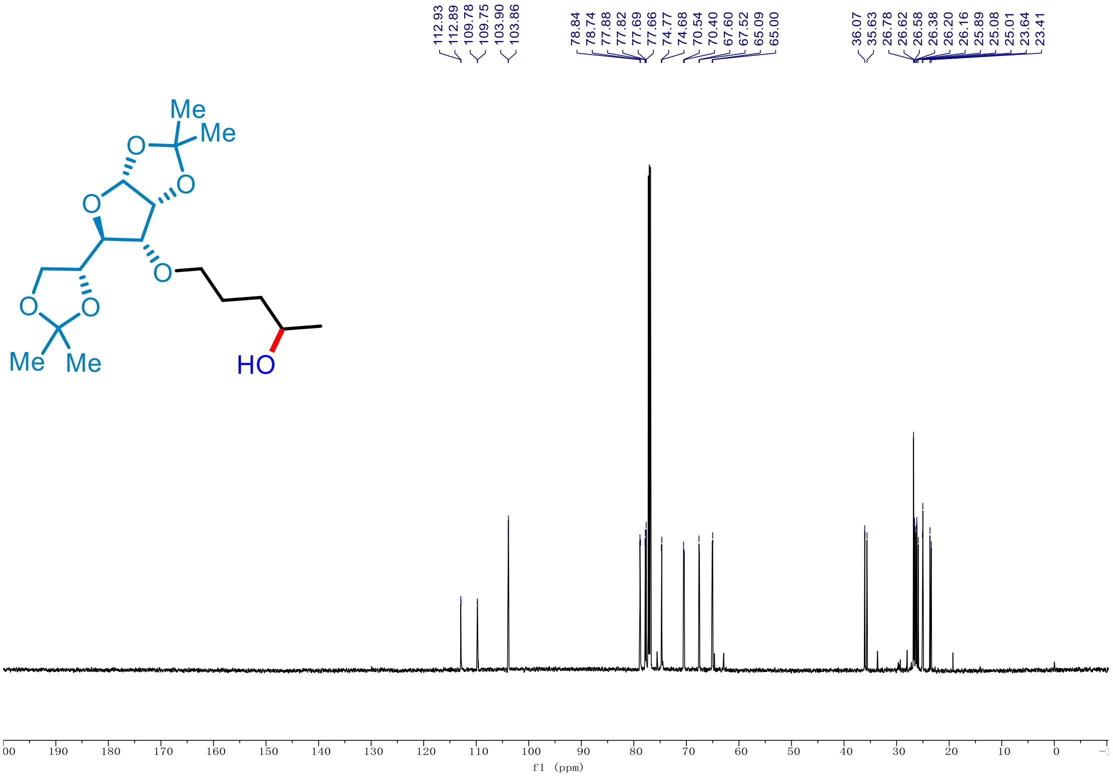

^{1}H NMR spectrum of **55** (**400 MHz, Chloroform-*d***)

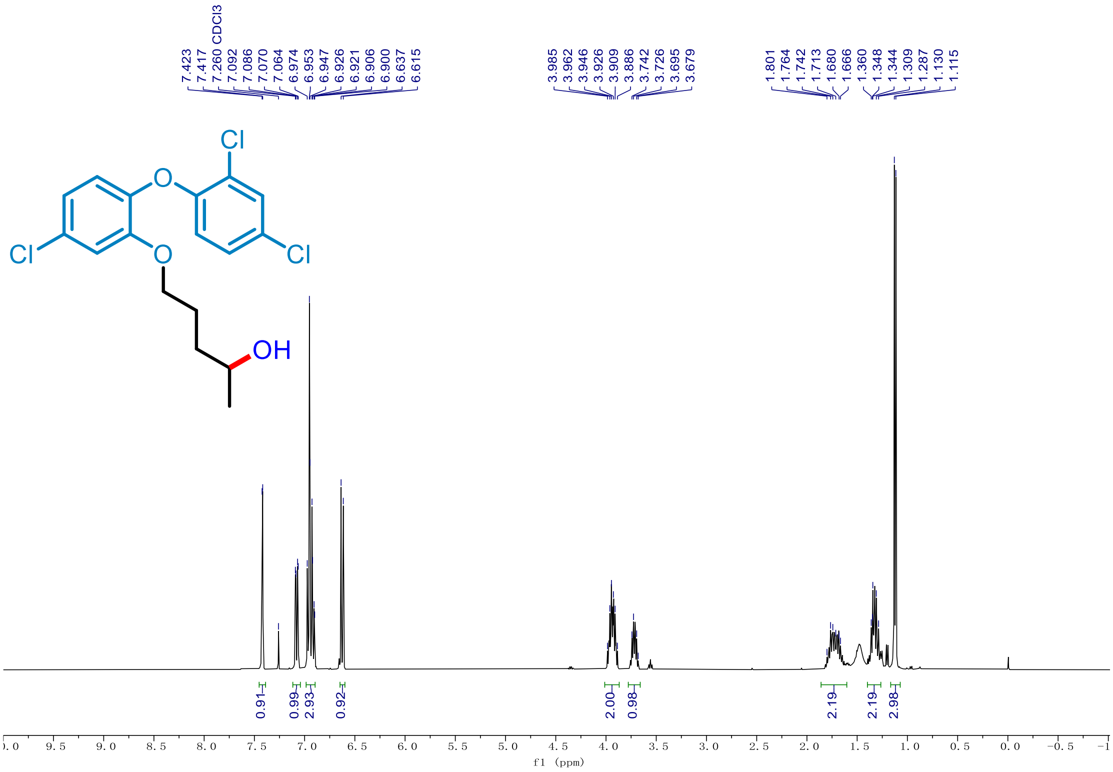

^{13}C NMR spectrum of **55** (**100 MHz, Chloroform-*d***)

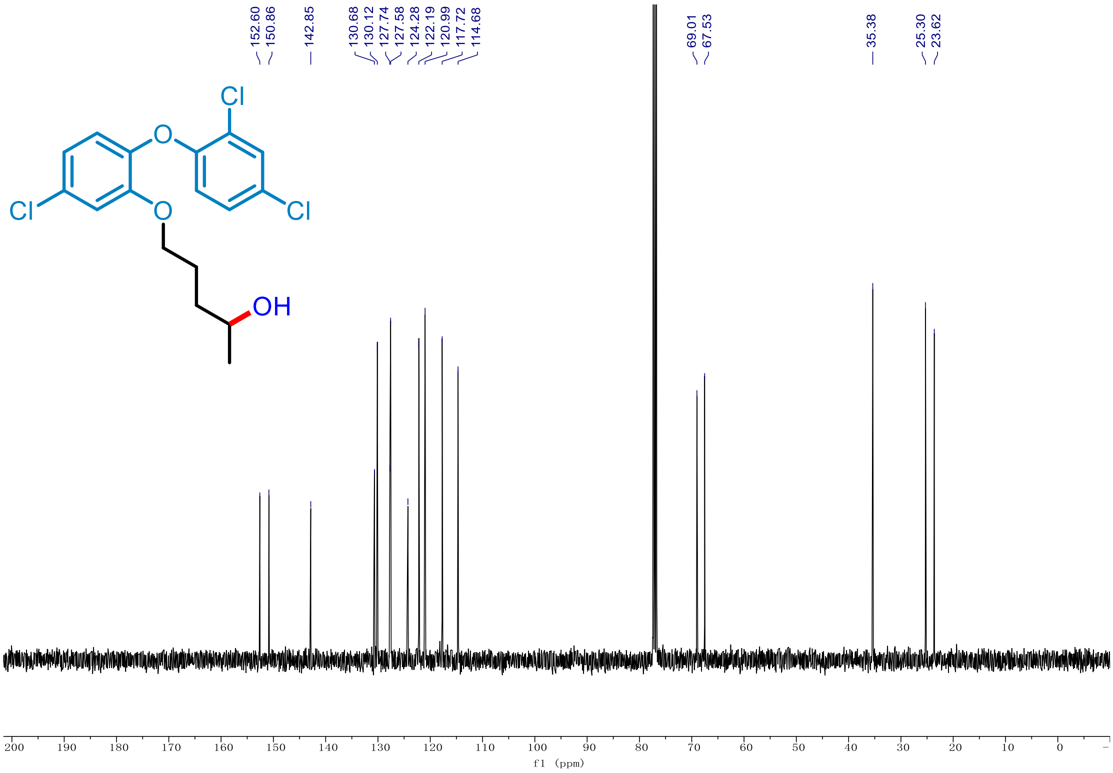

^{1}H NMR spectrum of **56** (**400 MHz, Chloroform-*d***)

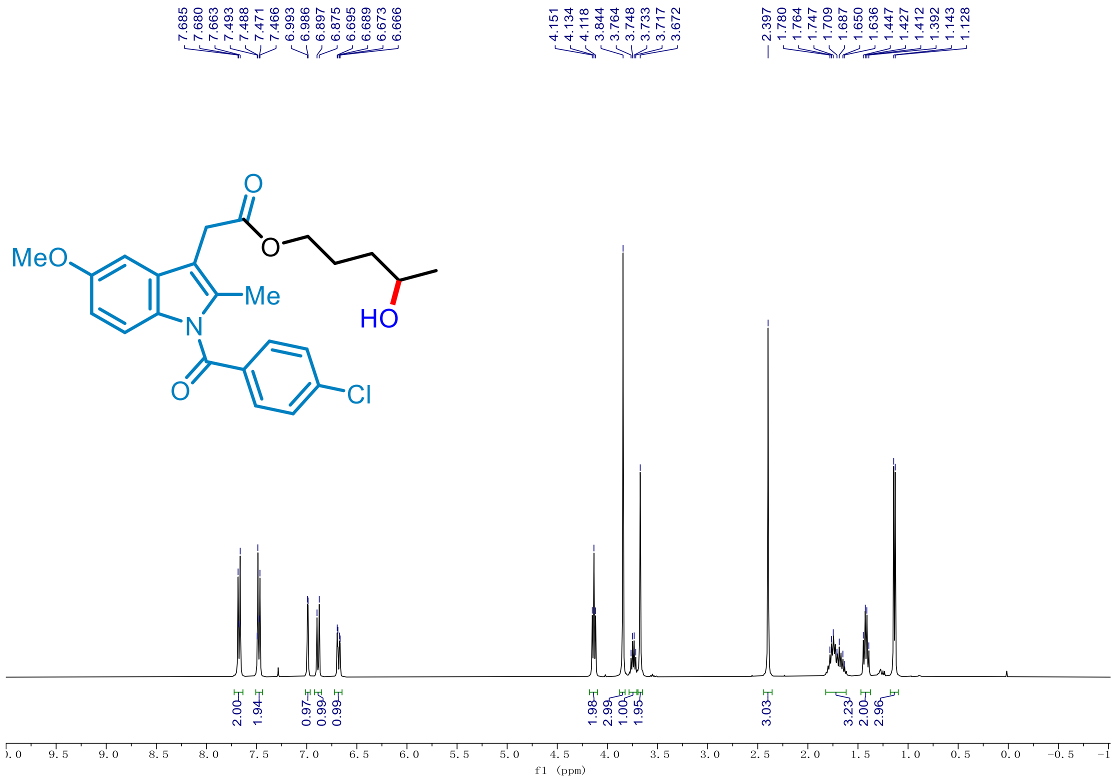

^{13}C NMR spectrum of **56** (**100 MHz, Chloroform-*d***)

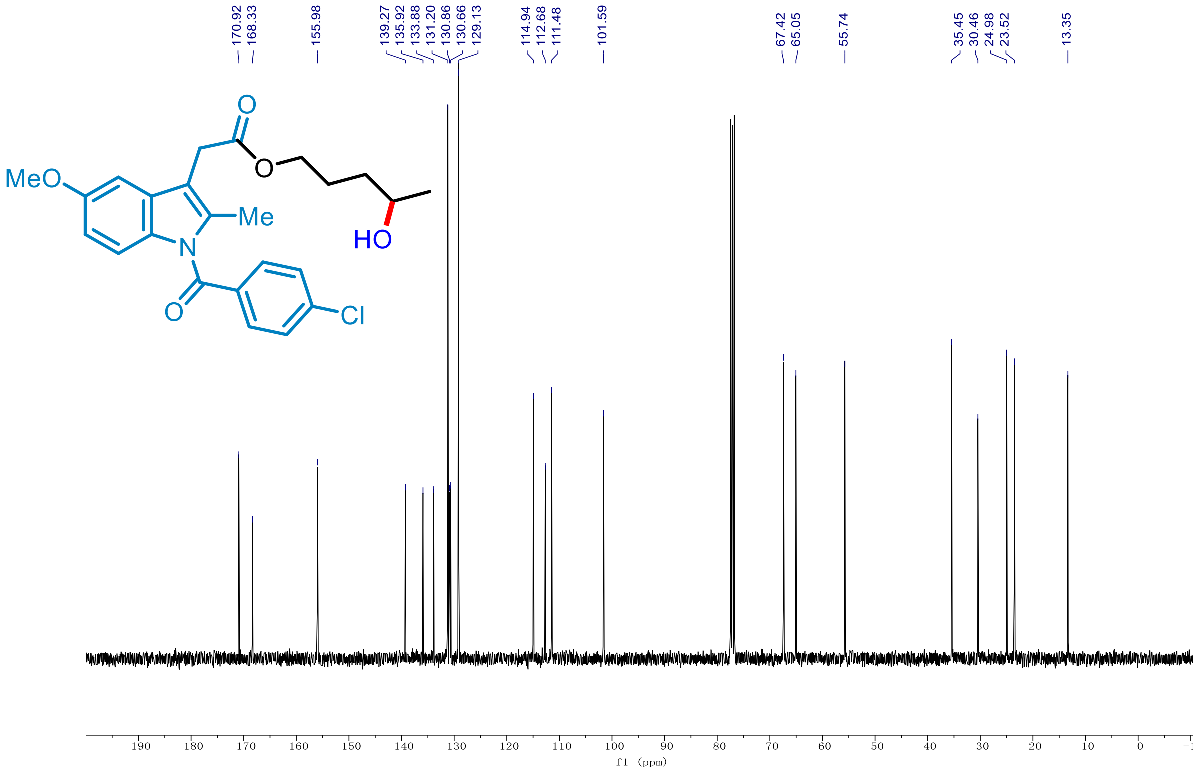

^{1}H NMR spectrum of **57** (**400 MHz, Chloroform-*d***)

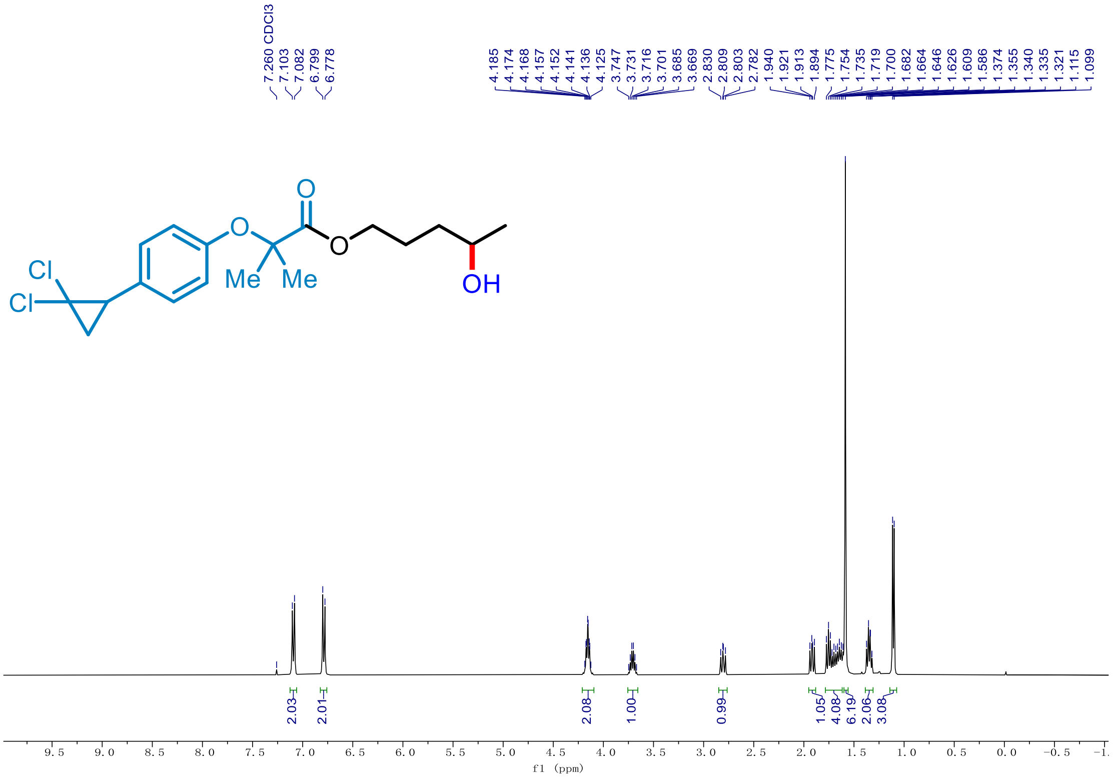

^{13}C NMR spectrum of **57** (**100 MHz, Chloroform-*d***)

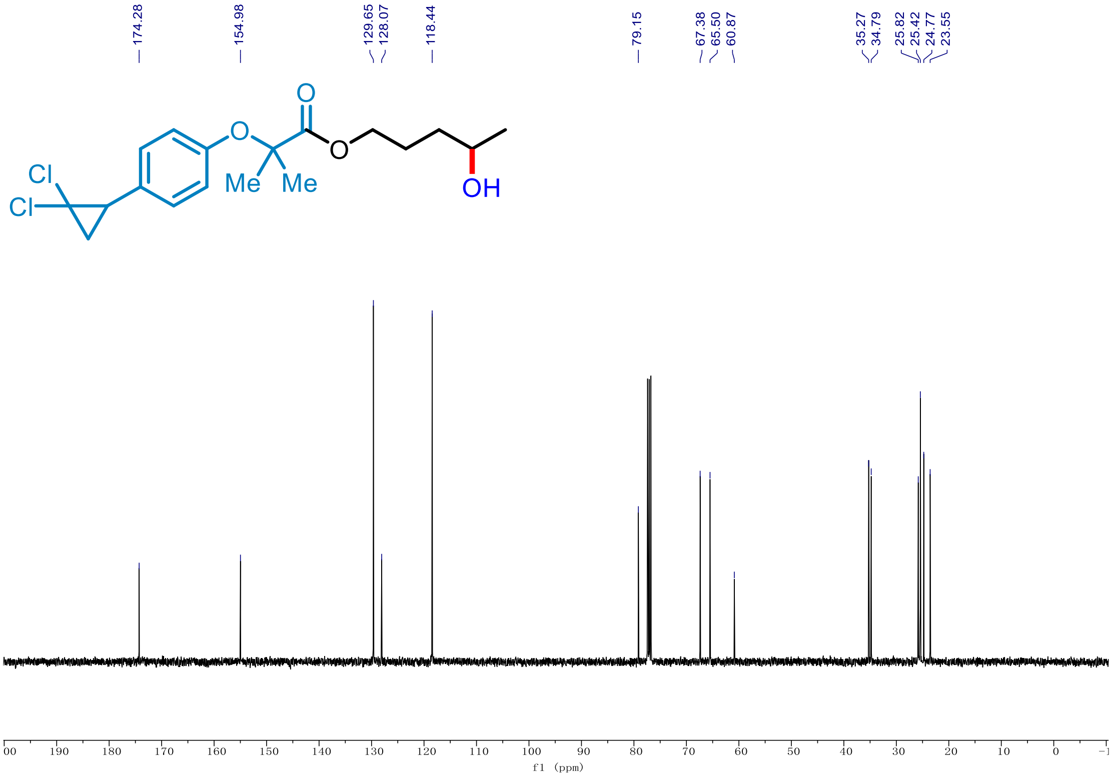

^{1}H NMR spectrum of **58** (**400 MHz, Chloroform-*d***)

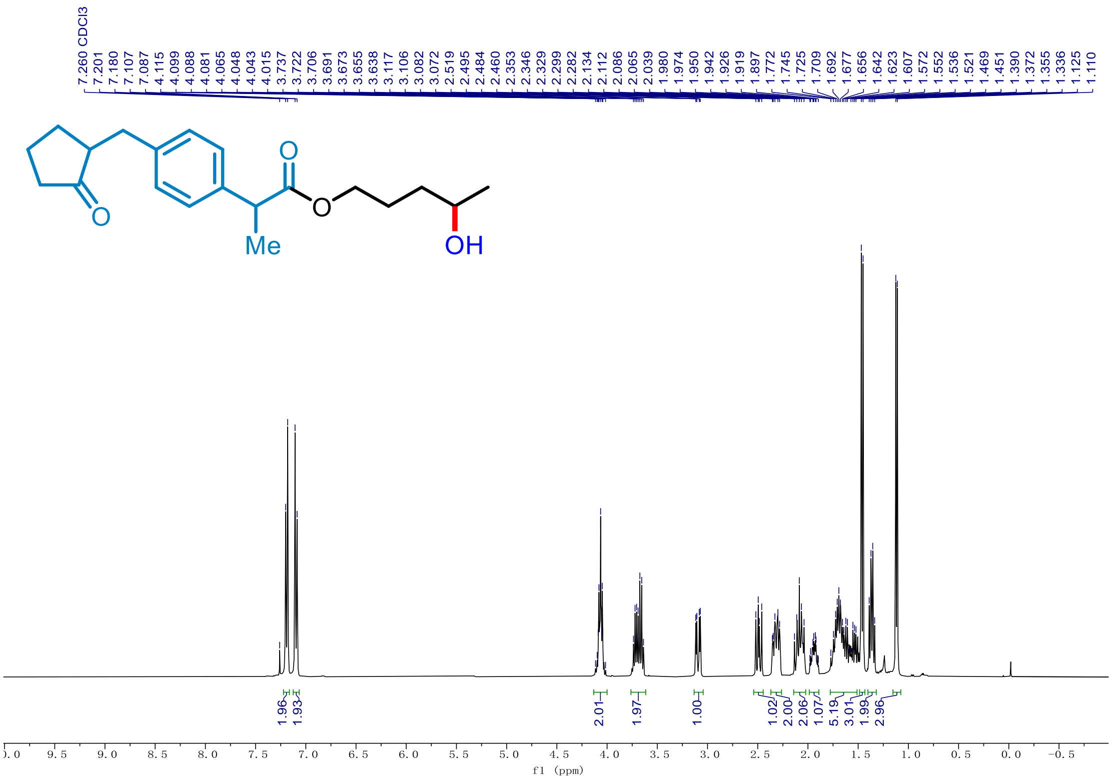

^{13}C NMR spectrum of **58** (**100 MHz, Chloroform-*d***)

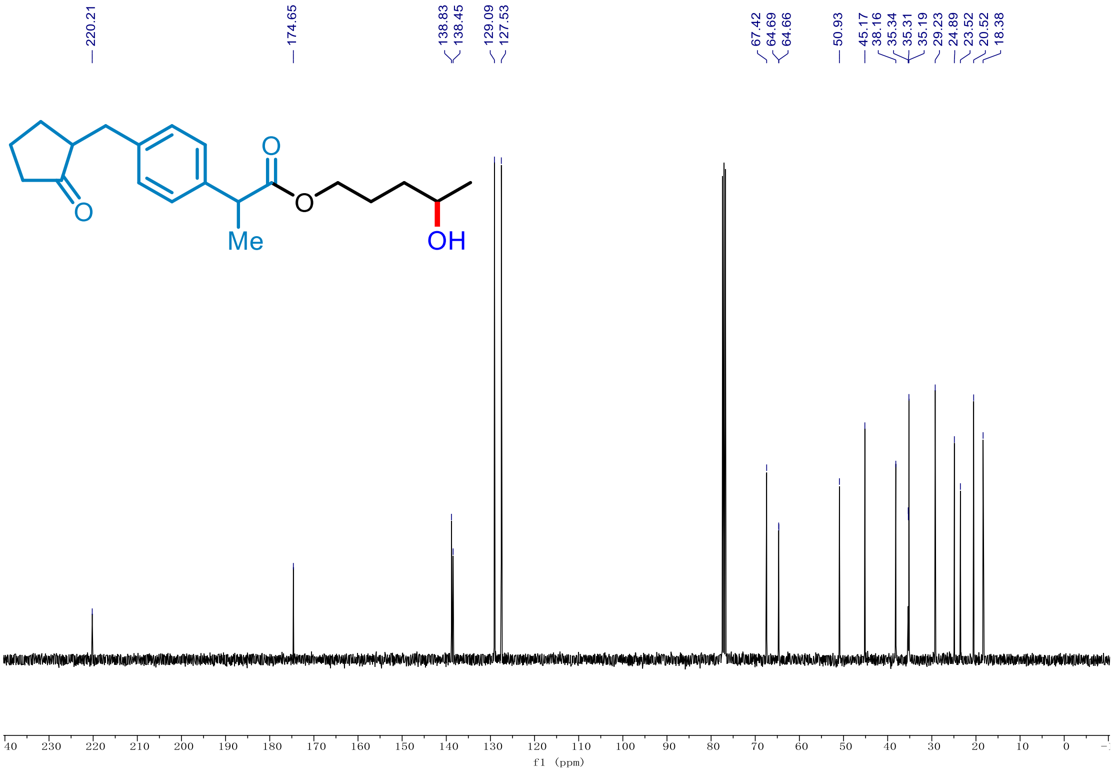

^{1}H NMR spectrum of **59** (**400 MHz, Chloroform-*d***)

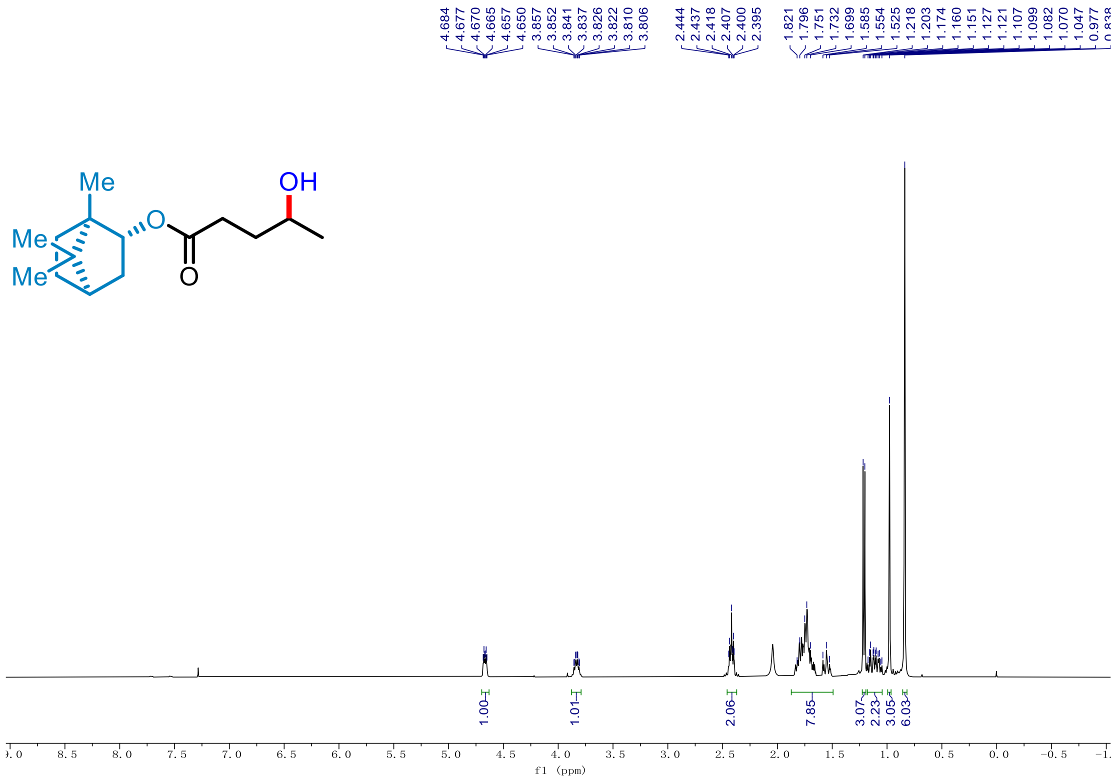

^{13}C NMR spectrum of **59** (**100 MHz, Chloroform-*d***)

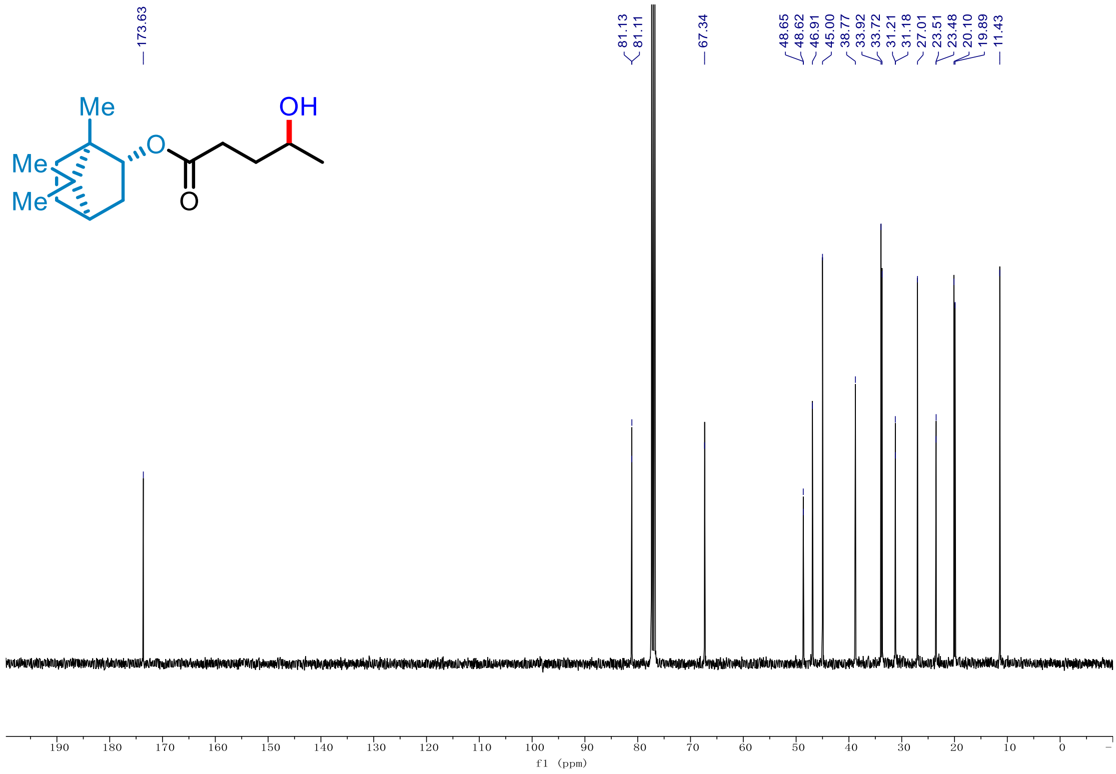

^{1}H NMR spectrum of **60** (**400 MHz, Chloroform-*d***)

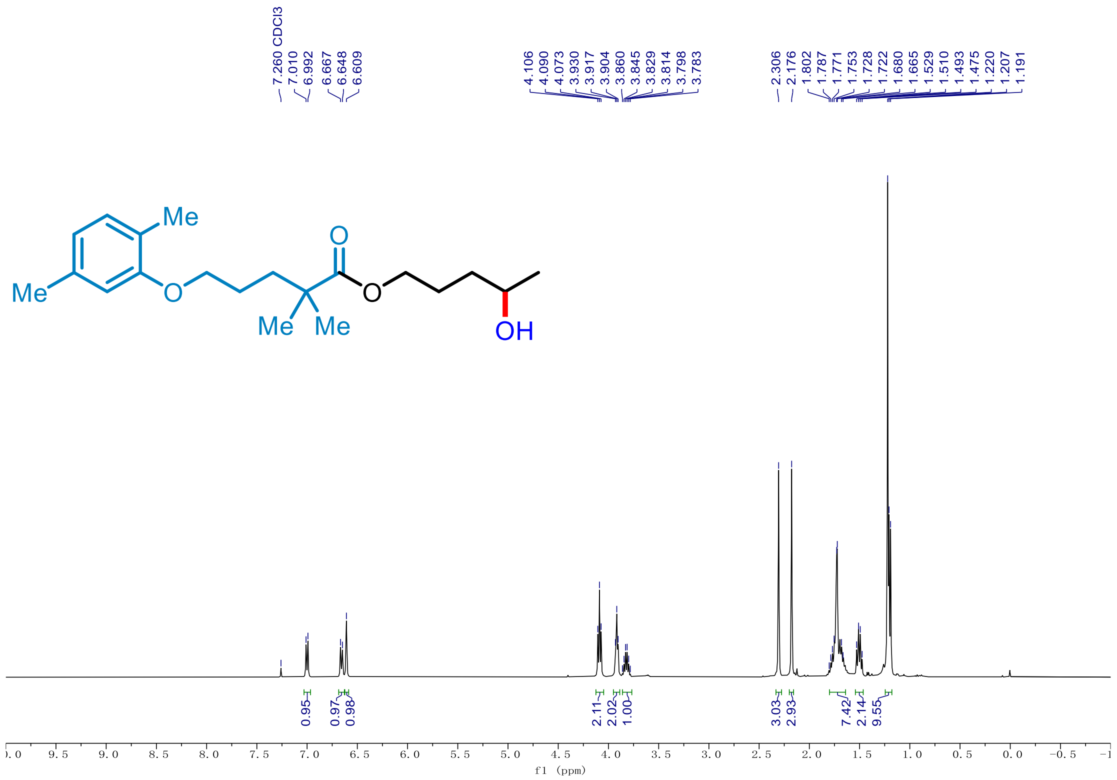

^{13}C NMR spectrum of **60** (**100 MHz, Chloroform-*d***)

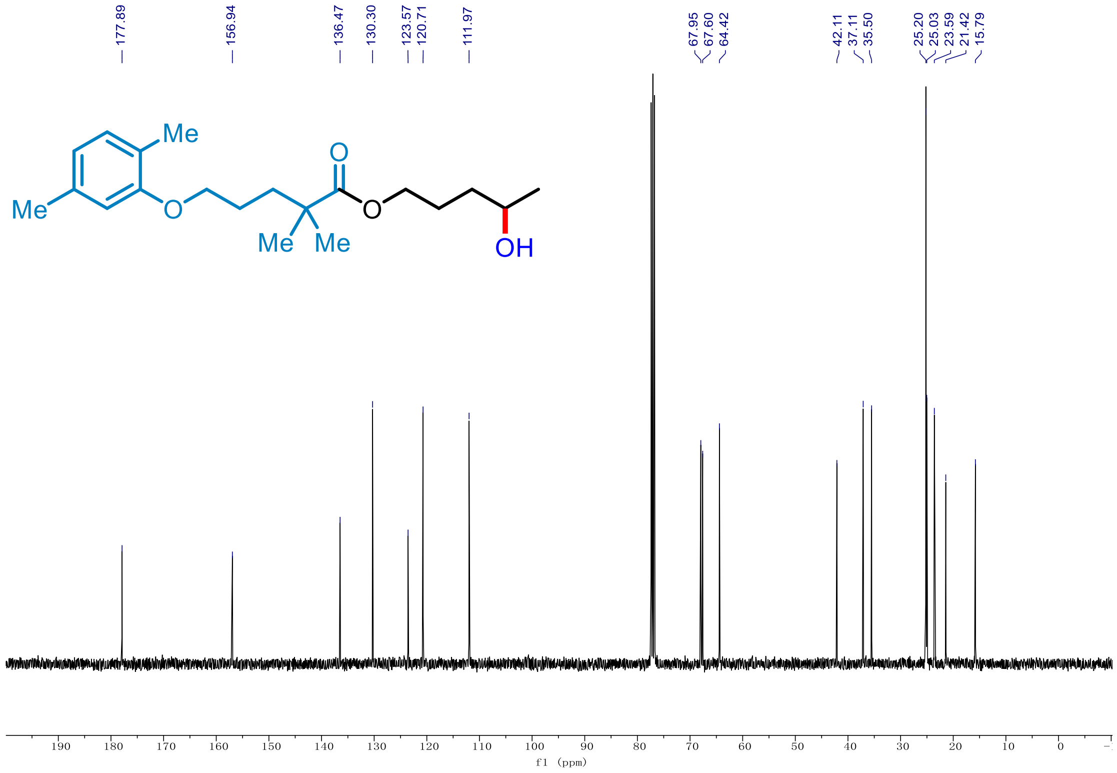

^{1}H NMR spectrum of **61** (**400 MHz, Chloroform-*d***)

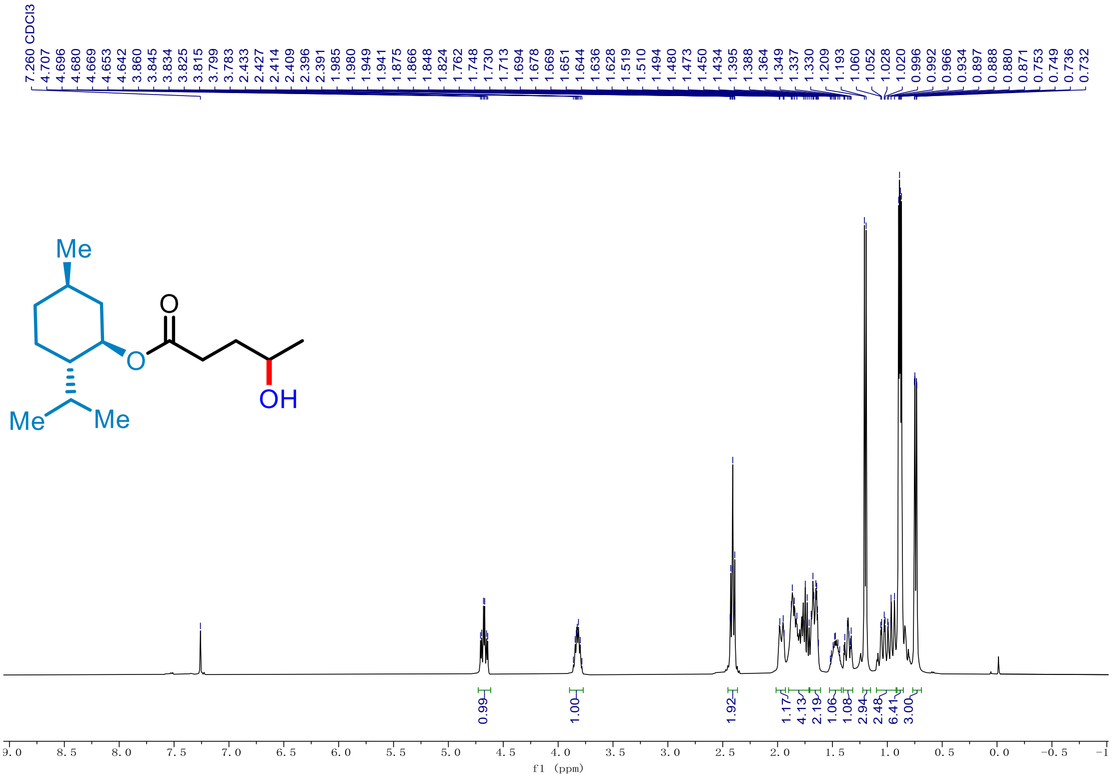

^{13}C NMR spectrum of **61** (**100 MHz, Chloroform-*d***)

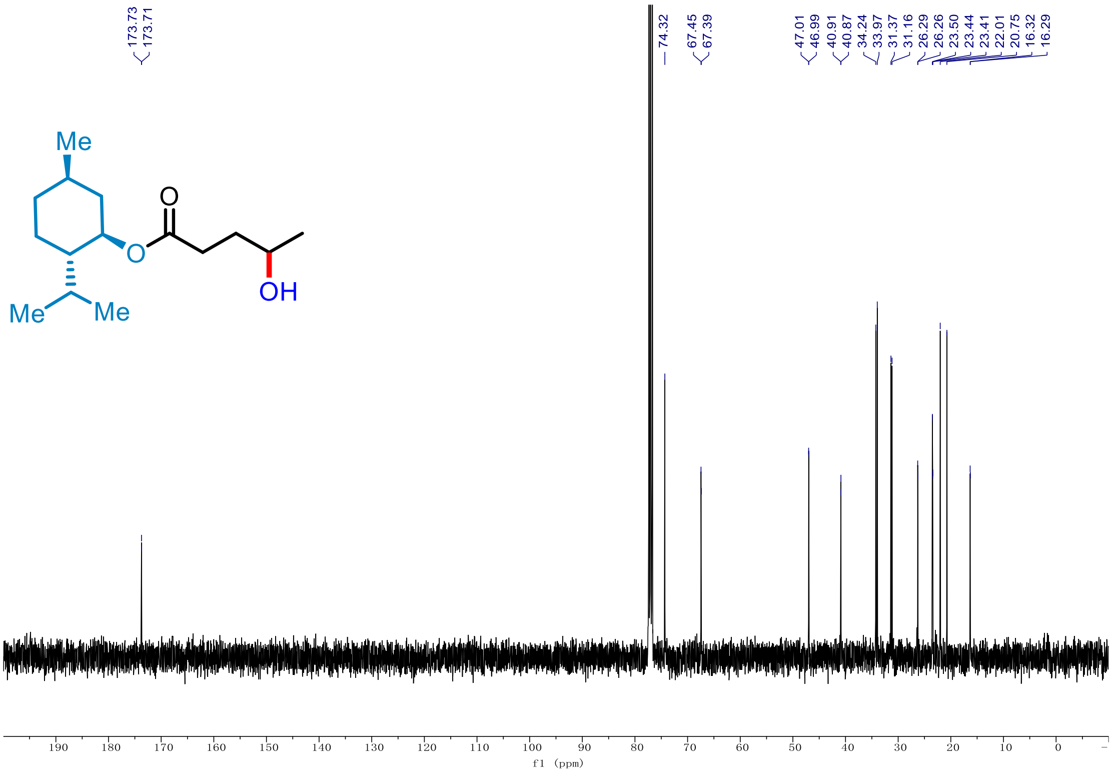

^{1}H NMR spectrum of **62** (**400 MHz, Chloroform-*d***)

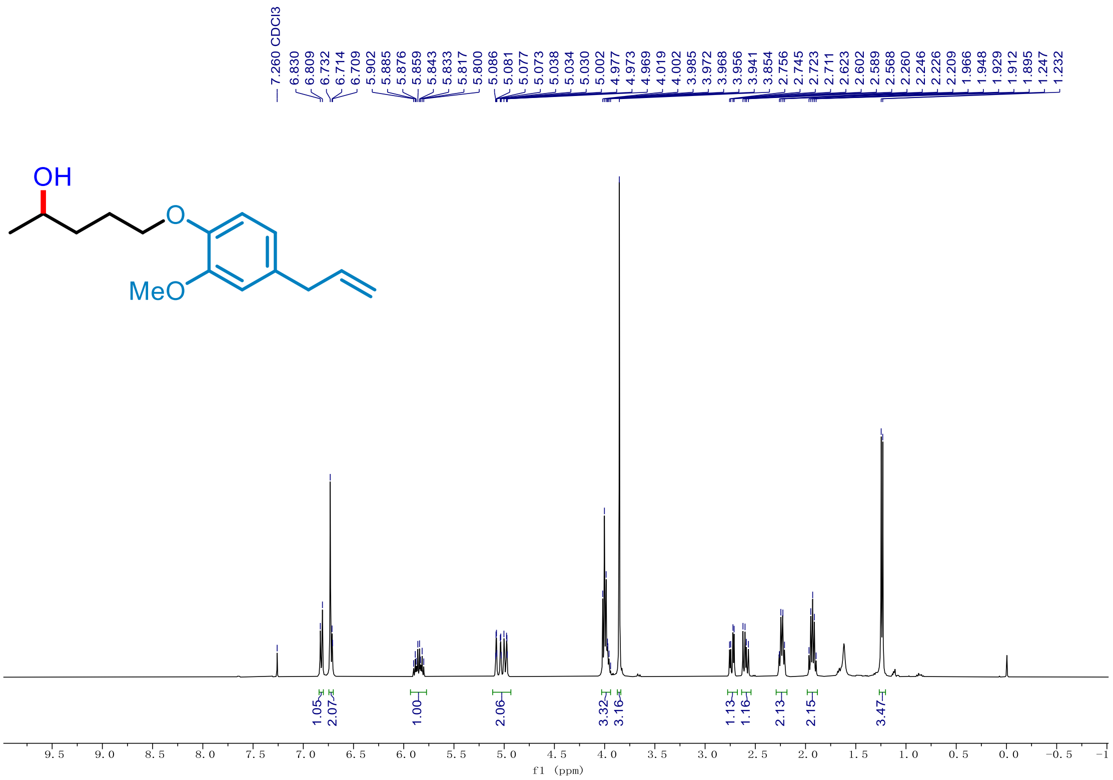

^{13}C NMR spectrum of **62** (**400 MHz, Chloroform-*d***)

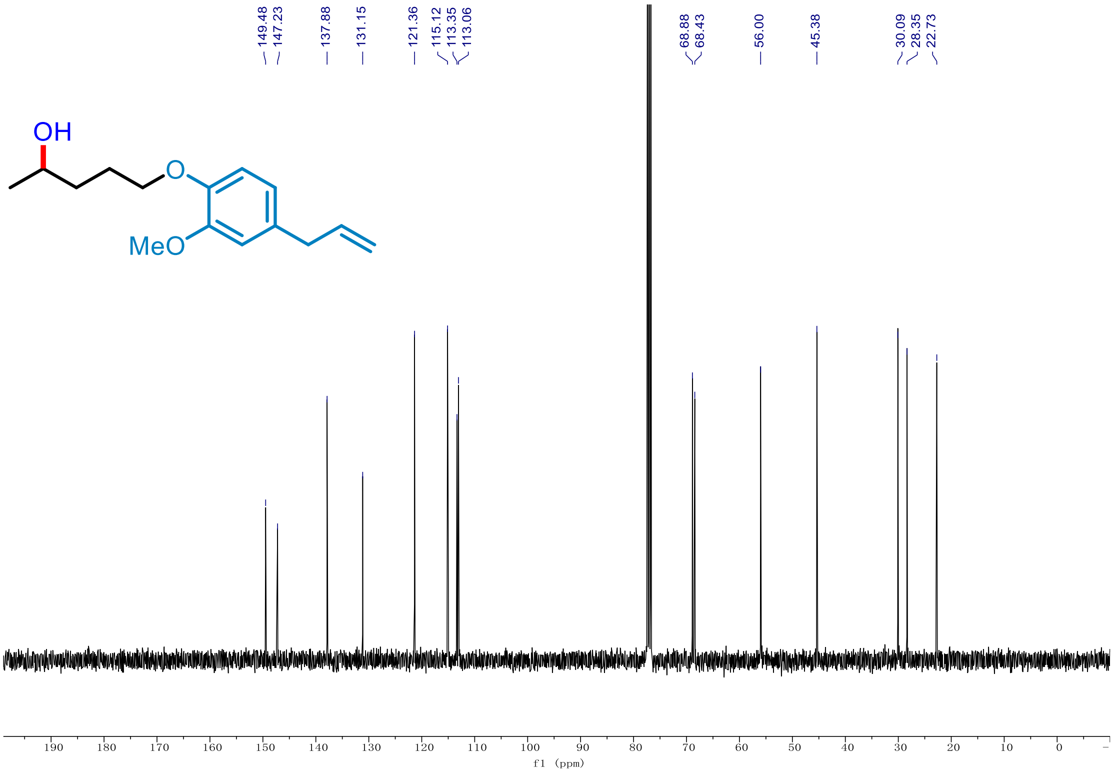

^{1}H NMR spectrum of **63** (**400 MHz, Chloroform-*d***)

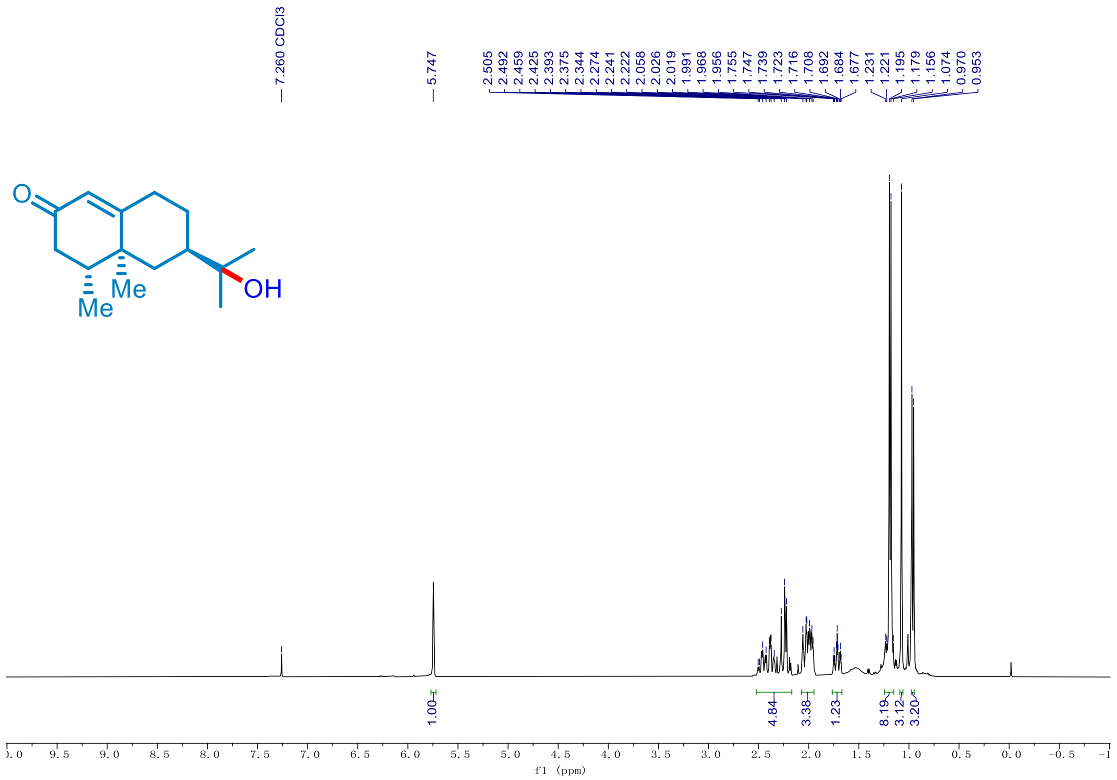

^{13}C NMR spectrum of **63** (**100 MHz, Chloroform-*d***)

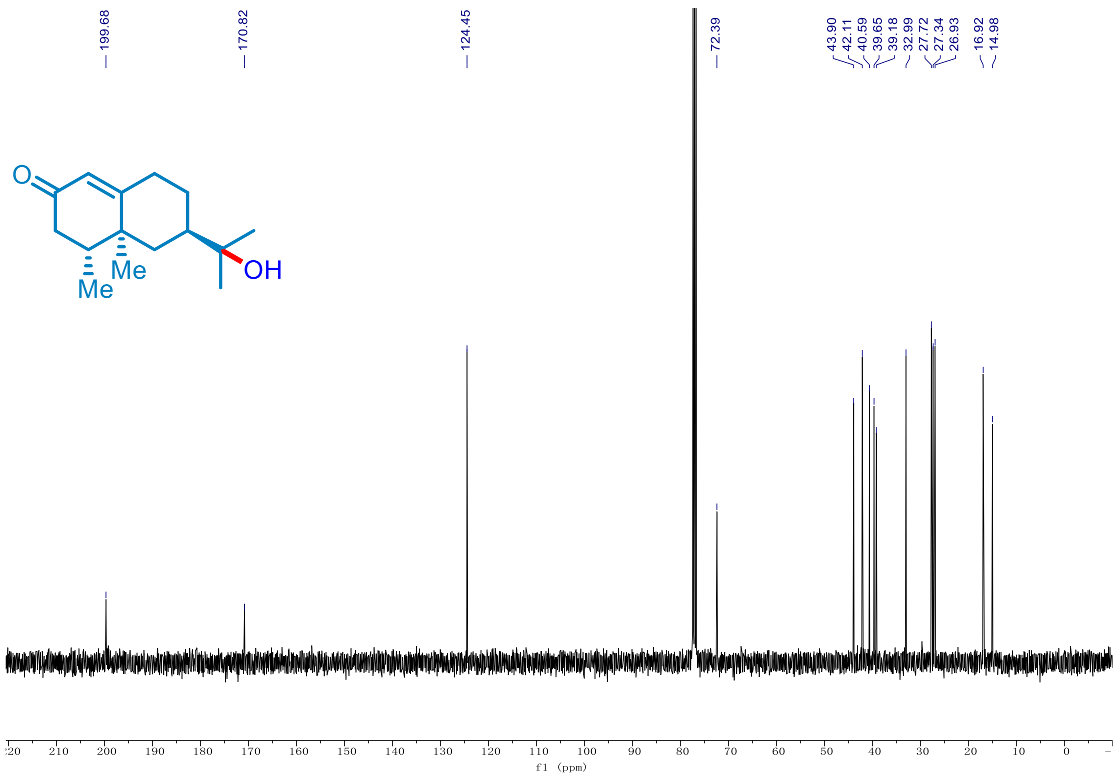

Supplement: nwag047_Supplemental_File [file nwag047_supplemental_file.pdf]
